# Supplementary material for: Implementing primary healthcare-based measurement, advice and treatment for heavy drinking and comorbid depression at the municipal level in three Latin American countries: final protocol for a quasiexperimental study (SCALA study)
Source: BMJ Open. 2020 Jul 28;10(7):e038226. doi: 10.1136/bmjopen-2020-038226 (PMC7390229; doi:10.1136/bmjopen-2020-038226)
Supplement: Supplementary data [file bmjopen-2020-038226supp001.pdf]

Supplement Box 1 Deviations from pre-grant submission pre-protocol

Moving from two-arm to four-arm design In the pre-submission pre-protocol for the quasi-experimental study [1], within each country, two municipal jurisdictions were to be investigator-selected, each with nine primary health care units (PHCU) as part of the study. In one municipal jurisdiction, the intervention municipality, the PHCU would receive both training and municipal support; in the other municipal jurisdiction, the comparator municipality, PHCU would continue practice as usual, with no training or municipal support. The hypothesis was that PHCU in the intervention municipality would measure the alcohol consumption of more patients and give advice to more heavy drinking patients than the PHCU in the comparator municipality.

In the final protocol, within each country, the nine PHCU in the comparator municipality are randomly allocated to five PHCU receiving training (new Arm 2) and four PHCU continuing practice as usual (new Arm 1). The rationale for this approach is that it will enable us to test the independent impact of municipal support over and above just training. The hypothesis to be tested is that PHCU that receive both training and municipal support in the intervention municipality will measure the alcohol consumption of more patients and give advice to more heavy drinking patients than the PHCU who just receive training (Arm 2).

In addition, in the final protocol, within each country, the nine PHCU in the intervention municipality are randomly allocated to four PHCU receiving a standard and longer clinical package and training (new Arm 4) and five PHCU receiving a shorter clinical package and training (new Arm 3), both new Arms 3 and 4 receiving municipal support. The hypothesis to be tested is that the PHCU that receive the standard and longer clinical package and training that is commonly implemented (new Arm 4) will not measure the alcohol consumption of more patients and not give advice to more heavy drinking patients than the PHCU that receive a shorter clinical package and training (new Arm 3). This will be tested over the first six months of the 18-month implementation period, and, if there is non-superiority of Arm 4 over Arm 3, Arm 4 will be collapsed into Arm 3 from month 8 onwards.

Cross-sectional patient self-complete questionnaire instead of prospective interview The deviation is to move from patient follow-up interviews to cross-sectional patient self-completed questionnaires. In the pre-submission pre-protocol, during month 3 of the 18-month implementation period, the first six consecutive screen-negative patients and the first six consecutive screen-positive patients identified by each PHCU were to be invited by the health care provider to give their written consent to complete two follow-up questionnaires, at six months and twelve months after the initial screening. In the final protocol, at two time points, during the 18-month implementation period (months 3 and 15), on two separate days in each of month 3 and 15, providers will seek consent from the patient to self-complete additional questions in the waiting room before leaving the PHCU, handing the completed questions to a researcher in attendance. The rationale for the change is that, primarily due to the nature of the catchments area of patients, it became apparent that it would be impossible to achieve sufficient follow-up rates required for valid analysis of data, with much too high a proportion of country-based resources used in order to try to achieve adequate follow-up rates.

Adjustment in primary outcome indicator The deviation is to change the denominator for the main outcome variable from number of consulting adult patients in a given time period (e.g., one month) to number of registered adult patients. In the pre-submission pre-protocol, the primary outcome was to be the proportion of consulting adult patients (aged 18+ years) intervened (alcohol consumption measured and advice given to heavy drinkers), calculated as the number of AUDIT-C positive patients that received oral advice or referral for advice to another provider in or outside the PHCU, divided by the total number of adult consultations of the participating providers per PHCU. In the final protocol, the primary outcome will be the cumulative proportion of the number of adults (aged 18+ years) registered with the PHCU that have their alcohol consumption measured with AUDIT-C. The rationale is that the revised primary outcome is a measure of coverage, which is considered more intuitive and relevant for health systems change (similar to blood pressure - the proportion of patients that have had their blood pressure measured).

Recalculation of statistical power The change in the main outcome measure required a re-calculation of the statistical power. The study remains adequately powered.

Supplement Table 1 Clinical Package and Training by Study Arm

	Standard package and training (Arm 4)	Shorter package and training (Arms 2 and 3)	Control (Arm 1)
**Instruments**	Short tally sheet: AUDIT-C [2] completed; if AUDIT-C ≥8, AUDIT-10 [3] and PHQ2 [4] completed; if PHQ2 ≥3, PHQ9 [5] completed.	Very short tally sheet: AUDIT-C completed; if AUDIT-C ≥8, PHQ2 completed.	Very short tally sheet: AUDIT-C completed; if AUDIT-C ≥8, PHQ2 completed.
**Provider material**	Provider booklet on alcohol and depression: 43 pages plus 12-page 'quick guide'.	Provider booklet on alcohol and depression: 16 pages.	Provider booklet on alcohol and depression: 11 pages.
**Patient advice and material for alcohol**	Alcohol advice: 5-minute 10-step plan plus 10-page patient brief advice booklet.	Alcohol advice: 1-minute simple advice that the patient needs to drink less, plus 1-page patient brief advice leaflet.	Alcohol advice: 1-minute simple advice that the patient needs to drink less and provide a brief advice leaflet (if available).
	Patient alcohol leaflet: 1 page folded in half to give 4 sides.	Patient alcohol leaflet: 1 page folded in half to give 4 sides.	SCALA patient leaflet on alcohol not given. Provider booklet advises "If available, provide a leaflet on self-management of heavy drinking."
**Patient advice and material for depression**	PHQ9 score 10-14, provide patient leaflet on depression; PHQ 9 ≥14, use clinical judgement to consider if referral is required - if not provide patient leaflet on depression.	PHQ2 ≥3, patient leaflet on depression given.	SCALA patient leaflet on depression not given. Provider booklet advises "If available, provide a leaflet on self-management of depression and action to take if symptoms persist or worsen."
	Patient depression advice leaflet: 1 page, 3 columns.	Patient depression advice leaflet: 1 page, 3 columns.	Present practice.
**Referral**	Referral for very heavy drinking, depression, suicide risk: existing clinical judgement and practice.	Referral for very heavy drinking, depression, suicide risk: existing clinical judgement and practice.	Referral for very heavy drinking, depression, suicide risk: existing clinical judgement and practice.
**Training**	Training: two times two-hours training plus two times one-hour booster sessions (six hours total).<br>Training will take place within	Training: one two-hours training in PHCU, plus one-hour booster session (three hours total).<br>Training will focus on	Present practice.

	the PHCU or clusters of PHCUs. Training will focus on practical skills in undertaking measurement and assessment, and in delivering brief advice, in using the questionnaires, and in knowing when and how to refer patients with more severe heavy drinking and moderately severe or severe depression to available services, such as community-based mental health and addiction centres. Training will, in addition, address attitudes, and perceived barriers and facilitators in implementing measurement and brief advice, contextualized to local circumstances.	practical skills in undertaking measurement and assessment, and in delivering brief advice for harmful alcohol use; instruction of ‘care-as-usual’ + leaflet for depression and severe cases requiring referral. Training will, in addition, address attitudes, and perceived barriers and facilitators in implementing measurement and brief advice, contextualized to local circumstances.	
	Training for both the standard and shorter packages will be undertaken by members of the research team, accredited teachers, or addiction consultants, who will receive a full two-day train-the-trainers session from a senior addiction specialist trainer. The training formats employed are didactic input, guided discussions, skills and practice modeled through videos and role plays. Training sessions are developed from [6-7].		

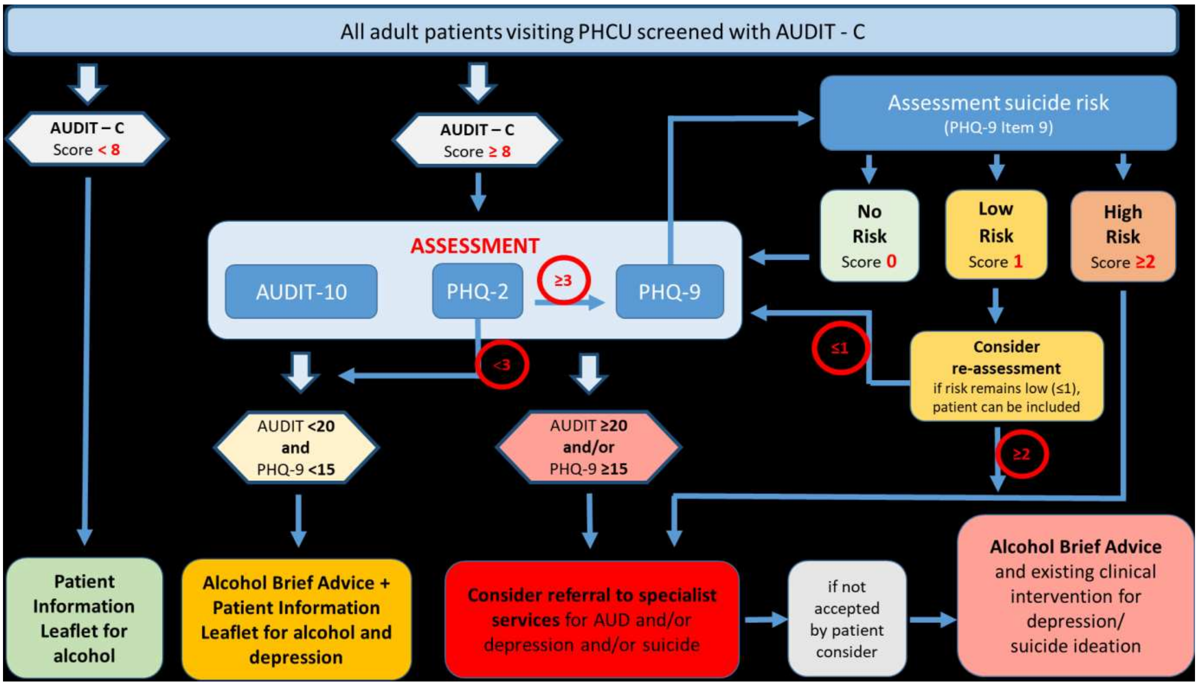

Supplement Figure 1. Standard Care Pathway for Arm 4

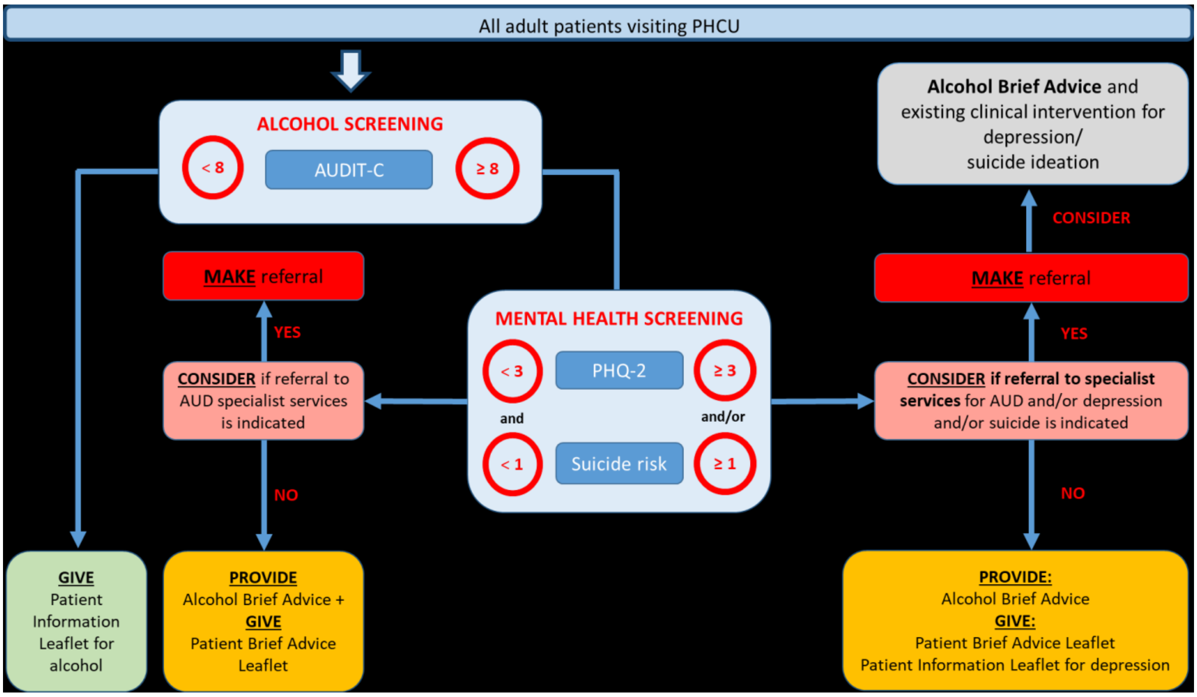

Supplement Figure 2. Short Care Pathway for Arms 1, 2, and 3

Supplement Table 2 Municipal Integration and Support by Study Arm

Intervention Municipal Area (Arms 3 and 4)	Comparator Municipal Area (Arms 1 and 2)
Community Advisory Board (CAB) of local stakeholders set up (including representatives of municipal area, PHCU, health services, non-governmental organizations, academia, media).	Present practice.
User Panel (UP) of local providers and patients set up.	Present practice.
CAB and UP review and tailor relevant materials of clinical package and training courses within the seven domains of: local and national guideline factors; individual health care provider factors; patient factors; interactions between different professional groups; incentives and resources; capacity for organizational change; and, social, political and legal factors [8-10].	Present practice.
CAB reviews barriers and facilitators and potential drivers of successful action [11-12].	Present practice.
CAB identifies potential adoption mechanisms and support systems [13], and reviews plans and components of community-based communication and media campaigns [14-16].	Present practice.
Integrator (champion and knowledge and practice broker) to serve as trusted and accountable leader [13]: facilitating agreement within the municipal area and health systems on shared goals and metrics; assessing and acting on relevant community resources; working at the systems level to make relevant practice changes for sustainability; gathering, analysing, monitoring, integrating, learning, and sharing data at the individual PHCU and city levels; identifying and connecting with system navigators who help PHCUs coordinate, access, and manage multiple services and supports; and developing a system of ongoing and intentional communication with PHCUs and cities.	Present practice.
Adoption mechanisms implemented [13], including: (i) demonstration of the superiority of the PHC package, its simplicity, and its alignment with the latest evidence of preventing and managing heavy drinking and of implementation science; (ii) engagement of identified leaders and building their capacity to lead and ensure broad adoption of the PHC package through guiding and supporting large-scale change; (iii) communicating the value of the PHC package to both municipal and PHC frontline staff; (iv) identifying and adjusting, as appropriate and possible, relevant policies at PHC and city levels to expedite the adoption of the PHC package, for example by adapting electronic health records; and, (v) identifying gaps in health system performance and the urgent need to prevent and manage heavy drinking to promote the needed will and energy to bring implementation of the PHC package to scale.	Present practice.
Support mechanisms implemented [13], including: (i) development of professional capacity for scale-up; (ii) development of infrastructure for scale-up, achieved through redesign rather than addition of new resources; (iii) linking to monitoring and evaluation, using reliable data collection and reporting systems that track and provide feedback on the performance of key processes and outcomes, for example monthly reporting on measurement and brief advice activity; (iv) setting up learning systems to capture change ideas that are shown to result in improved performance assembling ideas into a change package. Knowledge should be shared between municipal actors and PHCUs through regular electronic newsletters and communications; and, (v) creating design factors that enhance sustainability including high reliability of the new processes, inspection systems	Present practice.

to ensure desired results are being achieved, support for structural elements, and ongoing learning systems.	
Communication and media campaign implemented [14-16], including (i) posters, leaflets and/or brochures placed at visible spots in the intervention municipality, e.g., in waiting rooms of PHCUs, health departments, banks, markets; (ii) regular communications, including emails and WhatsApp messages) sent to the healthcare providers and other involved stakeholders in the intervention municipality, (iii) media presence through e.g. articles in local newspapers; interviews, reportages, promotion spots and/or media appearances on local radio, local TV and other local media, and (iv) workshops, forums and/or public local meetings for interested stakeholders such as healthcare providers, representatives of municipal health institutions and patients. All abovementioned activities will focus on reframing that it is heavy drinking that is the problem and that this can be helped to be reduced through primary health care-based measurement and advice programmes, addressing topics such as the harm of hazardous alcohol use in the general population, the (cost)effectiveness and importance of brief alcohol interventions and SCALA success stories.	Present practice.

Supplement Table 3 Data collected at municipal level (if not available, at city, regional or country level)

- Geographical location in city;
- Demographic size of municipal area;
- Indicators of deprivation;
- Information on prevalence of alcohol consumption and related harm;
- Information on prevalence of depression;
- Description of current action to reduce alcohol-related harm;
- Jurisdictional responsibilities for health-related prevention and treatment;
- Structural relationships with primary health care services;
- Structural relationships with hospital-based services;
- Available data mapped to OECD better life initiative [17], including material living conditions (housing, income and jobs) and quality of life (community, education, environment, governance, health, life satisfaction, safety and work-life balance);
- Sustainable Governance Indicators [18], including the Status Index, which 'examines each state's reform needs in terms of the quality of democracy and performance in key policy fields', and the Management Index, focused on 'governance capacities in terms of steering capability and accountability'; and,
- World Values Survey data [19] for cross-cultural variation (Traditional vs. Secular-rational; and, Survival vs. Self-expression).

Jané-Llopis E, *et al. BMJ Open* 2020; 10:e038226. doi: 10.1136/bmjopen-2020-038226

Supplement Table 4 Overview of the measures used in the provider questionnaire

Measure used	Constructs measured
Shortened Alcohol and Alcohol Problems Perception questionnaire [20]	Role security, therapeutic commitment
Abbreviated Maslach Burnout Inventory [21]	Emotional exhaustion, depersonalization, personal accomplishment
Utrecht Work Engagement Scale [22]	Work engagement
Alcohol knowledge [23]	Awareness of drinking guidelines, social norms regarding drinking
Perceived barriers questionnaire [24]	Perceived barriers
Opinion on screening (based on [25])	Pros and cons of screening, social norms of screening, intention to screen
Self-efficacy in delivering the SCALA protocol (based on [26])	Self-efficacy
Context assessment for community health (COACH) tool [27]	Resources, Community engagement, Monitoring services for action, Work culture, Leadership
Evaluation of SCALA community action [15]	Exposure to campaign/adoption mechanisms/support systems, perceptions of campaign/adoption mechanisms/support systems
Attributes of innovation questionnaire [28]<br>- Only intervention group	Relative advantage, Compatibility, Complexity, Trialability and Observability
Experienced barriers (based on the driver diagram [12])<br>- Only intervention group	Experienced barriers

Supplement Table 5. Country-level collection of economic data for return-of-investment analyses

**Costs of Investment**		**Gains of investment**	
Cost unit	***Data source***	***Cost unit***	***Data Source***
Cost of providing training and booster sessions to PHCU staff	Time and materials required, documented by study team	Costs and utilization of *primary health care* (number of visits) by major disease/injury categories	National statistics, ministry of health, local researchers, or other publications
Setting up and maintaining Community Advisory Boards and User Panels	Time and materials required, documented by study team	Costs and utilization of *emergency* facilities (number of admissions) by major disease/injury categories	National statistics, ministry of health, local researchers, or other publications
Direct costs for implementing the clinical pathway (routine measurement, further assessment, brief interventions, referral)	Staff salary and time required, documented by PHCU administration and providers	Costs and utilization of *inpatient* facilities (number of admissions, length of stay) and of *outpatient* facilities (number of admissions) by major disease/injury categories	National statistics, ministry of health, local researchers, or other publications
Additional costs for implementing the clinical pathway	Documented by PHCU administration	Avoided mortality	National statistics, ministry of health, local researchers, or other publications

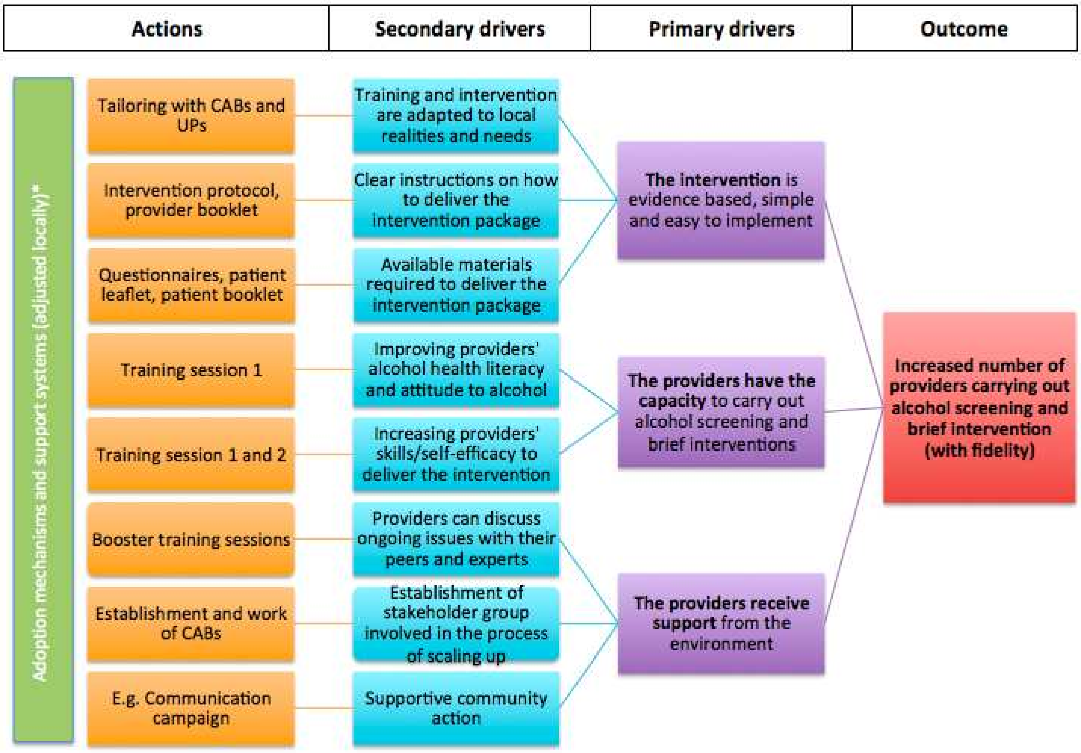

Supplement Figure 3. Driver diagram of the SCALA protocol

Supplement Table 6 Process evaluation topics based on MRC framework [29]

Part of process evaluation		Topic of investigation	Method
**Description of the intervention**		The description of the intervention and its causal assumptions	Driver diagram
**Implementation**	*Adaptation*	Experience of intervention tailoring	Key informant interview
		Experience with training tailoring	Key informant interview
	Dose delivered (completeness of delivery)	Implementation of the protocol (number of measurements, brief advice given, referrals done)	Tally sheets
		Length of implemented training	Observation
		Implementation of adoption mechanisms and support systems on municipal and organisational level	Key informant interview, Document analysis
		Implementation of CAB meetings	Observation, document analysis
		Implementation of communication campaign	Key informant interview, document analysis
	Fidelity (quality of implementation)	Following the care pathway as intended	Tally sheets, patient questionnaire
		Training active ingredient delivery	Observation
	Reach	Number of patients and providers involved	Document analysis
		Number of providers attending the training	Document analysis
**Mechanisms of impact**	*Participant responses*	Patients' perception of acceptability of intervention	Patient questionnaire
		Providers' satisfaction with the training	Post-training questionnaire
		Providers' perceived utility of training sessions	Post-training questionnaire
		Perception of the intervention	Key informant interview
		Perception of the campaign	Provider questionnaire, patient questionnaire
		Perception of the municipal action	Key stakeholder interview
	Mediators	Influence of training on attitude and self-efficacy	Provider questionnaire
		Influence of communication campaign on beliefs and social norms	Provider questionnaire
		Perception of the attributes of the intervention	Provider questionnaire
	Unintended consequences	Possible unexpected side effects emerging	Key stakeholder interview
**Context**		Perceptions of organisational context	Provider questionnaire
		Individual moderating characteristics	Provider questionnaire
		Description of organisational context changes	Key informant interview, logbook
		Contextual factors influencing training	Observation, key informant interview
		Contextual factors influencing municipal action	Key informant interview, document analysis
**Outcomes**		Integration of process evaluation information with the results of the outcome evaluation	Integration of data collected through abovementioned methods with the tally sheet data

Supplement Table 7 Completed seven-point checklist for SCALA study design [30]

Quality Measure	SCALA
1.Was the intervention/(answer "yes" to more than 1 item, if applicable)	
Allocated to (provided for / administered to / chosen by) individuals?	No
Allocated to (provided for / administered to / chosen by) clusters of individuals?	No
Clustered in the way it was provided (by practitioner or organisational unit)?	YES
**2. Were outcome data available: *(answer "yes" to only 1 item)***	
After intervention / comparator only (same individuals)?	-
After intervention / comparator only (not all same individuals)?	-
Before (once) AND after intervention / comparator (same individuals)?	YES
Before (once) AND after intervention / comparator (not all same individuals)?	-
Multiple times before AND multiple times after intervention / comparator(same individuals)?	-
Multiple times before AND multiple times after intervention / comparator (not all same individuals)?	-
**3. Was the intervention effect estimated by: *(answer "yes" to only 1 item)***	
CHANGE OVER TIME (same individuals at different time points)?	-
CHANGE OVER TIME (not all same individuals at different time points)?	-
DIFFERENCE BETWEEN GROUPS (of individuals or clusters receiving either intervention or comparator)?	YES
**4. Did the researchers aim to control for confounding (design or analysis) *(answer "yes" to only 1 item)*:**	
Using methods that control in principle for any confounding?	-
Using methods that control in principle for time invariant unobserved confounding?	-
Using methods that control only for confounding by observed covariates?	YES
**5. Were groups of individuals or clusters formed by *(answer "yes" to more than 1 item, if applicable)*:**	
· Randomization?	No
· Quasi-randomization?<br>· Explicit rule for allocation based on a threshold for a variable measured on a continuous or ordinal scale or boundary (in conjunction with identifying the variable dimension, below)?	No
· Some other action of researchers?	YES
· Time differences?	No
· Location differences?	YES
· Healthcare decision makers / practitioners?	No
· Participants' preferences?	No
· Policy maker	No
· On the basis of outcome?	No
· Some other process? (specify)	No
**6. Were the following features of the study carried out after the study was designed *(answer "yes" item, if applicable)*: *to more than 1***	
Characterization of individuals / clusters before intervention?	YES
Actions/choices leading to an individual/cluster becoming a member of a group?	YES
Assessment of outcomes?	YES

**7. Were the following variables measured before intervention:** ***(answer "yes" to more than 1 item,*** **If applicable)**	
Potential confounders?	YES
Outcome variable(s)?	YES

Jané-Llopis E, *et al. BMJ Open* 2020; 10:e038226. doi: 10.1136/bmjopen-2020-038226

References

1. Shadish WR, Cook TD, Campbell DT: Experimental and quasi-experimental designs for generalized causal inference. 2nd edition. Houghton Mifflin, Michigan,2002. Reference Source

2. SAMHSA. AUDIT-C. https://www.integration.samhsa.gov/images/res/tool_auditc.pdf

3. Babor TF, Higgins-Biddle JC, Saunders JB, Monteiro MG AUDIT: The Alcohol Use Disorders Identification Test Guidelines for Use in Primary Care (second edition). https://www.who.int/substance_abuse/publications/audit/en/

4. US Preventive Service task Force. Patient Health Questionnaire PHQ-9. https://www.uspreventiveservicestaskforce.org/Home/GetFileByID/218

5. Center for Quality Assessment and Improvement in Mental Health Patient Health Questionnaire PHQ-2. http://www.cqaimh.org/pdf/tool_phq2.pdf

6. Gual A, Anderson P, Segura L, et al.: Alcohol and Primary health care: Training programme on identification and brief interventions. Barcelona: department of Health of the Government of Catalonia,2005. Reference Source

7. Anderson P, Bendsten P, Spak F, et al.: Improving the delivery of brief interventions for heavy drinking in primary health care: outcome results of the Optimizing Delivery of Health Care Intervention (ODHIN) five-country cluster randomized factorial trial. *Addiction.* 2016;111(11):1935–1945. 27237081 10.1111/add.13476

8. Wensing M, Oxman A, Baker R, et al.: Tailored Implementation For Chronic Diseases (TICD): a project protocol. *Implement Sci.* 2011;6:103. 21899753 10.1186/1748-5908-6-103 3179734

9. Wensing M, Huntink E, van Lieshout J, et al.: Tailored Implementation of Evidence-Based Practice for Patients with Chronic Diseases. *PLoS One.* 2014;9(7):e101981. 25003371 10.1371/journal.pone.0101981 4087017

10. Flottorp SA, Oxman AD, Krause J, et al.: A checklist for identifying determinants of practice: a systematic review and synthesis of frameworks and taxonomies of factors that prevent or enable improvements in healthcare professional practice. *Implement Sci.* 2013;8:35. 23522377 10.1186/1748-5908-8-35 3617095

11. Prasad A, Kano M, Dagg KA, et al.: Prioritizing action on health inequities in cities: An evaluation of Urban Health Equity Assessment and Response Tool (Urban HEART) in 15 cities from Asia and Africa. *Soc Sci Med.* 2015;145(2015):237–242. 26456133 10.1016/j.socscimed.2015.09.031

12. Svoronos T, Mate KS: Evaluating large-scale health programmes at a district level in resource-limited countries. *Bull World Health Organ.* 2011;89(11):831–837. 22084529 10.2471/BLT.11.088138 3209726

13. Barker PM, Reid A, Schall MW: A framework for scaling up health interventions: lessons from large-scale improvement initiatives in Africa. *Implement Sci.* 2016;11(1):12. 26821910 10.1186/s13012-016-0374-x 4731989

14. Heather N: WHO Collaborative Project on Identification and Management of Alcohol-related Problems in PHC – Report to the World Health Organisation on Phase IV: Development of Country-wide Strategies for Implementing Early Identification and Brief Intervention in PHC. Geneva: World Health Organisation, Department of Mental Health and Substance Abuse:2006. Reference Source

15. Keller, P. A., & Lehmann, D. R. (2008). Designing effective health communications: a meta-analysis. *Journal of Public Policy & Marketing*, *27*(2), 117-130.

16. Rice, R. E., & Atkin, C. K. (Eds.). (2012). *Public communication campaigns*. Sage.

17. OECD: Compendium of OECD well-being indicators. OECD Better Life Initiative.2011; (Accessed 18 December 2016). Reference Source

18. Bertelsmann Stiftung. [website].2016; (Accessed 18 December 2016). Reference Source

19. Inglehart R, Welzel C: Modernization, Cultural Change and Democracy. New York: Cambridge University Press,2005. Reference Source

20. Anderson P, Clement S: The AAPPQ revisited: the measurement of general practitioners' attitudes to alcohol problems. *Br J Addict.* 1987;82(7):753–9. 3478065 10.1111/j.1360-0443.1987.tb01542.x

21 Riley MR, Mohr DC, Waddimba AC. The reliability and validity of three-item screening measures for burnout: Evidence from group-employed health care practitioners in upstate New York. Stress and Health. 2018 Feb;34(1):187-93.

22 Schaufeli WB, Shimazu A, Hakanen J, Salanova M, De Witte H. An Ultra-Short Measure for Work Engagement: The UWES-3 Validation Across Five Countries. European Journal of Psychological Assessment 2017

23 Rosenberg G, Bauld L, Hooper L et al. New national alcohol guidelines in the UK: public awareness, understanding and behavioural intentions. *Journal of Public Health*, 2018 40, 549–556,

24 Anderson P, Wojnar M, Jakubczyk A, Gual A, Reynolds J, Segura L, Sovinova H, Csemy L, Kaner E, Newbury-Birch D, Fornasin A. Managing alcohol problems in general practice in Europe: results from the European ODHIN survey of general practitioners. Alcohol and Alcoholism. 2014 Jul 16;49(5):531-9.

25 Francis J, Eccles MP, Johnston M, Walker AE, Grimshaw JM, Foy R, Kaner EF, Smith L, Bonetti D. Constructing questionnaires based on the theory of planned behaviour: A manual for health services researchers.

26 Bandura A. Guide for constructing self-efficacy scales. Self-efficacy beliefs of adolescents. 2006 Feb 1;5(1):307-37.

27 Bergström A, Skeen S, Duc DM, Blandon EZ, Estabrooks C, Gustavsson P, Hoa DT, Källestål C, Målqvist M, Nga NT, Persson LÅ. Health system context and implementation of evidence-based practices—development and validation of the Context Assessment for Community Health (COACH) tool for low-and middle-income settings. Implementation Science. 2015 Dec;10(1):120.

28 Scott SD, Plotnikoff RC, Karunamuni N, Bize R, Rodgers W. Factors influencing the adoption of an innovation: An examination of the uptake of the Canadian Heart Health Kit (HHK). Implementation Science. 2008 Dec;3(1):41.

29. Moore GF, Audrey S, Barker M, et al.: Process evaluation of complex interventions: Medical Research Council guidance. *BMJ.* 2015;350:h1258. 25791983 10.1136/bmj.h1258 4366184

30. Reeves BC, Wells GA, Waddington H: Quasi-experimental study designs series-paper 5: classifying studies evaluating effects of health interventions-a taxonomy without labels. *J Clin Epidemiol.* 2017; pii: S0895-4356(17)30288-3. 28351692 10.1016/j.jclinepi.2017.02.016
