# Supplementary material for: Implementing primary healthcare-based measurement, advice and treatment for heavy drinking and comorbid depression at the municipal level in three Latin American countries: final protocol for a quasiexperimental study (SCALA study)
Source: BMJ Open. 2020 Jul 28;10(7):e038226. doi: 10.1136/bmjopen-2020-038226 (PMC7390229; doi:10.1136/bmjopen-2020-038226)
Supplement: Supplementary data [file bmjopen-2020-038226supp002.pdf]

SCALA - DATA MANAGEMENT PLAN

Draft version 1: 23 January, 2018
Draft version 2: 1 February, 2018
Draft version 3: 27 February, 2018
Draft version 4: 2 March, 2018
Draft version 5: 15 March, 2018
Draft version 6: 15 May, 2018
Draft version 7: 23 May, 2018
version 8: 24 May, 2018

Abbreviations and definitions:

DMP	= data management plan
IRB	= Institutional Research Board
PHCCs	= primary health care centres
SCALA	= Scale-up of Prevention and Management of Alcohol Use Disorder in Latin America
Data center	= Technische Universität Dresden, Germany (supervisor: Jürgen Rehm)

Contents

1. Data Summary ... 1
2. FAIR data ... 5
2.1. Making data findable, including provisions for metadata ... 5
2.2. Making data openly accessible ... 5
2.3. Making data interoperable ... 6
2.4. Increase data re-use (through clarifying licences) ... 7
3. Allocation of resources ... 8
4. Data security ... 9
5. Ethical aspects ... 10
6. Other issues ... 11
7. Data analysis plan ... 12
7.1. REACH ... 13
7.2. EFFECTIVENESS ... 14
7.3. ADOPTION ... 14
7.4. IMPLEMENTATION ... 15
7.5. MAINTENANCE ... 15
8. Appendix ... 18

1. Data Summary

Introduction

During the course of the SCALA study, quantitative, qualitative, as well as publicly available data will be collected in PHCCs in three American countries: Mexico, Peru, Colombia. All collected data are required for a thorough evaluation of the main study goal and it corollaries, ie. to improve alcohol management in PHCCs by increasing screening rates and delivery of adequate advice and treatment for screen positives. The following qualitative and quantitative data will be obtained from patients and providers in PHCCs. All data will be transferred first to the data center serving as SCALA data repository at the TU Dresden (for details on data transfer, see ***section 4***). After cleaning the data and bringing it into the standard format (for details, see ***section 2.2***), the data will be forwarded to partners based on the workplan or upon request. While all data will be kept with the data center, they are collectively owned by all partners.

Data origin

Q1) **PHCC structure data (quantitative):**
Collection of data from the participating PHCCs before start of data collection. The PHCC administration will be asked to fill out a form (see '***Q1_PHCC Description Form.pdf***'), including the number of registered patients, as well as number of health professionals working in the centre. The data will be entered into spreadsheets (see '***Q1_PHCC Description Form_spreadsheet template.xlsx***'), which will then be sent to the data center.

Q2) **Short tally sheet for routine care data (quantitative):**
Collection of routine care data on **all** adult patients consulting PHCCs. For this purpose, a tally sheet (see '***Q2_Short Patient Tally Sheet.pdf***') will be applied to collect all necessary information on sociodemographics (sex, age, socioeconomic status) and drinking patterns (AUDIT-C) for all patients. For screen positives, the tally sheet will also capture the results of in-depth assessment of alcohol problems (AUDIT) and depression (PHQ-2 and - if above threshold - PHQ-9) and the decisions made concerning brief advice and treatment and referral to specialist care. The tally sheets will be collected by local researchers on a weekly basis and entered into spreadsheet templates (see '***Q2_Short Patient Tally Sheet_spreadsheet template.xlsx***'). These spreadsheets will be submitted monthly to the data center.

Q3) **Long tally sheet for quality control data (quantitative):**
Collection by respective PHCC of a more extensive set of routine care data for quality control on **a subset** of adult patients consulting PHCCs. Quality control data will only be collected during predefined periods during the 18 months implementation period, resulting in about 1 in 10 patients being assessed. In order to allow for comparisons between long tally sheet and interview data, the periods for application of long tally sheets will be aligned with realisation of patient interviews. The long tally sheet will cover all variables from the short tally sheet (see Q2 and '***Q3_Long Patient Tally Sheet.pdf***'), in addition to assessment of educational level (1 question), attempts on cutting down drinking (2 questions), alcohol health literacy (4 questions), and injunctive social norms (2 questions). As with short tally sheets, long tally sheets will also be collected weekly by local researchers and entered into spreadsheet templates (see '***Q3_Long Patient Tally Sheet_spreadsheet template.xlsx***'). These spreadsheets will be submitted to the data center whenever data were collected.

Jané-Llopis E, *et al. BMJ Open* 2020; 10:e038226. doi: 10.1136/bmjopen-2020-038226

Q4) Tally Sheets Cover Form (quantitative):
Short and long tally sheets will be distributed to the PHCCs by local researchers on a weekly basis and each set of tally sheets will have a cover form (see '***Q4_Tally Sheets Cover Form.pdf***'). On this cover form, the PHCC administration will be asked to fill in the number of adult consultations during the respective week for each participating provider. The cover forms will be collected together with the short/long tally sheets and will be entered in the same spreadsheets and then submitted to the data center.

Q5) Tally Sheet Appendix (consent taking for patient interview):
In predefined weeks during month 3 of the 18-month implementation period, PHCC providers will ask all patients to participate in researcher-conducted personal interviews. Patient consent and contact details will be collected on a form appended to either short or long tally sheets during these weeks (see '***Q5_Patient Tally Sheet Appendix.pdf***'). To allow for a stratified sampling of interviewees according to screening results (ratio of positively and negatively screened patients = 2:1) by local researchers, the providers will also note down the AUDIT-C screening result on the form. These forms will be collected alongside the short/long tally sheets and the data will only be used to sample and recruit interviewees.

Q6) Patient interview data:
Collection of individual data through patient interviews at month 3 and subsequent follow-ups at months 6 and 12. Random samples of positively and negatively screened patients (ratio 2:1) will be interviewed across all municipalities, resulting in a total number of N=1,080 patients. The interview will contain all questions from the long tally sheet (see '***Q3_Long Patient Tally Sheet.pdf***'), in addition to 2 questions for quality control assessing experience of screening/brief advice with PHCC providers, a six-item modified version of the HLS-EU-16 to assess alcohol health literacy, the World Health Organization Disability Assessment Schedule to assess the degree of disability, and questions on health resource utilization (see '***Q6_Patient Interview.pdf***'). The patient interview will be conducted as face-to-face or telephone interview and collected data will be entered into prepared spreadsheets (see '***Q6_Patient interview_spredsheet sample.xlsx***') and sent to the data center.

Q7) Provider questionnaire data (quantitative):
Collection of data from health care providers, which will be assessed prior to or during the 4-week baseline period and repeated at months 4.5 and 13.5. All providers will be asked to fill out questions on alcohol knowledge, alcohol health literacy, as well as on attitudes towards alcohol users and alcohol problems (SAAPP Questionnaire, see '***Q7_Provider questionnaire.pdf***'). The data will be entered into prepared spreadsheets (see '***Q7_Provider questionnaire_spredsheet sample.xlsx***') and sent to the data center.

Q8) Provider interview data (qualitative):
At the end of the 18-month implementation period, a random sample of 1 in 20 PHCC providers of both control and intervention groups will be invited to participate in a 15 minute semi-standardized interview (see '***Q8_Provider Interview from Annexe 25.pdf***'), which will be taped and conducted via telephone. The interviews aim to assess provider experiences on implementing the intervention package in their routines. Recordings of the provider interviews will be transcribed.

Q9) Process data interviews (qualitative):

As part of the process evaluation, semi-structured focus-group interviews will be conducted with the User Panels, Community Advisory Boards, and local research groups. The focus groups will cover the topics of tailoring of materials, and decision making processes for adoption mechanisms, support systems, and completing driver diagrams and barriers and facilitator tables.

Q10) **Recruitment documentation (quantitative):**
Local researchers will be given forms to document the entire PHCC recruitment process (see '***Q10_Recruitment documentation.pdf***'). For each municipality, they will document the total number of PHCCs and the number of contacted PHCCs for study participation. Among contacted PHCCs, the number of non-responding, refusing, and accepting PHCCs will be assessed. For each PHCC contacted for study participation, the following data will be assessed: number of registered patients and number of workers, type and number of contacts with PHCC, PHCC response (acceptance, refusal, non-response), and reasons for refusal or non-response if applicable. The data will be entered into prepared spreadsheets (see '***Q10_Recruitment documentation_spreadsheet template.xlsx***') and sent to the data center.

Q11) **Follow-up documentation (quantitative):**
Local researchers will monitor key activities of each PHCC provider during the course of the study using a standardized sheet (see '***Q11_Follow-up documentation.pdf***'). Key activities to be documented relate to participation in training sessions and potential reasons for non-participation. If providers drop out of the study prior to end of the 18 months implementation period, this will also be documented, in addition to any reasons for drop out. On the same follow-up documentation form, sex and age of the provider will be assessed as well. The data will be entered into prepared spreadsheets (see '***Q11_Follow-up documentation_spreadsheet template.xlsx***') and sent to the data center.

All quantitative data will be collected directly by PHC providers and the country research teams, through patient interviews or provider surveys.

Data types, format, and size

The total size of all quantitative data collected in the course of this study is unlikely to exceed 100MB and will be stored as easily accessible spreadsheets (.csv - format). Transcripts from qualitative interviews will be stored as Microsoft Word documents (.docx - format), not exceeding 100MB in total.

Purpose of data collection with regard to study objectives

The quantitative data will be required to evaluate if study objectives can be reached (for an overview of the study objectives, see '***Figure_RE-AIM.png***'). In particular, Q2 (short tally sheet), Q3 (long tally sheet) and Q4 (patient interview) data will provide outcome measures, which allows for evaluation of the ***REACH*** (maximising exposure to screening and brief advice/treatment in PHC) and ***EFFECTIVENESS*** (increasing adequate alcohol management in PHC) study objectives.

All qualitative data will be obtained through interviews with User Panels, Community Advisory Boards, local research groups, patients and providers, which will be used to evaluate the ***IMPLEMENTATION*** (factors affecting the implementation of intervention package) and ***ADOPTION*** (increase adoption of the intervention package in PHC) study objectives.

Furthermore, publicly available and process data will be obtained during the course of the study. In detail, this will comprise information necessary to characterize countries, cities and municipalities, contextual, political, socio-economic, and alcohol policy factors (e.g. legislation), and a thorough description of Community Advisory Boards. These data will contribute to the process evaluation (Work Package 5) and serve as base to evaluate the ***MAINTENANCE*** (long term effects of implementation) study objective.

A detailed description of the analytic steps planned to achieve study objectives can be found in ***section 7***.

Re-using data

Most of the data collected during the course of this study will be primary data collected through health care professionals and from patients directly. However, publicly available data form an important pillar in this study as it will be required for process evaluation and economic analyses.

Data utility

The collected data will not only be used to achieve the above listed study goals; they can be used by other researchers to plan similar studies, to examine other hypotheses, or for population modelling purposes.

2. FAIR data

2.1. Making data findable, including provisions for metadata

Making data discoverable, identifiable, and locatable

All quantitative data sets will be made publicly available through the UK Data Service after publication of the results, or, at the latest, 12 months after the finalization of the study.[1] Each data set published with the UK Data Service will be attached with a unique 'Digital Objective Identifier' (DOI).

Data derived from qualitative interviews will not be stored in the UK data archive as anonymity of qualitative interviews cannot be ensured.

Naming conventions and version numbers

For all data sets a predefined title standard ("SCALA_data_*NAME*_v1_*DATE.csv*") and the same author group ("SCALA study group") will always be used. Within titles, consecutive version numbers will be used to facilitate updates and corrections to uploaded data sets and to ensure unambiguous identification of data sets.

Key word conventions

All stored data will be labelled with the following keywords: SCALA, Americas, Mexico, Peru, Colombia, Primary Health Care, Alcohol, Heavy Drinking, Depression, Prevention, Screening, Brief Advice, Treatment. Additional keywords will be considered to characterize the respective data set. As data on resource use will be used for economic analyses, data sets containing relevant data will further be classified using 'JEL Classification Codes'.[2]

Meta data handling

There are no standards on handling metadata in this discipline and there is no intention to manage metadata of the publicly stored data sets apart from the measures listed above.

2.2. Making data openly accessible

Making data openly available

By default, all quantitative datasets generated in the course of the SCALA study will be made openly available through the UK Data Service upon publication of the results. Prior to publication, all data will be formatted to meet UK Data Service requirements.

Access conditions and required software

All quantitative data will be provided as 'comma separated values' (CSV) – an efficient and open source format to store larger data sets. This is a generic, widely used file format, which can be handled by all major software packages used for quantitative analyses (eg. Microsoft Excel, SAS, SPSS, Stata, R). In

[1] http://www.data-archive.ac.uk/
[2] https://en.wikipedia.org/wiki/JEL_classification_codes

Jané-Llopis E, *et al. BMJ Open* 2020; 10:e038226. doi: 10.1136/bmjopen-2020-038226

order to maintain accessibility, large data sets will be split into smaller parts, which will not exceed 50 MB file size.

Depositing metadata, documentation, and code

Each dataset stored with the UK Data Service will be accompanied by a set of documenting files, which comprises relevant publications, consent forms, questionnaires/interview guidelines, and codebooks. The codebooks stored alongside the dataset will be Excel files (".xlsx") that contain extensive metadata for each variable in the associated data set, such as original questions, value labels, defined missing values, and possible coding rules applied.

Arrangements with the UK Data Service

The UK Data Service has been contacted and the study team received a positive response with regard to storing study data with the service. When preparing files to be published online, guidelines and checklists of the UK Data Service will be considered (see [3,4]). Licence agreements will be finalized after obtaining approval of all IRBs.

Data not being made available

All qualitative data will be generated from semi-standardized interviews. Excerpts of these interviews will be appended to respective publications if applicable. However, full interview transcripts will not be published for the following reasons: first, sharing full interview transcripts is uncommon in this field; and, second, sharing poses a potential risk for disclosing the identity of the interviewee.

Restrictions of use and data access committee

As all relevant data will be made publicly available, there will be no need for a data access committee. If other researchers wish to examine interview transcripts, fully anonymized excerpts can be made available through the responsible researchers.

Ascertainment of identity of person accessing the data

It is aimed that all relevant data are to be shared as 'Open Data'.[5] This will imply that all data will be fully anonymized and there will be no means necessary to ascertain the identity of persons accessing the data.

2.3. Making data interoperable

Interoperability of data

All gathered data will be completely interoperable as they will be stored in widely used data formats, which make them accessible by a broad spectrum of data processing software packages, including open source applications.

[3] https://www.ukdataservice.ac.uk/deposit-data/preparing-data
[4] https://www.ukdataservice.ac.uk/media/440320/depositsurvey.pdf
[5] https://www.ukdataservice.ac.uk/get-data/data-access-policy/open-data

Data and metadata vocabularies, standards, or methodologies

As there is no standard vocabulary set for variable names in our discipline, a simple and easy-to-comprehend nomenclature will be developed and applied to all quantitative data sets and summarized in accompanying codebooks. For prospective assessments on the same individuals, data sets will be structured in a 'long data format', i.e. one variable will indicate the time of assessment of the same variables (see [6] for a more comprehensive explanation).

2.4. Increase data re-use (through clarifying licences)

Data licence

All study data stored with the UK Data Service will be published as "open data" if possible. For this storage mode, the information in the data set will not allow disclosure of any respondents. "Open data" is published using the Open Government Licence[7] and users will have direct access of data without prior registration with UK data service, facilitating wide reach and potential re-use of data collected in this study.

Time of data availability

All quantitative data sets will be made publicly available after publication of the results, or, at the latest, 12 months after the finalization of the study.

Duration of data storage

All data stored with the UK Data Service are held in perpetuity (see [8]).

Re-use by third parties

Data re-use by third parties is explicitly encouraged and will be facilitated by publication of codebooks and documentation along the data sets.

Data quality assurance processes

Prior to sharing the data with the UK Data Service, the study team will clean the data to ensure internal consistency. Several checks of the study team will be conducted before the data will be shared publicly.

[6] http://www.theanalysisfactor.com/wide-and-long-data/
[7] http://www.nationalarchives.gov.uk/doc/open-government-licence/version/2/
[8] https://www.ukdataservice.ac.uk/media/173249/UKDS_Collections_Development_Policy_02_00.pdf

3. Allocation of resources

Costs for open access publications

In total, the study budget includes €36,000 to pay 'open access' publication licence fees.

Costs for sharing data through repository

Storage of study data with the UK Data Service does not require any fees.

Long term costs for preservation

No long term costs are anticipated.

Data protection, data transfer and data sharing

The Data Protection Officers of both Technical University Dresden and of Maastricht University are the focal points for reviewing data protection, data transfer and data sharing, and required ethics reporting.

4. Data security

Data security - transfer

All collected data will be transferred to the data center in encrypted packages created with the open access 7-zip software. The 'Advanced Encryption Standard' (AES) with 256 bits will be applied, which has been widely recognized as standard encryption technique [9]. The same data transfer methods will be used to transfer the data to the other partners who request or need the data.

Copies of transcribed data notes that are required for the process evaluation in Work Package 6 will be sent by registered courier to ESADE.

Data security - storage

All electronic data will be stored on encrypted hard drives by respective partners. This will include mail communication, study documentation and codes applied to manipulate data and to generate results. Backup hard drives will be used to facilitate recovery of lost data.

All analogue data sources (tally sheets, interview notes, etc.) will be kept by the local research teams, where the data will be kept and stored adhering to local regulations.

All data stored with the UK Data Service are securely kept for perpetuity.

[9] https://en.wikipedia.org/wiki/Advanced_Encryption_Standard

Jané-Llopis E, *et al. BMJ Open* 2020; 10:e038226. doi: 10.1136/bmjopen-2020-038226

5. Ethical aspects

Ethical or legal issues regarding data sharing

After collection of the raw data, local researchers will assign predefined identification codes to each individual and remove all potentially identifying information from the data. The key to match individuals to the assigned identification code will remain with the local researchers. After the data has been securely transferred to the data center for cleaning and subsequent analyses, there will be no possibility no identify individuals from the data.

All data collection, processing, and sharing procedures will adhere to national and international laws including the General Data Protection Regulation (EU Regulation 2016/679).

The SCALA study team currently seeks approval for the study design, data collection and analysis from the research ethics board at the TU Dresden, Germany (registration number: 'EK 90032018'). In addition, ethical review is currently under way in Colombia, Mexico, and Peru.

Informed consent for data sharing and long term preservation

Informed consent will be obtained from providers and patients providing individual level data (through interviews or questionnaires) to allow data sharing through the UK Data Service.

6. Other issues

Use of other procedures for data management

Data management in the SCALA study will adhere to EU Regulation 2016/679. There are no further national or institutional requirements which would counteract or extend this regulation or any of the procedures specified in this document.

7. Data analysis plan

In Section 1, data sources are mapped to study goals. For each study goal, the required definition of variables and planned statistical analyses are described in the following.

General considerations

Given that SCALA is a quasi-experimental study design (technically, a non-randomized controlled trial (NRCT)), data for a range of potential confounders will be collected at baseline (with repeat measurements during the course of the 18-month implementation period) both to undertake propensity score matching between intervention and comparator municipalities, and include as confounders in the statistical analyses:

At the level of the PHCC, PHC-provider and patient:

- *Age, sex and profession (doctor, nurse, other health care worker) of provider:* Evidence suggests that the sex and age of the provider are unimportant in influencing screening and advice rates, whereas profession is. Nurses tend to screen more patients than doctors; doctors tend to advise more screen positive patients than nurses.
- *Number of monthly consultations:* Evidence suggests that the higher the number of consultations, the lower the proportion of patients screened.
- *Attitudes and knowledge of providers:* Evidence suggests that providers with more positive attitudes, in terms or role security and therapeutic commitment, and providers with high levels of alcohol-related knowledge, are more likely to screen and advise a greater proportion of patients.
- *AUDIT-C score:* The evidence suggests that the higher the AUDIT-C score, the greater the likelihood that screen positive patients will be given advice.

At the level of the municipality:

- A priori, comparator municipalities have been chosen to be similar to intervention municipalities in terms of socioeconomic and other characteristics which impact on drinking, health care and survival, comparable community mental health services. During the set-up phase, additional data will be collected form the municipalities on existing actions and training of PHC-based screening and brief advice for heavy drinking; availability and accessibility of specialist services for severe AUD and moderately severe or severe depression; and, existing municipal-based prevention and/or policy programmes to reduce heavy drinking

7.1. REACH

Primary outcome measures:

A1 Number of intervened patients per provider and per PHCC

Secondary outcome measures:

A2 Number of screened patients per provider and per PHCC
A3 Number of advised patients per provider and per PHCC
A4 Number of patients referred for severe AUD per provider and per PHCC

A5 Number of patients referred for moderately severe or severe depression per provider and per PHCC
A6 Provider attitudes
A7 Provider alcohol health literacy
A8 Representativeness of population intervened for AUD

Definition:

Measure A1 represents the *primary* outcome variables in this study and is assessed in three 4-week periods: in the first month 1 (t1), after 9 months (t2) and after 18 months (t3). It will be the proportion of consulting adult patients (aged 18+ years) intervened (screened and advice given to screen positives), calculated as the number of AUDIT-C positive patients that received oral advice or referral for advice to another provider in or outside the PHCC, divided by the total number of adult consultations of the participating providers per provider and per PHCC.

Measures A2 to A5 represent *secondary* outcome variables in this study and are assessed in the same three 4-week periods as measure A1: in the first month 1 (t1), after 9 months (t2) and after 18 months (t3). Measure A2 will be the proportion of patients screened, calculated as the number of completed screens divided by the total number of consultations of all adult patients per participating provider, and averaged per participating PHCC. Measure A3 will be the proportion of patients advised, calculated as the number of brief interventions delivered (received oral brief advice, and/or were referred to another provider in or outside the practice), divided by the total number of screen positives per participating provider and averaged per participating PHCC. Information will also be collected on the number of screen negatives who received brief advice. Measure A4 will be the proportion of patients with severe AUD referred to specialist treatment, calculated as the proportion of patients with an AUDIT-C score ≥8 and a full AUDIT score ≥20 documented as referred to treatment per participating provider, and per participating PHCC. Measure A5 will be the proportion of patients with moderately severe or severe depression referred to specialist treatment, calculated as the proportion of patients with an AUDIT-C score ≥8 and a PHQ-9 score ≥15 documented as referred to treatment per participating provider, and per participating PHCC.

Measures A6 and A7 are also *secondary* outcome variables in this study and will be assessed in three 4-week periods through provider questionnaires: at baseline (t1), after 4.5 months (t2) and after 13.5 months (t3). Measure A6 will be measured by the SAAPP questionnaire, with responses to be summed within the two scales of role security and therapeutic commitment. Individual missing values for any of the items in a domain will be assigned the mean value of the remaining items of the domain before summation. Measure A7 will be assessed through knowledge of risks due to drinking, and reported descriptive and injunctive social norms of drinking. Measure A8 will be determined through process evaluation activities conducted throughout the implementation period. Among other things, representativeness will be evaluated through comparing patients with people living in the catchment area of the respective PHC on a number of variables.

Analyses/Achievement:

For all measures, means and/or proportions (as applicable) will be presented descriptively by country, control and intervention municipality, and for the total sample. Given the relative rarity of some events (eg. measure A1 to A5) and the resulting distribution, we will use exact inference methods for comparison of intervention vs. comparator municipalities.

For further analyses, including covariates, regression models will be used, taking into consideration the hierarchical nature of the data, and characteristics at different hierarchy levels (i.e., characteristics of the PHCC, characteristics at the municipal level, such as patterns of drinking). Multilevel models are well suited for this purpose and will be built to evaluate the intervention effect for measures A1 to A7. For the primary outcome, the model will be built as follows:

- *Dependent variable:* proportion of patients intervened among all consultations per provider and per PHCC
- *Independent variable 1:* Time (t1-t3)
- *Independent variable 2*: Control vs. intervention municipality
- *Hierarchical cluster*: Provider nested within PHCC nested within country (to control for design effects)
- *Statistic*: Interaction effect between time and group allocation

After testing for the necessary assumptions, the above outlined generalized linear model will be applied to the actual distribution of the outcome measure. Thus, skewness of data resulting from rare events would be analysed using zero-inflated negative binomial regression. For all remaining outcome measures, similar models will be applied.

7.2. EFFECTIVENESS

Outcome measures:

B1 Increased health literacy in PHCC patients using a modified version of the UK-based Newest Vital Sign and a six-item adapted version of Health Literacy Survey-EU Questionnaire (HLS-EU-16)

B2 Reduction in alcohol consumption of AUD+ drinkers

Definition:

Data for measures B1 and B2 are collected through patient interviews (conducted in month 3, 6 and 12).

Analyses/Achievement:

Similar multilevel regression models as applied for primary and secondary outcomes mapped to study goal *REACH* will be applied to measures B1 and B2. The main difference will be that these measures will be analyzed on the individual level, which requires adding another level (patient nested with provider nested within PHCC nested within country) to the model.

7.3. ADOPTION

Outcome measures:

C1 Adoption rate and representativeness of PHCCs

C2 Adoption rate and representativeness of PHCC staff

Definition:

Adoption rate of PHCCs will be calculated as the number of PHCCs agreeing to be part of the study divided by the number of PHCCs contacted.

Adoption rate of PHCC providers within each PHCC that joins the study will be calculated as the number of PHCC providers agreeing to be part of the study divided by the total number of PHCC providers within each PHCC, stratified by profession (doctor, nurse, other).

Analyses/Achievement:

To determine the representativeness of PHCCs involved in the study, routine available data on the size, number of registered patients, and number and characteristics of staff will be used and compared between PHCCs who agreed to be part of the study and contacted PHCCs who declined to be part of the study.

To determine the representativeness of PHCC staff within the involved PHCC, routine available data on the number and characteristics of staff will be used to compare, within each PHCC, those staff who joined the study and those staff who declined to join the study.

7.4. IMPLEMENTATION

Outcome measures:

D1 Extent primary health care screening and advice package delivered as intended

D2 Multi-level evaluation of barriers/facilitators to scale-up using WHO's Urban Health Equity Assessment and Response Tool

D3 Extent implementation on city levels delivered as intended using Medical Research Council guidance

D4 Cost of package implementation

Definition:

All measures D1 to D3 will be assessed through process evaluation activities. The required data will be obtained through interviews with PHCC providers (D1) and with members from Community Advisory Boards (D2, D3). For D4, a comprehensive set of data will be required, comprising patient data on disability and health resource utilization obtained from patient interviews as well as data on unit costs obtained from public data sources.

Analyses/Achievement:

Measures D1 to D3 will be analyzed through qualitative evaluation. Measure D4 will be evaluated by a comprehensive economic evaluation, for which different sources of costs will be considered, such as costs attributable to implementation of the intervention routine as well as costs attributable to utilization of health care services. In a cost-effectiveness study, the hypothesized gain in quality of life among patients in intervention municipalities will be contrasted with recorded and calculated costs.

7.5. MAINTENANCE

Process measures:

E1 Assessment of outcomes 18 months post implementation

E2 Indicators of program-level maintenance

E3 Measures of cost of maintenance
E4 Dissemination / events

Definition:

For measure E1 data from PHC providers and patients up to 18 months after implementing the alcohol management routine need to be collected.

For measure E2, the required indicators will be collected through process evaluation activities, namely interviews with members of the Community Advisory Boards.

For measure E3, all costs will be collected throughout the implementation period within the economic evaluation framework (see measure D4), in order to estimate the costs of maintenance.

For measure E4, the study results will be disseminated through municipal, national, and international structures, following the 'Communication, Dissemination and Exploitation Plan'.

Analyses/Achievement:

Measure E1 will be achieved by continuous data collection across the entire implementation period of 18 months.

Measure E2 will be achieved by analysis of qualitative data. Measure E3 will be achieved through an economic evaluation of the implementation package considering the entire implementation period.

Measure E4 will be achieved by following the 'Communication, Dissemination and Exploitation Plan'.

8. Appendix

List of all documents referenced in the DMP:

Document	Page Number
1. Q1_PHCC Description Form template.pdf	18
2. Q1_PHCC Description Form_spreadsheet template.xlsx	Excel not attached
3. Q2_Short Patient Tally Sheet.pdf	19
4. Q2_Short Patient Tally Sheet_spreadsheet template.xlsx	Excel not attached
5. Q3_Long Patient Tally Sheet.pdf	22
6. Q3_Long Patient Tally Sheet_spreadsheet template.xlsx	Excel not attached
7. Q4_Tally Sheet Cover Form.pdf	26
8. Q5_Tally Sheet Appendix.pdf	27
9. Q6_Patient Interview.pdf	29
10. Q6_Patient interview_spreadsheet template.xlsx	Excel not attached
11. Q7_Provider questionnaire.pdf	34
12. Q7_Provider questionnaire_spreadsheet template.xlsx	Excel not attached
13. Q8_Provider Interview from Annexe 25.pdf	36
14. Q10_Recruitment documentation.pdf	53
15. Q10_Recruitment documentation_spreadsheet template.xlsx	Excel not attached
16. Q11_Follow-up documentation.pdf	55
17. Q11_Follow-up documentation_spreadsheet template.xlsx	Excel not attached
18. Figure_RE-AIM.png	58

Jané-Llopis E, *et al. BMJ Open* 2020; 10:e038226. doi: 10.1136/bmjopen-2020-038226

PHCU Description Form

Country and municipality details
(to be filled in by local research team)

**Country**	☐ Colombia	☐ Mexico	☐ Peru
**Municipality**	________________	**Control or Experimental**	☐ Control<br>☐ Experimental
**ID of PHCU**	________________		

PHCU details
(to be filled in by PHC administration)

**Name/Address of PHCU**		________________	
**Total number of registered patients**		________________	
**Total number of registered *adult* (18+) patients**		________________	
**Number of workers working in PHCU**	General Practitioners	**Part time**	________________
		**Full time**	________________
	Nurses	**Part time**	________________
		**Full time**	________________
	Assistants	**Part time**	________________
		**Full time**	________________
	Psychologists	**Part time**	________________
		**Full time**	________________
	Social workers	**Part time**	________________
		**Full time**	________________
	Others: __________	**Part time**	________________
		**Full time**	________________

Short Tally Sheet

Provider details and consultation

**Practice ID** (*pre-printed*)	________________	**Provider ID / Name** (*pre-printed*)	________________
**Date consultation**	____ / ____ / ____		

Patient details

**Sex**	☐ Male<br>☐ Female<br>☐ Other	**Age**	______ years
**Socioeconomic status**	☐ Below average	☐ Average	☐ Above average

AUDIT-C Alcohol Screening

Questions	0	1	2	3	4	Score
**1** **How often do you have a drink containing alcohol?**	Never	Monthly or less	2-4 times per month	2-3 times per week	4+ times per week	
**2** **How many units of alcohol do you drink on a typical day when you are drinking?**	1-2	3-4	5-6	7-9	10+	
**3** **How often do you have 6 or more units on one occasion?**	Never	Less than monthly	Monthly	Weekly	Daily or almost daily	

Standard Drinks Placeholder

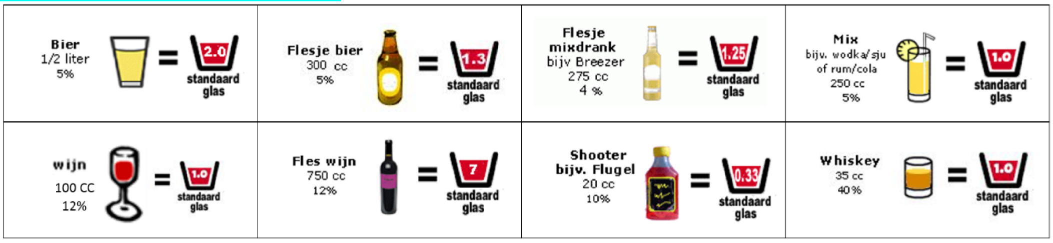

**Sum score AUDIT-C (possible range 0-12)**	__ __
**If AUDIT-C score ≥ 8 => Apply remaining AUDIT and PHQ-2 questionnaire**	

AUDIT (remaining scale)

Questions	0	1	2	3	4	Score
**4** **How often during the last year have you found that you were not able to stop drinking once you had started?**	Never	Less than monthly	Monthly	Weekly	Daily or almost daily	
**5** **How often during the last year have you failed to do what was normally**	Never	Less than monthly	Monthly	Weekly	Daily or almost daily	

Short Tally Sheet

	expected from you because of drinking?						
6	How often during the last year have you needed a first drink in the morning to get yourself going after a heavy drinking session?	Never	Less than monthly	Monthly	Weekly	Daily or almost daily	
7	How often during the last year have you had a feeling of guilt or remorse after drinking?	Never	Less than monthly	Monthly	Weekly	Daily or almost daily	
8	How often during the last year have you been unable to remember what happened the night before because you had been drinking?	Never	Less than monthly	Monthly	Weekly	Daily or almost daily	
9	Have you or someone else been injured as a result of your drinking?	No		Yes, but not in the last year		Yes, during the last year	
10	Has a relative or friend or a doctor or another health worker been concerned about your drinking or suggested you cut down?	No		Yes, but not in the last year		Yes, during the last year	
	Sum score (possible range 0-28)						__ __
	Sum score full AUDIT (possible range 0-40)						__ __
	If full AUDIT score ≥ 8 => Apply remaining AUDIT and PHQ-2 questionnaire						

PHQ-2 Depression Screening

Over the last 2 weeks, how often have you been bothered by any of the following problems?	Not at all	Several days	More than half the days	Nearly every day
1 Little interest or pleasure in doing things	0	1	2	3
2 Feeling down, depressed, or hopeless	0	1	2	3
Sum score (possible range 0-6) __ __				
If PHQ-2 score ≥ 3 => Apply remaining PHQ questionnaire				

PHQ-9 (remaining scale)

Over the last 2 weeks, how often have you been bothered by any of the following problems?	Not at all	Several days	More than half the days	Nearly every day
3 Trouble falling or staying asleep, or sleeping too much	0	1	2	3
4 Feeling tired or having little energy	0	1	2	3
5 Poor appetite or overeating	0	1	2	3

Short Tally Sheet

**6 Feeling bad about yourself or that you are a failure or have let yourself or your family down**	0	1	2	3
**7 Trouble concentrating on things, such as reading the newspaper or watching television**	0	1	2	3
**8 Moving or speaking so slowly that other people could have noticed. Or the opposite being so figety or restless that you have been moving around a lot more than usual**	0	1	2	3
**9 Thoughts that you would be better off dead, or of hurting yourself**	0	1	2	3
**Sum score (possible range 0-21)**	__ __			
**Sum score full PHQ-9 (possible range 0-27)**	__ __			

Taking record of brief advice or referral

**If full AUDIT < 26 and PHQ-9 < 15:**	
**Brief advice** *(more than one answer is possible)*	☐ Oral Brief Advice given<br>☐ Patient Leaflet given<br>☐ Continued Monitoring<br>☐ Patient referred to other provider in practice for brief advice<br>☐ Patient referred to other provider outside practice for brief advice<br>☐ Other ________________________<br>☐ Time did not allow, but<br>☐ I made follow-up appointment<br>☐ Patient declined brief advice<br>☐ Patient not screen positive, but reinforced about keeping low risk drinking habits
**If full AUDIT ≥ 26 and/or PHQ-9 ≥ 15:**	
**Patient referred to special services:**	☐ Yes<br>☐ No

Jané-Llopis E, *et al. BMJ Open* 2020; 10:e038226. doi: 10.1136/bmjopen-2020-038226

Long Tally Sheet

Provider details and consultation

**Practice ID** (*pre-printed*)	________________	**Provider ID / Name** (*pre-printed*)	________________
**Date consultation**	____ / ____ / ____		

Patient details

**Sex**	☐ Male<br>☐ Female<br>☐ Other	**Age**	______ years
**Socioeconomic status**	☐ Below average	☐ Average	☐ Above average
**Highest level of education**	☐ No schooling completed<br>☐ Junior high school completed<br>☐ Business/Technical training<br>☐ Doctorate degree		☐ Primary school completed<br>☐ High school completed<br>☐ Bachelor's/Master's degree

Alcohol exposure, health literacy, and social norms

During the last 12 months have you tried to cut down on your drinking by:		
Choosing lower strength alcohol	☐ Yes	☐ No
Using smaller glasses	☐ Yes	☐ No
How easy is it to understand health information about drinking of alcohol?	☐ Always easy<br>☐ Usually easy	☐ Sometimes difficult<br>☐ Often difficult<br>☐ Always difficult
To the best of your knowledge, can drinking alcohol cause any of the following:		
High blood pressure	☐ Yes	☐ No
Liver problems	☐ Yes	☐ No
Cancer	☐ Yes	☐ No
Thinking about your friends, would you say that it is acceptable or unacceptable for them to drink:		
Regularly more than two drinks a day?	☐ Acceptable	☐ Unacceptable
More than six drinks on an occasion?	☐ Acceptable	☐ Unacceptable

AUDIT-C Alcohol Screening

Questions		0	1	2	3	4	Score
1	**How often do you have a drink containing alcohol?**	Never	Monthly or less	2-4 times per month	2-3 times per week	4+ times per week	
2	**How many units of alcohol do you drink on a typical day when you are drinking?**	1-2	3-4	5-6	7-9	10+	

Long Tally Sheet

3	**How often do you have 6 or more units on one occasion?**	Never	Less than monthly	Monthly	Weekly	Daily or almost daily	

Standard Drinks Placeholder

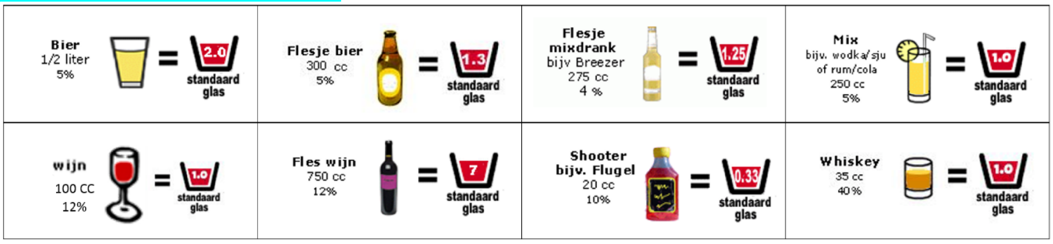

**Sum score AUDIT-C (possible range 0-12)**	__ __
**If AUDIT-C score ≥ 8 => Apply remaining AUDIT and PHQ-2 questionnaire**	

AUDIT (remaining scale)

Questions		0	1	2	3	4	Score
4	**How often during the last year have you found that you were not able to stop drinking once you had started?**	Never	Less than monthly	Monthly	Weekly	Daily or almost daily	
5	**How often during the last year have you failed to do what was normally expected from you because of drinking?**	Never	Less than monthly	Monthly	Weekly	Daily or almost daily	
6	**How often during the last year have you needed a first drink in the morning to get yourself going after a heavy drinking session?**	Never	Less than monthly	Monthly	Weekly	Daily or almost daily	
7	**How often during the last year have you had a feeling of guilt or remorse after drinking?**	Never	Less than monthly	Monthly	Weekly	Daily or almost daily	
8	**How often during the last year have you been unable to remember what happened the night before because you had been drinking?**	Never	Less than monthly	Monthly	Weekly	Daily or almost daily	
9	**Have you or someone else been injured as a result of your drinking?**	No		Yes, but not in the last year		Yes, during the last year	
10	**Has a relative or friend or a doctor or another health worker been concerned about your drinking or suggested you cut down?**	No		Yes, but not in the last year		Yes, during the last year	
	**Sum score (possible range 0-28)**						__ __
	**Sum score full AUDIT (possible range 0-40)**						

Jané-Llopis E, *et al. BMJ Open* 2020; 10:e038226. doi: 10.1136/bmjopen-2020-038226

Long Tally Sheet

	__ __
**If full AUDIT score ≥ 8 => Apply remaining AUDIT and PHQ-2 questionnaire**	

PHQ-2 Depression screening

Over the last 2 weeks, how often have you been bothered by any of the following problems?				
	**Not at all**	**Several days**	**More than half the days**	**Nearly every day**
**1 Little interest or pleasure in doing things**	0	1	2	3
**2 Feeling down, depressed, or hopeless**	0	1	2	3
**Sum score (possible range 0-6)**			__ __	
**If PHQ-2 score ≥ 3 => Apply remaining PHQ questionnaire**				

PHQ-9 (remaining scale)

Over the last 2 weeks, how often have you been bothered by any of the following problems?				
	**Not at all**	**Several days**	**More than half the days**	**Nearly every day**
**3 Trouble falling or staying asleep, or sleeping too much**	0	1	2	3
**4 Feeling tired or having little energy**	0	1	2	3
**5 Poor appetite or overeating**	0	1	2	3
**6 Feeling bad about yourself or that you are a failure or have let yourself or your family down**	0	1	2	3
**7 Trouble concentrating on things, such as reading the newspaper or watching television**	0	1	2	3
**8 Moving or speaking so slowly that other people could have noticed. Or the opposite being so figety or restless that you have been moving around a lot more than usual**	0	1	2	3
**9 Thoughts that you would be better off dead, or of hurting yourself**	0	1	2	3
**Sum score (possible range 0-21)**	__ __			
**Sum score full PHQ-9 (possible range 0-27)**	__ __			

Taking record of brief advice or referral

**If full AUDIT < 26 and PHQ-9 < 15:**	
**Brief advice** *(more than one answer is possible)*	☐ Oral Brief Advice given<br>☐ Patient Leaflet given<br>☐ Continued Monitoring<br>☐ Patient referred to other provider in practice for brief advice<br>☐ Patient referred to other provider outside practice for brief advice<br>☐ Other<br>______________________<br>☐ Time did not allow, but<br>☐ I made follow-up appointment

Long Tally Sheet

	☐ Patient declined brief advice<br>☐ Patient not screen positive, but reinforced about keeping low risk drinking habits	
**If full AUDIT ≥ 26 and/or PHQ-9 ≥ 15:**		
**Patient referred to special services:**		☐ Yes<br>☐ No

Tally Sheets Cover Form

Provider details, consultation and type of tally sheets
(to be filled in by local research team)

**Practice ID**	[pre-print]	**Provider ID / Name**	[pre-print]
**Consultation period**	____ / ____ / ____ - ____ / ____ / ____ (DD / MM / YY)		
**Type of tally sheets**	☐ Short tally sheets	☐ Long tally sheets	

Adult consultations
(to be filled in by PHC provider or administrator)

**Number of adult consultations during consultation period for this provider**	_ _ _ _

Tally Sheet Appendix

PHC provider and consultation details

**Practice ID** (*pre-printed*)	________________	**Provider ID / Name** (*pre-printed*)	________________
**Date consultation**	____ / ____ / ____		

Patient interview

**Alcohol screening result**	☐ Positive (AUDIT-C >= 8)	☐ Negative (AUDIT-C < 8)
**Asked patient for interview participation**	☐ Yes	☐ No
**Patient interested in interview participation**	☐ Yes	☐ No

Patient contact details for interview
(only if patient expressed interest in interview participation)

**Name**	____	
**Telephone number**	____	
**Address**	____	
**Preferred mode of interview**	☐ Face-to-face	☐ Telephone

Interview information

Introduction

The SCALA Study aims to find out the extent to which screening and brief advice implemented in primary health care can be increased to reduce the harmful use of alcohol. The study is taking place in cities from three countries from Latin America.
The harmful use of alcohol is prevalent in any countries, and alcohol, itself, is the seventh most important risk factor world-wide for ill-health and premature death (after high blood pressure, tobacco use, high fasting plasma glucose, high body mass index, poor diet, and low birthweight and short gestation).

Aim of the study

In this study, we aim to determine the extent of adequate prevention and management of harmful alcohol use in primary health care settings. Another major objective of this study is to improve the health of patients consulting primary health care centers.
The interview will take about 15 minutes and will cover questions on alcohol consumption, alcohol knowledge, wellbeing, and other health behavior. The same interview will be repeated twice, 3 and

9 months after the initial interview. Due to logistical reasons, not all patients agreed to be interviewed will eventually be asked for participation. If you have not been selected for interview participation, your contact details will be destroyed right away.

Data Handling and Sharing

Participation in this interview is entirely voluntary and you are free to skip any of the interview questions. During the interview, you will be asked questions on your personal wellbeing and health. The collected data will be entered into data bases and personal identifying information (such as name, address, and date of birth) will be replaced with an abstract personal identifier, the key to which remains with the local academic only. The data bases will be submitted to the data center at TU Dresden ('Technische Universität Dresden') in Germany using up-to-date encryption techniques. Here, all study data will be stored on encrypted hard drives and processed for further data analyses to be conducted by the study team. At all times, both analogue and digital data will be stored in secure environments. After publication of the study results, the relevant study data will be shared through the UK Data Service – a non-commercial data respository allowing other researchers to re-use the collected data for an indefinite period of time. All data shared through the UK Data Service will bear no risk of disclosure of the identity of the PHCC or of the participating providers.

Interview consent

Please check box

1. I confirm that I have read and understand the information for participating in the SCALA patient interview and have had the opportunity to ask questions. ☐

1. I consent that my contact details will be given to the SCALA study team and agree that the SCALA study team can use the contact details to ask me for interview participation and for repeating the interview. ☐

2. I understand that my participation is voluntary and that I am free to not participate, without giving any reason. ☐

3. I confirm that I have understand that study data collected through me will be processed at the TU Dresden (Germany) and shared through the UK Data Service. ☐

4.

________________________	______________	___________________
Name of patient	Date	Signature

PATIENT INTERVIEW

Formalities

**Practice ID** *(pre-printed)*	____________	**Provider ID / Name** *(pre-printed)*	____________
**Patient ID** *(filled in by interviewer)*	____________	**Interview date**	____ / ____ / ____

Sociodemographics

**Sex**	☐ Male<br>☐ Female<br>☐ Other	**Age**	______ years
**Socioeconomic status**	☐ Below average	☐ Average	☐ Above average
**Highest level of education**	☐ No schooling completed<br>☐ Junior high school completed<br>☐ Business/Technical training<br>☐ Doctorate degree		☐ Primary school completed<br>☐ High school completed<br>☐ Bachelor's/Master's degree

Alcohol exposure, health literacy, and social norms

During the last 12 months have you tried to cut down on your drinking by:		
Choosing lower strength alcohol	☐ Yes	☐ No
Using smaller glasses	☐ Yes	☐ No
How easy is it to understand health information about drinking of alcohol?	☐ Always easy<br>☐ Usually easy	☐ Sometimes difficult<br>☐ Often difficult<br>☐ Always difficult
In the last 12 months, has any doctor or health worker asked you about how much alcohol you drink?	☐ Yes	☐ No
In the last 12 months, has any doctor or health worker advised you to reduce or stop drinking alcohol?	☐ Yes	☐ No
To the best of your knowledge, can drinking alcohol cause any of the following:		
High blood pressure	☐ Yes	☐ No
Liver problems	☐ Yes	☐ No
Cancer	☐ Yes	☐ No
Thinking about your friends, would you say that it is acceptable or unacceptable for them to drink:		
Regularly more than two drinks a day?	☐ Acceptable	☐ Unacceptable
More than six drinks on an occasion?	☐ Acceptable	☐ Unacceptable

AUDIT Alcohol Screening

Questions		0	1	2	3	4	Score
1	How often do you have a drink containing alcohol?	Never	Monthly or less	2-4 times per month	2-3 times per week	4+ times per week	
2	How many units of alcohol do you drink on a typical day when you are drinking?	1-2	3-4	5-6	7-9	10+	
3	How often do you have 6 or more units on one occasion?	Never	Less than monthly	Monthly	Weekly	Daily or almost daily	

Standard Drinks Placeholder

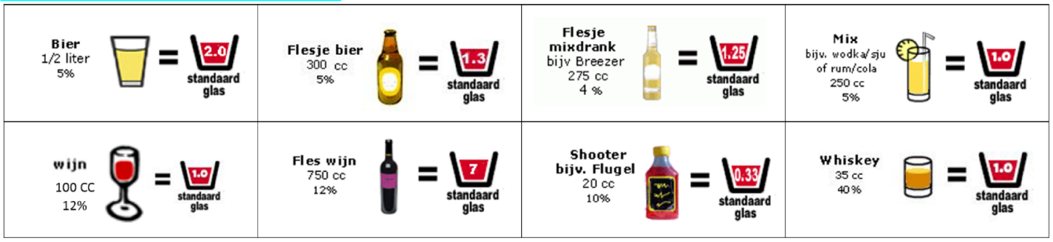

		0	1	2	3	4	Score
4	How often during the last year have you found that you were not able to stop drinking once you had started?	Never	Less than monthly	Monthly	Weekly	Daily or almost daily	
5	How often during the last year have you failed to do what was normally expected from you because of drinking?	Never	Less than monthly	Monthly	Weekly	Daily or almost daily	
6	How often during the last year have you needed a first drink in the morning to get yourself going after a heavy drinking session?	Never	Less than monthly	Monthly	Weekly	Daily or almost daily	
7	How often during the last year have you had a feeling of guilt or remorse after drinking?	Never	Less than monthly	Monthly	Weekly	Daily or almost daily	
8	How often during the last year have you been unable to remember what happened the night before because you had been drinking?	Never	Less than monthly	Monthly	Weekly	Daily or almost daily	
9	Have you or someone else been injured as a result of your drinking?	No		Yes, but not in the last year		Yes, during the last year	
10	Has a relative or friend or a doctor or another health worker been concerned about your drinking or suggested you cut down?	No		Yes, but not in the last year		Yes, during the last year	
	Sum score AUDIT (possible range 0-40)						__ __

PHQ-9 Depression Screening

Over the last 2 weeks, how often have you been bothered by any of the following problems?					
		Not at all	Several days	More than half the days	Nearly every day
1	Little interest or pleasure in doing things	0	1	2	3
2	Feeling down, depressed, or hopeless	0	1	2	3
3	Trouble falling or staying asleep, or sleeping too much	0	1	2	3
4	Feeling tired or having little energy	0	1	2	3
5	Poor appetite or overeating	0	1	2	3
6	Feeling bad about yourself or that you are a failure or have let yourself or your family down	0	1	2	3
7	Trouble concentrating on things, such as reading the newspaper or watching television	0	1	2	3
8	Moving or speaking so slowly that other people could have noticed. Or the opposite being so figety or restless that you have been moving around a lot more than usual	0	1	2	3
9	Thoughts that you would be better off dead, or of hurting yourself	0	1	2	3
	Sum score PHQ-9 (possible range 0-27)	__ __			

Alcohol Literacy Assessment

On a scale from very difficult to very easy, how easy would you say it is to: …						
		Very diffic ult	Fairly difficul t	Fairly easy	Very easy	Don't know
1	Question 1 Placeholder	0	1	2	3	5
2	Question 2 Placeholder	0	1	2	3	5
3	Question 3 Placeholder	0	1	2	3	5
4	Question 4 Placeholder	0	1	2	3	5
5	Question 5 Placeholder	0	1	2	3	5
6	Question 6 Placeholder	0	1	2	3	5
	Sum score (possible range XX-XX)	__ __				

WHODAS 2.0 Disability Assessment

This questionnaire asks about difficulties due to health conditions. Health conditions include diseases or illnesses, other health problems that may be short or long lasting, injuries, mental or emotional problems, and problems with alcohol or drugs.

Think back over the past 30 days and answer these questions, thinking about how much difficulty you had doing the following activities. For each question, please circle only one response.

In the past 30 days, how much difficulty did you have in:						
	Questions	None	Mild	Moderate	Severe	Extreme or cannot do
1	Standing for long periods such as 30 minutes?	1	2	3	4	5
2	Taking care of your household responsibilities?	1	2	3	4	5
3	Learning a new task, for example, learning how to get to a new place?	1	2	3	4	5
4	Joining in community activities (for example, festivities, religious or other activities) in the same way as anyone else can?	1	2	3	4	5
5	How much have you been emotionally affected by your health problems?	1	2	3	4	5
6	Concentrating on doing something for ten minutes?	1	2	3	4	5
7	Walking a long distance such as a kilometre [*or equivalent*]?	1	2	3	4	5
8	Washing your whole body?	1	2	3	4	5
9	Getting dressed?	1	2	3	4	5
10	Dealing with people you do not know?	1	2	3	4	5
11	Maintaining a friendship?	1	2	3	4	5
12	Your day-to-day work?	1	2	3	4	5
	Sum score (possible range 0-60)	__ __				
H1	Overall, in the past 30 days, how many days were these difficulties present?	Record number of days: __ __ (0-30)				
H2	In the past 30 days, for how many days were you totally unable to carry out your usual activities or work because of any health condition?	Record number of days: __ __ (0-30)				
H3	In the past 30 days, not counting the days that you were totally unable, for how many days did you cut back or reduce your usual activities or work because of any health condition?	Record number of days: __ __ (0-30)				

Health resource utilization

Title Placeholder				
		**Response 1**	**Response 2**	**Response 3**
**1**	Question 1 Placeholder	0	1	2
**2**	Question 2 Placeholder	0	1	2
**3**	Question 3 Placeholder	0	1	2
**4**	Question 4 Placeholder	0	1	2
**5**	Question 5 Placeholder	0	1	2
**6**	Question 6 Placeholder	0	1	2

Jané-Llopis E, *et al. BMJ Open* 2020; 10:e038226. doi: 10.1136/bmjopen-2020-038226

Primary Health Care Provider Questionnaire

Practice details and date

**Practice ID** (*pre-printed*)	__________________	**Provider ID / Name** (*pre-printed*)	__________________
**Date**	____ / ____ / ____	**Assessment**	☐ Baseline<br>☐ Follow-up 1<br>☐ Follow-up 2

Patient details

**Sex**	☐ Male<br>☐ Female<br>☐ Other	**Age**	_______ years
**Profession**	☐ Doctor<br>☐ Nurse<br>☐ Psychologist		☐ Practice Assistant<br>☐ Social worker<br>☐ Other: _______

Alcohol Knowledge

	Questions	Per Day	Per Week	Per Occasion
1	**Experts recommend that everyone should limit the amount of alcohol that they drink. What is this limit for men, in terms of drinks:**	_ _ drinks	_ _ drinks	_ _ drinks
2	**Experts recommend that everyone should limit the amount of alcohol that they drink. What is this limit for women, in terms of drinks:**	_ _ drinks	_ _ drinks	_ _ drinks
		**Acceptable**	**Unacceptable**	
3	**Would you say that it is acceptable or unacceptable for you to drink regularly more than two drinks a day?**			
4	**Would you say that it is acceptable or unacceptable for you to drink more than six drinks on anyone occasion?**			
5	**Would you say that it is acceptable or unacceptable for your friends to drink regularly more than two drinks a day?**			
6	**Would you say that it is acceptable or unacceptable for your friends to drink more than six drinks on anyone occasion?**			

Alcohol Health Literacy

On a scale from very difficult to very easy, how easy would you say it is to: …

		Very difficult	Fairly difficult	Fairly easy	Very easy	Don't know
1	**Question 1 Placeholder**	0	1	2	3	5
2	**Question 2 Placeholder**	0	1	2	3	5

Primary Health Care Provider Questionnaire

**3**	**Question 3 Placeholder**	0	1	2	3	5
**4**	**Question 4 Placeholder**	0	1	2	3	5
**5**	**Question 5 Placeholder**	0	1	2	3	5
**6**	**Question 6 Placeholder**	0	1	2	3	5
	**Sum score (possible range XX-XX)**	__ __				

The Short Alcohol and Alcohol Problems Perception Questionnaire

	**There are no right or wrong answers. Please indicate the extent to which you agree or disagree with the following statements**	**Strongly disagree**	**Quite strongly disagree**	**Disagree**	**Neither agree or disagree**	**Agree**	**Quite strongly agree**	**Strongly agree**
		**1**	**2**	**3**	**4**	**5**	**6**	**7**
1	I feel I know enough about causes of drinking problems to carry out my role when working with drinkers							
2	I feel I can appropriately advise my patients about drinking and its effects							
3	I feel I do not have much to be proud of when working with drinkers							
4	All in all, I am inclined to feel I am a failure with drinkers							
5	I want to work with drinkers							
6	Pessimism is the most realistic attitude to take towards drinkers							
7	I feel I have the right to ask patients questions about their drinking when necessary							
8	I feel that my patients believe I have the right to ask them questions about drinking when necessary							
9	In general, it is rewarding to work with drinkers							
10	In general, I like drinkers							

Annexe 25 Provider Interview

Telephone Interview of random sample of providers

Approximately 15-minute recorded telephone interview with open-ended questions

Country:

City:

PHCU ID Number:

PHC Provider ID Number:

Why?
Engagement: reasons for participating in the PHC action

How and for whom?
Description of the implementation process for screening and brief advice: description of proceedings and expectations of screening and brief advice

Under what circumstances?
What were the barriers and facilitators to following the guidelines on risky alcohol consumption?

What were the facilitators or barriers to implementing screening and brief advice?

Opinions and suggestions for organisational and political barriers and facilitators

Other thoughts and suggestions to speed up the implementation process

The responses will be analysed and coded according to Keurhorst et al. 2016:

Keurhorst M, Heinen M, Colom J et al. Strategies in primary healthcare to implement early identification of risky alcohol consumption: why do they work or not? A qualitative evaluation of the ODHIN study. Keurhorst et al. BMC Family Practice (2016) 17:70 DOI 10.1186/s12875-016-0461-8

SCALA – Documentation of PHCC Recruitment

1) Please specify the country as well as the name of the researcher responsible for PHCC recruitment:

**Country**	☐ Mexico<br>☐ Colombia<br>☐ Peru
**Responsible researcher**	____________________

2) During recruitment of the PHCCs, local researchers should document the following points *for each municipality*:

**Name of municipality**	____________________
**Control / Intervention**	☐ Control<br>☐ Intervention
**Total number of PHCCs in municipality**	____________________
**Number of PHCCs contacted for study participation**	____________________
**Number of non-responding PHCCs**	____________________
**Number of PHCCs refusing to participate**	____________________
**Number of PHCCs accepting to participate**	____________________

Jané-Llopis E, *et al. BMJ Open* 2020; 10:e038226. doi: 10.1136/bmjopen-2020-038226

3) Further, the following points need to be documented *for each contacted PHCC*:

**Name/Address/Identifier of PHCC**	____________________
**Characteristics of PHCC (if known)**	☐ Number of registered patients: _____<br>☐ Number of GPs: _____<br>☐ Number of nurses: _____<br>☐ Number of all workers: _____<br>☐ other:<br>______________________________
**Contact with PHCC**	☐ By mail<br>☐ By email<br>☐ By telephone<br>☐ Personal contact<br>☐ other:<br>______________________________
**Number of contacts with PHCC before decision (acceptance/refusal/non-response)**	____________________
**Accepted / Refused / No response**	☐ Accepted<br>☐ Refused<br>☐ No response
**If refused, give reasons**	_______________________________________<br>_______________________________________<br>_______________________________________
**If no response, any reasons suspected?**	_______________________________________<br>_______________________________________<br>_______________________________________

SCALA – Provider follow-up documentation

Provider details

During the course of the study, each PHC provider should be followed up with regard to participation in training sessions. Further, potential drop outs should be documented here. Please fill in this sheet ***for each provider.***

**Country**	☐ Mexico<br>☐ Colombia<br>☐ Peru
**Responsible researcher**	____________________
**Name of municipality**	____________________
**Control / Intervention**	☐ Control<br>☐ Intervention
**Name/Address/Identifier of PHCC**	____________________
**Name/Identifier of provider**	____________________
**Gender of provider**	☐ Female<br>☐ Male<br>☐ Other
**Age of provider**	____________________ (in years of age)
**Baseline month**	from ____ / ____ / ____ until ____ / ____ / ____<br>(DD / MM / YY)

Jané-Llopis E, *et al. BMJ Open* 2020; 10:e038226. doi: 10.1136/bmjopen-2020-038226

Participation in training sessions

**Training session**	☐<br>☐<br>☐<br>☐	Pre-implementation Training 1<br>Pre-implementation Training 2<br>Booster 1<br>Booster 2
**Date of training**	____ / ____ / ____ (DD / MM / YY)	
**Training participation**	☐<br>☐	Participated in training<br>Absent in training
**Reason for training absence**	☐<br>☐	with valid excuse, ie. _________________<br>without valid excuse
**If absent at training, could training be repeated?**	☐<br>☐	Yes, on ____ / ____ / ____ (DD / MM / YY)<br>No

Drop out

If the provider dropped out before end of the study, the following section need to be filled in:

**Date of drop out**	____ / ____ / ____ (DD / MM / YY)
**Date of last tally sheet completed by provider**	____ / ____ / ____ (DD / MM / YY)
**Drop out in relation to data collection**	☐ Before baseline data collection<br>☐ During baseline data collection<br>☐ After baseline data collection, but before 18-month implementation period<br>☐ During specific month of 18-month implementation period (enter number of month from 1 to 18).
**Reasons for drop out**	____<br>____<br>____<br>____<br>____

BMJ Publishing Group Limited (BMJ) disclaims all liability and responsibility arising from any reliance placed on this supplemental material which has been supplied by the author(s)

RE-AIM Dimension, SCALA aims	SCALA activities	Main outcome/process measures
**REACH**<br>• In PHC, to maximise exposure to screening for AUD<br>• In PHC, to maximise exposure to advice and treatment for AUD and comorbid depression<br>• In PHC, to maximise exposure to alcohol health literacy information materials	• Recruitment of PHCCs in each city with large population coverage of about 160,000 registered patients per PHCC<br>• Recruitment of representative PHCC population within cities to maximise<br>– Take-up of alcohol health literacy information materials<br>– Numbers screened for AUD<br>– Numbers receiving appropriate advice/referral for AUD/depression	• Total number of PHCC patients screened for AUD<br>• Total number of screen positive patients receiving appropriate advice/referral for AUD/depression<br>• Representativeness of population screened and/or receiving appropriate advice/referral for AUD
**EFFECTIVENESS**<br>• To design and apply an evidence-based care pathway to address AUD and comorbid depression in primary health care	Design and delivery of an intervention package within a primary health care based care pathway that incorporates:<br>• State-of-the-art alcohol health literacy information materials<br>• AUDIT-C screening instrument<br>• Brief advice and treatment for case positives<br>• Referral of severe AUD and comorbid depression	• Increased health literacy in PHCC patients using UK-based Newest Vital Sign and an adapted version of Health Literacy Survey-EU Questionnaire (HLS-EU Q)<br>• Reduction in alcohol consumption of AUD+ drinkers
**ADOPTION**<br>• To increase the adoption of the intervention package in primary health care	• Design of a pragmatic, easy to use and replicate PHCC intervention package and associated care pathway<br>• Tailoring of the PHCC package according to local needs (PHC setting, PHCC) by using Community Advisory Boards (CABs) and User Panels (UPs)<br>• Provision of specific practice-based training and ongoing support to PHCC<br>• Development of city-based adoption mechanisms and support systems	• Adoption rate and representativeness of PHCCs<br>• Adoption rate and representativeness of PHCC staff
**IMPLEMENTATION**<br>• To assess the fidelity and costs of implementing the intervention package<br>• To evaluate which factors affect the implementation of the intervention package	• Continuous feedback on PHCC level drivers to package implementation gathered via qualitative and quantitative metrics<br>• Application of WHO Urban Health Equity Assessment and Response Tool<br>• Application of MRC framework to map and understand progress towards effective scale-up	• Extent primary health care screening and advice package delivered as intended<br>• Multi-level evaluation of barriers/facilitators to scale-up using WHO's Urban Health Equity Assessment and Response Tool<br>• Extent implementation on city levels delivered as intended using Medical Research Council guidance<br>• Cost of package implementation
**MAINTENANCE**<br>• To report on long-term effects of package at individual and organisational levels<br>• To understand how the programme can be maintained and achieve longevity within the test cities	• Support at the system level to make relevant practice changes for sustainability<br>• Monitoring system on long-term effectiveness<br>• Monitoring system on performance on PHCC level<br>• Production of Step-by-step SCALA Framework and Strategy	• Assessment of outcomes 18 months post implementation<br>• Indicators of program-level maintenance<br>• Measures of cost of maintenance<br>• Dissemination / events
