# Supplementary material for: Implementing primary healthcare-based measurement, advice and treatment for heavy drinking and comorbid depression at the municipal level in three Latin American countries: final protocol for a quasiexperimental study (SCALA study)
Source: BMJ Open. 2020 Jul 28;10(7):e038226. doi: 10.1136/bmjopen-2020-038226 (PMC7390229; doi:10.1136/bmjopen-2020-038226)
Supplement: Author's manuscript [file bmjopen-2020-038226.draft_revisions.pdf]

BMJ Open is committed to open peer review. As part of this commitment we make the peer review history of every article we publish publicly available.

When an article is published we post the peer reviewers' comments and the authors' responses online. We also post the versions of the paper that were used during peer review. These are the versions that the peer review comments apply to.

The versions of the paper that follow are the versions that were submitted during the peer review process. They are not the versions of record or the final published versions. They should not be cited or distributed as the published version of this manuscript.

BMJ Open is an open access journal and the full, final, typeset and author-corrected version of record of the manuscript is available on our site with no access controls, subscription charges or pay-per-view fees (http://bmjopen.bmj.com).

If you have any questions on BMJ Open's open peer review process please email info.bmjopen@bmj.com

BMJ Open

Implementing primary health care-based measurement, advice and treatment for heavy drinking and comorbid depression at the municipal level in three Latin American countries: final protocol for a quasi-experimental study (SCALA study)

Journal:	*BMJ Open*
Manuscript ID	bmjopen-2020-038226
Article Type:	Protocol
Date Submitted by the Author:	03-Mar-2020

Complete List of Authors:	Jane LLopis, Eva; Ramon Llull University<br>Anderson, Peter; Maastricht Univ; Newcastle University, Institute Health and Society<br>Piazza, Marina; Universidad Peruana Cayetano Heredia<br>O'Donnell, Amy; Newcastle University, Institute of Health and Society<br>Gual, Antoni; Hospital Clínic de Barcelona, Addiction Unit<br>Schulte, Bernard; University Medical Center Hamburg-Eppendorf<br>Perez, Augusto; Corporación Nuevos Rumbos<br>de Vries, Hein; Maastricht University (CAPHRI), Health Promotion; Maastricht University<br>Natera Rey, Guillermina; Instituto Nacional de Psiquiatría Ramón de la Fuente Muñiz<br>Kokole, Dasa; Maastricht University Faculty of Health Medicine and Life Sciences<br>Bustamante, Ines; Universidad Peruana Cayetano Heredia<br>Braddick, Fleur; Hospital Clinic de Barcelona<br>Mejía Trujillo, Juliana; Corporación Nuevos Rumbos<br>Solovei, Adriana; Maastricht Universitair Medisch Centrum+<br>Pérez De León, Alexandra; Instituto Nacional de Psiquiatría Ramón de la Fuente Muñiz<br>Kaner, Eileen; Newcastle University, Institute<br>Matrai, Silvia; Hospital Clinic de Barcelona<br>Manthey, Jakob; Technische Universitat Dresden,<br>Mercken, Liesbeth; Maastricht University<br>López-Pelayo, Hugo; Hospital Clínic de Barcelona, Addiction Unit<br>Rowlands, Gillian; Newcastle University Institute for Health and Society,<br>Schmidt, CS; Universitatsklinikum Hamburg-Eppendorf,<br>Rehm, Jürgen; Centre for Addiction and Mental Health,

Keywords:	PRIMARY CARE, Substance misuse < PSYCHIATRY, Depression & mood disorders < PSYCHIATRY

SCHOLARONE™
Manuscripts

I, the Submitting Author has the right to grant and does grant on behalf of all authors of the Work (as defined in the below author licence), an exclusive licence and/or a non-exclusive licence for contributions from authors who are: i) UK Crown employees; ii) where BMJ has agreed a CC-BY licence shall apply, and/or iii) in accordance with the terms applicable for US Federal Government officers or employees acting as part of their official duties; on a worldwide, perpetual, irrevocable, royalty-free basis to BMJ Publishing Group Ltd ("BMJ") its licensees and where the relevant Journal is co-owned by BMJ to the co-owners of the Journal, to publish the Work in this journal and any other BMJ products and to exploit all rights, as set out in our licence.

The Submitting Author accepts and understands that any supply made under these terms is made by BMJ to the Submitting Author unless you are acting as an employee on behalf of your employer or a postgraduate student of an affiliated institution which is paying any applicable article publishing charge ("APC") for Open Access articles. Where the Submitting Author wishes to make the Work available on an Open Access basis (and intends to pay the relevant APC), the terms of reuse of such Open Access shall be governed by a Creative Commons licence – details of these licences and which Creative Commons licence will apply to this Work are set out in our licence referred to above.

Other than as permitted in any relevant BMJ Author's Self Archiving Policies, I confirm this Work has not been accepted for publication elsewhere, is not being considered for publication elsewhere and does not duplicate material already published. I confirm all authors consent to publication of this Work and authorise the granting of this licence.

For peer review only

Implementing primary health care-based measurement, advice and treatment for heavy drinking and comorbid depression at the municipal level in three Latin American countries: final protocol for a quasi-experimental study (SCALA study)

Eva Jané Llopis[1-3]
Peter Anderson[2,4]
Marina Piazza[5]
Amy O'Donnell[4]
Antoni Gual[6-8]
Bernd Schulte[9]
Augusto Pérez Gómez[10]
Hein de Vries[2]
Guillermina Natera Rey[11]
Dasa Kokole[2]
Ines V. Bustamante [5]
Fleur Braddick[7]
Juliana Mejía Trujillo[10]
Adriana Solovei[2]
Alejandra Pérez De León[11]
Eileen Kaner[4]
Silvia Matrai[7]
Jakob Manthey[9,12]
Liesbeth Mercken[2]
Hugo Lopez-Pelayo[6-8]
Gill Rowlands[4]
Christiane Schmidt[9]
Jürgen Rehm[3, 12-15]

[1] Univ. Ramon Llull, ESADE, Barcelona, Spain. Email: eva.jane@esade.edu

[2] Department of Health Promotion, CAPHRI Care and Public Health Research Institute, Maastricht University, POB 616, 6200 MD, Maastricht, The Netherlands. Email: e.llopis@maastrichtuniversity.nl; peter.anderson@maastrichtuniversity.nl; hein.devries@maastrichtuniversity.nl; d.kokole@maastrichtuniversity.nl; a.solovei@maastrichtuniversity.nl; Liesbeth.mercken@maastrichtuniversity.nl

[3] Institute for Mental Health Policy Research, CAMH, 33 Russell Street, Toronto, ON M5S 2S1, Canada. Email: evajanellopis@gmail.com; jtrehm@gmail.com

[4] Population Health Sciences Institute, Newcastle University, Baddiley-Clark Building, Richardson Road, Newcastle upon Tyne NE2 4AX, UK. Emails: Peter.Anderson@newcastle.ac.uk; amy.odonnell@newcastle.ac.uk; eileen.kaner@newcastle.ac.uk

[5]School of Public Health and Administration, Universidad Peruana Cayetano Heredia e-mail: marina.piazza@upch.pe; ines.bustamante@upch.pe

For peer review only - http://bmjopen.bmj.com/site/about/guidelines.xhtml

[6] Red de Trastornos Adictivos. Instituto Carlos III. Sinesio Delgado, 4; 28029 – Madrid. Email: TGUAL@clinic.cat; hlopez@clinic.cat

[7]Addictions Unit. Psychiatry Dept. Hospital Clínic. Villarroel 170; 08036 Barcelona, Spain. Email: TGUAL@clinic.cat, hlopez@clinic.cat; smatrai@clinic.cat; fmbooth@clinic.cat

[8] Institut d'Investigacions Biomèdiques August Pi Sunyer (IDIBAPS); Rosselló, 149-153; 08036 Barcelona, Spain. Email: TGUAL@clinic.cat; hlopez@clinic.cat

[9] Center for Interdisciplinary Addiction Research (ZIS), Department of Psychiatry and Psychotherapy, University Medical Center Hamburg-Eppendorf, Hamburg, Germany. Email: b.schulte@uke.uni-hamburg.de; christiane.schmidt@uke.de; jakobmanthey@snappyquest.org

[10] Corporación Nuevos Rumbos, Calle 108 A # 4-15, Bogotá, Colombia. Email: aperez@nuevosrumbos.org; jmejia@nuevosrumbos.org

[11] Instituto Nacional de Psiquiatría Ramón de la Fuente Muñiz, Calz México-Xochimilco 101, Huipulco, 14370 Ciudad de México, CDMX, Mexico. Email: naterar@imp.edu.mx; apdeleon@imp.edu.mx

[12] Institute for Clinical Psychology and Psychotherapy, TU Dresden, Chemnitzer Str. 46, 01187 Dresden, Germany. Email: jtrehm@gmail.com; jakob.manthey@tu-dresden.de

[13] Dalla Lana School of Public Health, University of Toronto, 155 College Street, 6th Floor, Toronto, ON M5T 3M7, Canada. Email: jtrehm@gmail.com

[14] Department of Psychiatry, University of Toronto, 250 College Street, 8th Floor, Toronto, ON M5T 1R8, Canada. Email: jtrehm@gmail.com

[15] Department of International Health Projects, Institute for Leadership and Health Management, I.M. Sechenov First Moscow State Medical University, Trubetskaya str., 8, b. 2, 119992, Moscow, Russian Federation jtrehm@gmail.com

Corresponding author: Eva Jané Llopis: eva.jane@esade.edu

For peer review only

Abstract

Introduction: While primary health care-based prevention and management of heavy drinking is clinically effective and cost-effective, it remains poorly implemented in routine practice. Systematic reviews and multi-country studies have demonstrated the ability of training and support programmes for healthcare professionals to increase primary health care-based measurement and brief advice activity to reduce heavy drinking. However, gains have been only modest and short term at best. WHO studies have concluded that a more effective uptake could be achieved by embedding primary health care activity within broader municipal-based support.

Methods and analysis: A quasi-experimental study will compare primary health care-based prevention and management of heavy drinking in three intervention municipal areas from Colombia, Mexico and Peru with three comparator municipal areas from the same countries. In the implementation municipal areas, primary health care units will receive training embedded within ongoing supportive municipal action over an 18-month implementation test period. In the comparator municipal areas, half the units will receive training, and the other half will continue with practice as usual. The primary outcome is the proportion of the adult population (aged 18+ years) registered with the unit that has their alcohol consumption measured. Return-on-investment analyses and full process evaluation will be undertaken, coupled with an analysis of potential contextual, financial and political-economy influencing factors.

Ethics and dissemination: The Ethics Committee of the Technical University of Dresden gave final ethical approval for the SCALA project on 12 April 2019, EK90032018. A dissemination strategy is in place with Ministries of Health at municipal and country levels; and, with Pan American Health Organization at Latin American level to scale up the implementation strategy, once validated.

Trial Registration: Clinical Trials.gov ID: NCT03524599; Registered 15 May 2018; https://clinicaltrials.gov/ct2/show/NCT03524599

Protocol Version: Final version, 25 February 2020.

Key words: Primary health care; municipal action; heavy drinking; comorbid depression; Institute for Health Care Improvement; implementation; measurement of alcohol consumption; AUDIT-C.

For peer review only

For peer review only - http://bmjopen.bmj.com/site/about/guidelines.xhtml

Strengths and Limitations of Study

1. Uses a theory-based approach to tailor clinical materials and training programmes, creating city-based Community Advisory Boards, and user-based User Panels to ensure that tailoring matches user needs, municipal services, and co-production of health;
2. Tests the added value of embedding and implementing primary health care activity within municipal-based adoption mechanisms and support systems, and community-based communication campaigns;
3. Has a longer time frame (18 months) than is traditionally used in implementation studies, to assess longer term impacts;
4. Gives considerable emphasis to process evaluation, developing logic models to document the fidelity of all implementation strategies, and to identify, the drivers and barriers and facilitators to successful implementation and scale-up; and
5. Due to municipal-based political and technical considerations, we are unable to randomize the involved municipal areas. We adopt a quasi-experimental design, optimizing comparator municipal areas for confounding, and by using propensity score matching.

For peer review only - http://bmjopen.bmj.com/site/about/guidelines.xhtml

INTRODUCTION

This paper outlines the protocol for a quasi-experimental study[1] to test the implementation of primary health care-based measurement, advice and treatment for heavy drinking and comorbid depression at the municipal level in three Latin American countries, Colombia, Mexico and Peru (SCALA study).

Heavy drinking is a cause of considerable disability, morbidity, and mortality[2]. Heavy drinking is a causal factor for some communicable diseases (including TB and HIV/AIDS), for many non-communicable diseases (including cancers, cardiovascular diseases and gastrointestinal diseases) and for many mental and behavioural disorders, including depression, dementias and suicide[3,4].

In PHC settings, two-fifths of people with heavy drinking have depression, with risks of incident depression higher for heavier as opposed to lighter drinkers[5]. In addition to its role in the aetiology of depression, heavy drinking is associated with worsening the depression course, including suicide risk, impaired social functioning and impaired health care utilization[6]. Given the strong comorbidity between heavy drinking and depression, our protocol includes screening for depression for those patients identified as heavy drinkers, with appropriate referral or PHC support for treatment.

Heavy drinking is also a major contributor to global health inequalities, with alcohol-related harm aggravated by lower socio-economic status[7] and extending beyond the individual drinker to families, communities, health systems, and the wider economy. Tackling the multiple individual and societal level harms caused by heavy drinking is essential for achieving global targets of reducing deaths from NCDs by 25% between 2010 and 2025[8], more so as risk of exposure to harmful use of alcohol increases with increasing socio-economic status[9]. In line with tackling harm due to lower socio-economic status, United Nations Sustainable Development Goals include Target 3.5, to strengthen the prevention and treatment of harmful use of alcohol, with two proposed indicators: coverage of treatment interventions (pharmacological, psychosocial and rehabilitation and aftercare services) for harmful use of alcohol; and per capita alcohol consumption[10,11].

Countries in Latin America have the highest alcohol-attributable disease burden after Eastern Europe and Sub-Saharan Africa, with particularly high risks in alcohol-attributable traffic injury including violence [12]. The burden of alcohol-attributable diseases in Latin America lead to marked economic costs, with numerous calls to implement effective and cost-effective policies (e.g.[13]).

A robust and extensive body of literature demonstrates the range of evidence-based strategies that can be implemented to reduce heavy drinking in health care settings[14]. Questionnaire-based measurement and brief advice programmes delivered in PHC are effective[15] and cost-effective[16,17] in reducing heavy drinking. In addition to brief advice, treatment for heavy drinking includes cognitive behavioural therapy and pharmacotherapy, both of which are found to be effective in reducing heavy drinking[18]. Were the proportion of eligible patients receiving advice and treatment for heavy drinking to increase to 30% of eligible patients, the prevalence of harmful use of alcohol could decrease by between 10% and 15% across OECD member countries[19]. However, to date, measurement and brief advice and treatment programmes have failed to achieve widespread take-up[19].

For peer review only - http://bmjopen.bmj.com/site/about/guidelines.xhtml

Two systematic reviews[20,21] and two multi-country studies[22-24] have demonstrated that the proportion of PHC patients whose alcohol consumption is measured, and of heavy drinking patients given advice can be increased by providing training and support to PHC providers, albeit from very low baseline levels, and with effects not generally sustained over the longer term. Moreover, whilst there has been some previous research in countries of Latin America[25-30], most implementation work to date has been undertaken in high-income countries. The SCALA study will build on previous evidence[31] to fast-track scale-up research and practice in Latin American primary health care settings.

Out of a range of implementation frameworks that include a sequential approach for scale-up, and that provide practical guidance for how to work with organizations, health systems, and communities to implement and scale-up best practices[32-39], we adopt the Institute for Healthcare Improvement's (IHI) Framework for going to Full Scale, which identifies adoption mechanisms and support systems for use across sequential steps, and describes the implementation methods that can be used at each step[40].

SCALA seeks to address three specific barriers to sustained implementation of primary health care-based measurement, advice and treatment for heavy drinking. The first barrier recognizes that most PHC-based programmes focus on providers alone, whereas successful implementation of health interventions within complex health system demands addressing a range of underlying structural and support systems[40]. Phase IV of the WHO study on the identification and management of alcohol-related problems in primary care concluded that embedding PHC-based measurement and brief advice programmes within the frame of supportive community and municipal environments might lead to improved outcomes[41], although this has never been formally evaluated. Similar conclusions were reached by the European ODHIN study[42] and the US-based SAMHSA SBIRT initiative[43-45].

The second barrier is that standard cut-off points for the frequently used alcohol measurement instrument, AUDIT-C[46] (commonly a score of five for both men and women, or five for men and four for women) to trigger advice are too low[47], being equivalent to an average daily alcohol consumption of about 20 grams of alcohol (around 2 standard drinks) or less[48]. Practitioners may well find it problematic to give advice at such levels, which would also have huge time implications, with one in three or four patients being eligible for advice in many countries, under this criterion. We have argued to adopt similar models to blood pressure, where cut-off points for managing raised blood pressure are often determined by levels of blood pressure at which treatment has shown to be effective[49,50]. Similarly, cut-off points for brief advice could be the baseline levels of alcohol consumption found in the randomized controlled trials that have investigated the effectiveness of PHC-delivered brief advice. In the first Cochrane review of the topic that focused on primary health care, mean baseline levels were 313 grams of alcohol per week[51], equivalent to an AUDIT-C cut-off of 8[48].

The third and final barrier concerns the cost of implementing measurement and brief-advice for heavy drinking in primary health care setting. Although, alcohol advice and treatment programmes can lead to substantial reductions in health care costs[16], freeing considerable numbers of working age people from alcohol-related diseases[19], their initial implementation can require a significant time-commitment on the part of providers, in terms of both initial training requirements and the time taken

For peer review only - http://bmjopen.bmj.com/site/about/guidelines.xhtml

to deliver advice in routine practice. The largest part of the costs of implementing measurement and brief advice for heavy drinking in primary health care settings are directly caused by the time spent by the health care providers delivering this intervention[54]. Moreover, this large amount of time is experienced by health care providers as an important barrier to deliver routine measurement and brief advice to their patients[55]. As evidence suggests that shorter sessions of brief advice are not less effective compared to shorter sessions[51], it seems that reducing the time spent by health care professionals in preparing for these sessions could be a viable strategy to increase the overall adoption and implementation of alcohol measurement and brief advice at primary health care level.

In the SCALA study, we implement three interventions (independent variables) for the PHCU:

i. Intensity of clinical package and training (standard, versus short, versus none);
ii. Training of providers (present, versus absent); and,
iii. Community integration and support (municipal action present, versus absent).

The main outcome (dependent variable) is the cumulative proportion of the adult (aged 18+ years) population registered with the PHCU that has their alcohol consumption measured within the 18-month implementation test period (defined as coverage). Three hypotheses are to be tested:

Hypothesis 1: Municipal action leads to more sustainable coverage. After 18 months, the difference in coverage between municipal action present and municipal action absent is larger than after 12 months;

Hypothesis 2: Training leads to higher coverage than no training; and,

Hypotheses 3: In the presence of municipal action, the short clinical package and short training do not lead to less measurement coverage than the standard clinical package and standard training.

METHODS AND ANALYSIS

The study is a quasi-experimental design[1], comparing changes in measurement and assessment for alcohol consumption and comorbid depression, and, if needed, advice and/or referral for treatment between primary health care units (PHCUs) in intervention municipal areas and PHCUs in similar comparator municipal areas. In 2017, prior to a grant application, we published a pre-protocol for a three-country study to test the scale-up of primary health care-based programmes to identify and manage the harmful use of alcohol and comorbid depression[56]. Since the application, and during the grant negotiation and planning phase, the design of the study has changed considerably, essentially moving from a two-arm design to a four-arm design, and changing the primary outcome measure to the proportion of the adult population registered with a PHCU that has their alcohol consumption measured, Supplement Box 1. With all changes approved by the concerned ethics committee, this paper outlines the final protocol for a quasi-experimental study to test the implementation of primary health care-based measurement, advice and treatment for heavy drinking and comorbid depression at the community level in three Latin American countries, Colombia, Mexico and Peru (SCALA study).

For peer review only - http://bmjopen.bmj.com/site/about/guidelines.xhtml

Intervention municipal areas are investigator-selected from Bogotá (Colombia), Mexico City (Mexico) and Callao – Lima (Peru). Comparator municipal areas are investigator-selected in the same cities, on the basis of comparability with the intervention municipal area in terms of socio-economic and other characteristics which impact on drinking, health care and survival, comparable community mental health services, and sufficient geographical separation to minimize spill over effects from the intervention municipal area. Randomized selection of the municipal areas was not feasible due to organizational limitations. Municipal areas are chosen as a scalable implementation unit at mesosystem level that can be replicated as the intervention is scaled-up[40], given their jurisdictional responsibilities for prevention and health care services.

The units of allocation and analysis, i.e., study participants, are primary health care units (PHCUs) and the providers working in them. Within each PHCU, eligible providers include any fully trained health care provider working in the PHCU and involved in medical and/or preventive care. The providers sign an informed consent for their participation. The overall study design is summarized in Figure 1.

Figure 1 here

Figure 1 Study flow diagram

The study timetable is included in the supplement. The data management plan is added as Annexe A.

For the first six months of an 18-month implementation and test period, a four-arm design is adopted, Figure 2. Within each municipal area, PHCUs are systematically invited to join the study, until nine PHCUs agree. Within the comparator municipal area, four PHCUs are randomly allocated to control (Arm 1), and five PHCUs to receive short training to implement a short clinical package (Arm 2). Within the intervention municipal area, in which all PHCU receive municipal action, five PHCUs are randomly allocated to receive short training to implement a short clinical package (Arm 3), and four PHCUs to receive standard training to implement a standard clinical package (Arm 4). Random allocation was undertaken using Excel random number generator.

Figure 2 here

Figure 2. Study design for the first six months of the 18-month implementation period

By Month 6, Hypotheses 3, i.e., non-superiority of Arm 4 (longer package with municipal action and training) over Arm 3 (short package with municipal action and training) will be tested. In the presence of clinical equivalence of a relative difference of the primary outcome, i.e., the cumulative coverage

For peer review only - http://bmjopen.bmj.com/site/about/guidelines.xhtml

of patients whose alcohol consumption is measured, of less than 10%, Arm 4 will be replaced by Arm 3 from month 8 onwards, Figure 3.

Figure 3 here

Figure 3. Study design from month 8 onwards, assuming no superiority of Arm 4 over Arm 3 during first six months of implementation.

The inputs to each of the four arms are summarized in Supplement Tables 1 and 2, and the standard and shorter clinical pathways that are implemented are summarized in Supplement Figures 1 and 2.

Data collection and instruments

Municipal level information

At the level of the municipal area (or, when not available, at whole city, regional or country level), the following information will be collected from routinely available data on socio-demographic factors, alcohol and mental health data, health system structures, quality of life, sustainable governance and values, Supplement Table 3.

PHCU and provider level information

All contacted PHCU, including those who did and did not agree to be part of the study, will provide information on:

- Numbers of registered patients, divided into age 0-17 years and 18+ years; and,
- Numbers and professions of provider staff (including physicians, nurses, nurse technicians, midwifes, psychologists, social workers, and others).

At recruitment, PHC providers will provide data on their:

- Age;
- Gender;
- Profession (doctor, nurse, practice assistant etc.);
- Time worked in the PHC.

Since we are unable to randomize the municipal areas involved, we will use propensity score matching (PSM) based on data collected at the level of the municipal area and the PHCU, to take into account potential confounding variables between comparator and intervention municipal areas, and minimise bias on account of these.

Provider-based measurement and assessment of alcohol consumption and comorbid depression and

For peer review only - http://bmjopen.bmj.com/site/about/guidelines.xhtml

record of advice and treatment given (tally sheets)

Based on the validated methodology of the ODHIN project[22,24], PHC providers will document activity by completing anonymous paper tally sheets that record eligible patients' (aged 18+ years) AUDIT-C scores[57], and, if administered, AUDIT-10[58], PHQ-2[59] and PHQ-9[60] scores, and the advice or treatment given to each patient. The tally sheets will record the age, sex, and educational level of the patient, the latter as a proxy measure of socio-economic status. Data will be collected for the one-month baseline measurement period, and for each calendar month of the 18-month implementation and test period. PHCUs will return data on the number of adult (aged 18+ years) consultations per provider for the one-month baseline measurement period, and for each of the 18 months of the implementation and test period. Monthly data will be collected and reported with accumulation of coverage over time. Formal reporting will be undertaken at baseline, and for coverage achieved by month 12 and by month 18 of the 18-month implementation and test period. Tally sheets will include an identifying code of the provider, PHCU, country and study arm, but no identifying code of the patient. Data will be extracted and sent to the project's data warehouse at Technical University Dresden on a monthly basis.

Extended Tally Sheets

As part of quality control, in all four Arms at two time points, during the 18-month implementation and test period (months 3 and 15), providers will complete extended tally sheets on two separate days in each month. The extended tally sheets will include an identifying code of the provider but no identifying code of the patient. The extended tally sheet will include: additional information from the patient on alcohol knowledge[61], social norms[62] and health literacy[63] applied to alcohol, as it informs the content of advice given; and, additional information from the provider on contextual characteristics that informed their advice giving. The extended tally sheets will include a consent form for the patient and self-completed additional questions for the patient to complete, once the consultation has ended.

Provider-based attitudes and experiences.

At recruitment, and at two time points during the 18-month implementation period (months 3 and 13), providers will provide data on their attitudes and experiences to working with patients with heavy drinking and comorbid depression, Supplement Table 4.

Providers will complete a short questionnaire after each of the training and booster sessions that they attended (before baseline assessment and at months 4 and 8). The questionnaires that are adapted based on specific training contents (standard or shorter package), will assess the participants' experience of the training, measuring satisfaction with the components of the training aspects, as well as their perceived utility. Two measures included in the main provider questionnaires, SAAPPQ[64] and self-efficacy[65], will be included in order to assess the specific impact of the training, independent of the effect of the implementation of the intervention.

The specific content, number and timing of the training-related questionnaires will depend on the study arm: Arm 2 and 3 participants will fill in two questionnaires, one after training and one after the booster session; while Arm 4 participants will fill in four questionnaires, one after each of the two

For peer review only - http://bmjopen.bmj.com/site/about/guidelines.xhtml

training sessions and one after each of the two booster sessions.

Self-completed additional questions by patient

On two separate days, during months 3 and 13, following the consultation with the extended tally sheet, patients who are able to read and write will be invited to give consent to self-complete additional questions in the waiting room before leaving the PHCU, handing the completed questions to a researcher in attendance. No patient identifying information will be included in the patient questionnaires. Six domains, serving as quality control, will be included:

i. AUDIT-C[57];
ii. PHQ-2[59];
iii. Experiences of the consultation;
iv. Views on being asked about alcohol consumption;
v. Health Literacy[63] as it applies to alcohol; and,
vi. Exposure to communication and media campaigns on alcohol.

On each day, 270 patient questionnaires will be collected across all PHCUs, with up to 1080 questionnaires completed in total across the four days.

Key informant interviews

A number of individual or group interviews will be undertaken throughout the project with key stakeholders – providers, user panel members, CAB members, project partners, and any other people involved in the implementation of the SCALA project. Depending on the stakeholder and their involvement in the project, the topics of the interviews will cover topics such as the necessary adaptation to the protocol; the experience of implementing the programme in primary health care practice; and the perception of the municipal support and the community campaigns.

Observations

The training sessions with the primary health care providers, and the meetings of the CABs will be observed by a neutral observer in order to take note of additional possible barriers in the implementation of the protocol that emerge through the training sessions and meetings. Participant responsiveness will also be observed.

Economic data for return-of-investment analyses

Within SCALA, we will conduct return-on-investment (RoI) analyses, by assessing for each EURO invested in scaling up delivery of screening and brief interventions in primary health care in Columbia, Mexico, and Peru, how many EUROs will be saved by reductions in future health care utilization. The return of investment will be defined as the [return on investment = (gain from investment – cost of investment) / cost of investment]. For details on the data required for RoI analyses, Supplement Table 5.

For peer review only - http://bmjopen.bmj.com/site/about/guidelines.xhtml

For the RoI analyses, the effects of increased coverage of alcohol brief advice among primary health care patients will be modelled using effect sizes from previous meta-analyses [64]. To translate the reduced intake of alcohol into health gains, we will calculate alcohol-attributable fractions for major disease and injury categories. These fractions will then be applied to the cost data outlined in Supplement Table 5 to estimate the alcohol-attributable costs per disease category.

Process evaluation

As the intervention is embedded in a complex system involving actions and actors at different levels (individual, organisational, municipal), a thorough process evaluation will be carried out to complement and better understand the outcomes. Through the process evaluation, the implementation with its fidelity and adaptation will be assessed, along with the drivers of scale-up and contextual factors influencing the implementation, the drivers, and the outcomes. This will be achieved in four blocks: driver diagram creation; barriers and facilitators analysis; assessment of implementation, mechanisms of impact and context; and, further contextual and policy analysis.

Driver diagrams

Driver diagrams[65] will be used in order to describe the intervention and its causal assumptions, providing the theory of change through displaying what contributes to intervention aim and what are the relationships between primary drivers, secondary drivers and specific change ideas/activities. The initial general driver diagram, Supplement Figure 3, will be modified based on local contexts and adapted throughout the duration of the project in order to understand how scale up varies in the different cities.

Barriers and facilitators assessment

Factors influencing the implementation of the SCALA protocol will be assessed before the implementation, as well as monitored throughout. The anticipated barriers and facilitators to implementation will be assessed through development of evaluation tool based on literature review[66-68] and implementation framework[69], with subsequent refinement and adaptation to the local context through focus group discussions and workshops with the CABs. The aim of the tool is to identify the barriers that would have to be addressed and monitored throughout implementation and the facilitators that would incentivize and engage providers and the PHCU unit managers in uptake and scaling up of the SCALA protocol. The experienced barriers and facilitators will be further monitored through meeting observations, provider questionnaires and interviews, as well as interviews with other involved stakeholders (e.g. CAB members, PHCU managers).

Implementation, mechanisms of impact and context

The factors influencing the progress from scale-up to outcomes will be identified and documented based on UK Medical Research Council guidance[70], analysing factors within five groups: (i) description of intervention and its causal assumptions; (ii) context; (iii) implementation; (iv) mechanisms of impact; and, (v) outcomes. All aspects of the intervention will be taken into consideration: the intervention, intervention tailoring, training, training tailoring, as well as the municipal action, consisting of the CABs and the communication campaign, combining both quantitative and qualitative

For peer review only - http://bmjopen.bmj.com/site/about/guidelines.xhtml

methods in order to obtain a comprehensive picture of the integration and interaction of included variables. A detailed description of the topics of interest and accompanied methods is presented in Supplement Table 6.

The five groups will be assessed as follows:

i. *Description of the intervention.* The description of the intervention and its causal assumptions draws from the previously described driver diagram;
ii. *Implementation.* Delivery of the training will be assessed though document analysis (reports from training), observation and self-reports from the trainers. Delivery of the intervention will be assessed through document analysis, interviews with patients and providers. The areas of focus will be fidelity, adaptation, dose and reach. Implementation of the CAB meetings and community action will be assessed mainly through document analysis, as well as key informant interviews;
iii. *Mechanisms of impact*. The following three areas will be covered: participant responses to the intervention, mediators and unintended consequences. Mechanisms of impact of intervention delivery will be assessed through patient and providers' questionnaires. The patient interviews will focus on their responsiveness to the intervention, specifically looking at perceived acceptability. In order to evaluate participants' responses to the training, a post-training questionnaire examining satisfaction with the training and perceived utility of training sessions will be applied, triangulated with data from observation and trainers' self-report. Additionally, providers' self-efficacy will be tested as potential mechanism of impact that links the implementation to the outcomes. Mechanisms of impact of the CAB meetings and community action will be examined through key informant interviews and questionnaires. Specific focus will be placed on perceptions and mechanisms of actions of the communication campaign, examining its effect on attitudes and social norms of both providers and patients;
iv. *Context.* Contextual factors that should be considered in order to better understand the success of the intervention will be assessed through meeting observation, document analysis, and provider questionnaires, as well as stakeholder interviews, with the main focus primarily on individual and organisational level characteristics of the context. For the training evaluation, context will be assessed through observation and trainers' self-report. Context of municipal level actions will be assessed through key informant interviews. Additionally, contextual and policy factors on national and municipal levels will be assessed as described below.
v. *Outcome.* The data collected through process evaluation will be combined with the outcomes and presented within the RE-AIM framework[71-73], evaluating SCALA's impact across the dimensions of reach, effectiveness, adoption, implementation and maintenance.

Contextual and policy factors

Based on methodology of Ysa et al[74], contextual and policy factors on national and municipal level will be identified through document analysis and key informant interviews. The main variables considered for contextual analysis will be: (1) available data similar to that of the OECD better life initiative[75]; (2) Sustainable Governance Indicators[76]; and, (3) World Values Survey data[77]]. For policy analysis, the

For peer review only - http://bmjopen.bmj.com/site/about/guidelines.xhtml

information sought will be for a for alcohol policy-related strategies, action plans, legislation and evaluations, both on country and municipal level. The existing contextual and policy factors will be mapped onto the test of the scale-up of the SCALA package to describe and identify those factors on national and municipal level that might influence going to full-scale beyond the tested scalable units.

Outcomes

Primary outcome:

The primary outcome will be the cumulative proportion of the number of adults (aged 18+ years) registered with the PHCU that have their alcohol consumption measured with a completed AUDIT-C instrument during the study period (coverage). The number of adults registered is provided by the administrative office of the PHCU and includes all adult patients covered by the PHCU, whether or not they consult during the 18-month implementation test period.

Secondary outcomes:

- **Proportion of consulting patients who have their alcohol consumption measured by AUDIT-C:** Calculated as the number of adults who have their alcohol consumption measured by AUDIT-C divided by the total number of adults who consult the PHCU during the same time period per participating provider and per PHCU;
- **At risk population receiving advice and/or treatment for heavy drinking:** Calculated as the number of adults with an AUDIT-C score of 8+ who receive brief advice and/or referral for their heavy drinking divided by the total number of patients with an AUDIT-C score of 8+ per participating provider and per PHCU. Information will also be collected on the number of patients with an AUDIT-C score of <8 who receive brief advice and/or treatment for their heavy drinking;
- **Proportion of patients with AUDIT-C score of 8+ who receive assessment for depression:** Calculated as the number of consulting adults with an AUDIT-C score of 8+ who complete PHQ-2 divided by the total number of patients with an AUDIT-C score of 8+ per participating provider and per PHCU;
- **At risk population receiving advice and/or treatment for comorbid depression:** Calculated as the number of adults with a PHQ-2 score of 3+ who receive a patient leaflet and/or referral for their depression divided by the total number of patients with a PHQ-2 score of 3+ per participating provider and per PHCU; and,
- **Provider attitudes:** Attitudes of the participating providers will be measured by the short version of the Alcohol and Alcohol Problems Perception questionnaire, SAAPPQ [64]. The responses will be summed within the two scales of role security and therapeutic commitment. Individual missing values for any of the items in a domain will be assigned the mean value of the remaining items of the domain before summation.

Statistical tests of key hypotheses

Primary study goal: Multilevel regression analyses will be undertaken at 12 months' time of the implementation test period, using cumulative results at months 1-12, and at 18 months' time using

For peer review only - http://bmjopen.bmj.com/site/about/guidelines.xhtml

cumulative results months 1-18. Both analyses will include co-variates of country and results during baseline month, analysed at the levels of the PHCU by study arm, taking into consideration the hierarchical nature of the data. For any PHCU that drops out during the study, outcome values for subsequent measurement points will be set at the last value obtained.

Hypothesis 1

Municipal action leads to more sustainable coverage. We will compare results on primary outcome after 18 months with results after 12 months between Arms 3 and 4 versus Arms 1 and 2 via regression.

Dependent variables:

- For each PHCU, cumulative results of months 1-18 of number of patients whose alcohol consumption is measured with AUDIT-C per 1,000 registered patients; and cumulative results of months 1-12 per 1,000 registered patients.

Random effects:

- Country as random intercept (test for inclusion)

Independent variables:

- Proportion of consulting patients who have their alcohol consumption measured with a completed AUDIT-C instrument during the baseline measurement month
- Conditions:
  - Municipal action (yes vs. no)
  - Training (yes vs. no)

It is postulated that coverage for Arms 3 and 4 will be significantly higher than for Arms 1 and 2.

Hypothesis 2

Training leads to higher coverage than no training. For both months 1-12 and months 1-18, compare cumulative coverage as per primary outcome between Arms 1 and 2 via multilevel regression analyses.

Dependent variable

- Cumulative results months 1-12, and cumulative results months 1-18 of number of patients whose alcohol consumption is measured with AUDIT-C per 1,000 registered patients with
- PHCU

Random effects:

- Country as random intercept (test for inclusion)

Independent variables:

- Conditions:

For peer review only - http://bmjopen.bmj.com/site/about/guidelines.xhtml

- Training (Arm 2 vs. Arm 1)

- Covariates:
  - Proportion of consulting patients who have their alcohol consumption measured with a completed AUDIT-C instrument during the baseline measurement month

It is postulated that coverage for Arm 2 will be significantly higher than for Arm 1.

Hypotheses 3

In the presence of municipal action, the short clinical package and short training do not lead to less coverage than the standard clinical package and standard training. In the presence of clinical equivalence of a relative difference of cumulative coverage of patients screened by less than 10% by month 6, the difference between Arm 3 and Arm 4 will be assessed with regression analyses. If Arm 4 is not superior to Arm 3, both arms will be collapsed into Arm 3 (shorter package) from month 8 onwards.

<u>Dependent variable</u>

- Cumulative results months 1-6 per 1,000 patients

<u>Random effects:</u>

- Country as random intercept (test for inclusion)

<u>Independent variables</u>

- Condition:
  - Length of clinical package (longer = arm 4 vs. shorter = arm 3)
- Covariates:
  - Proportion of consulting patients who have their alcohol consumption measured with a completed AUDIT-C instrument during the baseline measurement month

It is postulated that Arm 4 is not significantly superior to Arm 3.

Sample size calculations for main hypothesis

As the outcome of the primary study goal is predicted to be Arm3 > Arm2 > Arm1, we compared both Arm 2 > Arm 1, and Arm 3 > Arm 2.

Our power calculations are based on the following assumptions: given an average size of a PHCU of approximately 15,000 adults, with an average of 1500 new consultations per month, we expect a cumulative coverage after 12 months of 0.0325 of the registered adult population to have had their alcohol consumption measured in the control condition (Arm 1) (data extrapolated from month 3 and month 9 assessments of control group from ODHIN study[22,24]; Anderson, personal communication). For the short clinical package and short training (Arm 2), we expect this to increase to 0.075 (data extrapolated from month 3 and month 9 assessments of training group from ODHIN study[22,24]; Anderson, personal communication). Although the WHO Phase IV study predicts an additional

For peer review only - http://bmjopen.bmj.com/site/about/guidelines.xhtml

beneficial impact of municipal support[41], precise empirical data is not available – however, we consider an estimate for Arm 3, with municipal support, to be 0.15, a proportion that would need to be achieved to consider municipal support to be worthwhile. To detect the difference between Arm 2 and Arm 1, assuming a design effect of 15 PHCUs (clusters) across the three municipal areas in Arm 2, with 15,000 patients (items), and 12 PHCUs (clusters) in Arm 1, with 15,000 patients (items), with an ICC for PHCUs of 0.03 (data from ODHIN study[22,24]; Anderson, personal communication) we would have 82% power at a significance level of 5%[78]. For the difference between Arm 3 and Arm 2 (15 PHCUs/clusters in each arm), we would have 96.5% power.

ETHICS AND DISSEMINATION

This protocol outlines a quasi-experimental study[1] to test the extent to which embedding PHC-based measurement and brief advice activity within supportive municipal action leads to improved scale-up of an intervention package, with more patients having their alcohol consumption measured, and with heavy drinkers receiving subsequent appropriate advice and treatment. It is not envisaged that there will be any substantial protocol modifications during the course of the study. Any modification to the protocol will be described will be described in all scientific publications.

The Ethics Committee of the Technical University of Dresden gave final ethical approval for the SCALA project on 12 April 2019, EK90032018. All participating primary health care units and participating primary health care providers sign an informed consent form for participation with the country-based research team. Selected patients at two separate time points sign an informed consent form with the country-based research team to provide additional anonymized information following a consultation with a primary health care provider. The consent forms are included within Annexe Data Management Plan. All data collection, processing, and sharing procedures will adhere to national and international laws including the General Data Protection Regulation (EU Regulation 2016/679), as described within the Annexe Data Management Plan.

The study has several features worth mentioning. It:

1. uses a theory-based approach[69,79,80] to tailoring clinical materials and training programmes, creating city-based Community Advisory Boards, and user-based User Panels to ensure that tailoring matches user needs, municipal services[81], and co-production of health[82];
2. sets a higher cut-off score for AUDIT-C (8+) than is commonly used to trigger advice-giving, matching definitions of heavy drinking[83,84], and similar to baseline levels of alcohol consumption in PHC-based trials to reduce heavy drinking[51]. We set the same cut-offs for men and women, based on epidemiological evidence[85], and to minimize unintended consequences of using different cut offs for men and women[86]. We recognize the importance of comorbid depression [87,88] by building in identification, management, and referral mechanisms[89,90];
3. tests for non-superiority of implementing a standard measurement and 5-minute brief advice intervention with six hours of training, compared with implementing a shorter 1-minute brief

For peer review only - http://bmjopen.bmj.com/site/about/guidelines.xhtml

advice intervention with three hours of training, taking into account that brief advice is as effective and cost-effective as more extended advice or treatment in reducing heavy drinking[52,91,92], and the need for very brief clinical and training programmes for time-constrained providers;

4. tests the added value of embedding and implementing PHC activity within municipal-based adoption mechanisms and support systems[40], and communication campaigns over and above training programmes solely directed to primary health care providers;
5. has a longer time frame (18 months) than is traditionally used in implementation studies[93,94], to assess longer term impacts; and,
6. gives considerable emphasis to process evaluation[70], developing logic models to document the fidelity of all implementation strategies, and to identify, the drivers and barriers and facilitators to successful implementation and scale-up, and the political and economic contextual factors that might influence scale-up.

There are some limitations to the study design. A trial with random assignment of municipal areas is not feasible due municipal-based political and technical considerations. As we are unable to randomize the involved municipal areas, we adopt a quasi-experimental design[1], trying to optimize comparator municipal areas for confounding, and by using propensity score matching (PSM). While full comparisons via randomization, and thus establishment of causality, are not possible, together with the qualitative evaluation component of the study, we will be able to clearly identify the mechanisms which were crucial in leading to the outcomes. According to a recent 7-item checklist for classifying quasi-experimental studies for Cochrane reviews[95], our approach is, nevertheless, ranked as a strong design, Supplement Table 7.

Although our focus on embedding PHC activity within supportive municipal actions is hypothesized to increase measurement and brief activity over and above that previously demonstrated, such an approach also brings risks. Municipal and national governments change; and, thus health priorities may change. Although our approach minimizes the need for extra resources (and in some jurisdictions, could be resource saving[19], it is not resource free. Funding constraints could limit future scale-up and sustainability.

We have based our protocol adopted on a model of transdisciplinary research to promote sustainability. Such a model identifies, structures, analyses, and deals with specific problems in a way that grasps the complexity of problems[96]; it takes into account the diversity of real-world and scientific perceptions of problems; and develops knowledge and practices that promote what is generally accepted to be the common good[97]. As such, we include municipalities and health systems as stakeholders to form explicitly orchestrated and managed ecosystems that cross organizational boundaries. Municipal areas and health systems create an engagement platform that provides the necessary environment, including people and resources, for sustainability.

All materials are publicly available on the project website: https://www.scalaproject.eu/. According to the SCALA data management plan, by default, all quantitative datasets generated in the course of the SCALA study will be made openly available through the UK Data Service upon publication of the results

For peer review only - http://bmjopen.bmj.com/site/about/guidelines.xhtml

(http://www.data-archive.ac.uk/). Prior to publication, all data will be formatted to meet UK Data Service requirements.

Ministries of Health at municipal and country levels are represented in the Community Advisory Boards created in each intervention municipality to facilitate scale-up at municipal and country levels, once the implementation strategy is validated. SCALA works closely with the Pan American Health Organization (PAHO), with the principal investigator form Mexico being a Collaborating Centre with PAHO, to facilitate scale-up at Latin American levels, once the implementation strategy is validated.

DECLARATIONS

Ethics approval and consent to participate

The Ethics Committee of the Technical University of Dresden gave final ethical approval for the SCALA project on 12 April 2019, EK90032018. All participating primary health care units and participating primary health care providers sign an informed consent form for participation. Selected patients at two separate time points sign an informed consent form to provide additional anonymized information following a consultation with a primary health care provider.

Consent for publication

No individual person's data will be published in any form.

Availability of data and materials

All materials are publicly available on the project website: https://www.scalaproject.eu/. According to the SCALA data management plan, by default, all quantitative datasets generated in the course of the SCALA study will be made openly available through the UK Data Service upon publication of the results (http://www.data-archive.ac.uk/). Prior to publication, all data will be formatted to meet UK Data Service requirements.

Competing interests

None declared

Patient involvement

Through User Panels created as part of the tailoring process, people and patients have had the opportunity to comment on the materials and information designed for use by patients.

Funding

The research leading to these results or outcomes has received funding from the European Horizon 2020 Programme for research, technological development and demonstration under Grant Agreement no. 778048 – Scale-up of Prevention and Management of Alcohol Use Disorders and Comorbid Depression in Latin America (SCALA). Participant organisations in SCALA can be seen at:

For peer review only - http://bmjopen.bmj.com/site/about/guidelines.xhtml

www.scalaproject.eu. The views expressed here reflect those of the authors only and the European Union is not liable for any use that may be made of the information contained therein. The Funder was not involved in the study design. The funder will not be involved in the collection, analysis, interpretation of data, and preparations of any publication.

Authors' contributions

All authors contributed to the Grant Application, on which this protocol is based and adapted. EJ-L drafted the first version of the paper, and revised the paper based on author's feedback and comments. PA prepared the paper and material for submission and undertook the submission process. All authors commented on drafts of the manuscript and read and approved the final version. PA undertook random allocation generation. APG and JMT assigned PHCU to arms in Colombia; GNR and APdL assigned PHCU to arms in Mexico; MP and IVB assigned PHCU to arms in Peru.

Acknowledgements

Not applicable

peer review only

References

01. Shadish WR, Cook TD, Campbell DT: Experimental and quasi-experimental designs for generalized causal inference. 2nd edition. Houghton Mifflin, Michigan,2002. Reference Source

02. World Health Organization. (2018). Global Status Report on Alcohol and Health 2018. Geneva, Switzerland: WHO Press.

03. Rehm J, Gmel GE, Gmel G et al. The relationship between different dimensions of alcohol use and the burden of disease - an update. Addiction 2017 doi:10.1111/add.13757

04. Odlaug BL, Gual A, DeCourcy J, Perry R, Pike J, Heron L, Rehm J. Alcohol dependence, co-occurring conditions and attributable burden. Alcohol and Alcoholism 2016 51 201-209.

05. Bellos S, Skapinakis P, Rai D et al. Longitudinal association between different levels of alcohol consumption and a new onset of depression and generalized anxiety disorder: Results from an international study in primary care. Psychiatry Research 2016 243 30-34.

06. Boden J. M., Fergusson D. M. Alcohol and depression. Addiction 2011; 106: 906–14.

07. Probst C, Roerecke M, Behrendt S, Rehm J. Socioeconomic differences in alcohol-attributable mortality compared with all-cause mortality: A systematic review and meta-analysis. *Int J Epidemiol* 2014; **43**(4): 1314-27

08. Kontis V, Mathers, CD, Rehm J, Stevens G A, Shield K D, Bonita R, et al. Contribution of six risk factors to achieving the "25×25" NCD mortality reduction target. Lancet 2014 http://dx.doi.org/10.1016/ S0140-6736(14)60616-4.

For peer review only - http://bmjopen.bmj.com/site/about/guidelines.xhtml

09. Kontis V, Mathers CD, Bonita R et al. Regional contributions of six preventable risk factors to achieving the 25 × 25 non-communicable disease mortality reduction target: a modelling study. *Lancet Glob Health* 2015; 3: e746–57

10. United Nations General Assembly. Political declaration of the High-level Meeting of the General Assembly on the Prevention and Control of Non-communicable Diseases. http://www.un.org/en/ga/ncdmeeting2011/ Acessed 01/02/2017

11. GBD 2015 SDG Collaborators. Measuring the health-related Sustainable Development Goals in 188 countries: a baseline analysis from the Global Burden of Disease Study 2015. Lancet 2016 388 1813-50.

12. Shield, K.D., Manthey, J., Rylett, M., Probst, C., Wettlaufer, A., Parry, C.D.H. & Rehm, J. (2020). National, regional, and global burdens of disease from 2000 to 2016 attributable to alcohol use. Lancet Public Health, 5(1), e51–61. doi: 10.1016/S2468-2667(19)30231-

13. Economic Dimensions of Noncommunicable Diseases in Latin America and the Caribbean: Policy Agenda Considerations 143 George Alleyne. In: http://iris.paho.org/xmlui/bitstream/handle/123456789/28501/9789275119051_eng.pdf?sequence=1&isAllowed=y

14. Levin C, Chisholm D. Cost-Effectiveness and Affordability of Interventions, Policies, and Platforms for the Prevention and Treatment of Mental, Neurological, and Substance Use Disorders. In Patel, V., D. Chisholm., T. Dua, R. Laxminarayan, and M. E. Medina-Mora, editors. 2015. *Mental, Neurological, and Substance Use Disorders*. Disease Control Priorities, third edition, volume 4. Washington, DC: World Bank. doi:10.1596/978-1-4648-0426-7.

15. O'Donnell, A., Anderson, P., Newbury-Birch, D., Schulte, B., Schmidt, C., Reimer, J. & Kaner, E. The Impact of Brief Alcohol Interventions in Primary Healthcare: A Systematic Review of Reviews. Alcohol and Alcoholism doi: 10.1093/alcalc/agt170.

16. Angus C, Thomas C, Anderson P, et al. Estimating the cost-effectiveness of brief interventions for heavy drinking in primary health care across Europe. Eur J Public Health. 2017;27:345–351.

17. Rehm J & Barbosa C. (2018) The cost-effectiveness of therapies to treat alcohol use disorders, Expert Review of Pharmacoeconomics & Outcomes Research, 18:1, 43-49, DOI: 10.1080/14737167.2018.1392241

18. Rehm, J., Shield, K. D., Gmel, G., Rehm, M. X., & Frick, U. (2013). Modeling the impact of alcohol dependence on mortality burden and the effect of available treatment interventions in the European Union. European Neuropsychopharmacology, 23(2), 89–97.

19. Organisation for Economic Co-operation and Development. *Tackling Harmful Alcohol Use: Economics and Public Health Policy*. Paris: Organisation for Economic Co-operation and Development, 2015.

20. Anderson, P., M. Laurant, E. Kaner, M. Wensing and R. Grol (2004). "Engaging general practitioners in the management of hazardous and harmful alcohol consumption: results of a meta-analysis." J Stud Alcohol 65(2): 191-199.

For peer review only - http://bmjopen.bmj.com/site/about/guidelines.xhtml

21. Keurhorst M, van de Glind I, Bitarello do Amaral-Sabadini M, Anderson P, Kaner E, Newbury-Birch D, Braspenning J, Wensing M, Heinen M, Laurant M (2015) Determinants of successful implementation of screening and brief interventions for hazardous and harmful alcohol consumption in primary healthcare. A systematic review and meta-regression analysis. Addiction Addiction 110:877–900.

22. Anderson, P., Gual, T., Coulton, S., Kaner, E., Bendsten, P., Kłoda, K., Reynolds, J., Keurhorst., M. N., Segura, Wojnar, M., Mierzecki, A., Deluca, P., Newbury-Birch, D., Parkinson, K., Okulicz- Kozaryn, K., Drummond, C., Laurant, M. (2017) Improving the delivery of brief interventions for heavy drinking in primary health care: nine month outcomes of the ODHIN five country cluster randomized factorial trial. Annals of Family Practice. In Press.

23. Anderson, P., E. Kaner, S. Wutzke, M. Funk, N. Heather, M. Wensing, R. Grol, A. Gual, L. Pas and W. H. O. B. I. S. Group (2004). "Attitudes and managing alcohol problems in general practice: an interaction analysis based on findings from a WHO collaborative study." Alcohol Alcohol 39(4): 351-356.

24. Anderson, P., Bendsten, P., Spak, F., Reynolds, J., Drummond, C., Segura, L., Keurhorst., M. N., Palacio-Vieira, J., Wojnar, M., Parkinson, K., Colom, J., Kłoda, K., Deluca, P., Baena, B., Newbury-Birch, D., Wallace, P., Heinen, M., Wolstenholme, A., van Steenkiste, B., Mierzecki, A., Okulicz-Kozaryn, K., Ronda, G., Kaner, E., Laurant, M., Coulton, S., Gual, T. (2016) 'Improving the delivery of brief interventions for heavy drinking in primary health care: outcome results of the ODHIN five country cluster randomized factorial trial' Addiction DOI: 10.1111/add.13476.

25. Carlos Campillo Serrano, Rosa Díaz Martínez, Martha P. Romero Mendoza, Juan Cerrud Sánchez, Jorge Villatoro (1992). La intervención eficaz del médico general en el tratamiento de bebedores cuyo hábito alcohólico representa un riesgo para su salud o ya les ha ocasionado algún daño. (Preliminary results of a prospective double blind clinical trial). *Salud mental*, 15 (2).

26. Hoffman KA, Beltrán J, Ponce J, García-Fernandez L, Calderón M, Muench J, et al. Barreras para implementar el despistaje, intervenciones breves y referencia al tratamiento por problemas de consumo de alcohol y otras drogas en hospitales que atienden personas que viven con el VIH/SIDA en el Perú. *Rev Peru Med Exp Salud Publica*. 2016;33(3):432-7.

27. Lillian Gelberg, Guillermina Natera Rey, Ronald M. Andersen, Miriam Arroyo, Ietza Bojorquez-Chapela, Melvin W. Rico, Mani Vahidi, Julia Yacenda-Murphy, Lisa Arangua & Martin Serota (2017): Prevalence of Substance Use Among Patients of Community Health Centers in East Los Angeles and Tijuana, *Substance Use & Misuse*, 52:3, 359-372

28. Soto-Brandt, G. et al, (2013). Evidencia de validez en Chile del Alcohol, Smoking and Substance Involvement Screening Test (ASSIST). *Adiccione*s vol.26 (4).

29. Costa PH, Mota DC, Cruvinel E, Paiva FS, Ronzani TM. (2013). A methodology to implement preventive actions against harmful drug use in the context of primary health care in Latin America *Rev Panam Salud Publica*. 33(5):325-31.

30. Natera R et al., 2014: Final Narrative Report of the bi-national assist screening and quit using drugs intervention trial (quit) Tijuana/Los Angeles. Mexico, National Institute on Psychiatry de la Fuente Muniz.

For peer review only - http://bmjopen.bmj.com/site/about/guidelines.xhtml

31. Kaner E. Brief alcohol interventions – time for translational research. Addiction 2010; 105: 960-961.

32. Eccles M, Grimshaw J, Walker A, et al.: Changing the behavior of healthcare professionals: the use of theory in promoting the uptake of research findings. *J Clin Epidemiol.* 2005;58(2):107–112. 15680740 10.1016/j.jclinepi.2004.09.002

33. Norton W, Mittman B: Scaling-up Health Promotion/Disease Prevention Programmes in Community Settings: Barriers, Facilitators, and Initial Recommendations. West Hartford, Connecticut, The Patrick and Catherine Weldon Donaghue Medical Research Foundation.2010. Reference Source

34. Woolf SH, Johnson RE: The break-even point: when medical advances are less important than improving the fidelity with which they are delivered. *Ann Fam Med.* 2005;3(6):545–552. 16338919 10.1370/afm.406 1466946

35. Massoud R: An approach to rapid scale-up using HIV/AIDS treatment and care as an example. Geneva: World Health Organization;2004. Reference Source

36. Cooley L, Kohl R: Scaling up—from vision to large-scale change: a management framework for practitioners. Washington D.C: Management Systems International;2006. Reference Source

37. Damschroder LJ, Aron DC, Keith RE, et al.: Fostering implementation of health services research findings into practice: a consolidated framework for advancing implementation science. *Implement Sci.* 2009;4:50. 19664226 10.1186/1748-5908-4-50 2736161

38. Yamey G: Scaling up global health interventions: a proposed framework for success. *PLoS Med.* 2011;8(6):e1001049. 21738450 10.1371/journal.pmed.1001049 3125181

39. Adamou B: Guide for monitoring scale-up of health practices and interventions: MEASURE evaluation.2013; Accessed 4 November 2016. Reference Source

40. Barker PM, Reid A, Schall MW: A framework for scaling up health interventions: lessons from large-scale improvement initiatives in Africa. *Implement Sci.* 2016;11(1):12. 26821910 10.1186/s13012-016-0374-x 4731989

41. Heather N. editor. WHO Collaborative Project on Identification and Management of Alcohol-related Problems in Primary Health Care – Report to the World Health Organisation on Phase IV: Development of Country-wide Strategies for Implementing Early Identification and Brief Intervention in Primary Health Care. Geneva: World Health Organisation, Department of Mental Health and Substance Abuse: 2006. http://www.who.int/substance_abuse/publications/identification_management_alcoholproblems_phaseiv.pdf

42. Keurhorst M, Heinen M, Colom J et al. Strategies in primary healthcare to implement early identification of risky alcohol consumption: why do they work or not? A qualitative evaluation of the ODHIN study. Keurhorst et al. BMC Family Practice (2016) 17:70 DOI 10.1186/s12875-016-0461-8.

43. Babor T, Del Boca F, Bray JW. Screening, brief intervention and referral to treatment: implications of SAMHSA's SBIRT initiative for substance abuse policy and practice. Addiction 2017 112 (Supple. 2) 110-117.

For peer review only - http://bmjopen.bmj.com/site/about/guidelines.xhtml

44. Vendetti J., Gmyrek A., Damon D., Singh M., McRee B., Del Boca F. Screening, Brief Intervention and Referral to Treatment (SBIRT): implementation barriers, facilitators and model migration. Addiction 2017; 112 (Suppl. 2), 23–33.

45. Singh M., Gmyrek A., Hernandez A., Damon D., Hayashi S. Sustaining Screening, Brief Intervention and Referral to Treatment (SBIRT) services in health-care settings. Addiction 2017; 112 (Suppl. 2), 92–100.

46. Jonas DE, Garbutt JC, Brown JM, Amick HR, Brownley KA, Council CL, et al. Screening, Behavioral Counseling, and Referral in Primary Care to Reduce Alcohol Misuse. Comparative Effectiveness Review No. 64. Rockville, MD: Agency for Healthcare Research and Quality; July 2012. Accessed at www.ncbi .nlm.nih.gov/books/NBK99199/ on 16 April 2016.

47. Rehm J, Anderson P, Manthey J, Shield KD, Struzzo P, Wojnar M, Gual A. (2016). Alcohol use disorders in primary health are: what do we know and where do we go? Alcohol and Alcoholism 51 422-427.

48. Rubinsky, A.D., Dawson, D.A., Williams, E.C., Kivlahan, D.R. & Bradley, K.A. AUDIT-C Scores as a Scaled Marker of Mean Daily Drinking, Alcohol use disorders Severity, and Probability of Alcohol Dependence in a U.S. General Population Sample of Drinkers. Alcoholism: Clinical and Experimental Research 2013 37 1380-1390.

49. National Clinical Guideline Centre (2011). The clinical management of primary hypertension in adults. 2011 https://www.nice.org.uk/guidance/cg127/evidence/cg127-hypertension-full-guideline3 (accessed 1 December 2016).

50. Rehm, J., Anderson, P., Arbesu Prieto, J.A., Armstrong, I., Aubin, H.-J., Bachmann, M., Bastida Bastús N, Brotons, C., Burton, R., Cardoso, M., Colom, J., Duprez, D., Gmel, G., Gual, A., Kraus, L., Kreutz, R., Liira, H., Manthey, J., Møller, L., Okruhlica, L., Roerecke, M., Scafato, E., Schulte, B., Segura-Garcia, L., Shield, K.D., Sierra, C., Vyshinskiy, K., Wojnar, M., & Zarco, J. (2017). Towards new recommendations to reduce the burden of alcohol-induced hypertension in the European Union. BMC Medicine, 15, 173. doi: 10.1186/s12916-017-0934-1.

51. Kaner EF, Beyer FR, Dickinson HO, *et al.*: Effectiveness of brief alcohol interventions in primary care populations. *Cochrane Database Syst Rev.* 2007; (2): CD004148.

52. Platt L, Melendez-Torres GJ, O'Donnell A, et al.: How effective are brief interventions in reducing alcohol consumption: do the setting, practitioner group and content matter? Findings from a systematic review and metaregression analysis. *BMJ Open.* 2016;6(8):e011473. 27515753 10.1136/bmjopen-2016-011473 4985973

53. Kaner, E., Bland, M., Cassidy, P., Coulton, S., Dale, V., Deluca, P., ... & Newbury-Birch, D. (2013). Effectiveness of screening and brief alcohol intervention in primary care (SIPS trial): pragmatic cluster randomised controlled trial. *Bmj*, *346*, e8501. doi: 10.1136/bmj.e8501

54. Bray, J. W., Zarkin, G. A., Hinde, J. M., & Mills, M. J. (2012). Costs of alcohol screening and brief intervention in medical settings: a review of the literature. *Journal of studies on alcohol and drugs*, *73*(6), 911-919. doi: 10.15288/jsad.2012.73.911

For peer review only - http://bmjopen.bmj.com/site/about/guidelines.xhtml

55. Groves, P., Pick, S., Davis, P., Cloudesley, R., Cooke, R., Forsythe, M., & Pilling, S. (2010). Routine alcohol screening and brief interventions in general hospital in-patient wards: acceptability and barriers. *Drugs: education, prevention and policy*, *17*(1), 55-71.doi: 10.3109/09687630802088208

56. Anderson P, O'Donnell A, Kaner E *et al.* Scaling-up primary health care-based prevention and management of alcohol use disorder at the municipal level in middle-income countries in Latin America: Background and pre-protocol for a three-country quasi-experimental study. *F1000Research* 2017, 6:311 (doi: 10.12688/f1000research.11173.1).

57. SAMHSA. AUDIT-C. https://www.integration.samhsa.gov/images/res/tool_auditc.pdf

58. Babor TF, Higgins-Biddle JC, Saunders JB, Monteiro MG AUDIT: The Alcohol Use Disorders Identification Test Guidelines for Use in Primary Care (second edition). https://www.who.int/substance_abuse/publications/audit/en/

59. US Preventive Service task Force. Patient Health Questionnaire PHQ-9. https://www.uspreventiveservicestaskforce.org/Home/GetFileByID/218

60. Center for Quality Assessment and Improvement in Mental Health Patient Health Questionnaire PHQ-2. http://www.cqaimh.org/pdf/tool_phq2.pdf

61.Rosenberg G, Bauld L, Hooper L et al. New national alcohol guidelines in the UK: public awareness, understanding and behavioural intentions. *Journal of Public Health*, 2018 40, 549–556,

62. Chung A, Rimal RN: Social norms: A review. *Review of Communication Research.* 2016;4:1–29. 10.12840/issn.2255-4165.2016.04.01.008

63. Sørensen K, Van den Broucke S, Pelikan JM, et al.: Measuring health literacy in populations: illuminating the design and development process of the European Health Literacy Survey Questionnaire (HLS-EU-Q). *BMC Public Health.* 2013;13:948. 24112855 10.1186/1471-2458-13-948 4016258

64. Anderson P, Clement S: The AAPPQ revisited: the measurement of general practitioners' attitudes to alcohol problems. *Br J Addict.* 1987;82(7):753–9. 3478065 10.1111/j.1360-0443.1987.tb01542.x

64. Kaner EF, Dickinson HO, Beyer FR, Campbell F, Schlesinger C, Heather N, Saunders JB, Burnand B, Pienaar ED. Effectiveness of brief alcohol interventions in primary care populations. *Cochrane Database of Systematic Reviews* 2007, Issue 2. Art. No.: CD004148. DOI: 10.1002/14651858.CD004148.pub3.

65. Svoronos T, Mate KS: Evaluating large-scale health programmes at a district level in resource-limited countries. *Bull World Health Organ.* 2011;89(11):831–837. 22084529 10.2471/BLT.11.088138 3209726

65. Schaufeli WB, Shimazu A, Hakanen J, Salanova M, De Witte H. An Ultra-Short Measure for Work Engagement: The UWES-3 Validation Across Five Countries. European Journal of Psychological Assessment 2017

66. Derges, J., Kidger, J., Fox, F., Campbell, R., Kaner, E., & Hickman, M. (2017). Alcohol screening and brief interventions for adults and young people in health and community-based settings: A

For peer review only - http://bmjopen.bmj.com/site/about/guidelines.xhtml

qualitative systematic literature review. *BMC Public Health*, *17*(1), 1–12. https://doi.org/10.1186/s12889-017-4476-4

67. Johnson, M., Jackson, R., Guillaume, L., Meier, P., & Goyder, E. (2011). Barriers and facilitators to implementing screening and brief intervention for alcohol misuse: A systematic review of qualitative evidence. *Journal of Public Health*, *33*(3), 412–421. https://doi.org/10.1093/pubmed/fdq095

68. O'Donnell, A., Wallace, P., & Kaner, E. (2014). From efficacy to effectiveness and beyond: What next for brief interventions in primary care? *Frontiers in Psychiatry*, *5*(AUG), 1–8. https://doi.org/10.3389/fpsyt.2014.00113

69. Flottorp SA, Oxman AD, Krause J, et al.: A checklist for identifying determinants of practice: a systematic review and synthesis of frameworks and taxonomies of factors that prevent or enable improvements in healthcare professional practice. *Implement Sci.* 2013;8:35. 23522377 10.1186/1748-5908-8-35 3617095

70. Moore GF, Audrey S, Barker M, et al.: Process evaluation of complex interventions: Medical Research Council guidance. *BMJ.* 2015;350:h1258. 25791983 10.1136/bmj.h1258 4366184

71. Glasgow RE, Vogt TM, Boles SM: Evaluating the public health impact of health promotion interventions: the RE-AIM framework. *Am J Public Health.* 1999;89(9):1322–7. 10474547 10.2105/AJPH.89.9.1322 1508772

72. Glasgow RE, Klesges LM, Dzewaltowski DA, et al.: Evaluating the impact of health promotion programs: using the RE-AIM framework to form summary measures for decision making involving complex issues. *Health Educ Res.* 2006;21(5):688–694. 16945984 10.1093/her/cyl081

73. Harden SM, Gaglio B, Shoup JA, et al.: Fidelity to and comparative results across behavioral interventions evaluated through the RE-AIM framework: a systematic review. *Syst Rev.* 2015;4:155. 26547687 10.1186/s13643-015-0141-0 4637141

74. Ysa T, Colom J, Albareda A, et al.: Governance of Addictions. European Public Policies. Oxford University Press,2014. Reference Source

75. OECD: Compendium of OECD well-being indicators. OECD Better Life Initiative.2011; (Accessed 18 December 2016). Reference Source

76. Bertelsmann Stiftung. [website].2016; (Accessed 18 December 2016). Reference Source

77. Inglehart R, Welzel C: Modernization, Cultural Change and Democracy. New York: Cambridge University Press,2005. Reference Source

78. PASS[16] sample size software. https://www.ncss.com/software/pass/: Donner, A. and Klar, N. 2000. Design and Analysis of Cluster Randomization Trials in Health Research. Arnold. London.

79. Wensing M, Oxman A, Baker R, et al.: Tailored Implementation For Chronic Diseases (TICD): a project protocol. *Implement Sci.* 2011;6:103. 21899753 10.1186/1748-5908-6-103 3179734

80. Wensing M, Huntink E, van Lieshout J, et al.: Tailored Implementation of Evidence-Based Practice for Patients with Chronic Diseases. *PLoS One.* 2014;9(7):e101981. 25003371 10.1371/journal.pone.0101981 4087017

For peer review only - http://bmjopen.bmj.com/site/about/guidelines.xhtml

81. Dietz WH, Solomon LS, Pronk N, et al.: An Integrated Framework For The Prevention And Treatment Of Obesity And Its Related Chronic Diseases. *Health Aff (Millwood).* 2015;34(9):1456–1463. 26355046 10.1377/hlthaff.2015.0371

82. Palumbo R: Contextualizing co-production of health care: a systematic literature review. *Int J Public Se Manage.* 2016;29(1):72–90. 10.1108/IJPSM-07-2015-0125

83. Rehm J, Room R, Monteiro M, et al.: Alcohol Use. In: *Comparative quantification of health risks: global and regional burden of disease attributable to selected major risk factors*. Ezzati M *et al.* (eds), Geneva, Switzerland: World Health Organization.2004;959–1109. Reference Source

84. European Medicines Agency: Guideline on the development of medicinal products for the treatment of alcohol dependence.2010. Reference Source

85. Rehm J, Lachenmeier DW, Room R: Why does society accept a higher risk for alcohol than for other voluntary or involuntary risks? *BMC Med.* 2014;12:189. 25424648 10.1186/s12916-014-0189-z 4203927

86. Cunningham JA: Unintended impact of using different inclusion cut-offs for males and females in intervention trials for hazardous drinking. *Addiction.* 2017. 28168847 10.1111/add.13760

87. Wilson GB, Wray C, McGovern R, et al.: Intervention to reduce excessive alcohol consumption and improve comorbidity outcomes in hypertensive or depressed primary care patients: two parallel cluster randomized feasibility trials. *Trials.* 2014;15:235. 24947447 10.1186/1745-6215-15-235 4076249

88. Lai HM, Cleary M, Sitharthan T, et al.: Prevalence of comorbid substance use, anxiety and mood disorders in epidemiological surveys, 1990–2014: A systematic review and meta-analysis. *Drug Alcohol Depend.* 2015;154:1–13. 26072219 10.1016/j.drugalcdep.2015.05.031

89. Linde K, Sigterman K, Kriston L, et al.: Effectiveness of psychological treatments for depressive disorders in primary care: systematic review and meta-analysis. *Ann Fam Med.* 2015;13(1):56–68. 25583894 10.1370/afm.1719 4291267

90. Gilbody S, Whitty P, Grimshaw J, et al.: Educational and organizational interventions to improve the management of depression in primary care: a systematic review. *JAMA.* 2003;289(23):3145–3151. 12813120 10.1001/jama.289.23.3145

91. Aldridge A, Dowd W, Bray J: The relative impact of brief treatment versus brief intervention in primary health-care screening programs for substance use disorders. *Addiction.* 2017;112(Suppl 2):54–64. 28074568 10.1111/add.13653

92. Barbosa C, Cowell A, Dowd W, et al.: The cost-effectiveness of brief intervention versus brief treatment of screening, brief intervention, and referral to treatment (SBIRT) in the United States. *Addiction.* 2017;112(Suppl 2):73–81. 28074567 10.1111/add.13658

93. Anderson P, Kaner E, Keurhorst M, et al.: Attitudes and Learning through Practice Are Key to Delivering Brief Interventions for Heavy Drinking in Primary Health Care: Analyses from the ODHIN Five Country Cluster Randomized Factorial Trial. *Int J Environ Res Public Health.* 2017;14(2): pii: E121. 28134783 10.3390/ijerph14020121 5334675

For peer review only

For peer review only - http://bmjopen.bmj.com/site/about/guidelines.xhtml

94. Funk M, Wutzke S, Kaner E, et al.: A multicountry comparator led trial of strategies to promote dissemination and implementation of brief alcohol intervention in primary health care: findings of a World Health Organization collaborative study. *J Stud Alcohol.* 2005;66(3):379–388. 16047527 10.15288/jsa.2005.66.379

95. Reeves BC, Wells GA, Waddington H: Quasi-experimental study designs series-paper 5: classifying studies evaluating effects of health interventions-a taxonomy without labels. *J Clin Epidemiol.* 2017; pii: S0895-4356(17)30288-3. 28351692 10.1016/j.jclinepi.2017.02.016

96. Jantsch E: Inter- and Transdisciplinary University: A systems approach to education and innovation. *Policy Sciences.* 1970;1(1):403–428. 10.1007/BF00145222

97. Pohl C, Hirsch Hadorn G: Principles for Designing Transdisciplinary Research. proposed by the Swiss Academies of Arts and Sciences. München: oekom Verlag.2007. Reference Source

For peer review only

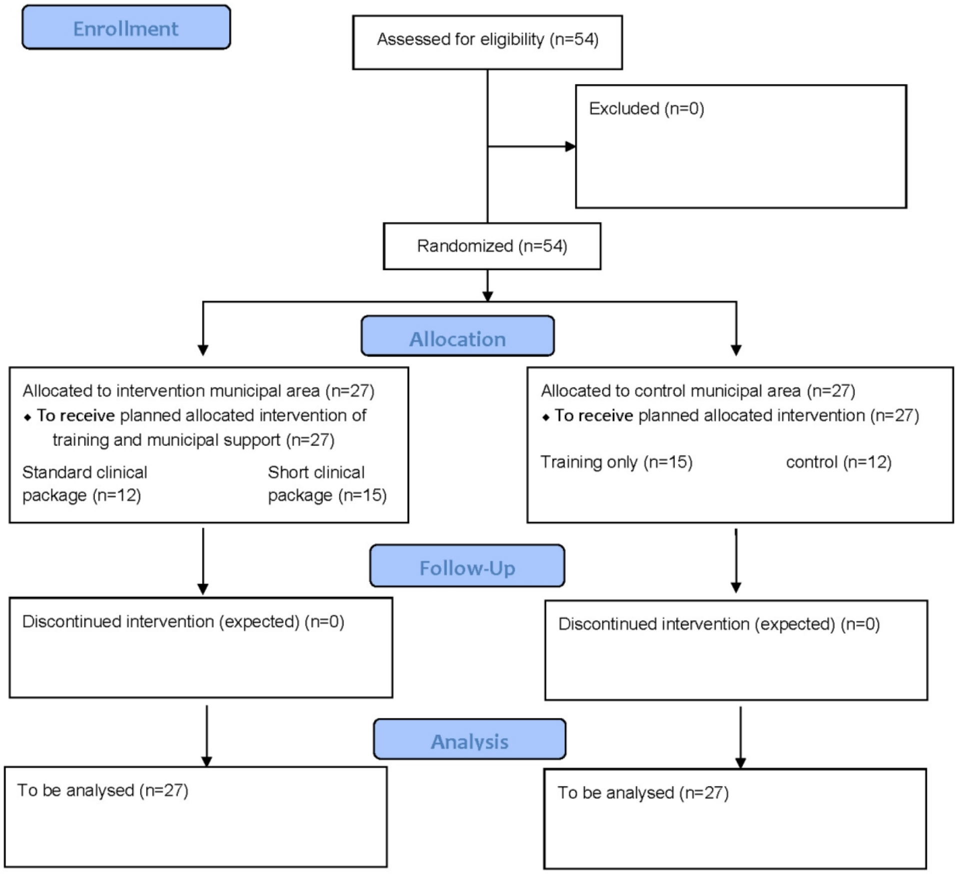

Figure 1 Study flow diagram

206x201mm (200 x 200 DPI)

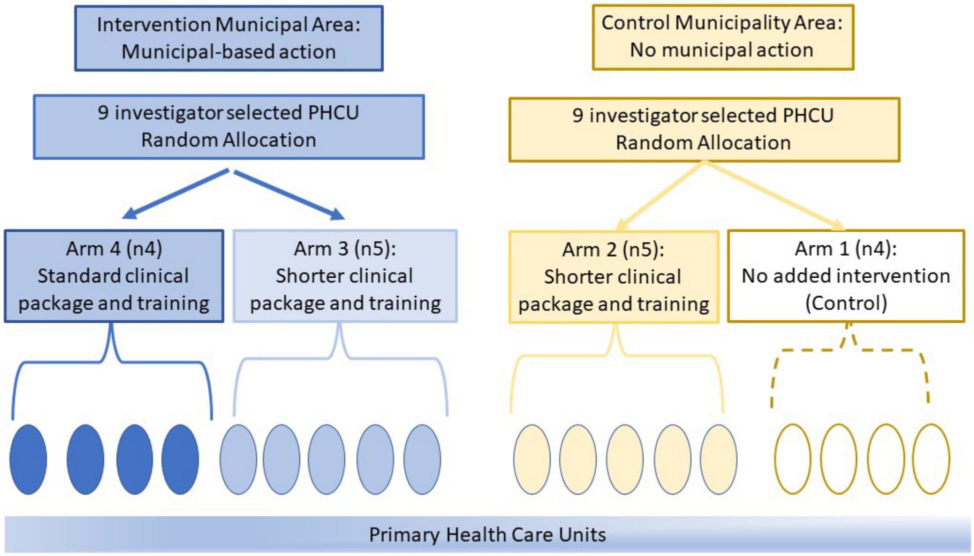

Figure 2. Study design for the first six months of the 18-month implementation period

282x166mm (96 x 96 DPI)

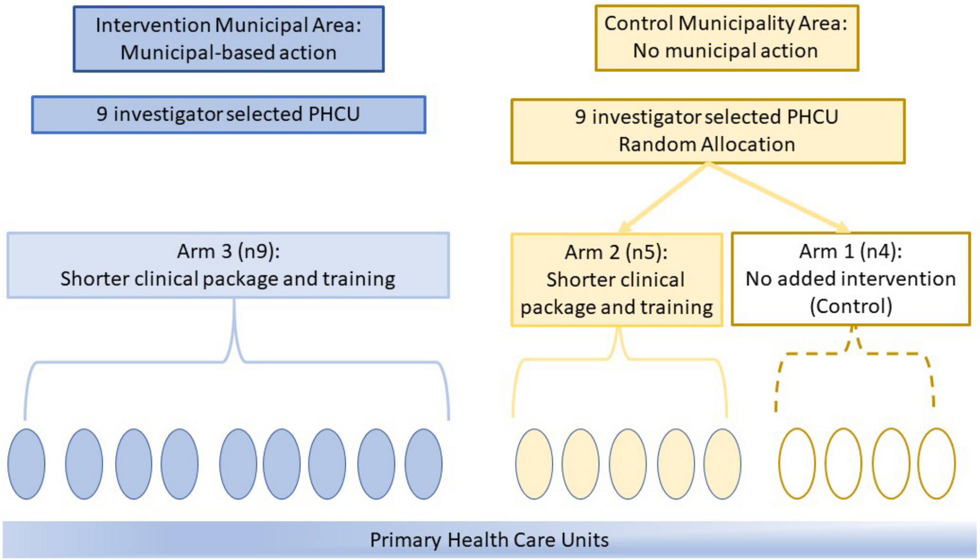

Figure 3. Study design from month 8 onwards, assuming no superiority of Arm 4 over Arm 3 during first six months of implementation.

279x162mm (96 x 96 DPI)

Supplement Box 1 Deviations from pre-grant submission pre-protocol

Moving from two-arm to four-arm design In the pre-submission pre-protocol for the quasi-experimental study [1], within each country, two municipal jurisdictions were to be investigator-selected, each with nine primary health care units (PHCU) as part of the study. In one municipal jurisdiction, the intervention municipality, the PHCU would receive both training and municipal support; in the other municipal jurisdiction, the comparator municipality, PHCU would continue practice as usual, with no training or municipal support. The hypothesis was that PHCU in the intervention municipality would measure the alcohol consumption of more patients and give advice to more heavy drinking patients than the PHCU in the comparator municipality.

In the final protocol, the nine PHCU in the comparator municipality are randomly allocated to five PHCU receiving training (new Arm 2) and four PHCU continuing practice as usual (new Arm 1). The rationale for this approach is that it will enable us to test the independent impact of municipal support over and above just training. The hypothesis to be tested is that PHCU that receive both training and municipal support in the intervention municipality will measure the alcohol consumption of more patients and give advice to more heavy drinking patients than the PHCU who just receive training (Arm 2).

In addition, in the final protocol, the nine PHCU in the intervention municipality are randomly allocated to four PHCU receiving a standard and longer clinical package and training (new Arm 4) and five PHCU receiving a shorter clinical package and training (new Arm 3), both new Arms 3 and 4 receiving municipal support. The hypothesis to be tested is that the PHCU that receive the standard and longer clinical package and training that is commonly implemented (new Arm 4) will not measure the alcohol consumption of more patients and not give advice to more heavy drinking patients than the PHCU that receive a shorter clinical package and training (new Arm 3). This will be tested over the first six months of the 18-month implementation period, and, if there is non-superiority of Arm 4 over Arm 3, Arm 4 will be collapsed into Arm 3 from month 8 onwards.

Cross-sectional patient self-complete questionnaire instead of prospective interview The deviation is to move from patient follow-up interviews to cross-sectional patient self-completed questionnaires. In the pre-submission pre-protocol, during month 3 of the 18-month implementation period, the first six consecutive screen-negative patients and the first six consecutive screen-positive patients identified by each PHCU were to be invited by the health care provider to give their written consent to complete two follow-up questionnaires, at six months and twelve months after the initial screening. In the final protocol, at two time points, during the 18-month implementation period (months 3 and 15), on two separate days in each of month 3 and 15, providers will seek consent from the patient to self-complete additional questions in the waiting room before leaving the PHCU, handing the completed questions to a researcher in attendance. The rationale for the change is that, primarily due to the nature of the catchments area of patients, it became apparent that it would be impossible to achieve sufficient follow-up rates required for valid analysis of data, with much too high a proportion of country-based resources used in order to try to achieve adequate follow-up rates.

Adjustment in primary outcome indicator The deviation is to change the denominator for the main outcome variable from number of consulting adult patients in a given time period (e.g., one month) to number of registered adult patients. In the pre-submission pre-protocol, the primary outcome was to be the proportion of consulting adult patients (aged 18+ years) intervened (alcohol consumption measured and advice given to heavy drinkers), calculated as the number of AUDIT-C positive patients that received oral advice or referral for advice to another provider in or outside the PHCU, divided by the total number of adult consultations of the participating providers per PHCU. In the final protocol, the primary outcome will be the cumulative proportion of the number of adults (aged 18+ years) registered with the PHCU that have their alcohol consumption measured with AUDIT-C. The rationale is that the revised primary outcome is a measure of coverage, which is considered more intuitive and relevant for health systems change (similar to blood pressure - the proportion of patients that have had their blood pressure measured).

Recalculation of statistical power The change in the main outcome measure required a re-calculation of the statistical power. The study remains adequately powered.

For peer review only - http://bmjopen.bmj.com/site/about/guidelines.xhtml

Supplement Table 1 Clinical Package and Training by Study Arm

	**Standard package and training (Arm 4)**	**Shorter package and training (Arms 2 and 3)**	**Control (Arm 1)**
**Instruments**	Short tally sheet: AUDIT-C [2] completed; if AUDIT-C ≥8, AUDIT-10 [3] and PHQ2 [4] completed; if PHQ2 ≥3, PHQ9 [5] completed.	Very short tally sheet: AUDIT-C completed; if AUDIT-C ≥8, PHQ2 completed.	Very short tally sheet: AUDIT-C completed; if AUDIT-C ≥8, PHQ2 completed.
**Provider material**	Provider booklet on alcohol and depression: 43 pages plus 12-page 'quick guide'.	Provider booklet on alcohol and depression: 16 pages.	Provider booklet on alcohol and depression: 11 pages.
**Patient advice and material for alcohol**	Alcohol advice: 5-minute 10-step plan plus 10-page patient brief advice booklet.	Alcohol advice: 1-minute simple advice that the patient needs to drink less, plus 1-page patient brief advice leaflet.	Alcohol advice: 1-minute simple advice that the patient needs to drink less and provide a brief advice leaflet (if available).
	Patient alcohol leaflet: 1 page folded in half to give 4 sides.	Patient alcohol leaflet: 1 page folded in half to give 4 sides.	SCALA patient leaflet on alcohol not given. Provider booklet advises "If available, provide a leaflet on self-management of heavy drinking."
**Patient advice and material for depression**	PHQ9 score 10-14, provide patient leaflet on depression; PHQ 9 ≥14, use clinical judgement to consider if referral is required - if not provide patient leaflet on depression.	PHQ2 ≥3, patient leaflet on depression given.	SCALA patient leaflet on depression not given. Provider booklet advises "If available, provide a leaflet on self-management of depression and action to take if symptoms persist or worsen."
	Patient depression advice leaflet: 1 page, 3 columns.	Patient depression advice leaflet: 1 page, 3 columns.	Present practice.
**Referral**	Referral for very heavy drinking, depression, suicide risk: existing clinical judgement and practice.	Referral for very heavy drinking, depression, suicide risk: existing clinical judgement and practice.	Referral for very heavy drinking, depression, suicide risk: existing clinical judgement and practice.
**Training**	Training: two times two-hours training plus two times one-hour booster sessions (six hours total).<br>Training will take place within	Training: one two-hours training in PHCU, plus one-hour booster session (three hours total).<br>Training will focus on	Present practice.

For peer review only

	the PHCU or clusters of PHCUs. Training will focus on practical skills in undertaking measurement and assessment, and in delivering brief advice, in using the questionnaires, and in knowing when and how to refer patients with more severe heavy drinking and moderately severe or severe depression to available services, such as community-based mental health and addiction centres. Training will, in addition, address attitudes, and perceived barriers and facilitators in implementing measurement and brief advice, contextualized to local circumstances.	practical skills in undertaking measurement and assessment, and in delivering brief advice for harmful alcohol use; instruction of 'care-as-usual' + leaflet for depression and severe cases requiring referral. Training will, in addition, address attitudes, and perceived barriers and facilitators in implementing measurement and brief advice, contextualized to local circumstances.	
	Training for both the standard and shorter packages will be undertaken by members of the research team, accredited teachers, or addiction consultants, who will receive a full two-day train-the-trainers session from a senior addiction specialist trainer. The training formats employed are didactic input, guided discussions, skills and practice modeled through videos and role plays. Training sessions are developed from [6-7].		

For peer review only - http://bmjopen.bmj.com/site/about/guidelines.xhtml

Supplement Table 2 Municipal Integration and Support by Study Arm

**Intervention Municipal Area (Arms 3 and 4)**	**Comparator Municipal Area (Arms 1 and 2)**
Community Advisory Board (CAB) of local stakeholders set up (including representatives of municipal area, PHCU, health services, non-governmental organizations, academia, media).	Present practice.
User Panel (UP) of local providers and patients set up.	Present practice.
CAB and UP review and tailor relevant materials of clinical package and training courses within the seven domains of: local and national guideline factors; individual health care provider factors; patient factors; interactions between different professional groups; incentives and resources; capacity for organizational change; and, social, political and legal factors [8-10].	Present practice.
CAB reviews barriers and facilitators and potential drivers of successful action [11-12].	Present practice.
CAB identifies potential adoption mechanisms and support systems [13], and reviews plans and components of community-based communication and media campaigns [14-16].	Present practice.
Integrator (champion and knowledge and practice broker) to serve as trusted and accountable leader [13]: facilitating agreement within the municipal area and health systems on shared goals and metrics; assessing and acting on relevant community resources; working at the systems level to make relevant practice changes for sustainability; gathering, analysing, monitoring, integrating, learning, and sharing data at the individual PHCU and city levels; identifying and connecting with system navigators who help PHCUs coordinate, access, and manage multiple services and supports; and developing a system of ongoing and intentional communication with PHCUs and cities.	Present practice.
Adoption mechanisms implemented [13], including: (i) demonstration of the superiority of the PHC package, its simplicity, and its alignment with the latest evidence of preventing and managing heavy drinking and of implementation science; (ii) engagement of identified leaders and building their capacity to lead and ensure broad adoption of the PHC package through guiding and supporting large-scale change; (iii) communicating the value of the PHC package to both municipal and PHC frontline staff; (iv) identifying and adjusting, as appropriate and possible, relevant policies at PHC and city levels to expedite the adoption of the PHC package, for example by adapting electronic health records; and, (v) identifying gaps in health system performance and the urgent need to prevent and manage heavy drinking to promote the needed will and energy to bring implementation of the PHC package to scale.	Present practice.
Support mechanisms implemented [13], including: (i) development of professional capacity for scale-up; (ii) development of infrastructure for scale-up, achieved through redesign rather than addition of new resources; (iii) linking to monitoring and evaluation, using reliable data collection and reporting systems that track and provide feedback on the performance of key processes and outcomes, for example monthly reporting on measurement and brief advice activity; (iv) setting up learning systems to capture change ideas that are shown to result in improved performance assembling ideas into a change package. Knowledge should be shared between municipal actors and PHCUs through regular electronic newsletters and communications; and, (v) creating design factors that enhance sustainability including high reliability of the new processes, inspection systems	Present practice.

For peer review only - http://bmjopen.bmj.com/site/about/guidelines.xhtml

to ensure desired results are being achieved, support for structural elements, and ongoing learning systems.	
Communication and media campaign implemented [14-16], including (i) posters, leaflets and/or brochures placed at visible spots in the intervention municipality, e.g., in waiting rooms of PHCUs, health departments, banks, markets; (ii) regular communications, including emails and WhatsApp messages) sent to the healthcare providers and other involved stakeholders in the intervention municipality, (iii) media presence through e.g. articles in local newspapers; interviews, reportages, promotion spots and/or media appearances on local radio, local TV and other local media, and (iv) workshops, forums and/or public local meetings for interested stakeholders such as healthcare providers, representatives of municipal health institutions and patients. All abovementioned activities will focus on reframing that it is heavy drinking that is the problem and that this can be helped to be reduced through primary health care-based measurement and advice programmes, addressing topics such as the harm of hazardous alcohol use in the general population, the (cost)effectiveness and importance of brief alcohol interventions and SCALA success stories.	Present practice.

For peer review only - http://bmjopen.bmj.com/site/about/guidelines.xhtml

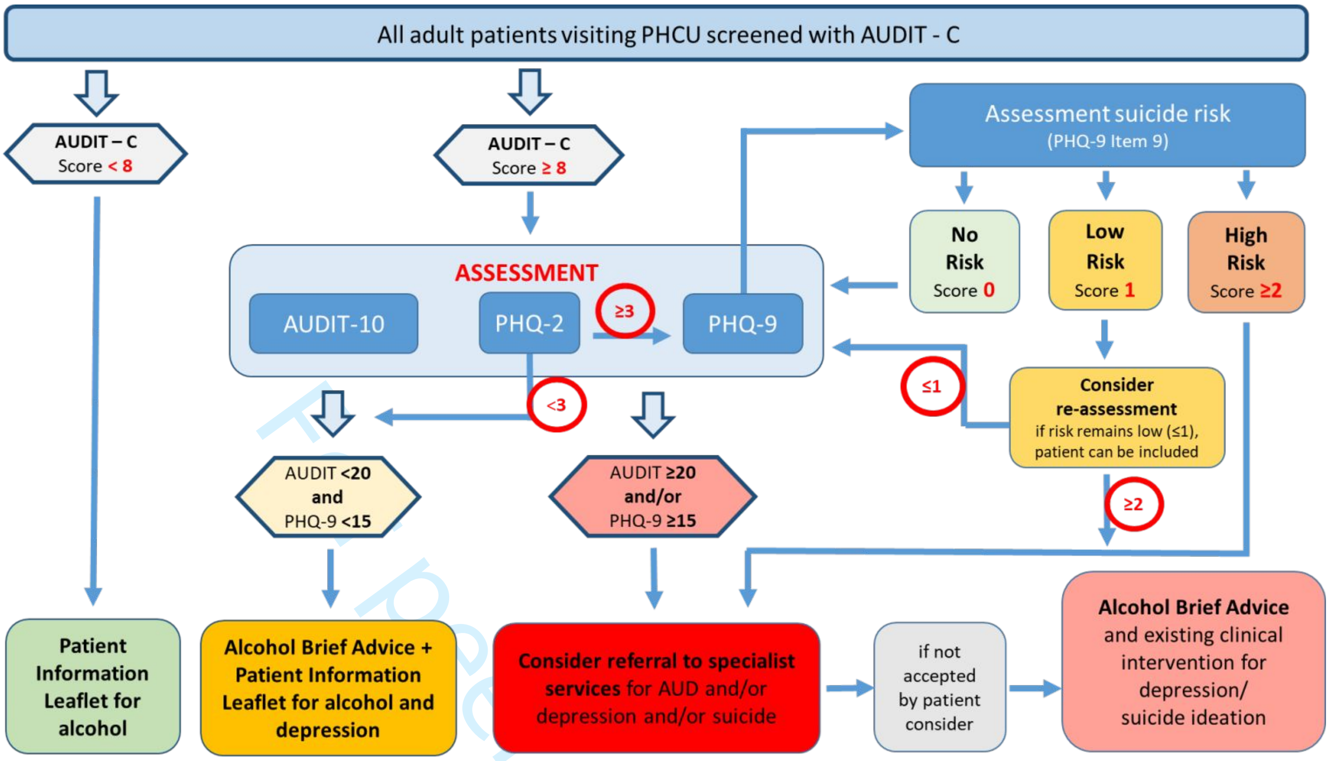

Supplement Figure 1. Standard Care Pathway for Arm 4

For peer review only - http://bmjopen.bmj.com/site/about/guidelines.xhtml

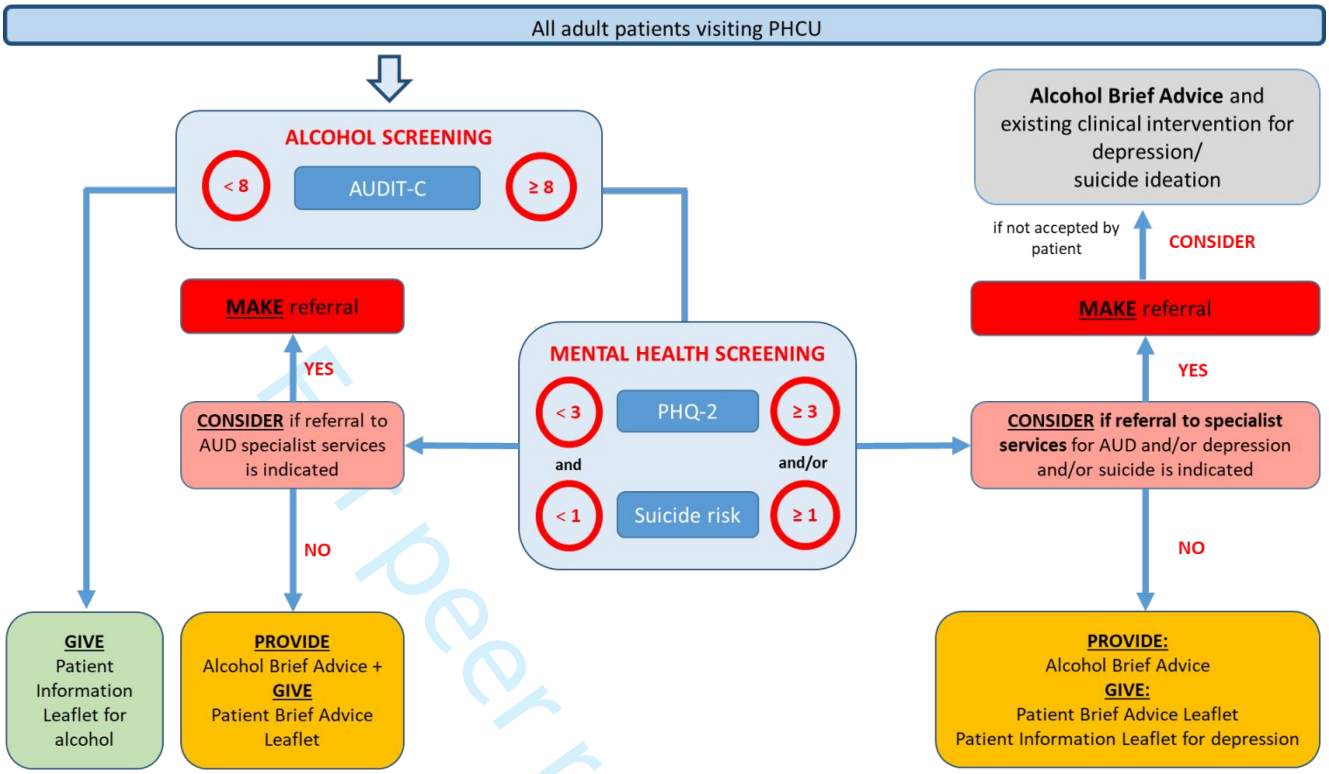

Supplement Figure 2. Short Care Pathway for Arms 1, 2, and 3

For peer review only - http://bmjopen.bmj.com/site/about/guidelines.xhtml

Supplement Table 3 Data collected at municipal level (if not available, at city, regional or country level)

-	Geographical location in city;
-	Demographic size of municipal area;
-	Indicators of deprivation;
-	Information on prevalence of alcohol consumption and related harm;
-	Information on prevalence of depression;
-	Description of current action to reduce alcohol-related harm;
-	Jurisdictional responsibilities for health-related prevention and treatment;
-	Structural relationships with primary health care services;
-	Structural relationships with hospital-based services;
-	Available data mapped to OECD better life initiative [17], including material living conditions (housing, income and jobs) and quality of life (community, education, environment, governance, health, life satisfaction, safety and work-life balance);
-	Sustainable Governance Indicators [18], including the Status Index, which 'examines each state's reform needs in terms of the quality of democracy and performance in key policy fields', and the Management Index, focused on 'governance capacities in terms of steering capability and accountability'; and,
-	World Values Survey data [19] for cross-cultural variation (Traditional vs. Secular-rational; and, Survival vs. Self-expression).

For peer review only

For peer review only - http://bmjopen.bmj.com/site/about/guidelines.xhtml

Supplement Table 4 Overview of the measures used in the provider questionnaire

Measure used	Constructs measured
Shortened Alcohol and Alcohol Problems Perception questionnaire [20]	Role security, therapeutic commitment
Abbreviated Maslach Burnout Inventory [21]	Emotional exhaustion, depersonalization, personal accomplishment
Utrecht Work Engagement Scale [22]	Work engagement
Alcohol knowledge [23]	Awareness of drinking guidelines, social norms regarding drinking
Perceived barriers questionnaire [24]	Perceived barriers
Opinion on screening (based on [25])	Pros and cons of screening, social norms of screening, intention to screen
Self-efficacy in delivering the SCALA protocol (based on [26])	Self-efficacy
Context assessment for community health (COACH) tool [27]	Resources, Community engagement, Monitoring services for action, Work culture, Leadership
Evaluation of SCALA community action [15]	Exposure to campaign/adoption mechanisms/support systems, perceptions of campaign/adoption mechanisms/support systems
Attributes of innovation questionnaire [28]<br>- Only intervention group	Relative advantage, Compatibility, Complexity, Trialability and Observability
Experienced barriers (based on the driver diagram [12])<br>- Only intervention group	Experienced barriers

For peer review only - http://bmjopen.bmj.com/site/about/guidelines.xhtml

Supplement Table 5. Country-level collection of economic data for return-of-investment analyses

Costs of Investment		Gains of investment	
Cost unit	***Data source***	***Cost unit***	***Data Source***
Cost of providing training and booster sessions to PHCU staff	Time and materials required, documented by study team	Costs and utilization of *primary health care* (number of visits) by major disease/injury categories	National statistics, ministry of health, local researchers, or other publications
Setting up and maintaining Community Advisory Boards and User Panels	Time and materials required, documented by study team	Costs and utilization of *emergency* facilities (number of admissions) by major disease/injury categories	National statistics, ministry of health, local researchers, or other publications
Direct costs for implementing the clinical pathway (routine measurement, further assessment, brief interventions, referral)	Staff salary and time required, documented by PHCU administration and providers	Costs and utilization of *inpatient* facilities (number of admissions, length of stay) and of *outpatient* facilities (number of admissions) by major disease/injury categories	National statistics, ministry of health, local researchers, or other publications
Additional costs for implementing the clinical pathway	Documented by PHCU administration	Avoided mortality	National statistics, ministry of health, local researchers, or other publications

For peer review only

For peer review only - http://bmjopen.bmj.com/site/about/guidelines.xhtml

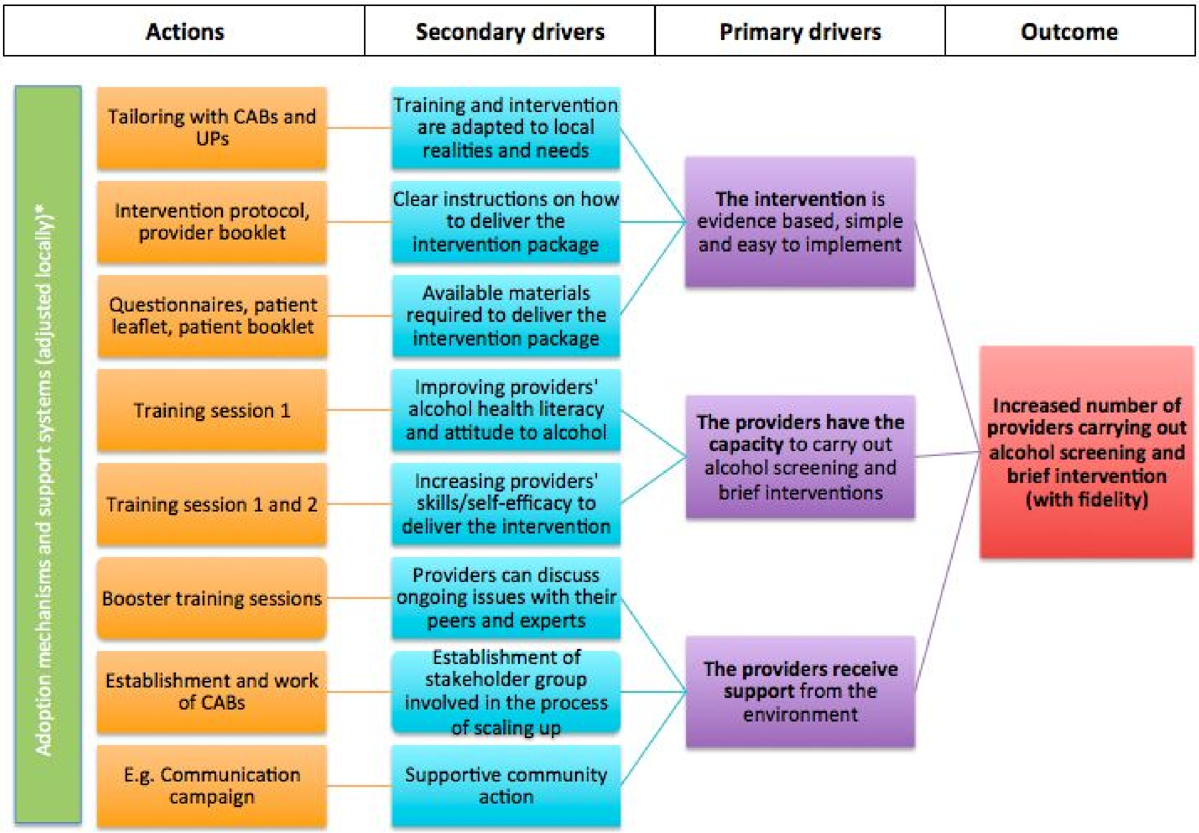

Supplement Figure 3. Driver diagram of the SCALA protocol

review only

For peer review only - http://bmjopen.bmj.com/site/about/guidelines.xhtml

Supplement Table 6 Process evaluation topics based on MRC framework [29]

Part of process evaluation		Topic of investigation	Method
**Implementation**	*Adaptation*	Experience of intervention tailoring	Key informant interview
		Experience with training tailoring	Key informant interview
	Dose delivered (completeness of delivery)	Implementation of the protocol (number of measurements, brief advice given, referrals done)	Tally sheets
		Length of implemented training	Observation
		Implementation of adoption mechanisms and support systems on municipal and organisational level	Key informant interview, Document analysis
		Implementation of CAB meetings	Observation, document analysis
		Implementation of communication campaign	Key informant interview, document analysis
	Fidelity (quality of implementation)	Following the care pathway as intended	Tally sheets, patient questionnaire
		Training active ingredient delivery	Observation
	Reach	Number of patients and providers involved	Document analysis
		Number of providers attending the training	Document analysis
**Mechanisms of impact**	*Participant responses*	Patients' perception of acceptability of intervention	Patient questionnaire
		Providers' satisfaction with the training	Post-training questionnaire
		Providers' perceived utility of training sessions	Post-training questionnaire
		Perception of the intervention	Key informant interview
		Perception of the campaign	Provider questionnaire, patient questionnaire
		Perception of the municipal action	Key stakeholder interview
	Mediators	Influence of training on attitude and self-efficacy	Provider questionnaire
		Influence of communication campaign on beliefs and social norms	Provider questionnaire
		Perception of the attributes of the intervention	Provider questionnaire
	Unintended consequences	Possible unexpected side effects emerging	Key stakeholder interview
**Context**		Perceptions of organisational context	Provider questionnaire
		Individual moderating characteristics	Provider questionnaire
		Description of organisational context changes	Key informant interview, logbook
		Contextual factors influencing training	Observation, key informant interview
		Contextual factors influencing municipal action	Key informant interview, document analysis

For peer review only

For peer review only - http://bmjopen.bmj.com/site/about/guidelines.xhtml

Supplement Table 7 Completed seven-point checklist for SCALA study design [30]

**Quality Measure**	**SCALA**
1.Was the intervention/(answer "yes" to more than 1 item, if applicable)	
Allocated to (provided for / administered to / chosen by) individuals?	No
Allocated to (provided for / administered to / chosen by) clusters of individuals?	No
Clustered in the way it was provided (by practitioner or organisational unit)?	YES
**2. Were outcome data available:** ***(answer "yes" to only 1 item)***	
After intervention / comparator only (same individuals)?	-
After intervention / comparator only (not all same individuals)?	-
Before (once) AND after intervention / comparator (same individuals)?	YES
Before (once) AND after intervention / comparator (not all same individuals)?	-
Multiple times before AND multiple times after intervention / comparator(same individuals)?	-
Multiple times before AND multiple times after intervention / comparator (not all same individuals)?	-
**3. Was the intervention effect estimated by:** ***(answer "yes" to only 1 item)***	
CHANGE OVER TIME (same individuals at different time points)?	-
CHANGE OVER TIME (not all same individuals at different time points)?	-
DIFFERENCE BETWEEN GROUPS (of individuals or clusters receiving either intervention or comparator)?	YES
**4. Did the researchers aim to control for confounding (design or analysis)** ***(answer "yes" to only 1 item)*****:**	
Using methods that control in principle for any confounding?	-
Using methods that control in principle for time invariant unobserved confounding?	-
Using methods that control only for confounding by observed covariates?	YES
**5. Were groups of individuals or clusters formed by** ***(answer "yes" to more than 1 item, if applicable)*****:**	
· Randomization?	No
· Quasi-randomization?<br>· Explicit rule for allocation based on a threshold for a variable measured on a continuous or ordinal scale or boundary (in conjunction with identifying the variable dimension, below)?	No
· Some other action of researchers?	YES
· Time differences?	No
· Location differences?	YES
· Healthcare decision makers / practitioners?	No
· Participants' preferences?	No
· Policy maker	No
· On the basis of outcome?	No
· Some other process? (specify)	No
**6. Were the following features of the study carried out after the study was designed** ***(answer "yes" item, if applicable): to more than 1***	
Characterization of individuals / clusters before intervention?	YES
Actions/choices leading to an individual/cluster becoming a member of a group?	YES
Assessment of outcomes?	YES

For peer review only - http://bmjopen.bmj.com/site/about/guidelines.xhtml

**7. Were the following variables measured before intervention:** ***(answer "yes" to more than 1 item,*** **If applicable)**	
Potential confounders?	YES
Outcome variable(s)?	YES

For peer review only

For peer review only - http://bmjopen.bmj.com/site/about/guidelines.xhtml

SCALA Timetable

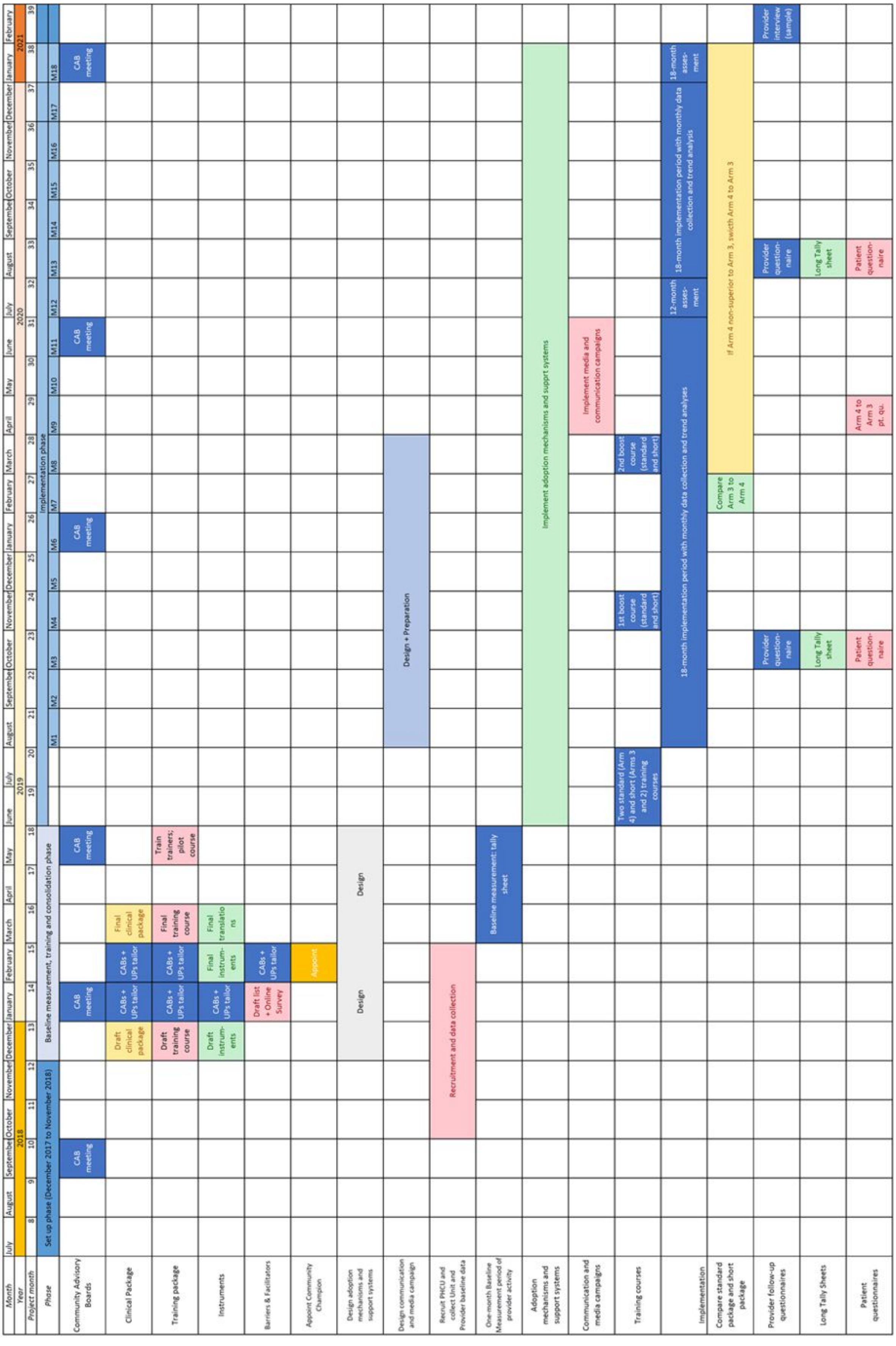

Supplement References

1. Shadish WR, Cook TD, Campbell DT: Experimental and quasi-experimental designs for generalized causal inference. 2nd edition. Houghton Mifflin, Michigan,2002. Reference Source

2. SAMHSA. AUDIT-C. https://www.integration.samhsa.gov/images/res/tool_auditc.pdf

3. Babor TF, Higgins-Biddle JC, Saunders JB, Monteiro MG AUDIT: The Alcohol Use Disorders Identification Test Guidelines for Use in Primary Care (second edition). https://www.who.int/substance_abuse/publications/audit/en/

4. US Preventive Service task Force. Patient Health Questionnaire PHQ-9. https://www.uspreventiveservicestaskforce.org/Home/GetFileByID/218

5. Center for Quality Assessment and Improvement in Mental Health Patient Health Questionnaire PHQ-2. http://www.cqaimh.org/pdf/tool_phq2.pdf

6. Gual A, Anderson P, Segura L, et al.: Alcohol and Primary health care: Training programme on identification and brief interventions. Barcelona: department of Health of the Government of Catalonia,2005. Reference Source

7. Anderson P, Bendsten P, Spak F, et al.: Improving the delivery of brief interventions for heavy drinking in primary health care: outcome results of the Optimizing Delivery of Health Care Intervention (ODHIN) five-country cluster randomized factorial trial. *Addiction.* 2016;111(11):1935–1945. 27237081 10.1111/add.13476

8. Wensing M, Oxman A, Baker R, et al.: Tailored Implementation For Chronic Diseases (TICD): a project protocol. *Implement Sci.* 2011;6:103. 21899753 10.1186/1748-5908-6-103 3179734

9. Wensing M, Huntink E, van Lieshout J, et al.: Tailored Implementation of Evidence-Based Practice for Patients with Chronic Diseases. *PLoS One.* 2014;9(7):e101981. 25003371 10.1371/journal.pone.0101981 4087017

10. Flottorp SA, Oxman AD, Krause J, et al.: A checklist for identifying determinants of practice: a systematic review and synthesis of frameworks and taxonomies of factors that prevent or enable improvements in healthcare professional practice. *Implement Sci.* 2013;8:35. 23522377 10.1186/1748-5908-8-35 3617095

11. Prasad A, Kano M, Dagg KA, et al.: Prioritizing action on health inequities in cities: An evaluation of Urban Health Equity Assessment and Response Tool (Urban HEART) in 15 cities from Asia and Africa. *Soc Sci Med.* 2015;145(2015):237–242. 26456133 10.1016/j.socscimed.2015.09.031

12. Svoronos T, Mate KS: Evaluating large-scale health programmes at a district level in resource-limited countries. *Bull World Health Organ.* 2011;89(11):831–837. 22084529 10.2471/BLT.11.088138 3209726

13. Barker PM, Reid A, Schall MW: A framework for scaling up health interventions: lessons from large-scale improvement initiatives in Africa. *Implement Sci.* 2016;11(1):12. 26821910 10.1186/s13012-016-0374-x 4731989

14. Heather N: WHO Collaborative Project on Identification and Management of Alcohol-related Problems in PHC – Report to the World Health Organisation on Phase IV: Development of Country-wide Strategies for Implementing Early Identification and Brief Intervention in PHC. Geneva: World Health Organisation, Department of Mental Health and Substance Abuse:2006. Reference Source

15. Keller, P. A., & Lehmann, D. R. (2008). Designing effective health communications: a meta-analysis. *Journal of Public Policy & Marketing*, *27*(2), 117-130.

16. Rice, R. E., & Atkin, C. K. (Eds.). (2012). *Public communication campaigns*. Sage.

For peer review only - http://bmjopen.bmj.com/site/about/guidelines.xhtml

17. OECD: Compendium of OECD well-being indicators. OECD Better Life Initiative.2011; (Accessed 18 December 2016). Reference Source

18. Bertelsmann Stiftung. [website].2016; (Accessed 18 December 2016). Reference Source

19. Inglehart R, Welzel C: Modernization, Cultural Change and Democracy. New York: Cambridge University Press,2005. Reference Source

20. Anderson P, Clement S: The AAPPQ revisited: the measurement of general practitioners' attitudes to alcohol problems. *Br J Addict.* 1987;82(7):753–9. 3478065 10.1111/j.1360-0443.1987.tb01542.x

21 Riley MR, Mohr DC, Waddimba AC. The reliability and validity of three-item screening measures for burnout: Evidence from group-employed health care practitioners in upstate New York. Stress and Health. 2018 Feb;34(1):187-93.

22 Schaufeli WB, Shimazu A, Hakanen J, Salanova M, De Witte H. An Ultra-Short Measure for Work Engagement: The UWES-3 Validation Across Five Countries. European Journal of Psychological Assessment 2017

23 Rosenberg G, Bauld L, Hooper L et al. New national alcohol guidelines in the UK: public awareness, understanding and behavioural intentions. *Journal of Public Health*, 2018 40, 549–556,

24 Anderson P, Wojnar M, Jakubczyk A, Gual A, Reynolds J, Segura L, Sovinova H, Csemy L, Kaner E, Newbury-Birch D, Fornasin A. Managing alcohol problems in general practice in Europe: results from the European ODHIN survey of general practitioners. Alcohol and Alcoholism. 2014 Jul 16;49(5):531-9.

25 Francis J, Eccles MP, Johnston M, Walker AE, Grimshaw JM, Foy R, Kaner EF, Smith L, Bonetti D. Constructing questionnaires based on the theory of planned behaviour: A manual for health services researchers.

26 Bandura A. Guide for constructing self-efficacy scales. Self-efficacy beliefs of adolescents. 2006 Feb 1;5(1):307-37.

27 Bergström A, Skeen S, Duc DM, Blandon EZ, Estabrooks C, Gustavsson P, Hoa DT, Källestål C, Målqvist M, Nga NT, Persson LÅ. Health system context and implementation of evidence-based practices—development and validation of the Context Assessment for Community Health (COACH) tool for low-and middle-income settings. Implementation Science. 2015 Dec;10(1):120.

28 Scott SD, Plotnikoff RC, Karunamuni N, Bize R, Rodgers W. Factors influencing the adoption of an innovation: An examination of the uptake of the Canadian Heart Health Kit (HHK). Implementation Science. 2008 Dec;3(1):41.

29. Moore GF, Audrey S, Barker M, et al.: Process evaluation of complex interventions: Medical Research Council guidance. *BMJ.* 2015;350:h1258. 25791983 10.1136/bmj.h1258 4366184

30. Reeves BC, Wells GA, Waddington H: Quasi-experimental study designs series-paper 5: classifying studies evaluating effects of health interventions-a taxonomy without labels. *J Clin Epidemiol.* 2017; pii: S0895-4356(17)30288-3. 28351692 10.1016/j.jclinepi.2017.02.016

For peer review only - http://bmjopen.bmj.com/site/about/guidelines.xhtml

SPIRIT

STANDARD PROTOCOL ITEMS: RECOMMENDATIONS FOR INTERVENTIONAL TRIALS

SPIRIT 2013 Checklist: Recommended items to address in a clinical trial protocol and related documents*

Section/item	ItemNo	Description	Page
**Administrative information**			
Title	1	Descriptive title identifying the study design, population, interventions, and, if applicable, trial acronym	Title page
Trial registration	2a	Trial identifier and registry name. If not yet registered, name of intended registry	Abstract Page
	2b	All items from the World Health Organization Trial Registration Data Set	Used Clinicaltrials.gov
Protocol version	3	Date and version identifier	Abstract Page
Funding	4	Sources and types of financial, material, and other support	19
Roles and responsibilities	5a	Names, affiliations, and roles of protocol contributors	19-20
	5b	Name and contact information for the trial sponsor	19
	5c	Role of study sponsor and funders, if any, in study design; collection, management, analysis, and interpretation of data; writing of the report; and the decision to submit the report for publication, including whether they will have ultimate authority over any of these activities	19
	5d	Composition, roles, and responsibilities of the coordinating centre, steering committee, endpoint adjudication committee, data management team, and other individuals or groups overseeing the trial, if applicable (see Item 21a for data monitoring committee)	n/a
**Introduction**			
Background and rationale	6a	Description of research question and justification for undertaking the trial, including summary of relevant studies (published and unpublished) examining benefits and harms for each intervention	5-7
	6b	Explanation for choice of comparators	5-7
Objectives	7	Specific objectives or hypotheses	7

For peer review only

For peer review only - http://bmjopen.bmj.com/site/about/guidelines.xhtml

Trial design	8	Description of trial design including type of trial (eg, parallel group, crossover, factorial, single group), allocation ratio, and framework (eg, superiority, equivalence, noninferiority, exploratory)	7
**Methods: Participants, interventions, and outcomes**			
Study setting	9	Description of study settings (eg, community clinic, academic hospital) and list of countries where data will be collected. Reference to where list of study sites can be obtained	8
Eligibility criteria	10	Inclusion and exclusion criteria for participants. If applicable, eligibility criteria for study centres and individuals who will perform the interventions (eg, surgeons, psychotherapists)	8
Interventions	11a	Interventions for each group with sufficient detail to allow replication, including how and when they will be administered	9 + supplement
	11b	Criteria for discontinuing or modifying allocated interventions for a given trial participant (eg, drug dose change in response to harms, participant request, or improving/worsening disease)	n/a
	11c	Strategies to improve adherence to intervention protocols, and any procedures for monitoring adherence (eg, drug tablet return, laboratory tests)	11-12
	11d	Relevant concomitant care and interventions that are permitted or prohibited during the trial	n/a
Outcomes	12	Primary, secondary, and other outcomes, including the specific measurement variable (eg, systolic blood pressure), analysis metric (eg, change from baseline, final value, time to event), method of aggregation (eg, median, proportion), and time point for each outcome. Explanation of the clinical relevance of chosen efficacy and harm outcomes is strongly recommended	14
Participant timeline	13	Time schedule of enrolment, interventions (including any run-ins and washouts), assessments, and visits for participants. A schematic diagram is highly recommended (see Figure)	Timetable in Supplement
Sample size	14	Estimated number of participants needed to achieve study objectives and how it was determined, including clinical and statistical assumptions supporting any sample size calculations	16-17

For peer review only - http://bmjopen.bmj.com/site/about/guidelines.xhtml

Recruitment	15	Strategies for achieving adequate participant enrolment to reach target sample size	16
**Methods: Assignment of interventions (for controlled trials)**			
Allocation:			8
Sequence generation	16a	Method of generating the allocation sequence (eg, computer-generated random numbers), and list of any factors for stratification. To reduce predictability of a random sequence, details of any planned restriction (eg, blocking) should be provided in a separate document that is unavailable to those who enrol participants or assign interventions	8
Allocation concealment mechanism	16b	Mechanism of implementing the allocation sequence (eg, central telephone; sequentially numbered, opaque, sealed envelopes), describing any steps to conceal the sequence until interventions are assigned	8
Implementati on	16c	Who will generate the allocation sequence, who will enrol participants, and who will assign participants to interventions	19
Blinding (masking)	17a	Who will be blinded after assignment to interventions (eg, trial participants, care providers, outcome assessors, data analysts), and how	n/a
	17b	If blinded, circumstances under which unblinding is permissible, and procedure for revealing a participant's allocated intervention during the trial	n/a
**Methods: Data collection, management, and analysis**			
Data collection methods	18a	Plans for assessment and collection of outcome, baseline, and other trial data, including any related processes to promote data quality (eg, duplicate measurements, training of assessors) and a description of study instruments (eg, questionnaires, laboratory tests) along with their reliability and validity, if known. Reference to where data collection forms can be found, if not in the protocol	9-12
	18b	Plans to promote participant retention and complete follow-up, including list of any outcome data to be collected for participants who discontinue or deviate from intervention protocols	14-15

Data management	19	Plans for data entry, coding, security, and storage, including any related processes to promote data quality (eg, double data entry; range checks for data values). Reference to where details of data management procedures can be found, if not in the protocol	Data mgt plan added as Annexe
Statistical methods	20a	Statistical methods for analysing primary and secondary outcomes. Reference to where other details of the statistical analysis plan can be found, if not in the protocol	14-16
	20b	Methods for any additional analyses (eg, subgroup and adjusted analyses)	14-16
	20c	Definition of analysis population relating to protocol non-adherence (eg, as randomised analysis), and any statistical methods to handle missing data (eg, multiple imputation)	14-16
**Methods: Monitoring**			
Data monitoring	21a	Composition of data monitoring committee (DMC); summary of its role and reporting structure; statement of whether it is independent from the sponsor and competing interests; and reference to where further details about its charter can be found, if not in the protocol. Alternatively, an explanation of why a DMC is not needed	DMC not required
	21b	Description of any interim analyses and stopping guidelines, including who will have access to these interim results and make the final decision to terminate the trial	n/a
Harms	22	Plans for collecting, assessing, reporting, and managing solicited and spontaneously reported adverse events and other unintended effects of trial interventions or trial conduct	11
Auditing	23	Frequency and procedures for auditing trial conduct, if any, and whether the process will be independent from investigators and the sponsor	n/a
**Ethics and dissemination**			
Research ethics approval	24	Plans for seeking research ethics committee/institutional review board (REC/IRB) approval	19
Protocol amendments	25	Plans for communicating important protocol modifications (eg, changes to eligibility criteria, outcomes, analyses) to relevant parties (eg, investigators, REC/IRBs, trial participants, trial registries, journals, regulators)	17

For peer review only - http://bmjopen.bmj.com/site/about/guidelines.xhtml

Consent or assent	26a	Who will obtain informed consent or assent from potential trial participants or authorised surrogates, and how (see Item 32)	17 and Annexe Data Mgt Plan
	26b	Additional consent provisions for collection and use of participant data and biological specimens in ancillary studies, if applicable	n/a
Confidentiality	27	How personal information about potential and enrolled participants will be collected, shared, and maintained in order to protect confidentiality before, during, and after the trial	17 and Annexe Data Mgt Plan
Declaration of interests	28	Financial and other competing interests for principal investigators for the overall trial and each study site	19
Access to data	29	Statement of who will have access to the final trial dataset, and disclosure of contractual agreements that limit such access for investigators	19
Ancillary and post-trial care	30	Provisions, if any, for ancillary and post-trial care, and for compensation to those who suffer harm from trial participation	n/a
Dissemination policy	31a	Plans for investigators and sponsor to communicate trial results to participants, healthcare professionals, the public, and other relevant groups (eg, via publication, reporting in results databases, or other data sharing arrangements), including any publication restrictions	19
	31b	Authorship eligibility guidelines and any intended use of professional writers	19-20
	31c	Plans, if any, for granting public access to the full protocol, participant-level dataset, and statistical code	19
**Appendices**			
Informed consent materials	32	Model consent form and other related documentation given to participants and authorised surrogates	Annexe Data Mgt Plan
Biological specimens	33	Plans for collection, laboratory evaluation, and storage of biological specimens for genetic or molecular analysis in the current trial and for future use in ancillary studies, if applicable	n/a

*It is strongly recommended that this checklist be read in conjunction with the SPIRIT 2013 Explanation & Elaboration for important clarification on the items. Amendments to the protocol should be tracked and dated. The SPIRIT checklist is copyrighted by the SPIRIT

For peer review only - http://bmjopen.bmj.com/site/about/guidelines.xhtml

Group under the Creative Commons "Attribution-NonCommercial-NoDerivs 3.0 Unported" license.

For peer review only

SCALA - DATA MANAGEMENT PLAN

Draft version 1: 23 January, 2018
Draft version 2: 1 February, 2018
Draft version 3: 27 February, 2018
Draft version 4: 2 March, 2018
Draft version 5: 15 March, 2018
Draft version 6: 15 May, 2018
Draft version 7: 23 May, 2018
version 8: 24 May, 2018

Abbreviations and definitions:

DMP	= data management plan
IRB	= Institutional Research Board
PHCCs	= primary health care centres
SCALA	= Scale-up of Prevention and Management of Alcohol Use Disorder in Latin America
Data center	= Technische Universität Dresden, Germany (supervisor: Jürgen Rehm)

Contents

1. Data Summary ... 1
2. FAIR data ... 5
2.1. Making data findable, including provisions for metadata ... 5
2.2. Making data openly accessible ... 5
2.3. Making data interoperable ... 6
2.4. Increase data re-use (through clarifying licences) ... 7
3. Allocation of resources ... 8
4. Data security ... 9
5. Ethical aspects ... 10
6. Other issues ... 11
7. Data analysis plan ... 12
7.1. REACH ... 13
7.2. EFFECTIVENESS ... 14
7.3. ADOPTION ... 14
7.4. IMPLEMENTATION ... 15
7.5. MAINTENANCE ... 15
8. Appendix ... 18

For peer review only

1. Data Summary

Introduction

During the course of the SCALA study, quantitative, qualitative, as well as publicly available data will be collected in PHCCs in three American countries: Mexico, Peru, Colombia. All collected data are required for a thorough evaluation of the main study goal and it corollaries, ie. to improve alcohol management in PHCCs by increasing screening rates and delivery of adequate advice and treatment for screen positives. The following qualitative and quantitative data will be obtained from patients and providers in PHCCs. All data will be transferred first to the data center serving as SCALA data repository at the TU Dresden (for details on data transfer, see ***section 4***). After cleaning the data and bringing it into the standard format (for details, see ***section 2.2***), the data will be forwarded to partners based on the workplan or upon request. While all data will be kept with the data center, they are collectively owned by all partners.

Data origin

Q1) **PHCC structure data (quantitative):**
Collection of data from the participating PHCCs before start of data collection. The PHCC administration will be asked to fill out a form (see '***Q1_PHCC Description Form.pdf***'), including the number of registered patients, as well as number of health professionals working in the centre. The data will be entered into spreadsheets (see '***Q1_PHCC Description Form_spreadsheet template.xlsx***'), which will then be sent to the data center.

Q2) **Short tally sheet for routine care data (quantitative):**
Collection of routine care data on **all** adult patients consulting PHCCs. For this purpose, a tally sheet (see '***Q2_Short Patient Tally Sheet.pdf***') will be applied to collect all necessary information on sociodemographics (sex, age, socioeconomic status) and drinking patterns (AUDIT-C) for all patients. For screen positives, the tally sheet will also capture the results of in-depth assessment of alcohol problems (AUDIT) and depression (PHQ-2 and - if above threshold - PHQ-9) and the decisions made concerning brief advice and treatment and referral to specialist care. The tally sheets will be collected by local researchers on a weekly basis and entered into spreadsheet templates (see '***Q2_Short Patient Tally Sheet_spreadsheet template.xlsx***'). These spreadsheets will be submitted monthly to the data center.

Q3) **Long tally sheet for quality control data (quantitative):**
Collection by respective PHCC of a more extensive set of routine care data for quality control on **a subset** of adult patients consulting PHCCs. Quality control data will only be collected during predefined periods during the 18 months implementation period, resulting in about 1 in 10 patients being assessed. In order to allow for comparisons between long tally sheet and interview data, the periods for application of long tally sheets will be aligned with realisation of patient interviews. The long tally sheet will cover all variables from the short tally sheet (see Q2 and '***Q3_Long Patient Tally Sheet.pdf***'), in addition to assessment of educational level (1 question), attempts on cutting down drinking (2 questions), alcohol health literacy (4 questions), and injunctive social norms (2 questions). As with short tally sheets, long tally sheets will also be collected weekly by local researchers and entered into spreadsheet templates (see '***Q3_Long Patient Tally Sheet_spreadsheet template.xlsx***'). These spreadsheets will be submitted to the data center whenever data were collected.

For peer review only - http://bmjopen.bmj.com/site/about/guidelines.xhtml

Q4) Tally Sheets Cover Form (quantitative):
Short and long tally sheets will be distributed to the PHCCs by local researchers on a weekly basis and each set of tally sheets will have a cover form (see '***Q4_Tally Sheets Cover Form.pdf***'). On this cover form, the PHCC administration will be asked to fill in the number of adult consultations during the respective week for each participating provider. The cover forms will be collected together with the short/long tally sheets and will be entered in the same spreadsheets and then submitted to the data center.

Q5) Tally Sheet Appendix (consent taking for patient interview):
In predefined weeks during month 3 of the 18-month implementation period, PHCC providers will ask all patients to participate in researcher-conducted personal interviews. Patient consent and contact details will be collected on a form appended to either short or long tally sheets during these weeks (see '***Q5_Patient Tally Sheet Appendix.pdf***'). To allow for a stratified sampling of interviewees according to screening results (ratio of positively and negatively screened patients = 2:1) by local researchers, the providers will also note down the AUDIT-C screening result on the form. These forms will be collected alongside the short/long tally sheets and the data will only be used to sample and recruit interviewees.

Q6) Patient interview data:
Collection of individual data through patient interviews at month 3 and subsequent follow-ups at months 6 and 12. Random samples of positively and negatively screened patients (ratio 2:1) will be interviewed across all municipalities, resulting in a total number of N=1,080 patients. The interview will contain all questions from the long tally sheet (see '***Q3_Long Patient Tally Sheet.pdf***'), in addition to 2 questions for quality control assessing experience of screening/brief advice with PHCC providers, a six-item modified version of the HLS-EU-16 to assess alcohol health literacy, the World Health Organization Disability Assessment Schedule to assess the degree of disability, and questions on health resource utilization (see '***Q6_Patient Interview.pdf***'). The patient interview will be conducted as face-to-face or telephone interview and collected data will be entered into prepared spreadsheets (see '***Q6_Patient interview_spredsheet sample.xlsx***') and sent to the data center.

Q7) Provider questionnaire data (quantitative):
Collection of data from health care providers, which will be assessed prior to or during the 4-week baseline period and repeated at months 4.5 and 13.5. All providers will be asked to fill out questions on alcohol knowledge, alcohol health literacy, as well as on attitudes towards alcohol users and alcohol problems (SAAPP Questionnaire, see '***Q7_Provider questionnaire.pdf***'). The data will be entered into prepared spreadsheets (see '***Q7_Provider questionnaire_spredsheet sample.xlsx***') and sent to the data center.

Q8) Provider interview data (qualitative):
At the end of the 18-month implementation period, a random sample of 1 in 20 PHCC providers of both control and intervention groups will be invited to participate in a 15 minute semi-standardized interview (see '***Q8_Provider Interview from Annexe 25.pdf***'), which will be taped and conducted via telephone. The interviews aim to assess provider experiences on implementing the intervention package in their routines. Recordings of the provider interviews will be transcribed.

Q9) Process data interviews (qualitative):

For peer review only - http://bmjopen.bmj.com/site/about/guidelines.xhtml

As part of the process evaluation, semi-structured focus-group interviews will be conducted with the User Panels, Community Advisory Boards, and local research groups. The focus groups will cover the topics of tailoring of materials, and decision making processes for adoption mechanisms, support systems, and completing driver diagrams and barriers and facilitator tables.

Q10) Recruitment documentation (quantitative):

Local researchers will be given forms to document the entire PHCC recruitment process (see '***Q10_Recruitment documentation.pdf***'). For each municipality, they will document the total number of PHCCs and the number of contacted PHCCs for study participation. Among contacted PHCCs, the number of non-responding, refusing, and accepting PHCCs will be assessed. For each PHCC contacted for study participation, the following data will be assessed: number of registered patients and number of workers, type and number of contacts with PHCC, PHCC response (acceptance, refusal, non-response), and reasons for refusal or non-response if applicable. The data will be entered into prepared spreadsheets (see '***Q10_Recruitment documentation_spreadsheet template.xlsx***') and sent to the data center.

Q11) Follow-up documentation (quantitative):

Local researchers will monitor key activities of each PHCC provider during the course of the study using a standardized sheet (see '***Q11_Follow-up documentation.pdf***'). Key activities to be documented relate to participation in training sessions and potential reasons for non-participation. If providers drop out of the study prior to end of the 18 months implementation period, this will also be documented, in addition to any reasons for drop out. On the same follow-up documentation form, sex and age of the provider will be assessed as well. The data will be entered into prepared spreadsheets (see '***Q11_Follow-up documentation_spreadsheet template.xlsx***') and sent to the data center.

All quantitative data will be collected directly by PHC providers and the country research teams, through patient interviews or provider surveys.

Data types, format, and size

The total size of all quantitative data collected in the course of this study is unlikely to exceed 100MB and will be stored as easily accessible spreadsheets (.csv - format). Transcripts from qualitative interviews will be stored as Microsoft Word documents (.docx - format), not exceeding 100MB in total.

Purpose of data collection with regard to study objectives

The quantitative data will be required to evaluate if study objectives can be reached (for an overview of the study objectives, see '***Figure_RE-AIM.png***'). In particular, Q2 (short tally sheet), Q3 (long tally sheet) and Q4 (patient interview) data will provide outcome measures, which allows for evaluation of the ***REACH*** (maximising exposure to screening and brief advice/treatment in PHC) and ***EFFECTIVENESS*** (increasing adequate alcohol management in PHC) study objectives.

All qualitative data will be obtained through interviews with User Panels, Community Advisory Boards, local research groups, patients and providers, which will be used to evaluate the ***IMPLEMENTATION*** (factors affecting the implementation of intervention package) and ***ADOPTION*** (increase adoption of the intervention package in PHC) study objectives.

For peer review only - http://bmjopen.bmj.com/site/about/guidelines.xhtml

Furthermore, publicly available and process data will be obtained during the course of the study. In detail, this will comprise information necessary to characterize countries, cities and municipalities, contextual, political, socio-economic, and alcohol policy factors (e.g. legislation), and a thorough description of Community Advisory Boards. These data will contribute to the process evaluation (Work Package 5) and serve as base to evaluate the ***MAINTENANCE*** (long term effects of implementation) study objective.

A detailed description of the analytic steps planned to achieve study objectives can be found in ***section 7***.

Re-using data

Most of the data collected during the course of this study will be primary data collected through health care professionals and from patients directly. However, publicly available data form an important pillar in this study as it will be required for process evaluation and economic analyses.

Data utility

The collected data will not only be used to achieve the above listed study goals; they can be used by other researchers to plan similar studies, to examine other hypotheses, or for population modelling purposes.

For peer review only

2. FAIR data

2.1. Making data findable, including provisions for metadata

Making data discoverable, identifiable, and locatable

All quantitative data sets will be made publicly available through the UK Data Service after publication of the results, or, at the latest, 12 months after the finalization of the study.[1] Each data set published with the UK Data Service will be attached with a unique 'Digital Objective Identifier' (DOI).

Data derived from qualitative interviews will not be stored in the UK data archive as anonymity of qualitative interviews cannot be ensured.

Naming conventions and version numbers

For all data sets a predefined title standard ("SCALA_data_*NAME*_v1_*DATE.csv*") and the same author group ("SCALA study group") will always be used. Within titles, consecutive version numbers will be used to facilitate updates and corrections to uploaded data sets and to ensure unambiguous identification of data sets.

Key word conventions

All stored data will be labelled with the following keywords: SCALA, Americas, Mexico, Peru, Colombia, Primary Health Care, Alcohol, Heavy Drinking, Depression, Prevention, Screening, Brief Advice, Treatment. Additional keywords will be considered to characterize the respective data set. As data on resource use will be used for economic analyses, data sets containing relevant data will further be classified using 'JEL Classification Codes'.[2]

Meta data handling

There are no standards on handling metadata in this discipline and there is no intention to manage metadata of the publicly stored data sets apart from the measures listed above.

2.2. Making data openly accessible

Making data openly available

By default, all quantitative datasets generated in the course of the SCALA study will be made openly available through the UK Data Service upon publication of the results. Prior to publication, all data will be formatted to meet UK Data Service requirements.

Access conditions and required software

All quantitative data will be provided as 'comma separated values' (CSV) – an efficient and open source format to store larger data sets. This is a generic, widely used file format, which can be handled by all major software packages used for quantitative analyses (eg. Microsoft Excel, SAS, SPSS, Stata, R). In

[1] http://www.data-archive.ac.uk/

[2] https://en.wikipedia.org/wiki/JEL_classification_codes

For peer review only

order to maintain accessibility, large data sets will be split into smaller parts, which will not exceed 50 MB file size.

Depositing metadata, documentation, and code

Each dataset stored with the UK Data Service will be accompanied by a set of documenting files, which comprises relevant publications, consent forms, questionnaires/interview guidelines, and codebooks. The codebooks stored alongside the dataset will be Excel files (".xlsx") that contain extensive metadata for each variable in the associated data set, such as original questions, value labels, defined missing values, and possible coding rules applied.

Arrangements with the UK Data Service

The UK Data Service has been contacted and the study team received a positive response with regard to storing study data with the service. When preparing files to be published online, guidelines and checklists of the UK Data Service will be considered (see [3,4]). Licence agreements will be finalized after obtaining approval of all IRBs.

Data not being made available

All qualitative data will be generated from semi-standardized interviews. Excerpts of these interviews will be appended to respective publications if applicable. However, full interview transcripts will not be published for the following reasons: first, sharing full interview transcripts is uncommon in this field; and, second, sharing poses a potential risk for disclosing the identity of the interviewee.

Restrictions of use and data access committee

As all relevant data will be made publicly available, there will be no need for a data access committee. If other researchers wish to examine interview transcripts, fully anonymized excerpts can be made available through the responsible researchers.

Ascertainment of identity of person accessing the data

It is aimed that all relevant data are to be shared as 'Open Data'.[5] This will imply that all data will be fully anonymized and there will be no means necessary to ascertain the identity of persons accessing the data.

2.3. <u>Making data interoperable</u>

Interoperability of data

All gathered data will be completely interoperable as they will be stored in widely used data formats, which make them accessible by a broad spectrum of data processing software packages, including open source applications.

---

[3] https://www.ukdataservice.ac.uk/deposit-data/preparing-data
[4] https://www.ukdataservice.ac.uk/media/440320/depositsurvey.pdf
[5] https://www.ukdataservice.ac.uk/get-data/data-access-policy/open-data

For peer review only - http://bmjopen.bmj.com/site/about/guidelines.xhtml

Data and metadata vocabularies, standards, or methodologies

As there is no standard vocabulary set for variable names in our discipline, a simple and easy-to-comprehend nomenclature will be developed and applied to all quantitative data sets and summarized in accompanying codebooks. For prospective assessments on the same individuals, data sets will be structured in a 'long data format', i.e. one variable will indicate the time of assessment of the same variables (see [6] for a more comprehensive explanation).

2.4. Increase data re-use (through clarifying licences)

Data licence

All study data stored with the UK Data Service will be published as "open data" if possible. For this storage mode, the information in the data set will not allow disclosure of any respondents. "Open data" is published using the Open Government Licence[7] and users will have direct access of data without prior registration with UK data service, facilitating wide reach and potential re-use of data collected in this study.

Time of data availability

All quantitative data sets will be made publicly available after publication of the results, or, at the latest, 12 months after the finalization of the study.

Duration of data storage

All data stored with the UK Data Service are held in perpetuity (see [8]).

Re-use by third parties

Data re-use by third parties is explicitly encouraged and will be facilitated by publication of codebooks and documentation along the data sets.

Data quality assurance processes

Prior to sharing the data with the UK Data Service, the study team will clean the data to ensure internal consistency. Several checks of the study team will be conducted before the data will be shared publicly.

[6] http://www.theanalysisfactor.com/wide-and-long-data/
[7] http://www.nationalarchives.gov.uk/doc/open-government-licence/version/2/
[8] https://www.ukdataservice.ac.uk/media/173249/UKDS_Collections_Development_Policy_02_00.pdf

For peer review only - http://bmjopen.bmj.com/site/about/guidelines.xhtml

3. Allocation of resources

Costs for open access publications

In total, the study budget includes €36,000 to pay 'open access' publication licence fees.

Costs for sharing data through repository

Storage of study data with the UK Data Service does not require any fees.

Long term costs for preservation

No long term costs are anticipated.

Data protection, data transfer and data sharing

The Data Protection Officers of both Technical University Dresden and of Maastricht University are the focal points for reviewing data protection, data transfer and data sharing, and required ethics reporting.

For peer review only

4. Data security

Data security - transfer

All collected data will be transferred to the data center in encrypted packages created with the open access 7-zip software. The 'Advanced Encryption Standard' (AES) with 256 bits will be applied, which has been widely recognized as standard encryption technique [9]. The same data transfer methods will be used to transfer the data to the other partners who request or need the data.

Copies of transcribed data notes that are required for the process evaluation in Work Package 6 will be sent by registered courier to ESADE.

Data security - storage

All electronic data will be stored on encrypted hard drives by respective partners. This will include mail communication, study documentation and codes applied to manipulate data and to generate results. Backup hard drives will be used to facilitate recovery of lost data.

All analogue data sources (tally sheets, interview notes, etc.) will be kept by the local research teams, where the data will be kept and stored adhering to local regulations.

All data stored with the UK Data Service are securely kept for perpetuity.

For peer review only

[9] https://en.wikipedia.org/wiki/Advanced_Encryption_Standard

5. Ethical aspects

Ethical or legal issues regarding data sharing

After collection of the raw data, local researchers will assign predefined identification codes to each individual and remove all potentially identifying information from the data. The key to match individuals to the assigned identification code will remain with the local researchers. After the data has been securely transferred to the data center for cleaning and subsequent analyses, there will be no possibility no identify individuals from the data.

All data collection, processing, and sharing procedures will adhere to national and international laws including the General Data Protection Regulation (EU Regulation 2016/679).

The SCALA study team currently seeks approval for the study design, data collection and analysis from the research ethics board at the TU Dresden, Germany (registration number: 'EK 90032018'). In addition, ethical review is currently under way in Colombia, Mexico, and Peru.

Informed consent for data sharing and long term preservation

Informed consent will be obtained from providers and patients providing individual level data (through interviews or questionnaires) to allow data sharing through the UK Data Service.

For peer review only

6. Other issues

Use of other procedures for data management

Data management in the SCALA study will adhere to EU Regulation 2016/679. There are no further national or institutional requirements which would counteract or extend this regulation or any of the procedures specified in this document.

For peer review only

7. Data analysis plan

In Section 1, data sources are mapped to study goals. For each study goal, the required definition of variables and planned statistical analyses are described in the following.

General considerations

Given that SCALA is a quasi-experimental study design (technically, a non-randomized controlled trial (NRCT)), data for a range of potential confounders will be collected at baseline (with repeat measurements during the course of the 18-month implementation period) both to undertake propensity score matching between intervention and comparator municipalities, and include as confounders in the statistical analyses:

At the level of the PHCC, PHC-provider and patient:

- *Age, sex and profession (doctor, nurse, other health care worker) of provider:* Evidence suggests that the sex and age of the provider are unimportant in influencing screening and advice rates, whereas profession is. Nurses tend to screen more patients than doctors; doctors tend to advise more screen positive patients than nurses.
- *Number of monthly consultations:* Evidence suggests that the higher the number of consultations, the lower the proportion of patients screened.
- *Attitudes and knowledge of providers:* Evidence suggests that providers with more positive attitudes, in terms or role security and therapeutic commitment, and providers with high levels of alcohol-related knowledge, are more likely to screen and advise a greater proportion of patients.
- *AUDIT-C score:* The evidence suggests that the higher the AUDIT-C score, the greater the likelihood that screen positive patients will be given advice.

At the level of the municipality:

- A priori, comparator municipalities have been chosen to be similar to intervention municipalities in terms of socioeconomic and other characteristics which impact on drinking, health care and survival, comparable community mental health services. During the set-up phase, additional data will be collected form the municipalities on existing actions and training of PHC-based screening and brief advice for heavy drinking; availability and accessibility of specialist services for severe AUD and moderately severe or severe depression; and, existing municipal-based prevention and/or policy programmes to reduce heavy drinking

7.1. REACH

Primary outcome measures:

A1 Number of intervened patients per provider and per PHCC

Secondary outcome measures:

A2 Number of screened patients per provider and per PHCC
A3 Number of advised patients per provider and per PHCC
A4 Number of patients referred for severe AUD per provider and per PHCC

For peer review only - http://bmjopen.bmj.com/site/about/guidelines.xhtml

A5 Number of patients referred for moderately severe or severe depression per provider and per PHCC

A6 Provider attitudes

A7 Provider alcohol health literacy

A8 Representativeness of population intervened for AUD

Definition:

Measure A1 represents the *primary* outcome variables in this study and is assessed in three 4-week periods: in the first month 1 (t1), after 9 months (t2) and after 18 months (t3). It will be the proportion of consulting adult patients (aged 18+ years) intervened (screened and advice given to screen positives), calculated as the number of AUDIT-C positive patients that received oral advice or referral for advice to another provider in or outside the PHCC, divided by the total number of adult consultations of the participating providers per provider and per PHCC.

Measures A2 to A5 represent *secondary* outcome variables in this study and are assessed in the same three 4-week periods as measure A1: in the first month 1 (t1), after 9 months (t2) and after 18 months (t3). Measure A2 will be the proportion of patients screened, calculated as the number of completed screens divided by the total number of consultations of all adult patients per participating provider, and averaged per participating PHCC. Measure A3 will be the proportion of patients advised, calculated as the number of brief interventions delivered (received oral brief advice, and/or were referred to another provider in or outside the practice), divided by the total number of screen positives per participating provider and averaged per participating PHCC. Information will also be collected on the number of screen negatives who received brief advice. Measure A4 will be the proportion of patients with severe AUD referred to specialist treatment, calculated as the proportion of patients with an AUDIT-C score ≥8 and a full AUDIT score ≥20 documented as referred to treatment per participating provider, and per participating PHCC. Measure A5 will be the proportion of patients with moderately severe or severe depression referred to specialist treatment, calculated as the proportion of patients with an AUDIT-C score ≥8 and a PHQ-9 score ≥15 documented as referred to treatment per participating provider, and per participating PHCC.

Measures A6 and A7 are also *secondary* outcome variables in this study and will be assessed in three 4-week periods through provider questionnaires: at baseline (t1), after 4.5 months (t2) and after 13.5 months (t3). Measure A6 will be measured by the SAAPP questionnaire, with responses to be summed within the two scales of role security and therapeutic commitment. Individual missing values for any of the items in a domain will be assigned the mean value of the remaining items of the domain before summation. Measure A7 will be assessed through knowledge of risks due to drinking, and reported descriptive and injunctive social norms of drinking. Measure A8 will be determined through process evaluation activities conducted throughout the implementation period. Among other things, representativeness will be evaluated through comparing patients with people living in the catchment area of the respective PHC on a number of variables.

Analyses/Achievement:

For all measures, means and/or proportions (as applicable) will be presented descriptively by country, control and intervention municipality, and for the total sample. Given the relative rarity of some events (eg. measure A1 to A5) and the resulting distribution, we will use exact inference methods for comparison of intervention vs. comparator municipalities.

For further analyses, including covariates, regression models will be used, taking into consideration the hierarchical nature of the data, and characteristics at different hierarchy levels (i.e., characteristics of the PHCC, characteristics at the municipal level, such as patterns of drinking). Multilevel models are well suited for this purpose and will be built to evaluate the intervention effect for measures A1 to A7. For the primary outcome, the model will be built as follows:

- *Dependent variable:* proportion of patients intervened among all consultations per provider and per PHCC
- *Independent variable 1:* Time (t1-t3)
- *Independent variable 2*: Control vs. intervention municipality
- *Hierarchical cluster*: Provider nested within PHCC nested within country (to control for design effects)
- *Statistic*: Interaction effect between time and group allocation

After testing for the necessary assumptions, the above outlined generalized linear model will be applied to the actual distribution of the outcome measure. Thus, skewness of data resulting from rare events would be analysed using zero-inflated negative binomial regression. For all remaining outcome measures, similar models will be applied.

7.2. EFFECTIVENESS

Outcome measures:

B1 Increased health literacy in PHCC patients using a modified version of the UK-based Newest Vital Sign and a six-item adapted version of Health Literacy Survey-EU Questionnaire (HLS-EU-16)

B2 Reduction in alcohol consumption of AUD+ drinkers

Definition:

Data for measures B1 and B2 are collected through patient interviews (conducted in month 3, 6 and 12).

Analyses/Achievement:

Similar multilevel regression models as applied for primary and secondary outcomes mapped to study goal *REACH* will be applied to measures B1 and B2. The main difference will be that these measures will be analyzed on the individual level, which requires adding another level (patient nested with provider nested within PHCC nested within country) to the model.

7.3. ADOPTION

Outcome measures:

C1 Adoption rate and representativeness of PHCCs

C2 Adoption rate and representativeness of PHCC staff

Definition:

Adoption rate of PHCCs will be calculated as the number of PHCCs agreeing to be part of the study divided by the number of PHCCs contacted.

For peer review only

Adoption rate of PHCC providers within each PHCC that joins the study will be calculated as the number of PHCC providers agreeing to be part of the study divided by the total number of PHCC providers within each PHCC, stratified by profession (doctor, nurse, other).

Analyses/Achievement:

To determine the representativeness of PHCCs involved in the study, routine available data on the size, number of registered patients, and number and characteristics of staff will be used and compared between PHCCs who agreed to be part of the study and contacted PHCCs who declined to be part of the study.

To determine the representativeness of PHCC staff within the involved PHCC, routine available data on the number and characteristics of staff will be used to compare, within each PHCC, those staff who joined the study and those staff who declined to join the study.

7.4. IMPLEMENTATION

Outcome measures:

D1 Extent primary health care screening and advice package delivered as intended

D2 Multi-level evaluation of barriers/facilitators to scale-up using WHO's Urban Health Equity Assessment and Response Tool

D3 Extent implementation on city levels delivered as intended using Medical Research Council guidance

D4 Cost of package implementation

Definition:

All measures D1 to D3 will be assessed through process evaluation activities. The required data will be obtained through interviews with PHCC providers (D1) and with members from Community Advisory Boards (D2, D3). For D4, a comprehensive set of data will be required, comprising patient data on disability and health resource utilization obtained from patient interviews as well as data on unit costs obtained from public data sources.

Analyses/Achievement:

Measures D1 to D3 will be analyzed through qualitative evaluation. Measure D4 will be evaluated by a comprehensive economic evaluation, for which different sources of costs will be considered, such as costs attributable to implementation of the intervention routine as well as costs attributable to utilization of health care services. In a cost-effectiveness study, the hypothesized gain in quality of life among patients in intervention municipalities will be contrasted with recorded and calculated costs.

7.5. MAINTENANCE

Process measures:

E1 Assessment of outcomes 18 months post implementation

E2 Indicators of program-level maintenance

For peer review only

E3 Measures of cost of maintenance
E4 Dissemination / events

Definition:

For measure E1 data from PHC providers and patients up to 18 months after implementing the alcohol management routine need to be collected.

For measure E2, the required indicators will be collected through process evaluation activities, namely interviews with members of the Community Advisory Boards.

For measure E3, all costs will be collected throughout the implementation period within the economic evaluation framework (see measure D4), in order to estimate the costs of maintenance.

For measure E4, the study results will be disseminated through municipal, national, and international structures, following the 'Communication, Dissemination and Exploitation Plan'.

Analyses/Achievement:

Measure E1 will be achieved by continuous data collection across the entire implementation period of 18 months.

Measure E2 will be achieved by analysis of qualitative data. Measure E3 will be achieved through an economic evaluation of the implementation package considering the entire implementation period.

Measure E4 will be achieved by following the 'Communication, Dissemination and Exploitation Plan'.

For peer review only

8. Appendix

List of all documents referenced in the DMP:

Document	Page Number
1. Q1_PHCC Description Form template.pdf	18
2. Q1_PHCC Description Form_spreadsheet template.xlsx	Excel not attached
3. Q2_Short Patient Tally Sheet.pdf	19
4. Q2_Short Patient Tally Sheet_spreadsheet template.xlsx	Excel not attached
5. Q3_Long Patient Tally Sheet.pdf	22
6. Q3_Long Patient Tally Sheet_spreadsheet template.xlsx	Excel not attached
7. Q4_Tally Sheet Cover Form.pdf	26
8. Q5_Tally Sheet Appendix.pdf	27
9. Q6_Patient Interview.pdf	29
10. Q6_Patient interview_spreadsheet template.xlsx	Excel not attached
11. Q7_Provider questionnaire.pdf	34
12. Q7_Provider questionnaire_spreadsheet template.xlsx	Excel not attached
13. Q8_Provider Interview from Annexe 25.pdf	36
14. Q10_Recruitment documentation.pdf	53
15. Q10_Recruitment documentation_spreadsheet template.xlsx	Excel not attached
16. Q11_Follow-up documentation.pdf	55
17. Q11_Follow-up documentation_spreadsheet template.xlsx	Excel not attached
18. Figure_RE-AIM.png	58

For peer review only

PHCU Description Form

Country and municipality details
(to be filled in by local research team)

**Country**	☐ Colombia	☐ Mexico		☐ Peru
**Municipality**	________________	**Control or Experimental**		☐ Control<br>☐ Experimental
**ID of PHCU**	________________			

PHCU details
(to be filled in by PHC administration)

**Name/Address of PHCU**		________________	
**Total number of registered patients**		________________	
**Total number of registered *adult* (18+) patients**		________________	
**Number of workers working in PHCU**	General Practitioners	**Part time**	________________
		**Full time**	________________
	Nurses	**Part time**	________________
		**Full time**	________________
	Assistants	**Part time**	________________
		**Full time**	________________
	Psychologists	**Part time**	________________
		**Full time**	________________
	Social workers	**Part time**	________________
		**Full time**	________________
	Others: __________	**Part time**	________________
		**Full time**	________________

For peer review only

Short Tally Sheet

Provider details and consultation

**Practice ID** (*pre-printed*)	________________	**Provider ID / Name** (*pre-printed*)	________________
**Date consultation**	____ / ____ / ____		

Patient details

**Sex**	☐ Male<br>☐ Female<br>☐ Other	**Age**	______ years
**Socioeconomic status**	☐ Below average	☐ Average	☐ Above average

AUDIT-C Alcohol Screening

Questions		0	1	2	3	4	Score
1	How often do you have a drink containing alcohol?	Never	Monthly or less	2-4 times per month	2-3 times per week	4+ times per week	
2	How many units of alcohol do you drink on a typical day when you are drinking?	1-2	3-4	5-6	7-9	10+	
3	How often do you have 6 or more units on one occasion?	Never	Less than monthly	Monthly	Weekly	Daily or almost daily	

Standard Drinks Placeholder

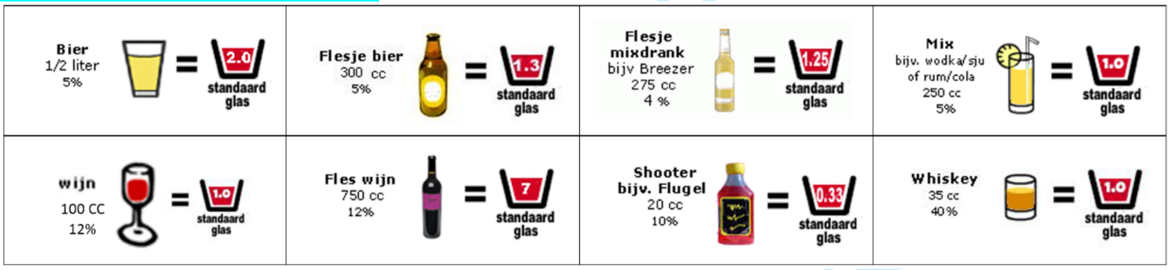

**Sum score AUDIT-C (possible range 0-12)**	__ __
**If AUDIT-C score ≥ 8 => Apply remaining AUDIT and PHQ-2 questionnaire**	

AUDIT (remaining scale)

Questions		0	1	2	3	4	Score
4	How often during the last year have you found that you were not able to stop drinking once you had started?	Never	Less than monthly	Monthly	Weekly	Daily or almost daily	
5	How often during the last year have you failed to do what was normally	Never	Less than monthly	Monthly	Weekly	Daily or almost daily	

For peer review

Short Tally Sheet

#	Question						Score
	expected from you because of drinking?						
6	How often during the last year have you needed a first drink in the morning to get yourself going after a heavy drinking session?	Never	Less than monthly	Monthly	Weekly	Daily or almost daily	
7	How often during the last year have you had a feeling of guilt or remorse after drinking?	Never	Less than monthly	Monthly	Weekly	Daily or almost daily	
8	How often during the last year have you been unable to remember what happened the night before because you had been drinking?	Never	Less than monthly	Monthly	Weekly	Daily or almost daily	
9	Have you or someone else been injured as a result of your drinking?	No		Yes, but not in the last year		Yes, during the last year	
10	Has a relative or friend or a doctor or another health worker been concerned about your drinking or suggested you cut down?	No		Yes, but not in the last year		Yes, during the last year	
	Sum score (possible range 0-28)						__ __
	Sum score full AUDIT (possible range 0-40)						__ __
	If full AUDIT score ≥ 8 => Apply remaining AUDIT and PHQ-2 questionnaire						

PHQ-2 Depression Screening

Over the last 2 weeks, how often have you been bothered by any of the following problems?	Not at all	Several days	More than half the days	Nearly every day
1 Little interest or pleasure in doing things	0	1	2	3
2 Feeling down, depressed, or hopeless	0	1	2	3
Sum score (possible range 0-6)		__ __		
If PHQ-2 score ≥ 3 => Apply remaining PHQ questionnaire				

PHQ-9 (remaining scale)

Over the last 2 weeks, how often have you been bothered by any of the following problems?	Not at all	Several days	More than half the days	Nearly every day
3 Trouble falling or staying asleep, or sleeping too much	0	1	2	3
4 Feeling tired or having little energy	0	1	2	3
5 Poor appetite or overeating	0	1	2	3

For peer review only - http://bmjopen.bmj.com/site/about/guidelines.xhtml

Short Tally Sheet

**6 Feeling bad about yourself or that you are a failure or have let yourself or your family down**	0	1	2	3
**7 Trouble concentrating on things, such as reading the newspaper or watching television**	0	1	2	3
**8 Moving or speaking so slowly that other people could have noticed. Or the opposite being so figety or restless that you have been moving around a lot more than usual**	0	1	2	3
**9 Thoughts that you would be better off dead, or of hurting yourself**	0	1	2	3
**Sum score (possible range 0-21)**	__ __			
**Sum score full PHQ-9 (possible range 0-27)**	__ __			

Taking record of brief advice or referral

If full AUDIT < 26 and PHQ-9 < 15:

Brief advice *(more than one answer is possible)*

- ☐ Oral Brief Advice given
- ☐ Patient Leaflet given
- ☐ Continued Monitoring
- ☐ Patient referred to other provider in practice for brief advice
- ☐ Patient referred to other provider outside practice for brief advice
- ☐ Other

____

- ☐ Time did not allow, but
  - ☐ I made follow-up appointment
- ☐ Patient declined brief advice
- ☐ Patient not screen positive, but reinforced about keeping low risk drinking habits

If full AUDIT ≥ 26 and/or PHQ-9 ≥ 15:

Patient referred to special services:

- ☐ Yes
- ☐ No

For peer review only

Long Tally Sheet

Provider details and consultation

**Practice ID** (*pre-printed*)	________________	**Provider ID / Name** (*pre-printed*)	________________
**Date consultation**	____ / ____ / ____		

Patient details

**Sex**	☐ Male<br>☐ Female<br>☐ Other	**Age**	______ years
**Socioeconomic status**	☐ Below average	☐ Average	☐ Above average
**Highest level of education**	☐ No schooling completed<br>☐ Junior high school completed<br>☐ Business/Technical training<br>☐ Doctorate degree	☐ Primary school completed<br>☐ High school completed<br>☐ Bachelor's/Master's degree	

Alcohol exposure, health literacy, and social norms

During the last 12 months have you tried to cut down on your drinking by:		
Choosing lower strength alcohol	☐ Yes	☐ No
Using smaller glasses	☐ Yes	☐ No
How easy is it to understand health information about drinking of alcohol?	☐ Always easy<br>☐ Usually easy	☐ Sometimes difficult<br>☐ Often difficult<br>☐ Always difficult
To the best of your knowledge, can drinking alcohol cause any of the following:		
High blood pressure	☐ Yes	☐ No
Liver problems	☐ Yes	☐ No
Cancer	☐ Yes	☐ No
Thinking about your friends, would you say that it is acceptable or unacceptable for them to drink:		
Regularly more than two drinks a day?	☐ Acceptable	☐ Unacceptable
More than six drinks on an occasion?	☐ Acceptable	☐ Unacceptable

AUDIT-C Alcohol Screening

Questions		0	1	2	3	4	Score
1	**How often do you have a drink containing alcohol?**	Never	Monthly or less	2-4 times per month	2-3 times per week	4+ times per week	
2	**How many units of alcohol do you drink on a typical day when you are drinking?**	1-2	3-4	5-6	7-9	10+	

Long Tally Sheet

3	**How often do you have 6 or more units on one occasion?**	Never	Less than monthly	Monthly	Weekly	Daily or almost daily	

Standard Drinks Placeholder

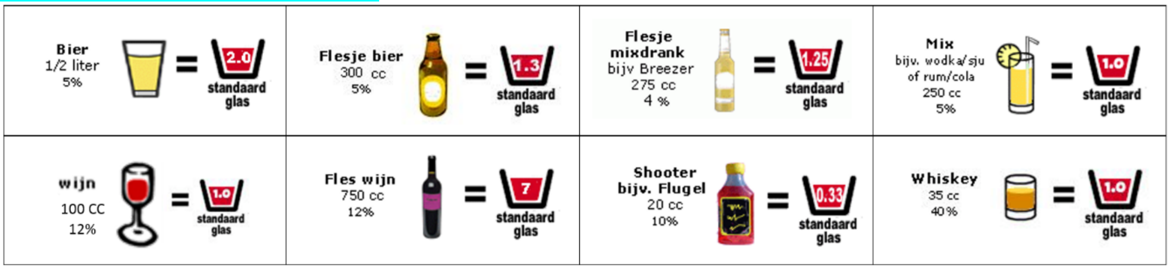

**Sum score AUDIT-C (possible range 0-12)**	__ __
**If AUDIT-C score ≥ 8 => Apply remaining AUDIT and PHQ-2 questionnaire**	

AUDIT (remaining scale)

Questions		0	1	2	3	4	Score
4	**How often during the last year have you found that you were not able to stop drinking once you had started?**	Never	Less than monthly	Monthly	Weekly	Daily or almost daily	
5	**How often during the last year have you failed to do what was normally expected from you because of drinking?**	Never	Less than monthly	Monthly	Weekly	Daily or almost daily	
6	**How often during the last year have you needed a first drink in the morning to get yourself going after a heavy drinking session?**	Never	Less than monthly	Monthly	Weekly	Daily or almost daily	
7	**How often during the last year have you had a feeling of guilt or remorse after drinking?**	Never	Less than monthly	Monthly	Weekly	Daily or almost daily	
8	**How often during the last year have you been unable to remember what happened the night before because you had been drinking?**	Never	Less than monthly	Monthly	Weekly	Daily or almost daily	
9	**Have you or someone else been injured as a result of your drinking?**	No		Yes, but not in the last year		Yes, during the last year	
10	**Has a relative or friend or a doctor or another health worker been concerned about your drinking or suggested you cut down?**	No		Yes, but not in the last year		Yes, during the last year	
	**Sum score (possible range 0-28)**						__ __
	**Sum score full AUDIT (possible range 0-40)**						

For peer review only

	__ __
**If full AUDIT score ≥ 8 => Apply remaining AUDIT and PHQ-2 questionnaire**	

PHQ-2 Depression screening

Over the last 2 weeks, how often have you been bothered by any of the following problems?	Not at all	Several days	More than half the days	Nearly every day
1 Little interest or pleasure in doing things	0	1	2	3
2 Feeling down, depressed, or hopeless	0	1	2	3
Sum score (possible range 0-6)		__ __		
**If PHQ-2 score ≥ 3 => Apply remaining PHQ questionnaire**				

PHQ-9 (remaining scale)

Over the last 2 weeks, how often have you been bothered by any of the following problems?	Not at all	Several days	More than half the days	Nearly every day
3 Trouble falling or staying asleep, or sleeping too much	0	1	2	3
4 Feeling tired or having little energy	0	1	2	3
5 Poor appetite or overeating	0	1	2	3
6 Feeling bad about yourself or that you are a failure or have let yourself or your family down	0	1	2	3
7 Trouble concentrating on things, such as reading the newspaper or watching television	0	1	2	3
8 Moving or speaking so slowly that other people could have noticed. Or the opposite being so figety or restless that you have been moving around a lot more than usual	0	1	2	3
9 Thoughts that you would be better off dead, or of hurting yourself	0	1	2	3
Sum score (possible range 0-21)	__ __			
Sum score full PHQ-9 (possible range 0-27)	__ __			

Taking record of brief advice or referral

**If full AUDIT < 26 and PHQ-9 < 15:**	
**Brief advice** *(more than one answer is possible)*	☐ Oral Brief Advice given<br>☐ Patient Leaflet given<br>☐ Continued Monitoring<br>☐ Patient referred to other provider in practice for brief advice<br>☐ Patient referred to other provider outside practice for brief advice<br>☐ Other<br>________________________<br>☐ Time did not allow, but<br>☐ I made follow-up appointment

For peer review only

Long Tally Sheet

	☐ Patient declined brief advice<br>☐ Patient not screen positive, but reinforced about keeping low risk drinking habits
**If full AUDIT ≥ 26 and/or PHQ-9 ≥ 15:**	
**Patient referred to special services:**	☐ Yes<br>☐ No

For peer review only

Tally Sheets Cover Form

Provider details, consultation and type of tally sheets
(to be filled in by local research team)

**Practice ID**	[pre-print]	**Provider ID / Name**	[pre-print]
**Consultation period**	____ / ____ / ____ - ____ / ____ / ____ (DD / MM / YY)		
**Type of tally sheets**	☐ Short tally sheets	☐ Long tally sheets	

Adult consultations
(to be filled in by PHC provider or administrator)

**Number of adult consultations during consultation period for this provider**	_ _ _ _

For peer review only

Tally Sheet Appendix

PHC provider and consultation details

**Practice ID** (*pre-printed*)	________________	**Provider ID / Name** (*pre-printed*)	________________
**Date consultation**	____ / ____ / ____		

Patient interview

**Alcohol screening result**	☐ Positive (AUDIT-C >= 8)	☐ Negative (AUDIT-C < 8)
**Asked patient for interview participation**	☐ Yes	☐ No
**Patient interested in interview participation**	☐ Yes	☐ No

Patient contact details for interview
(only if patient expressed interest in interview participation)

**Name**	____	
**Telephone number**	____	
**Address**	____	
**Preferred mode of interview**	☐ Face-to-face	☐ Telephone

Interview information

Introduction

The SCALA Study aims to find out the extent to which screening and brief advice implemented in primary health care can be increased to reduce the harmful use of alcohol. The study is taking place in cities from three countries from Latin America.
The harmful use of alcohol is prevalent in any countries, and alcohol, itself, is the seventh most important risk factor world-wide for ill-health and premature death (after high blood pressure, tobacco use, high fasting plasma glucose, high body mass index, poor diet, and low birthweight and short gestation).

Aim of the study

In this study, we aim to determine the extent of adequate prevention and management of harmful alcohol use in primary health care settings. Another major objective of this study is to improve the health of patients consulting primary health care centers.
The interview will take about 15 minutes and will cover questions on alcohol consumption, alcohol knowledge, wellbeing, and other health behavior. The same interview will be repeated twice, 3 and

For peer review only - http://bmjopen.bmj.com/site/about/guidelines.xhtml

9 months after the initial interview. Due to logistical reasons, not all patients agreed to be interviewed will eventually be asked for participation. If you have not been selected for interview participation, your contact details will be destroyed right away.

Data Handling and Sharing

Participation in this interview is entirely voluntary and you are free to skip any of the interview questions. During the interview, you will be asked questions on your personal wellbeing and health. The collected data will be entered into data bases and personal identifying information (such as name, address, and date of birth) will be replaced with an abstract personal identifier, the key to which remains with the local academic only. The data bases will be submitted to the data center at TU Dresden ('Technische Universität Dresden') in Germany using up-to-date encryption techniques. Here, all study data will be stored on encrypted hard drives and processed for further data analyses to be conducted by the study team. At all times, both analogue and digital data will be stored in secure environments. After publication of the study results, the relevant study data will be shared through the UK Data Service – a non-commercial data respository allowing other researchers to re-use the collected data for an indefinite period of time. All data shared through the UK Data Service will bear no risk of disclosure of the identity of the PHCC or of the participating providers.

Interview consent

Please check box

1. I confirm that I have read and understand the information for participating in the SCALA patient interview and have had the opportunity to ask questions. ☐

1. I consent that my contact details will be given to the SCALA study team and agree that the SCALA study team can use the contact details to ask me for interview participation and for repeating the interview. ☐

2. I understand that my participation is voluntary and that I am free to not participate, without giving any reason. ☐

3. I confirm that I have understand that study data collected through me will be processed at the TU Dresden (Germany) and shared through the UK Data Service. ☐

4.

___________________________	_________________	_____________________
Name of patient	Date	Signature

For peer review only

PATIENT INTERVIEW

Formalities

**Practice ID** *(pre-printed)*	____________	**Provider ID / Name** *(pre-printed)*	____________
**Patient ID** *(filled in by interviewer)*	____________	**Interview date**	____ / ____ / ____

Sociodemographics

**Sex**	☐ Male<br>☐ Female<br>☐ Other	**Age**	______ years
**Socioeconomic status**	☐ Below average	☐ Average	☐ Above average
**Highest level of education**	☐ No schooling completed<br>☐ Junior high school completed<br>☐ Business/Technical training<br>☐ Doctorate degree		☐ Primary school completed<br>☐ High school completed<br>☐ Bachelor's/Master's degree

Alcohol exposure, health literacy, and social norms

During the last 12 months have you tried to cut down on your drinking by:		
Choosing lower strength alcohol	☐ Yes	☐ No
Using smaller glasses	☐ Yes	☐ No
How easy is it to understand health information about drinking of alcohol?	☐ Always easy<br>☐ Usually easy	☐ Sometimes difficult<br>☐ Often difficult<br>☐ Always difficult
In the last 12 months, has any doctor or health worker asked you about how much alcohol you drink?	☐ Yes	☐ No
In the last 12 months, has any doctor or health worker advised you to reduce or stop drinking alcohol?	☐ Yes	☐ No
To the best of your knowledge, can drinking alcohol cause any of the following:		
High blood pressure	☐ Yes	☐ No
Liver problems	☐ Yes	☐ No
Cancer	☐ Yes	☐ No
Thinking about your friends, would you say that it is acceptable or unacceptable for them to drink:		
Regularly more than two drinks a day?	☐ Acceptable	☐ Unacceptable
More than six drinks on an occasion?	☐ Acceptable	☐ Unacceptable

For peer review only

AUDIT Alcohol Screening

Questions		0	1	2	3	4	Score
1	How often do you have a drink containing alcohol?	Never	Monthly or less	2-4 times per month	2-3 times per week	4+ times per week	
2	How many units of alcohol do you drink on a typical day when you are drinking?	1-2	3-4	5-6	7-9	10+	
3	How often do you have 6 or more units on one occasion?	Never	Less than monthly	Monthly	Weekly	Daily or almost daily	

Standard Drinks Placeholder

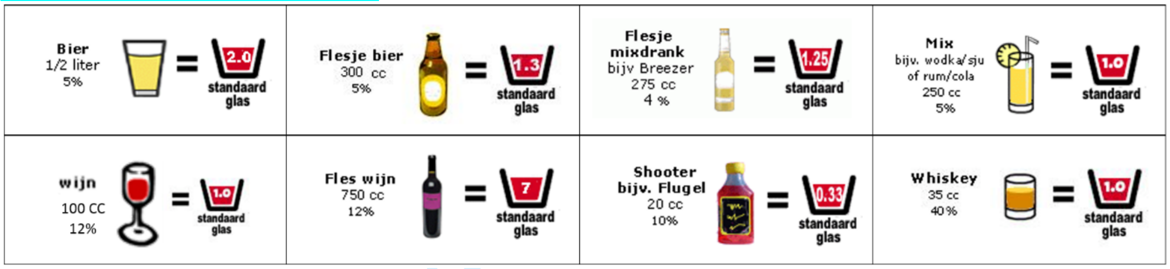

		0	1	2	3	4	Score
4	How often during the last year have you found that you were not able to stop drinking once you had started?	Never	Less than monthly	Monthly	Weekly	Daily or almost daily	
5	How often during the last year have you failed to do what was normally expected from you because of drinking?	Never	Less than monthly	Monthly	Weekly	Daily or almost daily	
6	How often during the last year have you needed a first drink in the morning to get yourself going after a heavy drinking session?	Never	Less than monthly	Monthly	Weekly	Daily or almost daily	
7	How often during the last year have you had a feeling of guilt or remorse after drinking?	Never	Less than monthly	Monthly	Weekly	Daily or almost daily	
8	How often during the last year have you been unable to remember what happened the night before because you had been drinking?	Never	Less than monthly	Monthly	Weekly	Daily or almost daily	
9	Have you or someone else been injured as a result of your drinking?	No		Yes, but not in the last year		Yes, during the last year	
10	Has a relative or friend or a doctor or another health worker been concerned about your drinking or suggested you cut down?	No		Yes, but not in the last year		Yes, during the last year	
	**Sum score AUDIT (possible range 0-40)**						__ __

For peer review only

PHQ-9 Depression Screening

Over the last 2 weeks, how often have you been bothered by any of the following problems?	Not at all	Several days	More than half the days	Nearly every day
1 Little interest or pleasure in doing things	0	1	2	3
2 Feeling down, depressed, or hopeless	0	1	2	3
3 Trouble falling or staying asleep, or sleeping too much	0	1	2	3
4 Feeling tired or having little energy	0	1	2	3
5 Poor appetite or overeating	0	1	2	3
6 Feeling bad about yourself or that you are a failure or have let yourself or your family down	0	1	2	3
7 Trouble concentrating on things, such as reading the newspaper or watching television	0	1	2	3
8 Moving or speaking so slowly that other people could have noticed. Or the opposite being so figety or restless that you have been moving around a lot more than usual	0	1	2	3
9 Thoughts that you would be better off dead, or of hurting yourself	0	1	2	3
Sum score PHQ-9 (possible range 0-27)	__ __			

Alcohol Literacy Assessment

On a scale from very difficult to very easy, how easy would you say it is to: …	Very difficult	Fairly difficult	Fairly easy	Very easy	Don't know
1 Question 1 Placeholder	0	1	2	3	5
2 Question 2 Placeholder	0	1	2	3	5
3 Question 3 Placeholder	0	1	2	3	5
4 Question 4 Placeholder	0	1	2	3	5
5 Question 5 Placeholder	0	1	2	3	5
6 Question 6 Placeholder	0	1	2	3	5
Sum score (possible range XX-XX)	__ __				

For peer review only

WHODAS 2.0 Disability Assessment

This questionnaire asks about difficulties due to health conditions. Health conditions include diseases or illnesses, other health problems that may be short or long lasting, injuries, mental or emotional problems, and problems with alcohol or drugs.

Think back over the past 30 days and answer these questions, thinking about how much difficulty you had doing the following activities. For each question, please circle only one response.

In the past 30 days, how much difficulty did you have in:					
**Questions**	None	Mild	Moderate	Severe	Extreme or cannot do
**1 Standing for long periods such as 30 minutes?**	1	2	3	4	5
**2 Taking care of your household responsibilities?**	1	2	3	4	5
**3 Learning a new task, for example, learning how to get to a new place?**	1	2	3	4	5
**4 Joining in community activities (for example, festivities, religious or other activities) in the same way as anyone else can?**	1	2	3	4	5
**5 How much have you been emotionally affected by your health problems?**	1	2	3	4	5
**6 Concentrating on doing something for ten minutes?**	1	2	3	4	5
**7 Walking a long distance such as a kilometre [*or equivalent*]?**	1	2	3	4	5
**8 Washing your whole body?**	1	2	3	4	5
**9 Getting dressed?**	1	2	3	4	5
**10 Dealing with people you do not know?**	1	2	3	4	5
**11 Maintaining a friendship?**	1	2	3	4	5
**12 Your day-to-day work?**	1	2	3	4	5
**Sum score (possible range 0-60)**	__ __				
**H1 Overall, in the past 30 days, how many days were these difficulties present?**	Record number of days: __ __ (0-30)				
**H2 In the past 30 days, for how many days were you <u>totally unable</u> to carry out your usual activities or work because of any health condition?**	Record number of days: __ __ (0-30)				
**H3 In the past 30 days, not counting the days that you were totally unable, for how many days did you <u>cut back or reduce</u> your usual activities or work because of any health condition?**	Record number of days: __ __ (0-30)				

For peer review only

Health resource utilization

Title Placeholder				
		Response 1	Response 2	Response 3
1	Question 1 Placeholder	0	1	2
2	Question 2 Placeholder	0	1	2
3	Question 3 Placeholder	0	1	2
4	Question 4 Placeholder	0	1	2
5	Question 5 Placeholder	0	1	2
6	Question 6 Placeholder	0	1	2

For peer review only

Primary Health Care Provider Questionnaire

Practice details and date

**Practice ID** (*pre-printed*)	________________	**Provider ID / Name** (*pre-printed*)	________________
**Date**	____ / ____ / ____	**Assessment**	☐ Baseline<br>☐ Follow-up 1<br>☐ Follow-up 2

Patient details

**Sex**	☐ Male<br>☐ Female<br>☐ Other	**Age**	______ years
**Profession**	☐ Doctor<br>☐ Nurse<br>☐ Psychologist		☐ Practice Assistant<br>☐ Social worker<br>☐ Other: ______

Alcohol Knowledge

	Questions	Per Day	Per Week	Per Occasion
1	**Experts recommend that everyone should limit the amount of alcohol that they drink. What is this limit for men, in terms of drinks:**	_ _ drinks	_ _ drinks	_ _ drinks
2	**Experts recommend that everyone should limit the amount of alcohol that they drink. What is this limit for women, in terms of drinks:**	_ _ drinks	_ _ drinks	_ _ drinks

		Acceptable	Unacceptable
3	**Would you say that it is acceptable or unacceptable for you to drink regularly more than two drinks a day?**		
4	**Would you say that it is acceptable or unacceptable for you to drink more than six drinks on anyone occasion?**		
5	**Would you say that it is acceptable or unacceptable for your friends to drink regularly more than two drinks a day?**		
6	**Would you say that it is acceptable or unacceptable for your friends to drink more than six drinks on anyone occasion?**		

Alcohol Health Literacy

On a scale from very difficult to very easy, how easy would you say it is to: …

		Very difficult	Fairly difficult	Fairly easy	Very easy	Don't know
1	**Question 1 Placeholder**	0	1	2	3	5
2	**Question 2 Placeholder**	0	1	2	3	5

For peer review only - http://bmjopen.bmj.com/site/about/guidelines.xhtml

Primary Health Care Provider Questionnaire

3	**Question 3 Placeholder**	0	1	2	3	5
4	**Question 4 Placeholder**	0	1	2	3	5
5	**Question 5 Placeholder**	0	1	2	3	5
6	**Question 6 Placeholder**	0	1	2	3	5
	**Sum score (possible range XX-XX)**	__ __				

The Short Alcohol and Alcohol Problems Perception Questionnaire

	**There are no right or wrong answers. Please indicate the extent to which you agree or disagree with the following statements**	**Strongly disagree**	**Quite strongly disagree**	**Disagree**	**Neither agree or disagree**	**Agree**	**Quite strongly agree**	**Strongly agree**
		**1**	**2**	**3**	**4**	**5**	**6**	**7**
1	I feel I know enough about causes of drinking problems to carry out my role when working with drinkers							
2	I feel I can appropriately advise my patients about drinking and its effects							
3	I feel I do not have much to be proud of when working with drinkers							
4	All in all, I am inclined to feel I am a failure with drinkers							
5	I want to work with drinkers							
6	Pessimism is the most realistic attitude to take towards drinkers							
7	I feel I have the right to ask patients questions about their drinking when necessary							
8	I feel that my patients believe I have the right to ask them questions about drinking when necessary							
9	In general, it is rewarding to work with drinkers							
10	In general, I like drinkers							

For peer review only

Annexe 25 Provider Interview

Telephone Interview of random sample of providers

Approximately 15-minute recorded telephone interview with open-ended questions

Country:

City:

PHCU ID Number:

PHC Provider ID Number:

Why?
Engagement: reasons for participating in the PHC action

How and for whom?
Description of the implementation process for screening and brief advice: description of proceedings and expectations of screening and brief advice

Under what circumstances?
What were the barriers and facilitators to following the guidelines on risky alcohol consumption?

What were the facilitators or barriers to implementing screening and brief advice?

Opinions and suggestions for organisational and political barriers and facilitators

Other thoughts and suggestions to speed up the implementation process

The responses will be analysed and coded according to the attached paper (Keurhorst et al. 2016).

For peer review only

Keurhorst *et al. BMC Family Practice* (2016) 17:70
DOI 10.1186/s12875-016-0461-8

BMC Family Practice

RESEARCH ARTICLE Open Access

Strategies in primary healthcare to implement early identification of risky alcohol consumption: why do they work or not? A qualitative evaluation of the ODHIN study

M. Keurhorst[1,2*], M. Heinen[1], J. Colom[3], C. Linderoth[4], U. Müssener[4], K. Okulicz-Kozaryn[5], J. Palacio-Vieira[3], L. Segura[3], F. Silfversparre[6], L. Słodownik[5], E. Sorribes[3], M. Laurant[1,7] and M. Wensing[1]

For peer review only

Abstract

Background: Screening and brief interventions (SBI) in primary healthcare are cost-effective in risky drinkers, yet they are not offered to all eligible patients. This qualitative study aimed to provide more insight into the factors and mechanisms of *why, how, for whom* and *under what circumstances* implementation strategies work or do not work in increasing SBI.

Methods: Semi-structured interviews were conducted between February and July 2014 with 40 GPs and 28 nurses in Catalonia, the Netherlands, Poland, and Sweden. Participants were purposefully selected from the European Optimising Delivery of Healthcare Interventions (ODHIN) trial. This randomised controlled trial evaluated the influence of training and support, financial reimbursement and an internet-based method of delivering advice on SBI. Amongst them were 38 providers with a high screening performance and 30 with a low screening performance from different allocation groups. Realist evaluation was combined with the Tailored Implementation for Chronic Diseases framework for identification of implementation determinants to guide the interviews and analysis. Transcripts were analysed thematically with the diagram affinity method.

Results: Training and support motivated SBI by improved knowledge, skills and prioritisation. Continuous provision, sufficient time to learn intervention techniques and to tailor to individual experienced barriers, seemed important T&S conditions. Catalan and Polish professionals perceived financial reimbursement to be an additional stimulating factor as well, as effects on SBI were smoothened by personnel levels and salary levels. Structural payment for preventive services rather than a temporary project based payment, might have increased the effects of financial reimbursement. Implementing e-BI seem to require more guidance than was delivered in ODHIN. Despite the allocation, important preconditions for SBI routine seemed frequent exposure of this topic in media and guidelines, SBI facilitating information systems, and having SBI in protocol-led care. Hence, the second order analysis revealed that the applied implementation strategies have high potential on the micro professional level and meso-organisational level, however due to influences from the macro- level such as societal and political culture the effects risks to get nullified.
(Continued on next page)

* Correspondence: myrna.keurhorst@radboudumc.nl
[1]Radboud university medical center, Radboud Institute for Health Sciences, IQ healthcare, Nijmegen, The Netherlands
[2]Saxion University of Applied Sciences, Centre for Nursing Research, Deventer/Enschede, The Netherlands
Full list of author information is available at the end of the article

BioMed Central

© 2016 The Author(s). **Open Access** This article is distributed under the terms of the Creative Commons Attribution 4.0 International License (http://creativecommons.org/licenses/by/4.0/), which permits unrestricted use, distribution, and reproduction in any medium, provided you give appropriate credit to the original author(s) and the source, provide a link to the Creative Commons license, and indicate if changes were made. The Creative Commons Public Domain Dedication waiver (http://creativecommons.org/publicdomain/zero/1.0/) applies to the data made available in this article, unless otherwise stated.

(Continued from previous page)

Conclusions: Essential determinants perceived for the implementation of SBI routines were identified, in particular for training and support and financial reimbursement. However, focusing only on the primary healthcare setting seems insufficient and a more integrated SBI culture, together with meso- and macro-focused implementation process is requested.

Trial registration: ClinicalTrials.gov. Trial identifier: NCT01501552.

Keywords: Screening and brief intervention, Alcohol prevention, Primary healthcare, Implementation, Qualitative evaluation

Background

Alcohol consumption is a substantial contributor to the global burden of disease. It is a leading factor for more than 200 diseases, injuries and other health conditions with ICD-10 codes [1]. The highest levels of alcohol consumption can be found in the European Union with approximately eleven litres alcohol per capita per year [1]. Evidence shows that 20–30 % of patients who present in primary healthcare are risky drinkers [2]. Several meta-analyses have shown that simple screening consisting of a few standardised questions, followed by a brief counselling intervention (consisting of simple advice or psychological counselling) significantly reduces alcohol consumption in primary healthcare populations [3–6]. However, there is a large gap between patients' needs and the actual provision of advice. In current European primary healthcare settings [7, 8] less than 10 % of the population at risk are identified, and less than 5 % of those who could benefit are offered screening and brief advice. Furthermore, alcohol is the least discussed lifestyle theme compared to smoking, physical activity and dietary habits in Dutch primary healthcare [9].

Barriers for screening and brief intervention (SBI) delivery by primary healthcare professionals have been identified in previous research and primarily comprised lack of knowledge in health providers; lack of adequate resources and support; and, time constrains in terms of perceived workload for SBI [10–12].

An increasing number of studies are being conducted in primary healthcare to stimulate the uptake of SBI for risky alcohol consumption (i.e. implementation strategies) [2, 13, 14], albeit with very limited success. The effectiveness of these so-called implementation strategies are summarised in several reviews [15–17]. In short, these reviews found that effectiveness of implementation programmes on SBI delivery increases when they are multi-component [15], contain higher intensity effort [16], and focus on GP's and mid-level professionals simultaneously [17]. These enablers of improvements are known as determinants of practice. The detailed process of these enablers in reaching actual uptake of SBI for risky alcohol consumption, are described in mechanisms of change [18]. More insight into determinants and actual mechanisms of change would help to tailor implementation programmes to key issues [18]. There are several qualitative studies conducted on barriers and facilitators for SBI delivery (e.g. [19–21]), although these give limited empirical insight into determinants of practice and mechanisms of change while implementing SBI in daily practice. This qualitative study was conducted after a controlled randomised trial to provide more insight into the factors and mechanisms of SBI implementation for risky alcohol consumption in primary healthcare. Linking theoretical knowledge from the implementation science database to practice-led experiences, views and attitudes from primary healthcare providers would add important knowledge on the current implementation gap. Therefore, the purpose of this qualitative study is to explore according to professionals' views on *why, how, for whom* and *under what circumstances* implementation strategies worked or did not work in increasing SBI.

Methods

Study design

We conducted a qualitative study with realist evaluation as methodological orientation after the Optimising Delivery of Healthcare Interventions (ODHIN) randomised controlled trial [22]. The ODHIN study attempted to overcome barriers for primary healthcare professional change by testing three different implementation strategies in a cluster randomised factorial trial in five European countries that represent the European alcohol levels (England, Catalonia, Sweden, Poland and the Netherlands). These countries differed in their organisation of primary care and their drinking patterns so the precise content of the implementation strategies were fine-tuned to country contexts. With regard to the lack of knowledge in healthcare professionals, we applied a training and support (T&S) implementation programme. In this programme the professionals' role security and therapeutic commitment were taken into account in order to address issues during training and support. The programme consisted of two initial 1–2 h face-to-face educational trainings, and one (10–30 min) telephone support call. With regard to lack of resources and support, we applied country-dependent financial reimbursement

For peer review only - http://bmjopen.bmj.com/site/about/guidelines.xhtml

(FR) schemes. FR concerned payment for screening and advice activities, with rates based on existing country-specific financial reimbursement for clinical preventive activities. Finally, perceived workload was addressed by an internet-based method of delivering advice (e-BI) instead of face-to-face brief interventions to save professionals' time [22]. In the trial, these strategies were tested in every possible combination and resulted consequently in eight allocation groups. The perspective of the Realist Evaluation [23, 24] is an approach that originates from educational research. The core of this approach were the 'how' and 'why' questions [23], which fitted our research question of evaluating the implementation strategies applied in the ODHIN study. From this perspective, we sought to establish what worked, for whom, in what circumstances, in what respect, to what extent, and why. Our focus thereby was on the processes by which the ODHIN trial achieved its outcomes. Its starting point was that it was not only the implementation strategy that changed professional behaviours or processes, but also the participants' reaction to the opportunities provided by the programme that triggered the change, in combination with reinforcing or hindering factors outside the programme [23].

The consolidated criteria for reporting qualitative research (COREQ-32) [25] were used to design and report the current study.

Ethics approval for the study was obtained from the relevant approval bodies within each country: In Catalonia, the Clinical Research Ethics Committee of the Jordi Gol I Gurina Primary Health Care Research Institute and from the Clinical Research Ethics Committee of Hospital Clínic de Barcelona; in Poland, Resolution No. KB- 0012/105/11 adopted by the Commission of Bioethics of the Pomeranian Medical University in Szczecin; and, in Sweden by the: Regional Ethical Review Board in Göteborg, reference number: 658/12, with approval granted for both sites in Göteborg and Linköping. In the Netherlands, the Committee on Research inv. Human Subjects (CMO) ethical board declared that no ethical approval was required in the Netherlands, reference number: 2012/281. In all four countries, all participating healthcare providers signed a written informed consent and the interviews did not place burdens on the participants.

Framework analysis

The 'Tailored Implementation for Chronic Diseases' framework (TICD) [18] was used in applying framework analysis. The TICD framework was primarily developed to implement changes in prevention and chronic disease management in primary healthcare, and is through a systematic review and consensus process based on an integrative analysis of 14 previously published frameworks, theories and models. The framework includes seven domains of implementation determinants: 1) guideline factors; 2) individual health professional factors; 3) patient factors; 4) professional interactions; 5) incentives and resources; 6) capacity for organisational change; 7) social, political and legal factors. The framework is designed to understand change of professional behaviour and organisation of practice [18] and was applied as an organising principle. Consequently, the framework was relevant in this more structured approach to qualitative data analysis, in order to build on previous body of research in barriers for implementation of evidence-based practice. Besides, it provides room to add concepts, other than already existing in the framework. This flexibility was relevant in facilitating the 'open' nature of the topic guide, which is provided below.

Participants and setting

Of the five trial countries, only England was not able to participate due to lack of funding. From the 96 participating Catalan, Swedish, Polish and Dutch primary healthcare units (PHCU), each country research team invited ODHIN participating professionals to participate to the qualitative study. The recruitment of individuals was based on purposive sampling throughout a range of maximum variation, to receive insight into why, how, for whom and under what circumstances the implementation strategies work. The sampling was based on three features:

1. occupation: GP or nurse, although in Poland only GPs were invited as no nurses participated in the trial [22]
2. screening performance after receiving implementation strategies: professionals with upper quartile versus lowest quartile of country screening rates. The screening rate was calculated as the number of completed screens divided by the total number of consultations of all patients eligible for screening.
3. implementation strategy: T&S versus no T&S. The T&S group includes professionals from 4 allocation groups: T&S alone, T&S + FR, T&S + e-BI and T&S + FR + e-BI. The non-T&S group includes professionals from the other four allocation groups: FR alone, e-BI alone, FR + e-BI, and no strategy. This sampling criterion ensured that professionals who received these different types of strategies were equally included in our study sample.

Professionals were invited by mail and by telephone. In case of non-response after email, we invited professionals directly by phone and planned the interviews.

Data collection

Interviews were performed between February and July 2014 by ODHIN trial researchers and focused on all three implementation strategies. Furthermore, field notes were made during and after the interviews. Researchers

in different countries varied somewhat in posing their questions about the three strategies. Sweden and the Netherlands pro-actively asked professionals about experiences with all three implementation strategies. Catalonia covered all three but focused on T&S, whereas Poland mainly focused on the project generally and asked for further explanation when any of the strategies was raised by the professionals themselves.

We conducted semi-structured individual interviews by telephone using interview guides and topic lists developed for this study. No other people were present at the time of the interviews, these were conducted in private rooms. Topic lists were piloted and revised according to the results of the first interviews in each of the countries. Both the realist evaluation perspective and TICD framework served as a guide in developing the topic list (the interview guide is available on request):

- Why?
  - Engagement: reasons for subscribing to the ODHIN trial
- How and for whom?
  - Description of the SBI implementation process: description of SBI proceedings and expectations
- Under what circumstances?
  - Barriers and facilitators to following the guidelines on risky alcohol consumption
  - Facilitators or barriers to implementing SBI, related to the allocation groups
  - Opinions and suggestions for organisational and political barriers and facilitators
  - Other thoughts and suggestions to speed up the implementation process

All interviews were audio taped, transcribed verbatim in each country's native language and anonymised.

Data analysis

The analysis consisted of four phases. First, each country coded independently - at least two researchers from each country independently coded fragments of the transcripts inductively and with constant discussion on interpretations, into English codes to facilitate building an international code book [26]. In this way, country researchers discussed on national and on international level their interpretation of the interviews, exchanged their views and came to an agreement for the appropriate code for the international code book. This final code book covered national as well as international interpretations, which allowed codes applied in single countries. Data collection and data analyses were alternated. Creditability was addressed by checking findings from analysis by further interviews. Furthermore, the research team included general practitioners and nurses as well. Each country used software and methods that they were familiar with, i.e. Atlas.ti version 7.1.5 (ATLAS.ti Scientific Software Development Company, GmbH, Berlin, Germany), Nvivo 10 or Microsoft Word to facilitate the coding process. Codes were structured by the seven broad TICD framework domains [18], followed by an open coding procedure, resulting in a largely inductive content analysis. When codes could not be structured by one of the seven TICD domains, they were organised in an eighth additional domain, based on appropriateness of the data.

Second, to minimise country differences in interpretations of same data, all emerging codes were classified in one Excel file code book and discussed by all researchers during face-to-face meetings, conference calls, and electronic mail correspondence. The research group agreed on the English translation of the developed codes to ensure codebook fidelity. Data collection proceeded until achievement of conceptual saturation on country level, which we defined as a state in which no new themes or codes could be generated [26]. Analyses were conducted by each country research team with the described internationally agreed format, which made it possible to perform meaningful analysis with large numbers of interviews.

Third, to maximise discussions of interpretations, exchange of views and reach of agreements, the affinity diagram method [27] was applied as an instrument in face-to-face meetings to achieve final international consensus in the research group about grouping codes and defining themes. Whereas Realist Evaluation and TICD were used as perspectives for interpretation of data, diagram affinity method was applied as an instrument to achieve consensus in analysis, as recommended in multinational qualitative research [27].

Fourth, resulting themes from the affinity diagram method were linked to the existing TICD framework domains. The general analyses were based on the themes from the third phase that had emerged nationally and internationally. To reach in-depth analyses level, the TICD concepts were not only described as domains separately, but as a second-order analysis we also explored the relations between the TICD concepts in order to catch the complexity of multinational implementation [28]. The Dutch researchers coordinated the analyses, which were subsequently evaluated and discussed by the partner researchers.

Results

Study population

Of the 138 professionals invited, 68 participated including 40 GPs and 28 nurses (mean response rate 49 %). The main reasons for not participating were lack of time and unanswered calls of the research team. Participant study and demographic characteristics were shown in Table 1. Participating professionals were mainly female with a mean age of

For peer review only - http://bmjopen.bmj.com/site/about/guidelines.xhtml

Table 1 Participating professional profiles

	Catalonia	Sweden	Poland	Netherlands	Total
N GPs	12	5	12	11	40
N nurses	10	10	0	8	28
N high performance	13	9	6	10	38
N low performance	9	6	6	9	30
N T&S	11	5	6	9	31
N no T&S	11	10	6	10	37
N FR	13	5	7	10	35
N no FR	9	10	5	9	33
N e-BI	9	6	3	11	29
N no e-BI	13	9	9	8	39
Male (%)	27	13	16	37	26
Mean age	47	52	47	44	47
Total	22	15	12	19	68

47. Catalonia needed the highest number of interviews to achieve data saturation and Poland had the lowest number of interviews, primarily because no nurses participated in the trial. Participants roughly evenly represented the three purposive sampling domains of occupation, screening performances and implementation strategy.

Barriers and facilitators to implementation

Table 2 links already existing theoretical TICD concepts with practice-led affinity diagram themes that rose from the data analyses. In more detail, there are seven TICD domains [18] that included 39 relevant concepts in light of our findings, being reflected in the two left-hand columns of the table. The two right-hand columns include 57 affinity diagram themes that derived from the grouped coded data. Thereby, this table links theory and practice and consequently gives insight into important determinants for practice within this population of health professionals. An eighth additional concept was added that did not fit within the original TICD framework and was related to 'Implementation strategy practicalities'.

As presented in Table 2, most affinity diagram extracted themes fit the 'individual factors' TICD domain. Also, the TICD domains 'professional interactions' and 'incentives and resources' were important in gaining insight into the mechanisms behind the allocations. The importance of the TICD domains 'guideline factors', 'patient factors', 'capacity for organisational change' and 'social, political and legal factors' in explaining the processes of the allocations, varied per allocation. High as well as low performer views equally covered the TICD domains, whereas GPs and nurses differed in covering TICD domains. GPs held clearer views than nurses on healthcare system barriers and facilitators, which resulted in the TICD domains 'capacity for organisational change' and 'social, political and legal factors' being mainly covered from the viewpoint of GPs.

Why?

Many professionals, both high and low screening performers and both nurses and GPs, had a positive role perception with regard to conducting SBI. Most professionals participated because of their awareness of the prevalence of alcohol-related problems and the willingness to contribute to the prevention of risky drinking. For most professionals also the likelihood of being allocated to T&S was an important motive for participation.

> *Alcohol problems are really big in this area. I've been observing them for years.(GP, FR, low performance, PL)*

Polish and Catalan GPs reported the additional value of FR besides their willingness to contribute to the prevention of risky drinking. Dutch and Swedish GPs as well as some Catalan nurses reported not being motivated to participate for a financial reimbursement, whereas Polish and Catalan GPs felt positive about providing good care and getting paid for it as well.

There were no professionals who mentioned any e-BI related motivation to participate in the trial. Most professionals, GPs as well as nurses, were ambivalent in their attitude towards e-health. The professionals who were positive about the e-BI concept primarily thought it was useful in information provision for patients.

How and for whom?

Aspects in three TICD domains appeared to be relevant in answering the question *how* and *for whom* T&S worked: *guideline factors*, *individual factors* and factors related to *incentives and resources*. Facilitating T&S ingredients for high SBI performance can be summarised into knowledge gained, application of tools, support offered by the trainer, and team-based education. Professionals who received training and support indicated factors that would make training and support even more effective, i.e. continuous training provision, more time to learn intervention techniques and more tailoring to experienced barriers, such as a perceived lack of time for conducting SBI. In Catalonia, Sweden and the Netherlands, training and support further raised awareness of the guidelines and stimulated many of the professionals to keep using them. Primarily for high performing GPs, training and support provided assistance in SBI application in daily practice. Most of the training and support allocated professionals perceived the guidelines to be feasible and compatible with daily practice. Most professionals in the ODHIN study wanted to know and to become skilled in *how* to implement and

For peer review only - http://bmjopen.bmj.com/site/about/guidelines.xhtml

Table 2 TICD domains and concepts linked to Affinity Diagram themes and codes

TICD Domain	Theory-led TICD concepts	• Empirically-led Affinity Diagram themes	Codes
1. Guideline factors	Cultural appropriateness	• Cultural appropriateness	SBI is not a task for PHCU_referall to specialised care outside the PHCU; no guideline available_SBI too late
	Strength of recommendation	• Barriers to adhere to the guideline	Too strict_nr of drinks; SBI does not fit in short time consult; doubts about effectiveness pro-active screening
	Compatibility	• Adherence TO guideline<br>• Routine<br>• Follow-up of SBI	Return to the habitual system; routine_Application of the screening in all cases; already a routine; routine_preventive activities; SBI part of the nurse's protocol; SBI part of GP's protocol/routine; follow-up after SBI suboptimal; policies_screening during initial general interview with every new patient; focus on alcohol addicted patients/co-addicts; focus on chronically ill patients; routine_follow-up of patients; repeat SBI
	Observability	• Facilitators to adhere to the guideline	Partly adherence to guideline; adherence to guideline; clear cut-off screening tool stimulates brief intervention; use evidence based knowledge/material; use evidence based knowledge/material – mi; adherence implementation takes a while; adherence_Initial difficulties; adherence_Simple adaptation process; interventions were feasible; feasibility_ of the instrument
	Feasibility	• Adherence to guideline<br>• Facilitators to adhere to the guideline<br>• Implementation of guidelines<br>• Feasible guidelines	Example of interventions
2. Individual factors	Agreement with recommendation	• Evaluating own performance<br>• Implementing new practice<br>• Role perception<br>• Screening opportunities<br>• Barriers	Screen to make patients aware of daily drinking habit; role perception_patient motivated when given BI from a GP; performance perception_effects of SBI; performance perception_no effects of SBI; my role to start the process; role perception SBI; barrier screening_perceived_not relevant in context; role perception_to recognise signs given by a patient; it's not my role; agreement recommendation; awareness _alcohol is not a medical problem
	Expected outcomes	• Personal motivation to participate from societal perspective<br>• Collaboration from individual perspective<br>• Evaluating own performance<br>• Role perception<br>• Professional's expectations<br>• I don't care<br>• Barriers	ODHIN outcome expectation_to catch more case positives; role perception_patients like GPs to ask about lifestyle; expectation_patient's reaction; expectation_conformed to expectations; professional age; motivation to participate ODHIN_curiosity about the outcomes; expected MI intervention outcome_high; expected intervention outcome_low; expectation_With no initial expectations; lack of motivation to change; barriers referral_big step; GP afraid of patient's reaction
	Emotions	• Implementing new practice<br>• Barriers	E-health_using e-health is a personal weakness; new patient; hard to screen GP's own friends or acquaintances
	Frustration	• Implementing new practice	ODHIN impact_more frustration
	Intention and motivation	• Personal motivation to participate from societal perspective<br>• Training<br>• Collaboration from individual perspective<br>• E-health<br>• Personal motivation individual perspective<br>• I don't care<br>• Barriers	Motivation to participate in ODHIN_to help patients; ODHIN training_positive but not fully attended; Motivation to participate ODHIN_motivation for intervention; motivation to participate ODHIN_the size of alcohol problem; motivation to participate ODHIN_easier with a network; e-health_positive in e-health; e-health_barrier referral; e-health_no time to become familiar with e-health intervention; e-health_not familiair with website content; e-health_negative

For peer review only

Table 2 TICD domains and concepts linked to Affinity Diagram themes and codes *(Continued)*

			attitude; motivation to participate ODHIN_consider load and benefit; not motivated by financial incentives; motivation to participate ODHIN_to act pro-socially; motivation to participate ODHIN_personal interest/benefit; motivated by ODHIN financial incentives; motivation to participate ODHIN_negative; motivation to participate ODHIN_ Interesting subject; not motivated to improve SBI; low patient awareness_inhibits professional; low motivation to change_inhibits professional; motivation to change_motivates BI; patient reactions_denial inhibits brief intervention;
	Learning style	• Training<br>• Implementing new practice<br>• Routines	ODHIN training_increases awareness of the problem; ODHIN training_temporary stimulation; ODHIN training_positive; ODHIN presence cause reminders/awareness_temporary; continuous triggers necessary for SBI; routine and practice
	Self-efficacy	• Self-efficacy	Self-efficacy in BI_high; high screening self-efficacy; self-efficacy; self-efficacy_frustration; self-efficacy in BI_moderate; performance perception_GP can always do something
	Awareness and familiarity with the recommendation	• Personal motivation to participate from societal perspective	ODHIN motivates to screen pro-active; awareness of alcohol problems; importance of screening
	Knowledge	• Training<br>• Implementing new practice<br>• I don't care<br>• Barriers<br>• Screening opportunities	Skills thank to previous training; ODHIN impact_encouragement to introduce more prevention; previous training_don't remember; barrier screening_language barrier; barrier screening_information from system not available; barrier BI_skills; Skills_plurimedication; Patient nightlife related with drugs/alcohol; patient known to drink too much; screen because of patient signals; skills_professional knows well patient's medical history; importance_associated pathology; screened patients suspected of drinking alcohol; patient drunk during the visit; problem reported by family member
	Knowledge about own practice	• Collaboration from individual perspective<br>• I don't care<br>• Barriers	Barrier screening_already SBI by colleague; barrier screening_other important health and other topics; barrier screening_sociodemographics; patient religious issues
	Skills needed to adhere	• Implementing new practice<br>• Personal motivation individual perspective<br>• Professional patient approach<br>• Professional's expectations<br>• Barriers<br>• Screening opportunities	ODHIN impact_new skills/procedures; motivation to participate ODHIN_need for more knowledge and skills; expectation_increase knowledge/skills about interventions; skills_no judgemental attitude/tolerance; skills_professional keeps motivating the patient; skills_individual approach to patient; alcohol is a sensitive issue/difficult subject; need for more knowledge & skills for SBI; performance perception_screening justified by the research project
	Capacity to plan change	• Personal motivation to participate from societal perspective<br>• Implementing new practice	Barrier screening_economic crisis situation; ODHIN impact_introduction of new data into patients' records
	Nature of the behaviour	• Implementing new practice	ODHIN impact_effort to perform
	Self monitoring or feedback	• Personal motivation to participate from societal perspective<br>• evaluating own performance<br>• implementing new practice<br>• screening opportunities<br>• I don't care<br>• Barriers	ODHIN outcome _catching patients in early stage of disease and follow-up; motivation to participate ODHIN_awareness of trivialising; satisfaction with own performance; lack of satisfaction with own performance; self-monitoring of screening; self monitoring of BI; insight SBI potential afterwards; ODHIN impact_more patient/new groups of patients screened; ODHIN presence cause reminders/awareness_own consumption behaviour; ODHIN presence cause reminders/awareness; ODHIN did not make any

For peer review only

Table 2 TICD domains and concepts linked to Affinity Diagram themes and codes *(Continued)*

			difference; ODHIN presence did not cause reflection on own consumption behavior; barrier screening_ simply forgotten; has routine; barrier screening_ experienced workload; Patient age; patient gender; physical GP's tiredess; Screened every patient (or tried to screen)
3. Patient factors	Patient behaviour	• Patient reactions	Patient reactions; feel suspected of being a drinker; afraid/suspicies; stressed/tense; not honest; honest; frustration; defensive; surprise; relief; no objection/ acceptance; negotion/trivialisation
	Patient beliefs and knowledge	• perceived patient awareness<br>• lack of interest in E-BI	Awareness_personal decision of the patients; awareness_self-control of drinking; patient reactions_awareness guidelines; BI_difficult when patients not aware; patient reactions_don't treat beer as alcohol; self-efficacy in BI_low/doubts if patiens will change anything; patient reactions_lack of interest e-health; patients not interested in e-BI
	Patient motivation	• Patient trust required<br>• Motivation to change	SBI requires patient's trust; motivation to change_Serious alcohol problem; motivation to change_Social support
	Patient preferences	• Patient reactions	Patient reactions_positive
4. Professional interactions	Communication and influence	• Decision to participate<br>• General assessment of PHCU routines and engagement	Decision to participate in ODHIN_agreement; decision to participate in ODHIN_GP decided to participate; decision to participate in ODHIN_nurses agreed; decision to participate in ODHIN_practice nurses not involved; motivation to participate ODHIN_order or influence of other professional/supervisor/colleague, etc.; GP takes the lead in ODHIN SBI; engaged other staff in alcohol discussions than those involved in the Odhin project; team (not) on the same line; different routines among the staff
	Referral processes	• Barriers<br>• Task division in the team<br>• Referral	Addiction care disappointing; GP internal referral to specialised professional; nurse referral to other(s); ODHIN initiates referral option specialised nurse; GP referral to addiction care; need for low barrier referral possibilities; conditions in the PHCU_additional support
	Team processes	• Barriers<br>• Organisation of SBI care<br>• Task division in the team<br>• Learning from each other<br>• Making agreement within the practice	Recent screening; colleagues less practice/ experience; organise care multidisciplinary; counseling done by other profession; care requires a specialized practice nurse; team process SBI_SBI only partly by nurse; unknown patient; practice nurses_have more time_for MI; other professionals have more time'; practice SBI in team; share experiences; lack of communication; sufficient communication; nurse not informed about procedures; agree on team objectives; agree on SBI strategy
	Undefined	• Difference in opinions	ODHIN_waisted money
5. Incentives and resources	Availability of necessary resources	• Physical working conditions in the PHCU<br>• Difference in opinions<br>• Tools as facilitators<br>• Screening tool usefulness<br>• Trigger for screening<br>• Importance of time	Conditions in the PHCU_privacy; conditions in the PHCU_disturbances; ODHIN did not lack resources; little bureaucracy; ODHIN provides tool for BI; need for patient information_low barrier patient information; more resources in the treatment of the patient; screening instrument not within reach; advice_use available training and tools; screening tool helps to structure; advice_use screening tool; ODHIN provides screening tool; screening instrument_Suitable instrument; screening tool did not help; screening instrument_too complicated for patients; screening instrument_easy to use; screening instrument_anonymous; visible screening instrument does not stimulate; visible

For peer review only

Table 2 TICD domains and concepts linked to Affinity Diagram themes and codes *(Continued)*

			screening instrument stimulates; need for summary card on desk; advice_time is necessary resource; GPs want more time per patient; increase available time for extra practice nurses; time pressure inhibits BI; time pressure inhibits GP's MI; time pressure inhibits screening; time is no barrier to screen; time is no barrier for advice; addicted patients need more time; time for creating right atmosphere; time pressure forces need for follow-up appointment
	Continuing education system	• Importance of training	Advice_continuous training; training should be organised in PHCU; more role playing; Providing training tools suitable for professionals
	Financial incentives and disincentives	• Importance of finances	No financial resources from health Insurance; finances required for practice nurse; financial incentives rewards your effort; financial incentives would create more priorities; more funds needed
	Information system	• Role in information system	Usual registration in information system; information system obligatory field; no use of information system; register SBI in information system; information system not adapted to SBI; information system not obligatory field
6. Capacity for organisational change	Assistance for organisational changes	• PHCU SBI policy<br>• Nurses protocol for SBI	Advice_invite a consulent; practice nurse not skilled
	Monitoring and feedback	• PHCU SBI policy	Need for ongoing evaluations
	Priority of necessary changes	• PHCU SBI POLICY	Advice_SBI prioritarisation
	Regulations, rules, policies	• Systematisation of SBI<br>• PHCU SBI policy<br>• Nurses protocol for SBI	Policies_need for a systematic approach to disease prevention; make it part of protocol; make it part of performance indicators; Nurses protocol adapted in line with ODHIN
7. Social, political, legal factors	Economic constraints on the healthcare budget	• Increase public awareness	Advice for improving public health_society should be richer
	Influential people	• Importance of regional policy<br>• Increase public awareness<br>• Awareness of prevention task of primary care	The board plays an important role; advice_increase public awareness (media); advice_increase public awareness (media)_broad lifestyle; advice_increase public awareness (media)_involve environment; advice_increase school and parent awareness; little effect public campaigns; synergy effect of advice from multiple people; less ads; change social attitudes; advice_increase primary care awareness outside PHCU; increase awareness in professionals; prevention task of PHCU
	Legislation	• Need for effective policy actions<br>• More strict legislation	Mandatory trainings for GPs'; advice_increase alcohol taxes_not effective; advice_increase alcohol taxes; advice_legislate higher age buying alcohol; advice_make alcohol less available; fear of bureaucracy
	Payer or funder policies	• Increase public awareness	Advice for improving public health_don't waist public money on projects like ODHIN
	Undefined	• Increase public awareness<br>• need for effective policy actions<br>• awareness of prevention task of primary care	Advice for improving public health_use disulfiram implants; advice for improving public health_state alcohol policy is schizophrenic; raise awareness of screening, BI and available tools; build trust between GPs and patients; advice_organise peer buddy's; increase knowledge in primary care professionals; Approach general/integral; policies_screening during initial general interview with every new patient; introduce more programs like ODHIN
8. Implementation strategy practicalities	Training and support		Caused awareness; MI requires long term practice; MI useful for other lifestyle issues; positive;

For peer review only - http://bmjopen.bmj.com/site/about/guidelines.xhtml

Table 2 TICD domains and concepts linked to Affinity Diagram themes and codes *(Continued)*

		preference for more factual knowledge; role playing_not favorable; temporary stimulus
	Financial reimbursement	No effect; extra motivation
	E-health	low outcome expectation; low patient motivation inhibits professional; easily accessible intervention; increases awareness; negative attitude; no time to become familiar with e-health; not applicable for elderly; not applicable for low SES; no effect

conduct SBI rather than be convinced of the *importance* of implementing:

> *"During my education there was some attention paid to motivational interviewing, but this training was very welcome as it cleared things up, such as fine-tuning my patient approach to their phase of behaviour change according to the behaviour change matrix."* *(Nurse, T&S, high performance, NL)*

High performing professionals reported that they gave more priority to SBI in their routines than before ODHIN. After attending training and support sessions, professionals felt that it was not only a matter of having time, it was also a matter of prioritising. They found that it was actually possible to frequently ask patients about alcohol consumption, even during high workload:

> *"The more often you ask the questions, it will become more of a routine, it takes time to incorporate new procedures and ask the questions, but most of the time you can ask these questions during each visit"* *(Nurse, T&S, high performance, SWE)*

> *"You have to decide beforehand whether you want to reserve time for this. Do we think it's important enough to spend time on?" (GP, T&S + FR + e-BI, high performance, NL)*

Furthermore, learning how to raise the 'alcohol topic' in patient groups with varying motivation to change was appreciated in the training and support sessions. Some high performing professionals used study participation to start the conversation and to make the topic more easily accessible:

> *"I stated: "We are taking part in a project aimed at people's wellbeing"" (GP, T&S + FR, high performance, PL)*

The high performing professionals who attended training and support, reported being stimulated in discussing SBI experiences within their team. This facilitated a team approach in doing SBI:

> *"We could have talked about this without the ODHIN project. But it gave us a reason to sit down and do so." (GP, T&S, high performance, SWE)*

Furthermore, many professionals already knew about the existence of SBI tools. Even so, they were additionally informed during T&S where to find the right tools and how to apply them appropriately.

However, both low and high performing professionals reported that training and support were felt to be a temporary stimulus, and that alcohol is just one of the many important themes to discuss. Embedding SBI in the long term requires a continuous trigger, such as booster sessions. This also facilitates prioritising:

> *"The emphasis on your work is on what you are currently busy with. It would be the same if I had participated in a study about cardiovascular diseases." (GP, T&S + FR + e-BI, high performance, NL)*

TICD domains *individual factors*, factors related to *incentives and resources* and *social, political and legal factors* were of relevance in evaluating *how* and *for whom* a financial reimbursement strategy would work. Financial reimbursement seemed to differ in impact between Poland and Catalonia compared to Sweden and the Netherlands, mainly due to low personnel levels and salary levels.

> *"Because with the cutbacks there are fewer of us and we have to…stand in for people and that's hard, isn't it?" (GP, T&S + FR, high performance, CAT)*

> *"Getting an incentive is always good. If this is financial or economic, I think it could be good, but I am not completely sure about it. When you get invited to participate in a study they ask you "Do you want to participate?" and you take part voluntarily. In the end, it turns out that someone publishes an article and your name is there, that's okay. Of course both the financial and professional incentives are important, but with the financial one you feel they treated you well." (Nurse, T&S + FR, high performance, CAT)*

For peer review only - http://bmjopen.bmj.com/site/about/guidelines.xhtml

Views of Swedish and Dutch professionals allocated to financial reimbursement did not differ between high and low performers and those not being allocated to financial reimbursement. Swedish and Dutch professionals thought it was important to get paid for the care provided, but they perceived it as inferior to being a good care provider:

> *"Now it is the diagnosis that brings in money, nothing out of this really benefits the patients, but that's something for financially educated managers to calculate and put in charts and to perform some kind of statistics. What is important in healthcare is the patient." (GP, T&S, low performance, SWE)*

Furthermore, in the ODHIN study the financial reimbursement scheme differed per country. In Poland and Catalonia, professionals were reimbursed directly, whereas in Sweden and the Netherlands reimbursement was applied on PHCU level. In Sweden and the Netherlands, professionals reported that financial resources in principle were of high importance. However, both high and low performers from these countries preferred being structurally paid for their preventive services by health insurance, rather than a temporary project based payment. They considered increased resources from health insurances required for long-term improvement of SBI:

> *"I have to pay my practice nurse. If I can only pay her for other tasks [other than asking for alcohol consumption], I have to pay for it myself when she is going to ask about alcohol consumption." (GP, T&S + e-BI, low performance, NL)*

It turned out that for all four countries patients' lack of interest inhibited both nurses and GPs from being active in referring patients to e-BI. It neither facilitated nor guided them in providing brief interventions, as patient reactions were frequently not very promising. Therefore, face-to-face interventions were the preferred method in such cases. Consequently, the high performers did not give any e-BI related explanations for their performance levels, whereas the low performers explained the non-facilitating role of e-BI.

> *"Well, I gave them the e-BI tool and asked them to access it. However, it is up to them, you can ask them to do it, but they don't always do so. It happens very often, your role as a professional is to say 'look, if you want more information here it is' but in my opinion this is a challenging thing." (Nurse, T&S + FR + e-BI, high performance, CAT)*

> *"If they didn't have a computer at home, or if they did not feel comfortable using one – then it was really not any use to recommend it to them. It was meant for those who felt that they wanted it... I don't know if they visited the website or not.. I have no idea..." (Nurse, e-BI, low performance, SWE)*

Under what circumstances?

The fact that many health professionals throughout the four countries participated in a trial concerning preventive services for risky alcohol consumption, raised their awareness and frequency of providing these services. That means that just putting this theme as item on the agenda already makes the professional more active in SBI, irrespective of their allocation. This was illustrated by a professional who had not received any of the implementation strategies but was still a high performer.

> *"I know that before ODHIN I did not pay as much attention to this as after ODHIN. I did not have specific barriers for asking about alcohol consumption, but if you participate in this kind of project it will become more part of your automatism in anamnesis." (GP, no strategy, high performance, NL)*

Consequently, before being able to receive a state of SBI routine, one should be increasingly aware of their SBI activities. Referral opportunities could provide stimulating thoughts for professionals to take up this activity. Another important precondition to make it part of a routine, is to include it in protocols and to set reminders.

> *"Include it in your protocol. Every time you see it [on your screen], you will be reminded." (Nurse, T&S + FR + e-BI, high performance, NL)*

However, there are some preconditions that can facilitate or hinder successful a implementation of brief interventions, such as information systems. As countries differed in their information systems, the role of the information system as a facilitator varied.

> *"Yes... it has facilitated our work a lot because we already had it implemented in our computerised medical record (E -CAP)... and ... and this is the usual computerised tool that we always use, as a result of this it has been much easier." (GP, T&S + FR + e-BI, High performance, CAT)*

> *"I do register, but it's a bit difficult as we do not have an appropriate ICPC [declaration] code" (GP, FR + e-BI, low performance, NL)*

> Subsequently, professionals frequently reported high workloads, which caused T&S not to be sufficient to increase performance.

For peer review only - http://bmjopen.bmj.com/site/about/guidelines.xhtml

There are not enough GPs … more time and more funds should be reserved … e.g. one extra hour per week for preventive visits should be founded by the National Health Fund (GP, T&S, low performance, PL)

Another inhibiting factor was that the alcohol subject seemed to compete with other lifestyle prevention themes. For example alcohol received less media attention compared to other lifestyle prevention themes:

"For professionals, you have to notice it more, read about it more, pay more attention to it in the media and literature. (…) The lobby for quitting smoking is much bigger than the lobby for drinking less." (GP, FR + e-BI, low performance, NL)

Second-order analysis: relations between framework domains

Many drivers for the trialled SBI implementation strategies were found in the TICD domains 'Individual health professional factors' and 'incentives and resources'. However, these were embedded in other TICD domains to influence SBI implementation in daily practice. In particular, political culture – part of 'social, political and legal factors' domain – is such an important contextual factor that exert the SBI implementation in daily practice. To create an environmental SBI culture, a facilitating political and social culture is essential:

"The state earns most on alcohol and tobacco. So limiting consumption is against its economic interests." (GP, T&S + FR, high performance, PL)

"There is a social acceptance for drinking." (GP, T&S + FR, high performance, PL)

Furthermore, the organisational environment challenges the SBI implementation, even when implementation strategies seem to work at the individual level i.e.:

"The system of work should be changed. Besides alcohol interventions, interventions on nicotine, obesity, physical activity should be conducted. And I have 10–15 minutes per patient."*(GP, no strategy, low performance, PL)*

"I do register [SBI], but it's a bit difficult as we do not have a good ICPC code [for health insurance declaration]." (GP, T&S + FR + e-BI, high performance, NL)

Implicitly, responses of both nurses and GPs show their perceived responsibility in SBI, yet as part of the SBI responsibility as society together. Despite their intrinsic motivation to prevent patients from alcohol-related disabilities, GPs and nurses feel more rationale for selective screening rather than opportunistic screening:

"When there are analytical alterations or when there's a sonogram that shows something, when there's a pathology behind it (…), it's easier to focus on it." (nurse, FR, low performance, CAT)

These insights taken cumulatively, it seems that implementation strategies should be applied in other healthcare settings as well, next to primary healthcare. The ODHIN study tested implementation strategies at micro-level and meso-level. Implementation determinants on the macro-level as described by TICD domains seemed to challenge the tested implementation strategy influences. Therefore it raises the need for an integrative SBI approach to take broader than primary healthcare.

Discussion

The aim of this study was to explore, according to professionals' opinions, *why, how, for whom* and *under what circumstances* the implementation strategies tested in ODHIN increased or did not increase SBI. T&S improved knowledge and skills in team-based approach and taught professionals to prioritise SBI. Continuous provision, sufficient time for learning intervention techniques and tailoring to individual experienced barriers, were important perceived facilitators. Catalan and Polish professionals perceived financial reimbursement as an additional stimulating factor, as SBI rates were smoothened by personnel levels and salary levels. Structural payment for preventive services, rather than a temporary project based payment, might have further increased the SBI rates. Implementing e-BI seem to require more guidance than was delivered in ODHIN, for example in connection with unmotivated patients. Other preconditions for SBI in routine care, irrespective of the allocation, are frequent exposure of this topic in media and guidelines; information systems that facilitate SBI (e.g. screening programmes); and having SBI in protocol-led care. However, despite having identified facilitating factors on the micro-individual level, the macro-level in which SBI is augmented to be implemented includes important barriers. These were mainly related to politics and social culture.

The purposive sampling strategy in this study was based on occupation, implementation strategy and screening performances. This qualitative study showed that allocation to T&S or FR influenced professionals' views, whereas e-BI did not seem to make any difference. Occupation did not seem to influence views, perceptions and opinions, although GPs reported higher

For peer review only - http://bmjopen.bmj.com/site/about/guidelines.xhtml

importance of financial resources and experienced barriers in implementing routine SBI. Furthermore, GPs had clearer views on the barriers and facilitators of the healthcare system, which we perceive a result of different tasks and functions by professionals in the organisation of primary healthcare. Tailored strategies seem important, also with regard to who makes decisions and who is financially responsible. Furthermore, despite positive SBI outcomes after T&S and FR during high workloads, time constraints remained. This indicated the need for more profound changes in the structure of the healthcare organisation to facilitate further SBI improvements in primary healthcare.

In line with the literature, our study confirms that very few professionals used e-health in patient care [29, 30]. An important barrier for implementing e-BI was that professionals from all countries were mixed in their trust in e-BI in principle and they noticed that their patient population was not interested in e-BI. Despite the effectiveness of SBI self-help via internet in principle [31], our findings imply that more efforts might be required in getting the facilitated e-BI access embedded into daily primary healthcare practice. For example, professionals seem to require clearer guidance in how the facilitated access can decrease their workload by using e-BI interventions that have proved to be effective [29, 32]. In the ODHIN programme offering e-BI might have been too much a matter of being 'dropped' as a strategy rather than personal guidance in using it with a population who is less familiar with the internet, such as the elderly or in a population with a low motivation to change alcohol consumption, as experienced during ODHIN.

When implementing lifestyle interventions such as alcohol related screening and brief interventions, it is important to address sustainable funding of services [33]. In the United Kingdom (UK), the Quality and Outcomes Framework (QOF) is a reimbursement scheme in which payment is based on fee-for-service and capitation systems rather than related to quality of care [34]. After 20 systematic reviews and one systematic reviews of systematic reviews [35], it is clear that pay for performance can be effective. However, policy makers should be warned that effects may be only realised on short-term and may be not as large as one may wish [35]. Pay for performance has potential, but it is not a "magic bullet". To achieve sustainable changes, it needs to be combined with other quality improvement initiations [35].

Of the total 57 concepts included in the seven domain TICD checklist framework [23], 39 concepts were covered in this study. Non-covered concepts were mainly associated with topics not relevant in the study context, such as corruption or political stability. For Poland specifically, it is no surprise that guideline topics were hardly covered, as no official guidelines exist. Furthermore, one can imagine that healthcare professionals talk more easily about their daily practice than about topics that are more general and policy-related, such as topics with *social, political and legal factors*. These topics were more indirectly covered in the second-order analysis. Other professional disciplines such as managers and policy makers could add on the more meso- and macro-perspective. In addition, more context-related items should receive attention– e.g. Poland mainly has solo-practitioners (GPs) who are not able to refer to other providers in the practice, or differences in country-specific guidelines to adapt SBI procedures.

Only four themes identified in the analysis did not match with the TICD checklist. These four were either very specific, such as opinions regarding specific medicaments, or very generally formulated, such as with increasing public awareness. However, these were of minor importance in answering the research question.

There are caveats as well as strengths to mention. The interview questions about allocation experiences and views varied across participating countries. Sweden and the Netherlands pro-actively asked professionals about their experiences with all three implementation strategies. Catalonia covered all three but focused on T&S, whereas Poland mainly focused on the project generally and asked for further explanation when any of the strategies was raised by the professionals themselves. Despite this systematic difference, there were minor differences in FR and e-BI data saturation due to the equally represented allocations. The e-BI coverage in the results section is less compared to FR and T&S. Despite reaching data-saturation, the participating professionals did not share much e-BI related data. Consequently, this data limitation impedes to provide full answer on the research questions related to e-BI and therefore deserves further research. Another caveat is the selection of professionals who are likely to be more motivated to prevent alcohol problems, compared to the greater primary healthcare professional population. This could make the implementation strategies less powerful, and it could make the conditional circumstances described of greater importance.

A strength of the study was the use of different country contexts when striving after code homogenisation of emerging themes in light of the Realist Evaluation built international code book. The Realist Evaluation then helped to distinguish between a context and a mechanism [36]. For instance, there were differences in the state of the art regarding SBI implementation. Catalan, Swedish and Dutch professionals already paid (some) attention to lifestyle prevention themes including alcohol, while many Polish professionals did not pay any attention to alcohol SBI before participating in ODHIN, which is in line with the absence of a Polish national guideline. Other examples are differences in countries' cutbacks in personnel and salaries,

For peer review only

policies and social progress towards SBI implementation differed, which made comparisons sometimes difficult. To increase meaningful analysis of the data on international level, we organised face-to-face discussions and conference calls to agree on scientific value of our findings over all four countries. In addition, a major strength of the study is that the approach of the realist evaluation was combined with the TICD framework analysis. The Realist Evaluation perspective was developed to unpack the 'how' and 'why' questions and illuminate the many, varied and interdependent, mechanisms by which interventions may work (or fail to work) in different contexts in education [23, 24]. This makes sense with regard to implementation programmes, as these are often complex and multifaceted [28, 37] and enabled the second-order analysis [28]. The interpretative approach of the realist evaluation [24] was considered to be appropriate in evaluating not only why our implementation strategies worked or did not work, but also in which type of context and in which situation. Another strength is that this is the first qualitative study evaluating implementation strategies with regard to SBI, next to numerous qualitative studies on this topic as presented in a review of Johnson et al. [21].

An issue that deserves consideration is the sustainability of the implementation efforts. Future implementation programmes should provide booster training sessions to update knowledge, to set alcohol SBI on the agenda, to maintain SBI skills and institutional support. Also when the professional team formation changes, booster session could be important to reformulate different professional roles within teams. Second, structural payment for preventive services, rather than a temporary project based payment, is important for both short term and for long term. More importantly, implementation strategies on the macro level should be applied to influence the societal and political culture. Only then, initiatives on the micro and meso-level can be highly successful. Successful e-BI strategies deserve further research attention, as the limited e-BI related data in this study impedes to provide full answer on the research questions related to e-BI.

We believe that the present study considerably advanced our understanding of alcohol SBI implementation processes in different contexts. A review of Chaudoir et al. [38] indicated that organisation, professional and innovation-level constructs have the most usable measures for implementing health innovations, whereas structural and patient-level constructs have the least usable measures [38]. Implementing guidelines like alcohol SBI, can be regarded as a 'health innovation'. When we compare the review results of Chaudoir et al. with the results from the present study, we found that most findings were in agreement with the indicated measures. Factors related to guidelines, individual professionals, incentives and resources as well as a capacity for organisational change were most important in reaching the aim of this study. This study adds the importance of meso- and macro-influences when implementing potentially powerful SBI drivers.

Conclusions

To summarise, T&S essential implementation ingredients seemed to be gained knowledge and skills, team-based training and learning to prioritise SBI during high workloads. FR directed SBI motivations appeared to be highly determined by country context and were influenced by the way reimbursement was provided and by the reimbursing parties. Structural payment is an important precondition. Despite e-BI proved effectiveness in previous lifestyle studies [31], this study showed that professionals require clear guidance in how the facilitated access can improve SBI in routine practice. To give a complete answer on the e-BI research question of this manuscript, additional research is needed.

These insights gained help to further tailor T&S, FR, and e-BI implementation strategies in order to achieve maximum gains in increasing alcohol SBI and risky alcohol consumption. However, the macro-level in which SBI is augmented to be implemented has an influential role. High potential implementation strategies on the micro level could get nullified due to influences from the macro- level such as societal and political culture. Focusing only on the primary healthcare setting seems insufficient and a more integrated SBI culture, together with meso- and macro-focused implementation process is requested.

Abbreviations

COREQ, consolidated criteria for reporting qualitative research; e-BI, internet-based method of delivering advice; FR, financial reimbursement; GP, general practitioner; PHCU, primary healthcare unit; SBI, screening and brief intervention; T&S, training & support; TICD, tailored implementation for chronic diseases

Acknowledgements

We would like to thank all participating GPs and nurses participating in this study. We would also like to thank Birgit Jansen (research assistant, Radboud university medical center, Scientific Institute for Quality of Healthcare) for executing many logistical procedures.

Funding

The research leading to these results or outcomes has received funding from the European Union's Seventh Framework Program for research, technological development and demonstration under grant agreement no 259268 – Optimizing delivery of healthcare intervention (ODHIN). Participant organisations in ODHIN can be seen at: www.odhinproject.eu. Radboud university medical center received co-funding from The Netherlands Organization for Health Research and Development (ZonMW, Prevention Program), under Grant Agreement no 200310017 – ODHIN – Optimizing delivery of healthcare interventions in the Netherlands, according Art.II.17 of the FP7 EC Grant Agreement.

Availability of data and materials

Table 2 presents codes that were based on primary data and which were summarised in the main text of the article. Individual data will not be shared in order to protect the participants' identity.

For peer review only - http://bmjopen.bmj.com/site/about/guidelines.xhtml

Authors' contributions
MK, ML and MH coordinated the study internationally. CL, UM and FS were responsible for data collection and analysis in Sweden. JC, JPV, LSe and ES were responsible for data collection and analysis in Catalonia. KO and LSl were responsible for data collection and analysis in Poland. MK, ML and MH were responsible for data collection and analysis in the Netherlands. MK, MH, ML, CL, FS, KO, LSl and LSe participated in the meetings and conference calls to discuss the outcomes and data-synthesis. MK wrote the first draft of the manuscript and all other authors revised the manuscript critically. MK, ML, and MH led the data-synthesis, meetings and first drafting of the manuscript. MW supervised the design of the study and analysis of data. All authors read and approved the final manuscript.

Competing interests
Miranda Laurant is Associate Editor of BMC Family Practice. The other authors have no competing interests.

Consent for publication
There was no consent for publication of individual person's data obtained.

Ethics approval and consent to participate
Ethics approval for the study was obtained from the relevant approval bodies within each country: In Catalonia, the Clinical Research Ethics Committee of the Jordi Gol I Gurina Primary Health Care Research Institute and from the Clinical Research Ethics Committee of Hospital Clínic de Barcelona; in Poland, Resolution No. KB- 0012/105/11 adopted by the Commission of Bioethics of the Pomeranian Medical University in Szczecin; and, in Sweden by the: Regional Ethical Review Board in Göteborg, reference number: 658/12, with approval granted for both sites in Göteborg and Linköping. In the Netherlands, the Committee on Research inv. Human Subjects (CMO) ethical board declared that no ethical approval was required in the Netherlands, reference number: 2012/281. In all four countries, all participating healthcare providers signed a written informed consent.

Author details
[1]Radboud university medical center, Radboud Institute for Health Sciences, IQ healthcare, Nijmegen, The Netherlands. [2]Saxion University of Applied Sciences, Centre for Nursing Research, Deventer/Enschede, The Netherlands. [3]Program on Substance Abuse, Public Health Agency, Government of Catalonia, Barcelona, Spain. [4]Department of Medicine and Health Sciences, Linköping University, Linköping, Sweden. [5]State Agency for Prevention of Alcohol-Related Problems, Warsaw, Poland. [6]The Section for Epidemiology and Social Medicine, University of Gothenburg, Gothenburg, Sweden. [7]HAN University of Applied Sciences, Faculty of Health and Social Studies, Nijmegen, The Netherlands.

Received: 7 November 2015 Accepted: 20 May 2016
Published online: 07 June 2016

References
1. World Health Organization. Global status report on alcohol and health 2014. Geneva: World Health Organization; 2014.
2. Funk M, Wutzke S, Kaner E, Anderson P, Pas L, McCormick R, Gual A, Barfod S, Saunders J, World Health Organization Brief Intervention Study G. A multicountry controlled trial of strategies to promote dissemination and implementation of brief alcohol intervention in primary health care: findings of a World Health Organization collaborative study. J Stud Alcohol. 2005;66:379–88.
3. Ballesteros J, Duffy JC, Querejeta I, Arino J, Gonzalez-Pinto A. Efficacy of brief interventions for hazardous drinkers in primary care: systematic review and meta-analyses. Alcohol Clin Exp Res. 2004;28:608–18.
4. Bertholet N, Daeppen JB, Wietlisbach V, Fleming M, Burnand B. Reduction of alcohol consumption by brief alcohol intervention in primary care: systematic review and meta-analysis. Arch Intern Med. 2005;165:986–95.
5. Kaner EF, Beyer F, Dickinson HO, Pienaar E, Campbell F, Schlesinger C, Heather N, Saunders J, Burnand B. Effectiveness of brief alcohol interventions in primary care populations. Cochrane Database Syst Rev. 2007;18(2):CD004148.
6. Moyer A, Finney JW, Swearingen CE, Vergun P. Brief interventions for alcohol problems: a meta-analytic review of controlled investigations in treatment-seeking and non-treatment-seeking populations. Addiction. 2002;97:279–92.
7. Drummond C, Wolstenholme A, Deluca P, Davey Z, Donoghue K, Elzerbi C, Gual A, Robles N, Goos C, Strizek J, et al. Alcohol interventions and treatment in Europe. In: Anderson P, Braddick F, Reynolds J, Gual A, editors. Alcohol policy in Europe: evidence from AMPHORA 2nd ed the AMPHORA project. 2013.
8. Anderson P. Overview of interventions to enhance primary-care provider management of patients with substance-use disorders. Drug Alcohol Rev. 2009;28:567–74.
9. Noordman J, Koopmans B, Korevaar JC, van der Weijden T, van Dulmen S. Exploring lifestyle counselling in routine primary care consultations: the professionals' role. Fam Pract. 2013;30:332–40.
10. Nilsen P. Brief alcohol intervention–where to from here? Challenges remain for research and practice. Addiction. 2010;105:954–9.
11. Roche AM, Freeman T. Brief interventions: good in theory but weak in practice. Drug Alcohol Rev. 2004;23:11–8.
12. University of Sheffield. Prevention and early identification of alcohol use disorders in adults and young people. Final draft of report 2 to the National Institute FOR Health & Clinical Excellence. Sheffield: University of Sheffield: School of Health and Related Research (ScHARR); 2009.
13. Drevenhorn E, Bengtson A, Nilsson PM, Nyberg P, Kjellgren KI. Consultation training of nurses for cardiovascular prevention - a randomized study of 2 years duration. Blood Press. 2012;21:293–9.
14. van Beurden I, Anderson P, Akkermans RP, Grol RP, Wensing M, Laurant MG. Involvement of general practitioners in managing alcohol problems: a randomized controlled trial of a tailored improvement programme. Addiction. 2012;107:1601–11.
15. Anderson P, Laurant M, Kaner E, Wensing M, Grol R. Engaging general practitioners in the management of hazardous and harmful alcohol consumption: results of a meta-analysis. J Stud Alcohol. 2004;65:191–9.
16. Nilsen P, Aalto M, Bendtsen P, Seppa K. Effectiveness of strategies to implement brief alcohol intervention in primary healthcare. A systematic review. Scand J Prim Health Care. 2006;24:5–15.
17. Williams EC, Johnson ML, Lapham GT, Caldeiro RM, Chew L, Fletcher GS, McCormick KA, Weppner WG, Bradley KA. Strategies to implement alcohol screening and brief intervention in primary care settings: a structured literature review. Psychol Addict Behav. 2011;25:206–14.
18. Flottorp SA, Oxman AD, Krause J, Musila NR, Wensing M, Godycki-Cwirko M, Baker R, Eccles MP. A checklist for identifying determinants of practice: a systematic review and synthesis of frameworks and taxonomies of factors that prevent or enable improvements in healthcare professional practice. Implement Sci. 2013;8:35.
19. Rubio-Valera M, Pons-Vigues M, Martinez-Andres M, Moreno-Peral P, Berenguera A, Fernandez A. Barriers and facilitators for the implementation of primary prevention and health promotion activities in primary care: a synthesis through meta-ethnography. PLoS One. 2014;9:e89554.
20. Amaral MB, Ronzani TM, Souza-Formigoni ML. Process evaluation of the implementation of a screening and brief intervention program for alcohol risk in primary health care: an experience in Brazil. Drug Alcohol Rev. 2010;29:162–8.
21. Johnson M, Jackson R, Guillaume L, Meier P, Goyder E. Barriers and facilitators to implementing screening and brief intervention for alcohol misuse: a systematic review of qualitative evidence. J Public Health (Oxf). 2011;33:412–21.
22. Keurhorst MN, Anderson P, Spak F, Bendtsen P, Segura L, Colom J, Reynolds J, Drummond C, Deluca P, van Steenkiste B, et al. Implementing training and support, financial reimbursement, and referral to an internet-based brief advice program to improve the early identification of hazardous and harmful alcohol consumption in primary care (ODHIN): study protocol for a cluster randomized factorial trial. Implement Sci. 2013;8:11.
23. Wong G, Greenhalgh T, Westhorp G, Pawson R. Realist methods in medical education research: what are they and what can they contribute? Med Educ. 2012;46:89–96.
24. Pawson R, Tilley N. Realistic evaluation. London: Sage; 1997.
25. Tong A, Sainsbury P, Craig J. Consolidated criteria for reporting qualitative research (COREQ): a 32-item checklist for interviews and focus groups. Int J Qual Health Care. 2007;19:349–57.
26. Corbin J, Strauss A. Grounded theory research: procedures, canons, and evaluative criteria. Qual Sociol. 1990;13:3–21.
27. Johnson JK, Barach P, Vernooij-Dassen M, Collaborative HR. Conducting a multicentre and multinational qualitative study on patient transitions. BMJ Qual Saf. 2012;21 Suppl 1:i22–8.
28. Hawe P. Lessons from complex interventions to improve health. Annu Rev Public Health. 2015;36:307–23.

For peer review only

29. Trimbos-instituut. E-mental health by practice nurse specialized in mental health and the GP. Wat do we need for upscaling? (E-mental health door POH-GGZ en huisarts. Wat is er nodig voor opschaling?), vol. AF1364. Utrecht: Trimbos-instituut; 2014.
30. Bransen E, de Gee A, Drenthen T, Boon B. Online help works for alcohol problems [Onlinehulp werkt goed bij alcoholprobleem]. Medisch Contact. 2015;10:450–2.
31. Donoghue K, Patton R, Phillips T, Deluca P, Drummond C. The effectiveness of electronic screening and brief intervention for reducing levels of alcohol consumption: a systematic review and meta-analysis. J Med Internet Res. 2014;16:e142.
32. Wallace P, Murray E, McCambridge J, Khadjesari Z, White IR, Thompson SG, Kalaitzaki E, Godfrey C, Linke S. On-line randomized controlled trial of an internet based psychologically enhanced intervention for people with hazardous alcohol consumption. PLoS One. 2011;6:e14740.
33. van de Glind I, Heinen M, Geense W, Mesters I, Wensing M, van Achterberg T. Making the connection-factors influencing implementation of evidence supported and non-evaluated lifestyle interventions in healthcare: a multiple case study. Health Educ Res. 2015;30:521–41.
34. Kontopantelis E, Springate DA, Ashworth M, Webb RT, Buchan IE, Doran T. Investigating the relationship between quality of primary care and premature mortality in England: a spatial whole-population study. BMJ. 2015;350:h904.
35. Roland M, Campbell S. Successes and failures of pay for performance in the United Kingdom. N Engl J Med. 2014;370:1944–9.
36. Dalkin SM, Greenhalgh J, Jones D, Cunningham B, Lhussier M. What's in a mechanism? Development of a key concept in realist evaluation. Implement Sci. 2015;10:49.
37. Rycroft-Malone J, Fontenla M, Bick D, Seers K. A realistic evaluation: the case of protocol-based care. Implement Sci. 2010;5:38.
38. Chaudoir SR, Dugan AG, Barr CH. Measuring factors affecting implementation of health innovations: a systematic review of structural, organizational, provider, patient, and innovation level measures. Implement Sci. 2013;8:22.

For peer review only

Submit your next manuscript to BioMed Central and we will help you at every step:

- We accept pre-submission inquiries
- Our selector tool helps you to find the most relevant journal
- We provide round the clock customer support
- Convenient online submission
- Thorough peer review
- Inclusion in PubMed and all major indexing services
- Maximum visibility for your research

Submit your manuscript at www.biomedcentral.com/submit

BioMed Central

SCALA – Documentation of PHCC Recruitment

1) Please specify the country as well as the name of the researcher responsible for PHCC recruitment:

**Country**	☐ Mexico<br>☐ Colombia<br>☐ Peru
**Responsible researcher**	______________________

For peer review only

2) During recruitment of the PHCCs, local researchers should document the following points *for each municipality*:

**Name of municipality**	______________________
**Control / Intervention**	☐ Control<br>☐ Intervention
**Total number of PHCCs in municipality**	______________________
**Number of PHCCs contacted for study participation**	______________________
**Number of non-responding PHCCs**	______________________
**Number of PHCCs refusing to participate**	______________________
**Number of PHCCs accepting to participate**	______________________

3) Further, the following points need to be documented *for each contacted PHCC*:

**Name/Address/Identifier of PHCC**	______________________
**Characteristics of PHCC (if known)**	☐ Number of registered patients: ______<br>☐ Number of GPs: ______<br>☐ Number of nurses: ______<br>☐ Number of all workers: ______<br>☐ other:<br>__________________________________
**Contact with PHCC**	☐ By mail<br>☐ By email<br>☐ By telephone<br>☐ Personal contact<br>☐ other:<br>__________________________________
**Number of contacts with PHCC before decision (acceptance/refusal/non-response)**	______________________
**Accepted / Refused / No response**	☐ Accepted<br>☐ Refused<br>☐ No response
**If refused, give reasons**	____<br>____<br>____
**If no response, any reasons suspected?**	____<br>____<br>____

For peer review only

SCALA – Provider follow-up documentation

Provider details

During the course of the study, each PHC provider should be followed up with regard to participation in training sessions. Further, potential drop outs should be documented here. Please fill in this sheet ***for each provider.***

**Country**	☐ Mexico<br>☐ Colombia<br>☐ Peru
**Responsible researcher**	______________________
**Name of municipality**	______________________
**Control / Intervention**	☐ Control<br>☐ Intervention
**Name/Address/Identifier of PHCC**	______________________
**Name/Identifier of provider**	______________________
**Gender of provider**	☐ Female<br>☐ Male<br>☐ Other
**Age of provider**	______________________ (in years of age)
**Baseline month**	from ____ / ____ / ____ until ____ / ____ / ____<br>(DD / MM / YY)

For peer review only

Participation in training sessions

**Training session**	☐ Pre-implementation Training 1<br>☐ Pre-implementation Training 2<br>☐ Booster 1<br>☐ Booster 2
**Date of training**	____ / ____ / ____ (DD / MM / YY)
**Training participation**	☐ Participated in training<br>☐ Absent in training
**Reason for training absence**	☐ with valid excuse, ie. ________________<br>☐ without valid excuse
**If absent at training, could training be repeated?**	☐ Yes, on ____ / ____ / ____ (DD / MM / YY)<br>☐ No

For peer review only

Drop out

If the provider dropped out before end of the study, the following section need to be filled in:

**Date of drop out**	____ / ____ / ____ (DD / MM / YY)
**Date of last tally sheet completed by provider**	____ / ____ / ____ (DD / MM / YY)
**Drop out in relation to data collection**	☐ Before baseline data collection<br>☐ During baseline data collection<br>☐ After baseline data collection, but before 18-month implementation period<br>☐ During specific month of 18-month implementation period (enter number of month from 1 to 18).
**Reasons for drop out**	____<br>____<br>____<br>____<br>____

For peer review only

RE-AIM Dimension, SCALA aims	SCALA activities	Main outcome/process measures
**REACH**<br>• In PHC, to maximise exposure to screening for AUD<br>• In PHC, to maximise exposure to advice and treatment for AUD and comorbid depression<br>• In PHC, to maximise exposure to alcohol health literacy information materials	• Recruitment of PHCCs in each city with large population coverage of about 160,000 registered patients per PHCC<br>• Recruitment of representative PHCC population within cities to maximise<br>– Take-up of alcohol health literacy information materials<br>– Numbers screened for AUD<br>– Numbers receiving appropriate advice/referral for AUD/depression	• Total number of PHCC patients screened for AUD<br>• Total number of screen positive patients receiving appropriate advice/referral for AUD/depression<br>• Representativeness of population screened and/or receiving appropriate advice/referral for AUD
**EFFECTIVENESS**<br>• To design and apply an evidence-based care pathway to address AUD and comorbid depression in primary health care	Design and delivery of an intervention package within a primary health care based care pathway that incorporates:<br>• State-of-the-art alcohol health literacy information materials<br>• AUDIT-C screening instrument<br>• Brief advice and treatment for case positives<br>• Referral of severe AUD and comorbid depression	• Increased health literacy in PHCC patients using UK-based Newest Vital Sign and an adapted version of Health Literacy Survey-EU Questionnaire (HLS-EU Q)<br>• Reduction in alcohol consumption of AUD+ drinkers
**ADOPTION**<br>• To increase the adoption of the intervention package in primary health care	• Design of a pragmatic, easy to use and replicate PHCC intervention package and associated care pathway<br>• Tailoring of the PHCC package according to local needs (PHC setting, PHCC) by using Community Advisory Boards (CABs) and User Panels (UPs)<br>• Provision of specific practice-based training and ongoing support to PHCC<br>• Development of city-based adoption mechanisms and support systems	• Adoption rate and representativeness of PHCCs<br>• Adoption rate and representativeness of PHCC staff
**IMPLEMENTATION**<br>• To assess the fidelity and costs of implementing the intervention package<br>• To evaluate which factors affect the implementation of the intervention package	• Continuous feedback on PHCC level drivers to package implementation gathered via qualitative and quantitative metrics<br>• Application of WHO Urban Health Equity Assessment and Response Tool<br>• Application of MRC framework to map and understand progress towards effective scale-up	• Extent primary health care screening and advice package delivered as intended<br>• Multi-level evaluation of barriers/facilitators to scale-up using WHO's Urban Health Equity Assessment and Response Tool<br>• Extent implementation on city levels delivered as intended using Medical Research Council guidance<br>• Cost of package implementation
**MAINTENANCE**<br>• To report on long-term effects of package at individual and organisational levels<br>• To understand how the programme can be maintained and achieve longevity within the test cities	• Support at the system level to make relevant practice changes for sustainability<br>• Monitoring system on long-term effectiveness<br>• Monitoring system on performance on PHCC level<br>• Production of Step-by-step SCALA Framework and Strategy	• Assessment of outcomes 18 months post implementation<br>• Indicators of program-level maintenance<br>• Measures of cost of maintenance<br>• Dissemination / events

BMJ Open

Implementing primary health care-based measurement, advice and treatment for heavy drinking and comorbid depression at the municipal level in three Latin American countries: final protocol for a quasi-experimental study (SCALA study)

Journal:	*BMJ Open*
Manuscript ID	bmjopen-2020-038226.R1
Article Type:	Protocol
Date Submitted by the Author:	21-Apr-2020
Complete List of Authors:	Jane LLopis, Eva; Ramon Llull University<br>Anderson, Peter; Maastricht Univ; Newcastle University, Institute Health and Society<br>Piazza, Marina; Universidad Peruana Cayetano Heredia<br>O'Donnell, Amy; Newcastle University, Institute of Health and Society<br>Gual, Antoni; Hospital Clínic de Barcelona, Addiction Unit<br>Schulte, Bernard; University Medical Center Hamburg-Eppendorf<br>Perez, Augusto; Corporación Nuevos Rumbos<br>de Vries, Hein; Maastricht University (CAPHRI), Health Promotion; Maastricht University<br>Natera Rey, Guillermina; Instituto Nacional de Psiquiatría Ramón de la Fuente Muñiz<br>Kokole, Dasa; Maastricht University Faculty of Health Medicine and Life Sciences<br>Bustamante, Ines; Universidad Peruana Cayetano Heredia<br>Braddick, Fleur; Hospital Clinic de Barcelona<br>Mejía Trujillo, Juliana; Corporación Nuevos Rumbos<br>Solovei, Adriana; Maastricht Universitair Medisch Centrum+<br>Pérez De León, Alexandra; Instituto Nacional de Psiquiatría Ramón de la Fuente Muñiz<br>Kaner, Eileen; Newcastle University, Institute<br>Matrai, Silvia; Hospital Clinic de Barcelona<br>Manthey, Jakob; Technische Universitat Dresden,<br>Mercken, Liesbeth; Maastricht University<br>López-Pelayo, Hugo; Hospital Clínic de Barcelona, Addiction Unit<br>Rowlands, Gillian; Newcastle University Institute for Health and Society,<br>Schmidt, CS; Universitatsklinikum Hamburg-Eppendorf,<br>Rehm, Jürgen; Centre for Addiction and Mental Health,
**Primary Subject Heading**:	General practice / Family practice
Secondary Subject Heading:	Public health
Keywords:	PRIMARY CARE, Substance misuse < PSYCHIATRY, Depression & mood disorders < PSYCHIATRY

For peer review only - http://bmjopen.bmj.com/site/about/guidelines.xhtml

SCHOLARONE™
Manuscripts

I, the Submitting Author has the right to grant and does grant on behalf of all authors of the Work (as defined in the below author licence), an exclusive licence and/or a non-exclusive licence for contributions from authors who are: i) UK Crown employees; ii) where BMJ has agreed a CC-BY licence shall apply, and/or iii) in accordance with the terms applicable for US Federal Government officers or employees acting as part of their official duties; on a worldwide, perpetual, irrevocable, royalty-free basis to BMJ Publishing Group Ltd ("BMJ") its licensees and where the relevant Journal is co-owned by BMJ to the co-owners of the Journal, to publish the Work in this journal and any other BMJ products and to exploit all rights, as set out in our licence.

The Submitting Author accepts and understands that any supply made under these terms is made by BMJ to the Submitting Author unless you are acting as an employee on behalf of your employer or a postgraduate student of an affiliated institution which is paying any applicable article publishing charge ("APC") for Open Access articles. Where the Submitting Author wishes to make the Work available on an Open Access basis (and intends to pay the relevant APC), the terms of reuse of such Open Access shall be governed by a Creative Commons licence – details of these licences and which Creative Commons licence will apply to this Work are set out in our licence referred to above.

Other than as permitted in any relevant BMJ Author's Self Archiving Policies, I confirm this Work has not been accepted for publication elsewhere, is not being considered for publication elsewhere and does not duplicate material already published. I confirm all authors consent to publication of this Work and authorise the granting of this licence.

For peer review only

Implementing primary health care-based measurement, advice and treatment for heavy drinking and comorbid depression at the municipal level in three Latin American countries: final protocol for a quasi-experimental study (SCALA study)

Eva Jané Llopis[1-3]
Peter Anderson[2,4]
Marina Piazza[5]
Amy O'Donnell[4]
Antoni Gual[6-8]
Bernd Schulte[9]
Augusto Pérez Gómez[10]
Hein de Vries[2]
Guillermina Natera Rey[11]
Dasa Kokole[2]
Ines V. Bustamante [5]
Fleur Braddick[7]
Juliana Mejía Trujillo[10]
Adriana Solovei[2]
Alexandra Pérez De León[11]
Eileen Kaner[4]
Silvia Matrai[7]
Jakob Manthey[9,12]
Liesbeth Mercken[2]
Hugo Lopez-Pelayo[6-8]
Gill Rowlands[4]
Christiane Schmidt[9]
Jürgen Rehm[3, 12-15]

[1] Univ. Ramon Llull, ESADE, Barcelona, Spain. Email: eva.jane@esade.edu

[2] Department of Health Promotion, CAPHRI Care and Public Health Research Institute, Maastricht University, POB 616, 6200 MD, Maastricht, The Netherlands. Email: e.llopis@maastrichtuniversity.nl; peter.anderson@maastrichtuniversity.nl; hein.devries@maastrichtuniversity.nl; d.kokole@maastrichtuniversity.nl; a.solovei@maastrichtuniversity.nl; Liesbeth.mercken@maastrichtuniversity.nl

[3] Institute for Mental Health Policy Research, CAMH, 33 Russell Street, Toronto, ON M5S 2S1, Canada. Email: evajanellopis@gmail.com; jtrehm@gmail.com

[4] Population Health Sciences Institute, Newcastle University, Baddiley-Clark Building, Richardson Road, Newcastle upon Tyne NE2 4AX, UK. Emails: Peter.Anderson@newcastle.ac.uk; amy.odonnell@newcastle.ac.uk; eileen.kaner@newcastle.ac.uk

[5]School of Public Health and Administration, Universidad Peruana Cayetano Heredia e-mail: marina.piazza@upch.pe; ines.bustamante@upch.pe

For peer review only

For peer review only - http://bmjopen.bmj.com/site/about/guidelines.xhtml

[6] Red de Trastornos Adictivos. Instituto Carlos III. Sinesio Delgado, 4; 28029 – Madrid. Email: TGUAL@clinic.cat; hlopez@clinic.cat

[7]Addictions Unit. Psychiatry Dept. Hospital Clínic. Villarroel 170; 08036 Barcelona, Spain. Email: TGUAL@clinic.cat, hlopez@clinic.cat; smatrai@clinic.cat; fmbooth@clinic.cat

[8] Institut d'Investigacions Biomèdiques August Pi Sunyer (IDIBAPS); Rosselló, 149-153; 08036 Barcelona, Spain. Email: TGUAL@clinic.cat; hlopez@clinic.cat

[9] Center for Interdisciplinary Addiction Research (ZIS), Department of Psychiatry and Psychotherapy, University Medical Center Hamburg-Eppendorf, Hamburg, Germany. Email: b.schulte@uke.uni-hamburg.de; christiane.schmidt@uke.de; jakobmanthey@snappyquest.org

[10] Corporación Nuevos Rumbos, Calle 108 A # 4-15, Bogotá, Colombia. Email: aperez@nuevosrumbos.org; jmejia@nuevosrumbos.org

[11] Instituto Nacional de Psiquiatría Ramón de la Fuente Muñiz, Calz México-Xochimilco 101, Huipulco, 14370 Ciudad de México, CDMX, Mexico. Email: naterar@imp.edu.mx; apdeleon@imp.edu.mx

[12] Institute for Clinical Psychology and Psychotherapy, TU Dresden, Chemnitzer Str. 46, 01187 Dresden, Germany. Email: jtrehm@gmail.com; jakob.manthey@tu-dresden.de

[13] Dalla Lana School of Public Health, University of Toronto, 155 College Street, 6th Floor, Toronto, ON M5T 3M7, Canada. Email: jtrehm@gmail.com

[14] Department of Psychiatry, University of Toronto, 250 College Street, 8th Floor, Toronto, ON M5T 1R8, Canada. Email: jtrehm@gmail.com

[15] Department of International Health Projects, Institute for Leadership and Health Management, I.M. Sechenov First Moscow State Medical University, Trubetskaya str., 8, b. 2, 119992, Moscow, Russian Federation jtrehm@gmail.com

Corresponding author: Eva Jané Llopis: eva.jane@esade.edu

For peer review only

Abstract

Introduction: While primary health care-based prevention and management of heavy drinking is clinically effective and cost-effective, it remains poorly implemented in routine practice. Systematic reviews and multi-country studies have demonstrated the ability of training and support programmes for healthcare professionals to increase primary health care-based measurement and brief advice activity to reduce heavy drinking. However, gains have been only modest and short term at best. WHO studies have concluded that a more effective uptake could be achieved by embedding primary health care activity within broader municipal-based support.

Methods and analysis: A quasi-experimental four-arm study will compare primary health care-based prevention and management of heavy drinking and co-morbid depression in three intervention municipal areas from Colombia, Mexico and Peru with three control municipal areas from the same countries. Fifty-four primary health care units will be enrolled. In the implementation municipal areas, 27 primary health care units will receive training on measuring alcohol consumption and managing heavy drinking and comorbid depression embedded within ongoing supportive municipal action over an 18-month implementation test period; 12 units will implement a standard alcohol measurement and advice package (Arm 4), and 15 units a short package (Arm 3). In the control municipal areas, 15 units will receive training (Arm 2), and 12 units will continue with practice as usual (Arm 1). All patients identified as heavy drinkers will be assessed and managed, as appropriate, for comorbid depression. The primary outcome is the proportion of the adult population (aged 18+ years) registered with the unit that has their alcohol consumption measured. Return-on-investment analyses and full process evaluation will be undertaken, coupled with an analysis of potential contextual, financial and political-economy influencing factors.

Ethics and dissemination: The Ethics Committee of the Technical University of Dresden gave final ethical approval for the SCALA project on 12 April 2019, EK90032018. A dissemination strategy is in place with Ministries of Health at municipal and country levels; and, with Pan American Health Organization at Latin American level to scale up the implementation strategy, once validated.

Trial Registration: Clinical Trials.gov ID: NCT03524599; Registered 15 May 2018; https://clinicaltrials.gov/ct2/show/NCT03524599

Protocol Version: Final version, 25 February 2020.

Key words: Primary health care; municipal action; heavy drinking; comorbid depression; Institute for Health Care Improvement; implementation; measurement of alcohol consumption; AUDIT-C.

For peer review only - http://bmjopen.bmj.com/site/about/guidelines.xhtml

Strengths and Limitations of Study

1. Uses a theory-based approach to tailor clinical materials and training programmes, creating city-based Community Advisory Boards, and user-based User Panels to ensure that tailoring matches user needs, municipal services, and co-production of health;
2. Tests the added value of embedding and implementing primary health care activity within municipal-based adoption mechanisms and support systems, and community-based communication campaigns;
3. Has a longer time frame (18 months) than is traditionally used in implementation studies, to assess longer term impacts;
4. Gives considerable emphasis to process evaluation, developing logic models to document the fidelity of all implementation strategies, and to identify, the drivers and barriers and facilitators to successful implementation and scale-up; and
5. Due to municipal-based political and technical considerations, we are unable to randomize the involved municipal areas. We adopt a quasi-experimental design, optimizing comparator municipal areas for confounding, and by using propensity score matching.

For peer review only

For peer review only - http://bmjopen.bmj.com/site/about/guidelines.xhtml

ABBREVIATIONS AND ACRONYMS

AIDS: Acquired Immune Deficiency Syndrome

AUDIT-10: Alcohol Use Disorders Identification Test, full 10-item version

AUDIT-C: Alcohol Use Disorders Identification Test, 3-item consumption version

CAB: Community Advisory Board

HIV: Human Immunodeficiency Virus Infection

IHI: Institute for Healthcare Improvement.

NCD: Non-Communicable Disease

ODHIN: Optimizing Delivery of Health Care Interventions

OECD: Organization for Economic Cooperation and Development

PHC: Primary Health Care

PHCU: Primary Health Care Unit

PHQ-2: Patient Health Questionnaire (mental disorders), 2-item version

PHQ-9: Patient Health Questionnaire (mental disorders), 9-item version

PSM: Propensity Score Matching

RE-AIM: Reach, Effectiveness, Adoption, Implementation, and Maintenance

ROI: Return on Investment

SAAPPQ: Short Alcohol and Alcohol Problems Perception Questionnaire

SAMHSA: Substance Abuse and Mental Health Services Administration

SBIRT: Screening, Brief Intervention and Referral to Treatment

SCALA: Scale-up of Prevention and Management of Alcohol Use Disorders and Comorbid Depression in Latin America

TB: Tuberculosis

UP: User Panel

WHO: World Health Organization

For peer review only

For peer review only - http://bmjopen.bmj.com/site/about/guidelines.xhtml

INTRODUCTION

This paper outlines the protocol for a quasi-experimental study[1] to test the implementation of primary health care-based measurement, advice and treatment for heavy drinking and comorbid depression at the municipal level in three Latin American countries, Colombia, Mexico and Peru (SCALA study).

Heavy drinking is a cause of considerable disability, morbidity, and mortality[2]. Heavy drinking is a causal factor for some communicable diseases (including TB and HIV/AIDS), for many non-communicable diseases (including cancers, cardiovascular diseases and gastrointestinal diseases) and for many mental and behavioural disorders, including depression, dementias and suicide[3,4].

In PHC settings, two-fifths of people with heavy drinking have depression, with risks of incident depression higher for heavier as opposed to lighter drinkers[5]. In addition to its role in the aetiology of depression, heavy drinking is associated with worsening the depression course, including suicide risk, impaired social functioning and impaired health care utilization[6].

Heavy drinking is also a major contributor to global health inequalities, with alcohol-related harm aggravated by lower socio-economic status[7] and extending beyond the individual drinker to families, communities, health systems, and the wider economy. Tackling the multiple individual and societal level harms caused by heavy drinking is essential for achieving global targets of reducing deaths from NCDs by 25% between 2010 and 2025[8], more so as risk of exposure to harmful use of alcohol increases with increasing socio-economic status[9]. In line with tackling harm due to lower socio-economic status, United Nations Sustainable Development Goals include Target 3.5, to strengthen the prevention and treatment of harmful use of alcohol, with two proposed indicators: coverage of treatment interventions (pharmacological, psychosocial and rehabilitation and aftercare services) for harmful use of alcohol; and per capita alcohol consumption[10,11].

Countries in Latin America have the highest alcohol-attributable disease burden after Eastern Europe and Sub-Saharan Africa, with particularly high risks in alcohol-attributable traffic injury including violence[12]. The burden of alcohol-attributable diseases in Latin America lead to marked economic costs, with numerous calls to implement effective and cost-effective policies (e.g.[13]).

A robust and extensive body of literature demonstrates the range of evidence-based strategies that can be implemented to reduce heavy drinking in health care settings[14]. Questionnaire-based measurement and brief advice programmes delivered in PHC are effective[15] and cost-effective[16,17] in reducing heavy drinking. In addition to brief advice, treatment for heavy drinking includes cognitive behavioural therapy and pharmacotherapy, both of which are found to be effective in reducing heavy drinking[18]. Were the proportion of eligible patients receiving advice and treatment for heavy drinking to increase to 30% of eligible patients, the prevalence of harmful use of alcohol could decrease by between 10% and 15% across OECD member countries[19]. However, to date, measurement and brief advice and treatment programmes have failed to achieve widespread take-up[19].

Two systematic reviews[20,21] and two multi-country studies[22-24] have demonstrated that the proportion of PHC patients whose alcohol consumption is measured, and of heavy drinking patients given advice

For peer review only - http://bmjopen.bmj.com/site/about/guidelines.xhtml

can be increased by providing training and support to PHC providers, albeit from very low baseline levels, and with effects not generally sustained over the longer term. Moreover, whilst there has been some previous research in countries of Latin America[25-30], most implementation work to date has been undertaken in high-income countries. The SCALA study will build on previous evidence[31] to fast-track scale-up research and practice in Latin American primary health care settings.

Out of a range of implementation frameworks that include a sequential approach for scale-up, and that provide practical guidance for how to work with organizations, health systems, and communities to implement and scale-up best practices[32-39], we adopt the Institute for Healthcare Improvement's (IHI) Framework for going to Full Scale, which identifies adoption mechanisms and support systems for use across sequential steps, and describes the implementation methods that can be used at each step[40].

SCALA seeks to address three specific barriers to sustained implementation of primary health care-based measurement, advice and treatment for heavy drinking. The first barrier recognizes that most PHC-based programmes focus on providers alone, whereas successful implementation of health interventions within complex health system demands addressing a range of underlying structural and support systems[40]. Phase IV of the WHO study on the identification and management of alcohol-related problems in primary care concluded that embedding PHC-based measurement and brief advice programmes within the frame of supportive community and municipal environments might lead to improved outcomes[41], although this has never been formally evaluated. Similar conclusions were reached by the European ODHIN study[42] and the US-based SAMHSA SBIRT initiative[43-45].

The second barrier is that standard cut-off points for the frequently used alcohol measurement instrument, AUDIT-C[46] (commonly a score of five for both men and women, or five for men and four for women) to trigger advice are too low[47], being equivalent to an average daily alcohol consumption of about 20 grams of alcohol (around 2 standard drinks) or less[48]. Practitioners may well find it problematic to give advice at such levels, which would also have huge time implications, with one in three or four patients being eligible for advice in many countries, under this criterion[24, 49]. We have argued to adopt similar models to blood pressure, where cut-off points for managing raised blood pressure are often determined by levels of blood pressure at which treatment has shown to be effective[50,51]. Similarly, cut-off points for brief advice could be the baseline levels of alcohol consumption found in the randomized controlled trials that have investigated the effectiveness of PHC-delivered brief advice. In the first Cochrane review of the topic that focused on primary health care, mean baseline levels were 313 grams of alcohol per week[52], equivalent to an AUDIT-C cut-off of 8[48].

The third and final barrier concerns the cost of implementing measurement and brief-advice for heavy drinking in primary health care setting. Although, alcohol advice and treatment programmes can lead to substantial reductions in health care costs[16], freeing considerable numbers of working age people from alcohol-related diseases[19], their initial implementation can require a significant time-commitment on the part of providers, in terms of both initial training requirements and the time taken to deliver advice in routine practice. The largest part of the costs of implementing measurement and

For peer review only - http://bmjopen.bmj.com/site/about/guidelines.xhtml

brief advice for heavy drinking in primary health care settings are directly caused by the time spent by the health care providers delivering this intervention[53]. Moreover, this large amount of time is experienced by health care providers as an important barrier to deliver routine measurement and brief advice to their patients[54]. As evidence suggests that shorter sessions of brief advice are not less effective compared to shorter sessions[52, 55, 56], it seems that reducing the time spent by health care professionals in preparing for these sessions could be a viable strategy to increase the overall adoption and implementation of alcohol measurement and brief advice at primary health care level.

Given the strong comorbidity between heavy drinking and depression, our protocol includes screening for depression for those patients identified as heavy drinkers, with appropriate referral or PHC support for treatment[57, 58, 59].

In the SCALA study, we implement three interventions (independent variables) for the PHCU:

i. Intensity of clinical package and training (standard, versus short, versus none);
ii. Training of providers (present, versus absent); and,
iii. Community integration and support (municipal action present, versus absent).

The main outcome (dependent variable) is the cumulative proportion of the adult (aged 18+ years) population registered with the PHCU that has their alcohol consumption measured within the 18-month implementation test period (defined as coverage). Three hypotheses are to be tested:

Hypothesis 1: Municipal action leads to more sustainable coverage. After 18 months, the difference in coverage between municipal action present and municipal action absent for those PHCU that receive training is larger than after 12 months;

Hypothesis 2: In the absence of municipal action, PHCU that have received training obtain higher coverage than PHCU that do not receive training; and,

Hypotheses 3: In the presence of municipal action, the short clinical package and short training do not lead to less measurement coverage than the standard clinical package and standard training.

METHODS AND ANALYSIS

The study is a quasi-experimental design[1], comparing changes in measurement and assessment for alcohol consumption and comorbid depression, and, if needed, advice and/or referral for treatment between primary health care units (PHCUs) in intervention municipal areas and PHCUs in similar control municipal areas. In 2017, prior to a grant application, we published a pre-protocol for a three-country study to test the scale-up of primary health care-based programmes to identify and manage the harmful use of alcohol and comorbid depression[60]. Since the application, and during the grant negotiation and planning phase, the design of the study has changed considerably, essentially moving from a two-arm design to a four-arm design, and changing the primary outcome measure to the proportion of the adult population registered with a PHCU that has their alcohol consumption

For peer review only - http://bmjopen.bmj.com/site/about/guidelines.xhtml

measured, Supplement File 1, Box 1. With all changes approved by the concerned ethics committee, this paper outlines the final protocol for a quasi-experimental study to test the implementation of primary health care-based measurement, advice and treatment for heavy drinking and comorbid depression at the community level in three Latin American countries, Colombia, Mexico and Peru (SCALA study).

Intervention municipal areas are investigator-selected from Bogotá (Colombia), Mexico City (Mexico) and Callao – Lima (Peru). Control municipal areas are investigator-selected in the same cities, on the basis of comparability with the intervention municipal area in terms of socio-economic and other characteristics which impact on drinking, health care and survival, comparable community mental health services, and sufficient geographical separation to minimize spill over effects from the intervention municipal area. Randomized selection of the municipal areas was not feasible due to organizational limitations. Municipal areas are chosen as a scalable implementation unit at mesosystem level that can be replicated as the intervention is scaled-up[40], given their jurisdictional responsibilities for prevention and health care services.

Within each intervention municipal area, a local Community Advisory Board (CAB) is created of key stakeholders, including representatives of local and regional government, directors of primary health care services, non-governmental organizations active in providing counselling and treatment services for alcohol and mental health, academic experts, and local media. The CABs meet regularly during the course of the study, giving advice on tailoring materials for local use, giving advice on adoption mechanisms, support systems and communication campaigns to support the action, and preparing for sustainability and scale-up at the end of the action.

The units of allocation and analysis, i.e., study participants, are 54 primary health care units (PHCUs) and the providers working in them. Within each PHCU, eligible providers include any fully trained health care provider working in the PHCU and involved in medical and/or preventive care. The providers sign an informed consent for their participation. The overall study design is summarized in Figure 1. Fifty-four PHCU are invited to join the study until 27 are achieved within each of the two municipal areas (intervention and control) across the three countries (nine per municipal area within each of the three countries).

Within each intervention municipal area, a User Panel is created of providers and patients drawn from the primary health care centres to advise on the tailoring of patient and provider materials and on provider training programmes.

Figure 1 here

Figure 1 Study flow diagram

For the first six months of the 18-month implementation and test period, a four-arm design is adopted, Figure 2. Within the comparator municipal area, twelve PHCUs out of the 27 are randomly allocated to control (Arm 1), and 15 are allocated to receive short training to implement a short clinical package

For peer review only - http://bmjopen.bmj.com/site/about/guidelines.xhtml

(Arm 2). Within the intervention municipal area, in which all 27 PHCU receive municipal action, 15 PHCUs are randomly allocated to receive short training to implement a short clinical package (Arm 3), and twelve PHCUs are allocated to receive standard training to implement a standard clinical package (Arm 4). Random allocation was undertaken using Excel random number generator.

Figure 2 here

Figure 2. Study design for the first six months of the 18-month implementation period

The clinical package comprises measurement instruments, patient information and advice material, and provider guidance material, with the differences between the standard and short clinical materials are described in Supplement File 1, Table 1, with references. Supplement File 1, Table 1 also lists the material used in control Arm 1. The standard material is essentially that used in common clinical practice[60] and the short version a simplified version deliverable in practice during a short period of time. The packages include measurement instruments and patient advice material for comorbid depression implemented with patients with an AUDIT-C score of 8+. Supplement File 1, Table 1 summarizes the differences between the standard and short versions of the training programme.

The standard and short care pathways that are implemented are summarized in Supplement File 1, Figures 1 and 2.

Essentially, in all arms, primary health care providers are asked to measure the alcohol consumption of all adult patients who consult for whatever reason using AUDIT-C. The three AUDIT-C questions are included in a paper tally sheet completed by the provider, in which the providers document the outcome of the consultation (advice given, patient referred etc.). The local researchers visit each PHCU on a two to four weekly basis to collect completed tally sheets and deliver new tally sheets as required. The local researchers collect information on the total number of adult patients (aged 18+ years) registered with each PHCU and the monthly number of total adult consultations with each provider. Patients who score <8 with AUDIT-C are given a patient information leaflet. Patients who score 8+ with AUDIT-C are assessed and manged as appropriate for depression, and are advised to reduce their alcohol consumption, unless there are clinical indications for referral. Arm 4 differs from Arm 3 in having a lengthier assessment, if indicated, and a longer session of advice giving.

By Month 6, Hypotheses 3, i.e., non-superiority of Arm 4 (standard package with municipal action and standard training) over Arm 3 (short package with municipal action and short training) will be tested. In the presence of clinical equivalence of a relative difference of the primary outcome, i.e., the cumulative coverage of patients whose alcohol consumption is measured, of less than 10%, Arm 4 will

For peer review only - http://bmjopen.bmj.com/site/about/guidelines.xhtml

be replaced by Arm 3 from month 8 onwards, Figure 3.

Figure 3 here

Figure 3. Study design from month 8 onwards, assuming no superiority of Arm 4 over Arm 3 during first six months of implementation.

The municipal integration and support inputs to Arms 3 and 4 within the intervention municipal area are summarized in Supplement File 1, Table 2, with references. Municipal integration and support comprises:

i. Creation of local Community Advisory Boards of local stakeholders to advise on tailoring of materials, support local implementation and review drivers of successful action;
ii. Appointment of local project champion to advocate for successful implementation of programmes;
iii. Implementation of five evidence-based adoption mechanisms;
iv. Implementation of five evidence-based support systems; and
v. Implementation of community-based communication campaigns.

Tailoring

The CABs and UPs review and tailor relevant materials of the clinical package and training courses and of the municipal integration and support inputs within the seven domains of: (i) local and national guideline factors; (ii) individual health care provider factors; (iii) patient factors; (iv) interactions between different professional groups; (v) incentives and resources; (vi) capacity for organizational change; and, (vii) social, political and legal factors[61-63].

The study timetable is summarized in Figure 4. The data management plan, as submitted to the European Commission, is available as Supplement File 2.

Figure 4 here

Figure 4. Study timetable.

For peer review only - http://bmjopen.bmj.com/site/about/guidelines.xhtml

Data collection and instruments

1. **During set-up phase for Arms 1-4**

Municipal level information

At the level of the municipal area (or, when not available, at whole city, regional or country level), the following information will be collected from routinely available data on socio-demographic factors, alcohol and mental health data, health system structures, quality of life, sustainable governance and values, Supplement File 1, Table 3.

PHCU and provider level information

All contacted PHCU, including those who did and did not agree to be part of the study, will provide information on:

- Numbers of registered patients, divided into age 0-17 years and 18+ years; and,
- Numbers and professions of provider staff (including physicians, nurses, nurse technicians, midwifes, psychologists, social workers, and others).

At recruitment, PHC providers will provide data on their:

- Age;
- Gender;
- Profession (doctor, nurse, practice assistant etc.);
- Time worked in the PHC;
- data on their attitudes and experiences to working with patients with heavy drinking and comorbid depression (Supplement File 1, Table 4).

Since we are unable to randomize the municipal areas involved, we will use propensity score matching (PSM) based on data collected at the level of the municipal area and the PHCU, to take into account potential confounding variables between control and intervention municipal areas, and minimise bias on account of these.

2. **During one-month baseline measurement period for Arms 1-4**

Provider-based measurement and assessment of alcohol consumption and comorbid depression and record of advice and treatment given (tally sheets)

Based on the validated methodology of the ODHIN project[22,24], PHC providers will document activity by completing anonymous paper tally sheets that record eligible patients' (aged 18+ years) AUDIT-C scores[64], and, if administered (as documented in Supplement File 1, Table 1), AUDIT-10[65], PHQ-2[66] and PHQ-9[67] scores, and the advice or treatment given to each patient. The tally sheets will record the age, sex, and educational level of the patient, the latter as a proxy measure of socio-economic status. PHCUs will return data on the number of adult (aged 18+ years) consultations per provider for the one-month baseline measurement period.

For peer review only - http://bmjopen.bmj.com/site/about/guidelines.xhtml

3. During training prior to implementation for Arms 2-4

Providers will complete a short questionnaire after the initial training sessions. The questionnaires, which are adapted based on specific training contents (standard or short package), will assess the participants' experience of the training, measuring satisfaction with the components of the training aspects, as well as their perceived utility. Two measures included in the main provider questionnaires, SAAPPQ[68] and self-efficacy[69], will be included in order to assess the specific impact of the training, independent of the effect of the implementation of the intervention.

4. During 18-month implementation period for Arms 1-4

Provider-based measurement and assessment of alcohol consumption and comorbid depression and record of advice and treatment given (tally sheets)

The same mechanism, for tally sheets used during the baseline measurement period will continue for each calendar month of the 18-month implementation period. Monthly data will be collected and reported with accumulation of coverage over time. Formal reporting will be undertaken at baseline, and for coverage achieved by month 12 and by month 18 of the 18-month implementation and test period. Tally sheets will include an identifying code of the provider, PHCU, country and study arm, but no identifying code of the patient. Data will be extracted and sent to the project's data warehouse at Technical University Dresden on a monthly basis.

Extended Tally Sheets

As part of quality control, in all four Arms at two time points, during the 18-month implementation and test period (months 3 and 15), providers will complete extended tally sheets on two separate days in each month. The extended tally sheets will include an identifying code of the provider but no identifying code of the patient. The extended tally sheet will include: additional information from the patient on alcohol knowledge[70], social norms[71] and health literacy[72] applied to alcohol, as it informs the content of advice given; and, additional information from the provider on contextual characteristics that informed their advice giving. The extended tally sheets will include a consent form for the patient and self-completed additional questions for the patient to complete, once the consultation has ended.

Self-completed additional questions by patient

On two separate days, during months 3 and 13, coinciding with and following the consultation with the provider using the extended tally sheet, patients who are able to read and write will be invited to give consent to self-complete additional questions to the extended tally sheet in the waiting room before leaving the PHCU, handing the completed tally sheet and questions to a researcher in attendance. No patient identifying information will be included in the questionnaires. Six domains, serving as quality control, will be included:

i. AUDIT-C[64];
ii. PHQ-2[66];
iii. Experiences of the consultation;
iv. Views on being asked about alcohol consumption;

For peer review only - http://bmjopen.bmj.com/site/about/guidelines.xhtml

v. Health Literacy[72] as it applies to alcohol; and,

vi. Exposure to communication and media campaigns on alcohol.

On each day, 270 patient questionnaires will be collected across all PHCUs, with up to 1080 (540 during each of months 3 and 13) questionnaires completed in total across the four days.

Provider-based attitudes and experiences.

At two time points during the 18-month implementation period (months 3 and 13), providers will provide data on their attitudes and experiences to working with patients with heavy drinking and comorbid depression, Supplement File 1, Table 4.

Providers will complete a short questionnaire after each of the booster training sessions that they attended (at months 4 and 8). The specific content, number and timing of the training-related questionnaires will depend on the study arm: Arm 2 and 3 participants will fill in one questionnaire after the booster session; while Arm 4 participants will fill in two after each of the two booster sessions.

Observations

The training sessions with the primary health care providers, and the meetings of the CABs will be observed by a neutral observer in order to take note of additional possible barriers in the implementation of the protocol that emerge through the training sessions and meetings. Participant responsiveness will also be observed.

Economic data for return-of-investment analyses

Within SCALA, we will conduct return-on-investment (RoI) analyses, by assessing for each EURO invested in scaling up delivery of screening and brief interventions in primary health care in Columbia, Mexico, and Peru, how many EUROs will be saved by reductions in future health care utilization. The return of investment will be defined as the [return on investment = (gain from investment – cost of investment) / cost of investment]. For details on the data required for RoI analyses, Supplement File 1, Table 5.

For the RoI analyses, the effects of increased coverage of alcohol brief advice among primary health care patients will be modelled using effect sizes from previous meta-analyses[52, 73]. To translate the reduced intake of alcohol into health gains, we will calculate alcohol-attributable fractions for major disease and injury categories. These fractions will then be applied to the cost data outlined in Supplement File 1, Table 5 to estimate the alcohol-attributable costs per disease category.

For peer review only - http://bmjopen.bmj.com/site/about/guidelines.xhtml

Process evaluation

As the intervention is embedded in a complex system involving actions and actors at different levels (individual, organisational, municipal), a thorough process evaluation will be carried out to complement and better understand the outcomes. Through the process evaluation, the implementation with its fidelity and adaptation will be assessed, along with the drivers of scale-up and contextual factors influencing the implementation, the drivers, and the outcomes. This will be achieved in four blocks: driver diagram creation; barriers and facilitators analysis; assessment of implementation, mechanisms of impact and context; and, further contextual and policy analysis.

Key informant interviews

A number of individual or group interviews will be undertaken throughout the project with key stakeholders – providers, user panel members, CAB members, municipal and primary health care-based clinical leaders, project partners, and any other people involved in the implementation of the SCALA project. Depending on the stakeholder and their involvement in the project, the topics of the interviews will cover topics such as the necessary adaptation to the protocol; the experience of implementing the programme in primary health care practice; and the perception of the municipal support and the community campaigns.

Driver diagrams

Driver diagrams[74] will be used in order to describe the intervention and its causal assumptions, providing the theory of change through displaying what contributes to intervention aim and what are the relationships between primary drivers, secondary drivers and specific change ideas/activities. The initial general driver diagram, Supplement File 1, Figure 3, will be modified based on local contexts and adapted throughout the duration of the project in order to understand how scale up varies in the different cities.

Barriers and facilitators assessment

Factors influencing the implementation of the SCALA protocol will be assessed before the implementation, as well as monitored throughout. The anticipated barriers and facilitators to implementation will be assessed through development of evaluation tool based on literature review[75-77] and implementation framework[61], with subsequent refinement and adaptation to the local context through focus group discussions and workshops with the CABs. The aim of the tool is to identify the barriers that would have to be addressed and monitored throughout implementation and the facilitators that would incentivize and engage providers and the PHCU unit managers in uptake and scaling up of the SCALA protocol. The experienced barriers and facilitators will be further monitored through meeting observations, provider questionnaires and interviews, as well as interviews with other involved stakeholders (e.g. CAB members, PHCU managers).

Implementation, mechanisms of impact and context

The factors influencing the progress from scale-up to outcomes will be identified and documented based on UK Medical Research Council guidance[70], analysing factors within five groups: (i) description of intervention and its causal assumptions; (ii) implementation; (iii)mechanisms of impact ; (iv)context

For peer review only - http://bmjopen.bmj.com/site/about/guidelines.xhtml

; and, (v) outcomes. All aspects of the intervention will be taken into consideration: the intervention, intervention tailoring, training, training tailoring, as well as the municipal action, consisting of the CABs and the communication campaign, combining both quantitative and qualitative methods in order to obtain a comprehensive picture of the integration and interaction of included variables. A detailed description of the topics of interest and accompanied methods is presented in Supplement File 1, Table 6.

The five groups will be assessed as follows:

i. *Description of the intervention.* The description of the intervention and its causal assumptions draws from the previously described driver diagram;
ii. *Implementation.* Delivery of the training will be assessed though document analysis (reports from training), observation and self-reports from the trainers. Delivery of the intervention will be assessed through document analysis, interviews with patients and providers. The areas of focus will be fidelity, adaptation, dose and reach. Implementation of the CAB meetings and community action will be assessed mainly through document analysis, as well as key informant interviews;
iii. *Mechanisms of impact*. The following three areas will be covered: participant responses to the intervention, mediators and unintended consequences. Mechanisms of impact of intervention delivery will be assessed through patient and providers' questionnaires. The patient interviews will focus on their responsiveness to the intervention, specifically looking at perceived acceptability. In order to evaluate participants' responses to the training, a post-training questionnaire examining satisfaction with the training and perceived utility of training sessions will be applied, triangulated with data from observation and trainers' self-report. Additionally, providers' self-efficacy will be tested as potential mechanism of impact that links the implementation to the outcomes. Mechanisms of impact of the CAB meetings and community action will be examined through key informant interviews and questionnaires. Specific focus will be placed on perceptions and mechanisms of actions of the communication campaign, examining its effect on attitudes and social norms of both providers and patients;
iv. *Context.* Contextual factors that should be considered in order to better understand the success of the intervention will be assessed through meeting observation, document analysis, and provider questionnaires, as well as stakeholder interviews, with the main focus primarily on individual and organisational level characteristics of the context. For the training evaluation, context will be assessed through observation and trainers' self-report. Context of municipal level actions will be assessed through key informant interviews. Additionally, contextual and policy factors on national and municipal levels will be assessed as described below.
v. *Outcomes.* The data collected through process evaluation will be combined with the outcomes and presented within the RE-AIM framework[79-81], evaluating SCALA's impact across the dimensions of reach, effectiveness, adoption, implementation and maintenance.

Contextual and policy factors

Based on methodology of Ysa et al[82], contextual and policy factors on national and municipal level will

For peer review only - http://bmjopen.bmj.com/site/about/guidelines.xhtml

be identified through document analysis and key informant interviews. The main variables considered for contextual analysis will be: (1) available data similar to that of the OECD better life initiative[83]; (2) Sustainable Governance Indicators[84]; and, (3) World Values Survey data[85]]. For policy analysis, the information sought will be for a for alcohol policy-related strategies, action plans, legislation and evaluations, both on country and municipal level. The existing contextual and policy factors will be mapped onto the test of the scale-up of the SCALA package to describe and identify those factors on national and municipal level that might influence going to full-scale beyond the tested scalable units.

Outcomes

Primary outcome:

The primary outcome will be the cumulative proportion of the number of adults (aged 18+ years) registered with the PHCU that have their alcohol consumption measured with a completed AUDIT-C instrument during the study period (coverage). The number of adults registered is provided by the administrative office of the PHCU and includes all adult patients covered by the PHCU, whether or not they consult during the 18-month implementation test period.

Secondary outcomes:

- **Proportion of consulting patients who have their alcohol consumption measured by AUDIT-C:** Calculated as the number of adults who have their alcohol consumption measured by AUDIT-C divided by the total number of adults who consult the PHCU during the same time period per participating provider and per PHCU;
- **At risk population receiving advice and/or treatment for heavy drinking:** Calculated as the number of adults with an AUDIT-C score of 8+ who receive brief advice and/or referral for their heavy drinking divided by the total number of patients with an AUDIT-C score of 8+ per participating provider and per PHCU. Information will also be collected on the number of patients with an AUDIT-C score of <8 who receive brief advice and/or treatment for their heavy drinking;
- **Proportion of patients with AUDIT-C score of 8+ who receive assessment for depression:** Calculated as the number of consulting adults with an AUDIT-C score of 8+ who complete PHQ-2 divided by the total number of patients with an AUDIT-C score of 8+ per participating provider and per PHCU;
- **At risk population receiving advice and/or treatment for comorbid depression:** Calculated as the number of adults with a PHQ-2 score of 3+ who receive a patient leaflet and/or referral for their depression divided by the total number of patients with a PHQ-2 score of 3+ per participating provider and per PHCU; and,
- **Provider attitudes:** Attitudes of the participating providers will be measured by the short version of the Alcohol and Alcohol Problems Perception questionnaire, SAAPPQ [64]. The responses will be summed within the two scales of role security and therapeutic commitment. Individual missing values for any of the items in a domain will be assigned the mean value of the remaining items of the domain before summation.

For peer review only - http://bmjopen.bmj.com/site/about/guidelines.xhtml

Statistical tests of key hypotheses

Primary study goal: Multilevel regression analyses will be undertaken at 12 months' time of the implementation test period, using cumulative results at months 1-12, and at 18 months' time using cumulative results months 1-18. Both analyses will include co-variates of country and results during baseline month, analysed at the levels of the PHCU by study arm, taking into consideration the hierarchical nature of the data. For any PHCU that drops out during the study, outcome values for subsequent measurement points will be set at the last value obtained.

Hypothesis 1
Municipal action leads to more sustainable coverage amongst PHCU that receive training. We will compare results on primary outcome after 18 months with results after 12 months between Arms 3 and 4 versus Arm 2 via regression.

Dependent variables:

- For each PHCU, cumulative results of months 1-18 of number of patients whose alcohol consumption is measured with AUDIT-C per 1,000 registered patients; and cumulative results of months 1-12 per 1,000 registered patients.

Random effects:

- Country as random intercept (test for inclusion)

Independent variables:

- Proportion of consulting patients who have their alcohol consumption measured with a completed AUDIT-C instrument during the baseline measurement month
- Condition:
    - Municipal action (yes vs. no)
- Covariate:
    - Proportion of consulting patients who have their alcohol consumption measured with a completed AUDIT-C instrument during the baseline measurement month

It is postulated that coverage for Arms 3 and 4 will be significantly higher than for Arm 2.

Hypothesis 2
Training leads to higher coverage than no training. For both months 1-12 and months 1-18, compare cumulative coverage as per primary outcome between Arms 1 and 2 via multilevel regression analyses.

Dependent variable

- Cumulative results months 1-12, and cumulative results months 1-18 of number of patients

For peer review only - http://bmjopen.bmj.com/site/about/guidelines.xhtml

whose alcohol consumption is measured with AUDIT-C per 1,000 registered patients with

- PHCU

Random effects:

- Country as random intercept (test for inclusion)

Independent variables:

- Condition:
  - Training (Arm 2 vs. Arm 1)
- Covariate:
  - Proportion of consulting patients who have their alcohol consumption measured with a completed AUDIT-C instrument during the baseline measurement month

It is postulated that coverage for Arm 2 will be significantly higher than for Arm 1.

Hypotheses 3

In the presence of municipal action, the short clinical package and short training do not lead to less coverage than the standard clinical package and standard training. In the presence of clinical equivalence of a relative difference of cumulative coverage of patients screened by less than 10% by month 6, the difference between Arm 3 (all 15 PHCU across the three countries) and Arm 4 (all 12 PHCU across the three countries) will be assessed with regression analyses. If Arm 4 is not superior to Arm 3, both arms will be collapsed into Arm 3 (shorter package) from month 8 onwards.

Dependent variable

- Cumulative results months 1-6 per 1,000 patients

Random effects:

- Country as random intercept (test for inclusion)

Independent variables

- Condition:
  - Length of clinical package (longer = arm 4 vs. shorter = arm 3)
- Covariate:
  - Proportion of consulting patients who have their alcohol consumption measured with a completed AUDIT-C instrument during the baseline measurement month

It is postulated that Arm 4 is not significantly superior to Arm 3.

Sample size calculations for main hypothesis

As the outcome of the primary study goal is predicted to be Arm3 > Arm2 > Arm1, we compared both Arm 2 > Arm 1, and Arm 3 > Arm 2.

For peer review only - http://bmjopen.bmj.com/site/about/guidelines.xhtml

Our power calculations are based on the following assumptions: given an average size of a PHCU of approximately 15,000 adults, with an average of 1500 new consultations per month, we expect a cumulative coverage after 12 months of 0.0325 of the registered adult population to have had their alcohol consumption measured in the control condition (Arm 1) (data extrapolated from month 3 and month 9 assessments of control group from ODHIN study[22,24]; Anderson, personal communication). For the short clinical package and short training (Arm 2), we expect this to increase to 0.075 (data extrapolated from month 3 and month 9 assessments of training group from ODHIN study[22,24]; Anderson, personal communication). Although the WHO Phase IV study predicts an additional beneficial impact of municipal support[41], precise empirical data is not available – however, we consider an estimate for Arm 3, with municipal support, to be 0.15, a proportion that would need to be achieved to consider municipal support to be worthwhile. To detect the difference between Arm 2 and Arm 1, assuming a design effect of 15 PHCUs (clusters) across the three municipal areas in Arm 2, with 15,000 patients (items), and 12 PHCUs (clusters) in Arm 1, with 15,000 patients (items), with an ICC for PHCUs of 0.03 (data from ODHIN study[22,24]; Anderson, personal communication) we would have 82% power at a significance level of 5%[86]. For the difference between Arm 3 and Arm 2 (15 PHCUs/clusters in each arm), we would have 96.5% power.

Patient and public involvement

Patients were not involved in the design of the study but are involved in the tailoring processes. Existing literature suggests that most patients find it acceptable for primary health care providers to ask about their drinking using validated measurement instruments, and support the delivery of brief advice to those drinking above recommended levels[87-95]. However, the majority of the evidence to date draws on research conducted in Europe, and thus the findings are potentially less transferable to Latin American populations. In order to ensure the design and content of the intervention package, including related outcome measures, are appropriate for implementation in the target SCALA sites, we work closely with patients in each city to tailor patient materials. Within the intervention municipal areas in each of the three countries, User Panels are created with representatives of patients from the primary health care centres. As part of the tailoring process, people and patients within the User Panels have the opportunity to comment on the materials and information designed for use by patients. The results of the study will be disseminated directly to patients and the public through information made available via the primary health care units.

, people and patients

ETHICS AND DISSEMINATION

This protocol outlines a quasi-experimental study[1] to test the extent to which embedding PHC-based measurement and brief advice activity within supportive municipal action leads to improved scale-up of an intervention package, with more patients having their alcohol consumption measured, and with heavy drinkers receiving subsequent appropriate advice and treatment. It is not envisaged that there will be any substantial protocol modifications during the course of the study. Any modification to the

For peer review only - http://bmjopen.bmj.com/site/about/guidelines.xhtml

protocol will be described will be described in all scientific publications.

The Ethics Committee of the Technical University of Dresden gave final ethical approval for the SCALA project on 12 April 2019, EK90032018. All participating primary health care units and participating primary health care providers sign an informed consent form for participation with the country-based research team. Selected patients at two separate time points sign an informed consent form with the country-based research team to provide additional anonymized information following a consultation with a primary health care provider. The consent forms are included within Annexe Data Management Plan. All data collection, processing, and sharing procedures will adhere to national and international laws including the General Data Protection Regulation (EU Regulation 2016/679), as described within the Annexe Data Management Plan.

All materials are publicly available on the project website: https://www.scalaproject.eu/. According to the SCALA data management plan, by default, all quantitative datasets generated in the course of the SCALA study will be made openly available through the UK Data Service upon publication of the results (http://www.data-archive.ac.uk/). Prior to publication, all data will be formatted to meet UK Data Service requirements.

Ministries of Health at municipal and country levels are represented in the Community Advisory Boards created in each intervention municipality to facilitate scale-up at municipal and country levels, once the implementation strategy is validated. SCALA works closely with the Pan American Health Organization (PAHO), with the principal investigator form Mexico being a Collaborating Centre with PAHO, to facilitate scale-up at Latin American levels, once the implementation strategy is validated.

DISCUSSION

The study has several features worth mentioning. It:

1. uses a theory-based approach[61-63] to tailoring clinical materials and training programmes, creating city-based Community Advisory Boards, and user-based User Panels to ensure that tailoring matches user needs, municipal services[96], and co-production of health[97];
2. sets a higher cut-off score for AUDIT-C (8+) than is commonly used to trigger advice-giving, matching definitions of heavy drinking[98, 99], and similar to baseline levels of alcohol consumption in PHC-based trials to reduce heavy drinking[52]. We set the same cut-offs for men and women, based on epidemiological evidence[100], and to minimize unintended consequences of using different cut offs for men and women[101]. We recognize the importance of comorbid depression by building in identification, management, and referral mechanisms[57-59];
3. tests for non-superiority of implementing a standard measurement and 5-minute brief advice intervention with six hours of training, compared with implementing a shorter 1-minute brief advice intervention with three hours of training, taking into account that brief advice is as effective and cost-effective as more extended advice or treatment in reducing heavy drinking[55, 102, 103], and the need for very brief clinical and training programmes for time-constrained providers;

For peer review only - http://bmjopen.bmj.com/site/about/guidelines.xhtml

4. tests the added value of embedding and implementing PHC activity within municipal-based adoption mechanisms and support systems[40], and communication campaigns over and above training programmes solely directed to primary health care providers;
5. has a longer time frame (18 months) than is traditionally used in implementation studies[104, 105], to assess longer term impacts; and,
6. gives considerable emphasis to process evaluation[78], developing logic models to document the fidelity of all implementation strategies, and to identify, the drivers and barriers and facilitators to successful implementation and scale-up, and the political and economic contextual factors that might influence scale-up.

There are some limitations to the study design. A trial with random assignment of municipal areas is not feasible due municipal-based political and technical considerations. As we are unable to randomize the involved municipal areas, we adopt a quasi-experimental design[1], trying to optimize control municipal areas for confounding, and by using propensity score matching (PSM). While full comparisons via randomization, and thus establishment of causality, are not possible, together with the qualitative evaluation component of the study, we will be able to clearly identify the mechanisms which were crucial in leading to the outcomes. According to a recent 7-item checklist for classifying quasi-experimental studies for Cochrane reviews[106], our approach is, nevertheless, ranked as a strong design, Supplement Table 7.

Although our focus on embedding PHC activity within supportive municipal actions is hypothesized to increase measurement and brief activity over and above that previously demonstrated, such an approach also brings risks. Municipal and national governments change; and, thus health priorities may change. Although our approach minimizes the need for extra resources (and in some jurisdictions, could be resource saving[19], it is not resource free. Funding constraints could limit future scale-up and sustainability.

We have based our protocol adopted on a model of transdisciplinary research to promote sustainability. Such a model identifies, structures, analyses, and deals with specific problems in a way that grasps the complexity of problems[107]; it takes into account the diversity of real-world and scientific perceptions of problems; and develops knowledge and practices that promote what is generally accepted to be the common good[108]. As such, we include municipalities and health systems as stakeholders to form explicitly orchestrated and managed ecosystems that cross organizational boundaries. Municipal areas and health systems create an engagement platform that provides the necessary environment, including people and resources, for sustainability.

For peer review only - http://bmjopen.bmj.com/site/about/guidelines.xhtml

DECLARATIONS

Ethics approval and consent to participate

The Ethics Committee of the Technical University of Dresden gave final ethical approval for the SCALA project on 12 April 2019, EK90032018. All participating primary health care units and participating primary health care providers sign an informed consent form for participation. Selected patients at two separate time points sign an informed consent form to provide additional anonymized information following a consultation with a primary health care provider.

Consent for publication

No individual person's data will be published in any form.

Availability of data and materials

All materials are publicly available on the project website: https://www.scalaproject.eu/. According to the SCALA data management plan, by default, all quantitative datasets generated in the course of the SCALA study will be made openly available through the UK Data Service upon publication of the results (http://www.data-archive.ac.uk/). Prior to publication, all data will be formatted to meet UK Data Service requirements.

Competing interests

None declared

Funding

The research leading to these results or outcomes has received funding from the European Horizon 2020 Programme for research, technological development and demonstration under Grant Agreement no. 778048 – Scale-up of Prevention and Management of Alcohol Use Disorders and Comorbid Depression in Latin America (SCALA). Participant organisations in SCALA can be seen at: www.scalaproject.eu. The views expressed here reflect those of the authors only and the European Union is not liable for any use that may be made of the information contained therein. The Funder was not involved in the study design. The funder will not be involved in the collection, analysis, interpretation of data, and preparations of any publication.

Authors' contributions

EJL, PA, MP, AO'D, AG, BS, APG, HdV, GNR, DK, IVB, FB, JMT, AS, APdL, EK, SM, JM, LM, HLP, GR, CS, and JR contributed to the Grant Application, on which this protocol is based and adapted. EJL drafted the first version of the paper, and revised the paper based on author's feedback and comments. PA prepared the paper and material for submission and undertook the submission process. EJL, PA, MP, AO'D, AG, BS, APG, HdV, GNR, DK, IVB, FB, JMT, AS, APdL, EK, SM, JM, LM, HLP, GR, CS, and JR commented on drafts of the manuscript and read and approved the final version. PA undertook random allocation generation. APG and JMT assigned PHCU to arms in Colombia; GNR and APdL assigned PHCU to arms in Mexico; MP and IVB assigned PHCU to arms in Peru.

For peer review only - http://bmjopen.bmj.com/site/about/guidelines.xhtml

Acknowledgements
Not applicable

References

01. Shadish WR, Cook TD, Campbell DT: Experimental and quasi-experimental designs for generalized causal inference. 2nd edition. Houghton Mifflin, Michigan,2002. Reference Source

02. World Health Organization. (2018). Global Status Report on Alcohol and Health 2018. Geneva, Switzerland: WHO Press.

03. Rehm J, Gmel GE, Gmel G et al. The relationship between different dimensions of alcohol use and the burden of disease - an update. Addiction 2017 doi:10.1111/add.13757

04. Odlaug BL, Gual A, DeCourcy J, Perry R, Pike J, Heron L, Rehm J. Alcohol dependence, co-occurring conditions and attributable burden. Alcohol and Alcoholism 2016 51 201-209.

05. Bellos S, Skapinakis P, Rai D et al. Longitudinal association between different levels of alcohol consumption and a new onset of depression and generalized anxiety disorder: Results from an international study in primary care. Psychiatry Research 2016 243 30-34.

06. Boden J. M., Fergusson D. M. Alcohol and depression. Addiction 2011; 106: 906–14.

07. Probst C, Roerecke M, Behrendt S, Rehm J. Socioeconomic differences in alcohol-attributable mortality compared with all-cause mortality: A systematic review and meta-analysis. *Int J Epidemiol* 2014; **43**(4): 1314-27

08. Kontis V, Mathers, CD, Rehm J, Stevens G A, Shield K D, Bonita R, et al. Contribution of six risk factors to achieving the “25×25” NCD mortality reduction target. Lancet 2014 http://dx.doi.org/10.1016/ S0140-6736(14)60616-4.

09. Kontis V, Mathers CD, Bonita R et al. Regional contributions of six preventable risk factors to achieving the 25 × 25 non-communicable disease mortality reduction target: a modelling study. *Lancet Glob Health* 2015; 3: e746–57

10. United Nations General Assembly. Political declaration of the High-level Meeting of the General Assembly on the Prevention and Control of Non-communicable Diseases. http://www.un.org/en/ga/ncdmeeting2011/ Acessed 01/02/2017

11. GBD 2015 SDG Collaborators. Measuring the health-related Sustainable Development Goals in 188 countries: a baseline analysis from the Global Burden of Disease Study 2015. Lancet 2016 388 1813-50.

12. Shield, K.D., Manthey, J., Rylett, M., Probst, C., Wettlaufer, A., Parry, C.D.H. & Rehm, J. (2020). National, regional, and global burdens of disease from 2000 to 2016 attributable to alcohol use. Lancet Public Health, 5(1), e51–61. doi: 10.1016/S2468-2667(19)30231-

13. Economic Dimensions of Noncommunicable Diseases in Latin America and the Caribbean: Policy Agenda Considerations 143 George Alleyne. In:

For peer review only - http://bmjopen.bmj.com/site/about/guidelines.xhtml

http://iris.paho.org/xmlui/bitstream/handle/123456789/28501/9789275119051_eng.pdf?sequence=1&isAllowed=y

14. Levin C, Chisholm D. Cost-Effectiveness and Affordability of Interventions, Policies, and Platforms for the Prevention and Treatment of Mental, Neurological, and Substance Use Disorders. In Patel, V., D. Chisholm., T. Dua, R. Laxminarayan, and M. E. Medina-Mora, editors. 2015. *Mental, Neurological, and Substance Use Disorders*. Disease Control Priorities, third edition, volume 4. Washington, DC: World Bank. doi:10.1596/978-1-4648-0426-7.

15. O'Donnell, A., Anderson, P., Newbury-Birch, D., Schulte, B., Schmidt, C., Reimer, J. & Kaner, E. The Impact of Brief Alcohol Interventions in Primary Healthcare: A Systematic Review of Reviews. Alcohol and Alcoholism doi: 10.1093/alcalc/agt170.

16. Angus C, Thomas C, Anderson P, et al. Estimating the cost-effectiveness of brief interventions for heavy drinking in primary health care across Europe. Eur J Public Health. 2017;27:345–351.

17. Rehm J & Barbosa C. (2018) The cost-effectiveness of therapies to treat alcohol use disorders, Expert Review of Pharmacoeconomics & Outcomes Research, 18:1, 43-49, DOI: 10.1080/14737167.2018.1392241

18. Rehm, J., Shield, K. D., Gmel, G., Rehm, M. X., & Frick, U. (2013). Modeling the impact of alcohol dependence on mortality burden and the effect of available treatment interventions in the European Union. European Neuropsychopharmacology, 23(2), 89–97.

19. Organisation for Economic Co-operation and Development. *Tackling Harmful Alcohol Use: Economics and Public Health Policy*. Paris: Organisation for Economic Co-operation and Development, 2015.

20. Anderson, P., M. Laurant, E. Kaner, M. Wensing and R. Grol (2004). "Engaging general practitioners in the management of hazardous and harmful alcohol consumption: results of a meta-analysis." J Stud Alcohol 65(2): 191-199.

21. Keurhorst M, van de Glind I, Bitarello do Amaral-Sabadini M, Anderson P, Kaner E, Newbury-Birch D, Braspenning J, Wensing M, Heinen M, Laurant M (2015) Determinants of successful implementation of screening and brief interventions for hazardous and harmful alcohol consumption in primary healthcare. A systematic review and meta-regression analysis. Addiction Addiction 110:877–900.

22. Anderson, P., Gual, T., Coulton, S., Kaner, E., Bendsten, P., Kłoda, K., Reynolds, J., Keurhorst., M. N., Segura, Wojnar, M., Mierzecki, A., Deluca, P., Newbury-Birch, D., Parkinson, K., Okulicz- Kozaryn, K., Drummond, C., Laurant, M. (2017) Improving the delivery of brief interventions for heavy drinking in primary health care: nine month outcomes of the ODHIN five country cluster randomized factorial trial. Annals of Family Practice. In Press.

23. Anderson, P., E. Kaner, S. Wutzke, M. Funk, N. Heather, M. Wensing, R. Grol, A. Gual, L. Pas and W. H. O. B. I. S. Group (2004). "Attitudes and managing alcohol problems in general practice: an interaction analysis based on findings from a WHO collaborative study." Alcohol Alcohol 39(4): 351-356.

For peer review only - http://bmjopen.bmj.com/site/about/guidelines.xhtml

24. Anderson, P., Bendsten, P., Spak, F., Reynolds, J., Drummond, C., Segura, L., Keurhorst., M. N., Palacio-Vieira, J., Wojnar, M., Parkinson, K., Colom, J., Kłoda, K., Deluca, P., Baena, B., Newbury-Birch, D., Wallace, P., Heinen, M., Wolstenholme, A., van Steenkiste, B., Mierzecki, A., Okulicz-Kozaryn, K., Ronda, G., Kaner, E., Laurant, M., Coulton, S., Gual, T. (2016) 'Improving the delivery of brief interventions for heavy drinking in primary health care: outcome results of the ODHIN five country cluster randomized factorial trial' Addiction DOI: 10.1111/add.13476.

25. Carlos Campillo Serrano, Rosa Díaz Martínez, Martha P. Romero Mendoza, Juan Cerrud Sánchez, Jorge Villatoro (1992). La intervención eficaz del médico general en el tratamiento de bebedores cuyo hábito alcohólico representa un riesgo para su salud o ya les ha ocasionado algún daño. (Preliminary results of a prospective double blind clinical trial). *Salud mental*, 15 (2).

26. Hoffman KA, Beltrán J, Ponce J, García-Fernandez L, Calderón M, Muench J, et al. Barreras para implementar el despistaje, intervenciones breves y referencia al tratamiento por problemas de consumo de alcohol y otras drogas en hospitales que atienden personas que viven con el VIH/SIDA en el Perú. *Rev Peru Med Exp Salud Publica*. 2016;33(3):432-7.

27. Lillian Gelberg, Guillermina Natera Rey, Ronald M. Andersen, Miriam Arroyo, Ietza Bojorquez-Chapela, Melvin W. Rico, Mani Vahidi, Julia Yacenda-Murphy, Lisa Arangua & Martin Serota (2017): Prevalence of Substance Use Among Patients of Community Health Centers in East Los Angeles and Tijuana, *Substance Use & Misuse*, 52:3, 359-372

28. Soto-Brandt, G. et al, (2013). Evidencia de validez en Chile del Alcohol, Smoking and Substance Involvement Screening Test (ASSIST). *Adicciones* vol.26 (4).

29. Costa PH, Mota DC, Cruvinel E, Paiva FS, Ronzani TM. (2013). A methodology to implement preventive actions against harmful drug use in the context of primary health care in Latin America *Rev Panam Salud Publica*. 33(5):325-31.

30. Natera R et al., 2014: Final Narrative Report of the bi-national assist screening and quit using drugs intervention trial (quit) Tijuana/Los Angeles. Mexico, National Institute on Psychiatry de la Fuente Muniz.

31. Kaner E. Brief alcohol interventions – time for translational research. Addiction 2010; 105: 960-961.

32. Eccles M, Grimshaw J, Walker A, et al.: Changing the behavior of healthcare professionals: the use of theory in promoting the uptake of research findings. *J Clin Epidemiol.* 2005;58(2):107–112. 15680740 10.1016/j.jclinepi.2004.09.002

33. Norton W, Mittman B: Scaling-up Health Promotion/Disease Prevention Programmes in Community Settings: Barriers, Facilitators, and Initial Recommendations. West Hartford, Connecticut, The Patrick and Catherine Weldon Donaghue Medical Research Foundation.2010. Reference Source

34. Woolf SH, Johnson RE: The break-even point: when medical advances are less important than improving the fidelity with which they are delivered. *Ann Fam Med.* 2005;3(6):545–552. 16338919 10.1370/afm.406 1466946

35. Massoud R: An approach to rapid scale-up using HIV/AIDS treatment and care as an example.

For peer review only - http://bmjopen.bmj.com/site/about/guidelines.xhtml

Geneva: World Health Organization;2004. Reference Source

36. Cooley L, Kohl R: Scaling up—from vision to large-scale change: a management framework for practitioners. Washington D.C: Management Systems International;2006. Reference Source

37. Damschroder LJ, Aron DC, Keith RE, et al.: Fostering implementation of health services research findings into practice: a consolidated framework for advancing implementation science. *Implement Sci.* 2009;4:50. 19664226 10.1186/1748-5908-4-50 2736161

38. Yamey G: Scaling up global health interventions: a proposed framework for success. *PLoS Med.* 2011;8(6):e1001049. 21738450 10.1371/journal.pmed.1001049 3125181

39. Adamou B: Guide for monitoring scale-up of health practices and interventions: MEASURE evaluation.2013; Accessed 4 November 2016. Reference Source

40. Barker PM, Reid A, Schall MW: A framework for scaling up health interventions: lessons from large-scale improvement initiatives in Africa. *Implement Sci.* 2016;11(1):12. 26821910 10.1186/s13012-016-0374-x 4731989

41. Heather N. editor. WHO Collaborative Project on Identification and Management of Alcohol-related Problems in Primary Health Care – Report to the World Health Organisation on Phase IV: Development of Country-wide Strategies for Implementing Early Identification and Brief Intervention in Primary Health Care. Geneva: World Health Organisation, Department of Mental Health and Substance Abuse: 2006. http://www.who.int/substance_abuse/publications/identification_management_alcoholproblems_phaseiv.pdf

42. Keurhorst M, Heinen M, Colom J et al. Strategies in primary healthcare to implement early identification of risky alcohol consumption: why do they work or not? A qualitative evaluation of the ODHIN study. Keurhorst et al. BMC Family Practice (2016) 17:70 DOI 10.1186/s12875-016-0461-8.

43. Babor T, Del Boca F, Bray JW. Screening, brief intervention and referral to treatment: implications of SAMHSA's SBIRT initiative for substance abuse policy and practice. Addiction 2017 112 (Supple. 2) 110-117.

44. Vendetti J., Gmyrek A., Damon D., Singh M., McRee B., Del Boca F. Screening, Brief Intervention and Referral to Treatment (SBIRT): implementation barriers, facilitators and model migration. Addiction 2017; 112 (Suppl. 2), 23–33.

45. Singh M., Gmyrek A., Hernandez A., Damon D., Hayashi S. Sustaining Screening, Brief Intervention and Referral to Treatment (SBIRT) services in health-care settings. Addiction 2017; 112 (Suppl. 2), 92–100.

46. Jonas DE, Garbutt JC, Brown JM, Amick HR, Brownley KA, Council CL, et al. Screening, Behavioral Counseling, and Referral in Primary Care to Reduce Alcohol Misuse. Comparative Effectiveness Review No. 64. Rockville, MD: Agency for Healthcare Research and Quality; July 2012. Accessed at www.ncbi .nlm.nih.gov/books/NBK99199/ on 16 April 2016.

47. Rehm J, Anderson P, Manthey J, Shield KD, Struzzo P, Wojnar M, Gual A. (2016). Alcohol use disorders in primary health are: what do we know and where do we go? Alcohol and Alcoholism 51 422-427.

For peer review only - http://bmjopen.bmj.com/site/about/guidelines.xhtml

48. Rubinsky, A.D., Dawson, D.A., Williams, E.C., Kivlahan, D.R. & Bradley, K.A. AUDIT-C Scores as a Scaled Marker of Mean Daily Drinking, Alcohol use disorders Severity, and Probability of Alcohol Dependence in a U.S. General Population Sample of Drinkers. Alcoholism: Clinical and Experimental Research 2013 37 1380-1390.

49. Rehm J., Shield K. D., Rehm M. X., Gmel G., Frick U. Alcohol Consumption, Alcohol Dependence, and Attributable Burden of Disease in Europe: Potential Gains From Effective Interventions for Alcohol Dependence. Toronto, Canada: Centre for Addiction and Mental Health; 2012.

50. National Clinical Guideline Centre (2011). The clinical management of primary hypertension in adults. 2011 https://www.nice.org.uk/guidance/cg127/evidence/cg127-hypertension-full-guideline3 (accessed 1 December 2016).

51. Rehm, J., Anderson, P., Arbesu Prieto, J.A., Armstrong, I., Aubin, H.-J., Bachmann, M., Bastida Bastús N, Brotons, C., Burton, R., Cardoso, M., Colom, J., Duprez, D., Gmel, G., Gual, A., Kraus, L., Kreutz, R., Liira, H., Manthey, J., Møller, L., Okruhlica, L., Roerecke, M., Scafato, E., Schulte, B., Segura-Garcia, L., Shield, K.D., Sierra, C., Vyshinskiy, K., Wojnar, M., & Zarco, J. (2017). Towards new recommendations to reduce the burden of alcohol-induced hypertension in the European Union. BMC Medicine, 15, 173. doi: 10.1186/s12916-017-0934-1.

52. Kaner EF, Beyer FR, Dickinson HO, *et al.*: Effectiveness of brief alcohol interventions in primary care populations. *Cochrane Database Syst Rev.* 2007; (2): CD004148.

53 Bray, J. W., Zarkin, G. A., Hinde, J. M., & Mills, M. J. (2012). Costs of alcohol screening and brief intervention in medical settings: a review of the literature. *Journal of studies on alcohol and drugs*, *73*(6), 911-919. doi: 10.15288/jsad.2012.73.911

54. Groves, P., Pick, S., Davis, P., Cloudesley, R., Cooke, R., Forsythe, M., & Pilling, S. (2010). Routine alcohol screening and brief interventions in general hospital in-patient wards: acceptability and barriers. *Drugs: education, prevention and policy*, *17*(1), 55-71.doi: 10.3109/09687630802088208

55. Platt L, Melendez-Torres GJ, O'Donnell A, et al.: How effective are brief interventions in reducing alcohol consumption: do the setting, practitioner group and content matter? Findings from a systematic review and metaregression analysis. *BMJ Open.* 2016;6(8):e011473. 27515753 10.1136/bmjopen-2016-011473 4985973

56. Kaner, E., Bland, M., Cassidy, P., Coulton, S., Dale, V., Deluca, P., ... & Newbury-Birch, D. (2013). Effectiveness of screening and brief alcohol intervention in primary care (SIPS trial): pragmatic cluster randomised controlled trial. *Bmj*, *346*, e8501. doi: 10.1136/bmj.e8501

57. Wilson GB, Wray C, McGovern R, et al.: Intervention to reduce excessive alcohol consumption and improve comorbidity outcomes in hypertensive or depressed primary care patients: two parallel cluster randomized feasibility trials. *Trials.* 2014;15:235. 24947447 10.1186/1745-6215-15-235 4076249

58. Linde K, Sigterman K, Kriston L, et al.: Effectiveness of psychological treatments for depressive disorders in primary care: systematic review and meta-analysis. *Ann Fam Med.* 2015;13(1):56–68. 25583894 10.1370/afm.1719 4291267

For peer review only - http://bmjopen.bmj.com/site/about/guidelines.xhtml

59. Gilbody S, Whitty P, Grimshaw J, et al.: Educational and organizational interventions to improve the management of depression in primary care: a systematic review. *JAMA.* 2003;289(23):3145–3151. 12813120 10.1001/jama.289.23.3145

60. Anderson P, O'Donnell A, Kaner E *et al.* Scaling-up primary health care-based prevention and management of alcohol use disorder at the municipal level in middle-income countries in Latin America: Background and pre-protocol for a three-country quasi-experimental study. *F1000Research* 2017, 6:311 (doi: 10.12688/f1000research.11173.1).

61. Flottorp SA, Oxman AD, Krause J, et al.: A checklist for identifying determinants of practice: a systematic review and synthesis of frameworks and taxonomies of factors that prevent or enable improvements in healthcare professional practice. *Implement Sci.* 2013;8:35. 23522377 10.1186/1748-5908-8-35 3617095

62. Wensing M, Oxman A, Baker R, et al.: Tailored Implementation For Chronic Diseases (TICD): a project protocol. *Implement Sci.* 2011;6:103. 21899753 10.1186/1748-5908-6-103 3179734

63. Wensing M, Huntink E, van Lieshout J, et al.: Tailored Implementation of Evidence-Based Practice for Patients with Chronic Diseases. *PLoS One.* 2014;9(7):e101981. 25003371 10.1371/journal.pone.0101981 4087017

64. SAMHSA. AUDIT-C. https://www.integration.samhsa.gov/images/res/tool_auditc.pdf

65. Babor TF, Higgins-Biddle JC, Saunders JB, Monteiro MG AUDIT: The Alcohol Use Disorders Identification Test Guidelines for Use in Primary Care (second edition). https://www.who.int/substance_abuse/publications/audit/en/

66. Center for Quality Assessment and Improvement in Mental Health Patient Health Questionnaire PHQ-2. http://www.cqaimh.org/pdf/tool_phq2.pdf

67. US Preventive Service task Force. Patient Health Questionnaire PHQ-9. https://www.uspreventiveservicestaskforce.org/Home/GetFileByID/218

68. Anderson P, Clement S: The AAPPQ revisited: the measurement of general practitioners' attitudes to alcohol problems. *Br J Addict.* 1987;82(7):753–9. 3478065 10.1111/j.1360-0443.1987.tb01542.x

69. Schaufeli WB, Shimazu A, Hakanen J, Salanova M, De Witte H. An Ultra-Short Measure for Work Engagement: The UWES-3 Validation Across Five Countries. European Journal of Psychological Assessment 2017

70.Rosenberg G, Bauld L, Hooper L et al. New national alcohol guidelines in the UK: public awareness, understanding and behavioural intentions. *Journal of Public Health*, 2018 40, 549–556,

71. Chung A, Rimal RN: Social norms: A review. *Review of Communication Research.* 2016;4:1–29. 10.12840/issn.2255-4165.2016.04.01.008

72. Sørensen K, Van den Broucke S, Pelikan JM, et al.: Measuring health literacy in populations: illuminating the design and development process of the European Health Literacy Survey Questionnaire (HLS-EU-Q). *BMC Public Health.* 2013;13:948. 24112855 10.1186/1471-2458-13-948 4016258

For peer review only - http://bmjopen.bmj.com/site/about/guidelines.xhtml

73. Kaner EF, Dickinson HO, Beyer FR, Campbell F, Schlesinger C, Heather N, Saunders JB, Burnand B, Pienaar ED. Effectiveness of brief alcohol interventions in primary care populations. *Cochrane Database of Systematic Reviews* 2007, Issue 2. Art. No.: CD004148. DOI: 10.1002/14651858.CD004148.pub3.

74. Svoronos T, Mate KS: Evaluating large-scale health programmes at a district level in resource-limited countries. *Bull World Health Organ.* 2011;89(11):831–837. 22084529 10.2471/BLT.11.088138 3209726

75. Derges, J., Kidger, J., Fox, F., Campbell, R., Kaner, E., & Hickman, M. (2017). Alcohol screening and brief interventions for adults and young people in health and community-based settings: A qualitative systematic literature review. *BMC Public Health*, *17*(1), 1–12. https://doi.org/10.1186/s12889-017-4476-4

76. Johnson, M., Jackson, R., Guillaume, L., Meier, P., & Goyder, E. (2011). Barriers and facilitators to implementing screening and brief intervention for alcohol misuse: A systematic review of qualitative evidence. *Journal of Public Health*, *33*(3), 412–421. https://doi.org/10.1093/pubmed/fdq095

77. O'Donnell, A., Wallace, P., & Kaner, E. (2014). From efficacy to effectiveness and beyond: What next for brief interventions in primary care? *Frontiers in Psychiatry*, *5*(AUG), 1–8. https://doi.org/10.3389/fpsyt.2014.00113

78. Moore GF, Audrey S, Barker M, et al.: Process evaluation of complex interventions: Medical Research Council guidance. *BMJ.* 2015;350:h1258. 25791983 10.1136/bmj.h1258 4366184

79. Glasgow RE, Vogt TM, Boles SM: Evaluating the public health impact of health promotion interventions: the RE-AIM framework. *Am J Public Health.* 1999;89(9):1322–7. 10474547 10.2105/AJPH.89.9.1322 1508772

80. Glasgow RE, Klesges LM, Dzewaltowski DA, et al.: Evaluating the impact of health promotion programs: using the RE-AIM framework to form summary measures for decision making involving complex issues. *Health Educ Res.* 2006;21(5):688–694. 16945984 10.1093/her/cyl081

81. Harden SM, Gaglio B, Shoup JA, et al.: Fidelity to and comparative results across behavioral interventions evaluated through the RE-AIM framework: a systematic review. *Syst Rev.* 2015;4:155. 26547687 10.1186/s13643-015-0141-0 4637141

82. Ysa T, Colom J, Albareda A, et al.: Governance of Addictions. European Public Policies. Oxford University Press,2014. Reference Source

83. OECD: Compendium of OECD well-being indicators. OECD Better Life Initiative.2011; (Accessed 18 December 2016). Reference Source

84. Bertelsmann Stiftung. [website].2016; (Accessed 18 December 2016). Reference Source

85. Inglehart R, Welzel C: Modernization, Cultural Change and Democracy. New York: Cambridge University Press,2005. Reference Source

86. PASS[16] sample size software. https://www.ncss.com/software/pass/: Donner, A. and Klar, N. 2000. Design and Analysis of Cluster Randomization Trials in Health Research. Arnold. London.

For peer review only - http://bmjopen.bmj.com/site/about/guidelines.xhtml

87. Hutchings, D., et al., *Implementing screening and brief alcohol interventions in primary care: views from both sides of the consultation.* Primary Health Care Research and Development, 2006. **7**: p. 221-229.

88. Tam, C.W.M., et al., *Alcohol enquiry by GPs - Understanding patients' perspectives: A qualitative study.* Australian family physician, 2015. **44**(11): p. 833-838.

89. Lock, C.A., *Alcohol and brief intervention in primary health care: what do patients think?* Primary Health Care Research and Development, 2006. **5**(2): p. 162-178.

90. Khadjesari, Z., et al., *'I'm not a real boozer': a qualitative study of primary care patients' views on drinking and its consequences.* Journal of public health (Oxford, England), 2018: p. fdy067-fdy067.

91. O'Donnell, A., et al., *Beliefs and attitudes about addressing alcohol consumption in health care: a population survey in England.* BMC Public Health, 2018. **18**(1): p. 391.

92. Miller, P.M., S.E. Thomas, and R. Mallin, *PATIENT ATTITUDES TOWARDS SELF-REPORT AND BIOMARKER ALCOHOL SCREENING BY PRIMARY CARE PHYSICIANS.* Alcohol and Alcoholism, 2006. **41**(3): p. 306-310.

93. Nilsen, P., et al., *Brief interventions in routine health care: a population-based study of conversations about alcohol in Sweden.* Addiction, 2011. **106**: p. 1748 - 1756.

94. Aalto, M., P. Pekuri, and K. Seppä, *Primary health care professionals' activity in intervening in patients' alcohol drinking: a patient perspective.* Drug and Alcohol Dependence, 2002. **66**(1): p. 39-43.

95. O'Donnell, A., et al., *Patients' experiences of alcohol screening and advice in primary care: A qualitative study.* BMC Family Practice, in press. **DOI: 10.1186/s12875-020-01142-9**.

96. Dietz WH, Solomon LS, Pronk N, et al.: An Integrated Framework For The Prevention And Treatment Of Obesity And Its Related Chronic Diseases. *Health Aff (Millwood).* 2015;34(9):1456–1463. 26355046 10.1377/hlthaff.2015.0371

97. Palumbo R: Contextualizing co-production of health care: a systematic literature review. *Int J Public Se Manage.* 2016;29(1):72–90. 10.1108/IJPSM-07-2015-0125

98. Rehm J, Room R, Monteiro M, et al.: Alcohol Use. In: *Comparative quantification of health risks: global and regional burden of disease attributable to selected major risk factors*. Ezzati M *et al.* (eds), Geneva, Switzerland: World Health Organization.2004;959–1109. Reference Source

99. European Medicines Agency: Guideline on the development of medicinal products for the treatment of alcohol dependence.2010. Reference Source

100. Rehm J, Lachenmeier DW, Room R: Why does society accept a higher risk for alcohol than for other voluntary or involuntary risks? *BMC Med.* 2014;12:189. 25424648 10.1186/s12916-014-0189-z 4203927

101. Cunningham JA: Unintended impact of using different inclusion cut-offs for males and females in intervention trials for hazardous drinking. *Addiction.* 2017. 28168847 10.1111/add.13760

102. Aldridge A, Dowd W, Bray J: The relative impact of brief treatment versus brief intervention in

For peer review only - http://bmjopen.bmj.com/site/about/guidelines.xhtml

primary health-care screening programs for substance use disorders. *Addiction.* 2017;112(Suppl 2):54–64. 28074568 10.1111/add.13653

103. Barbosa C, Cowell A, Dowd W, et al.: The cost-effectiveness of brief intervention versus brief treatment of screening, brief intervention, and referral to treatment (SBIRT) in the United States. *Addiction.* 2017;112(Suppl 2):73–81. 28074567 10.1111/add.13658

104. Anderson P, Kaner E, Keurhorst M, et al.: Attitudes and Learning through Practice Are Key to Delivering Brief Interventions for Heavy Drinking in Primary Health Care: Analyses from the ODHIN Five Country Cluster Randomized Factorial Trial. *Int J Environ Res Public Health.* 2017;14(2): pii: E121. 28134783 10.3390/ijerph14020121 5334675

105. Funk M, Wutzke S, Kaner E, et al.: A multicountry comparator led trial of strategies to promote dissemination and implementation of brief alcohol intervention in primary health care: findings of a World Health Organization collaborative study. *J Stud Alcohol.* 2005;66(3):379–388. 16047527 10.15288/jsa.2005.66.379

106. Reeves BC, Wells GA, Waddington H: Quasi-experimental study designs series-paper 5: classifying studies evaluating effects of health interventions-a taxonomy without labels. *J Clin Epidemiol.* 2017; pii: S0895-4356(17)30288-3. 28351692 10.1016/j.jclinepi.2017.02.016

107. Jantsch E: Inter- and Transdisciplinary University: A systems approach to education and innovation. *Policy Sciences.* 1970;1(1):403–428. 10.1007/BF00145222

108. Pohl C, Hirsch Hadorn G: Principles for Designing Transdisciplinary Research. proposed by the Swiss Academies of Arts and Sciences. München: oekom Verlag.2007. Reference Source

For peer review only

For peer review only - http://bmjopen.bmj.com/site/about/guidelines.xhtml

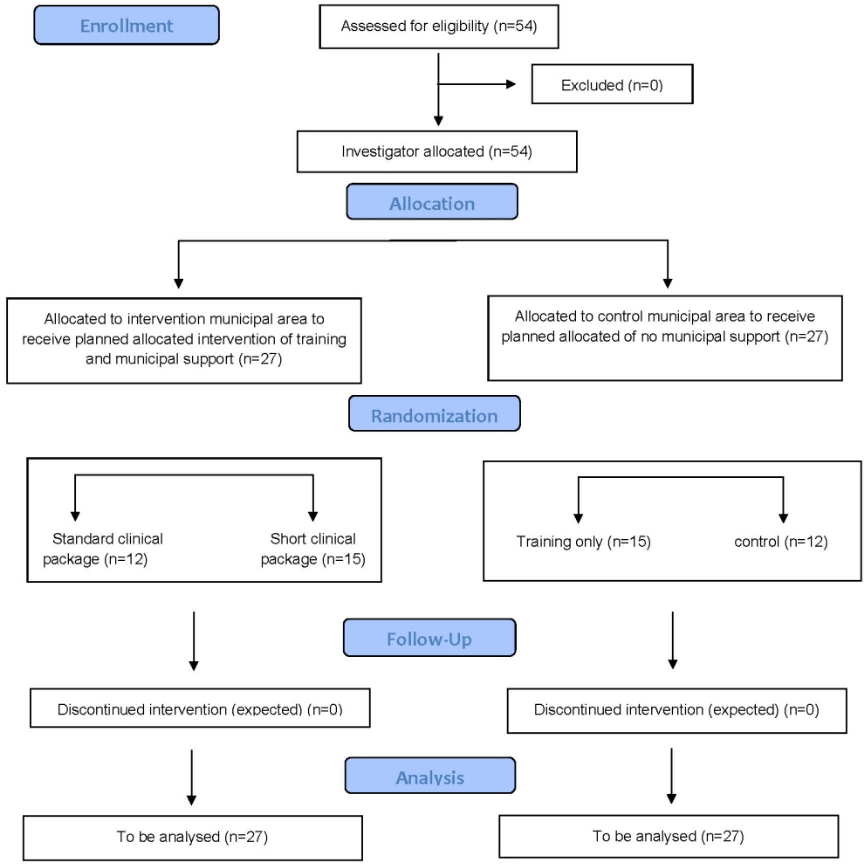

Figure 1 Study flow diagram

215x279mm (200 x 200 DPI)

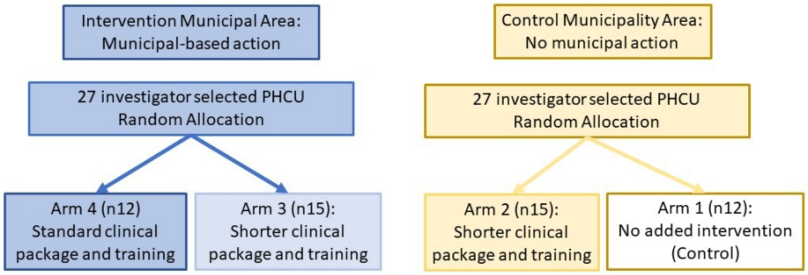

Figure 2. Study design for the first six months of the 18-month implementation period

338x190mm (96 x 96 DPI)

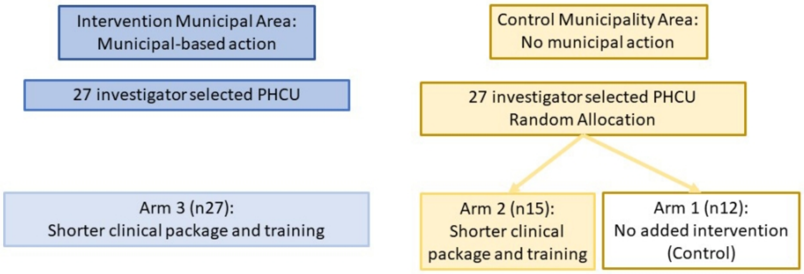

Figure 3. Study design from month 8 onwards, assuming no superiority of Arm 4 over Arm 3 during first six months of implementation.

338x190mm (96 x 96 DPI)

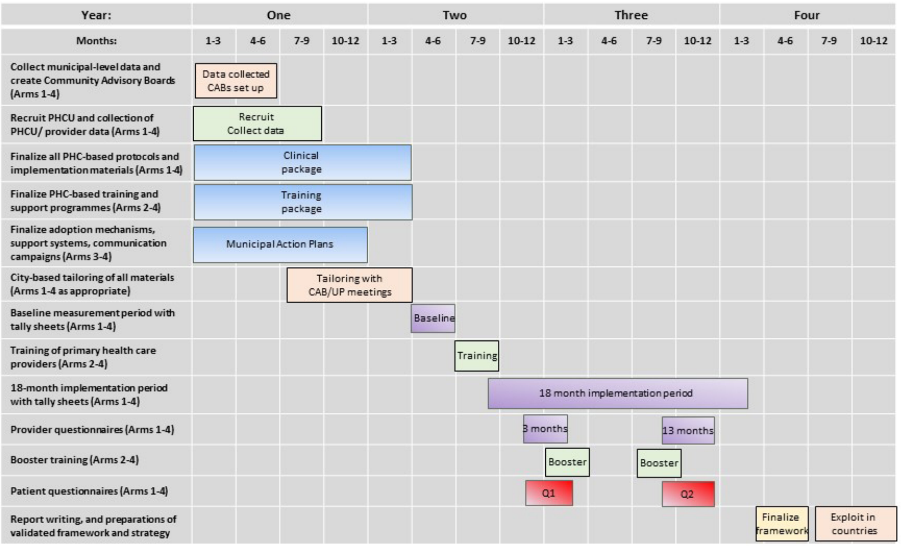

Figure 4. Study timetable.

254x190mm (96 x 96 DPI)

Supplement Box 1 Deviations from pre-grant submission pre-protocol

Moving from two-arm to four-arm design In the pre-submission pre-protocol for the quasi-experimental study [1], within each country, two municipal jurisdictions were to be investigator-selected, each with nine primary health care units (PHCU) as part of the study. In one municipal jurisdiction, the intervention municipality, the PHCU would receive both training and municipal support; in the other municipal jurisdiction, the comparator municipality, PHCU would continue practice as usual, with no training or municipal support. The hypothesis was that PHCU in the intervention municipality would measure the alcohol consumption of more patients and give advice to more heavy drinking patients than the PHCU in the comparator municipality.

In the final protocol, within each country, the nine PHCU in the comparator municipality are randomly allocated to five PHCU receiving training (new Arm 2) and four PHCU continuing practice as usual (new Arm 1). The rationale for this approach is that it will enable us to test the independent impact of municipal support over and above just training. The hypothesis to be tested is that PHCU that receive both training and municipal support in the intervention municipality will measure the alcohol consumption of more patients and give advice to more heavy drinking patients than the PHCU who just receive training (Arm 2).

In addition, in the final protocol, within each country, the nine PHCU in the intervention municipality are randomly allocated to four PHCU receiving a standard and longer clinical package and training (new Arm 4) and five PHCU receiving a shorter clinical package and training (new Arm 3), both new Arms 3 and 4 receiving municipal support. The hypothesis to be tested is that the PHCU that receive the standard and longer clinical package and training that is commonly implemented (new Arm 4) will not measure the alcohol consumption of more patients and not give advice to more heavy drinking patients than the PHCU that receive a shorter clinical package and training (new Arm 3). This will be tested over the first six months of the 18-month implementation period, and, if there is non-superiority of Arm 4 over Arm 3, Arm 4 will be collapsed into Arm 3 from month 8 onwards.

Cross-sectional patient self-complete questionnaire instead of prospective interview The deviation is to move from patient follow-up interviews to cross-sectional patient self-completed questionnaires. In the pre-submission pre-protocol, during month 3 of the 18-month implementation period, the first six consecutive screen-negative patients and the first six consecutive screen-positive patients identified by each PHCU were to be invited by the health care provider to give their written consent to complete two follow-up questionnaires, at six months and twelve months after the initial screening. In the final protocol, at two time points, during the 18-month implementation period (months 3 and 15), on two separate days in each of month 3 and 15, providers will seek consent from the patient to self-complete additional questions in the waiting room before leaving the PHCU, handing the completed questions to a researcher in attendance. The rationale for the change is that, primarily due to the nature of the catchments area of patients, it became apparent that it would be impossible to achieve sufficient follow-up rates required for valid analysis of data, with much too high a proportion of country-based resources used in order to try to achieve adequate follow-up rates.

Adjustment in primary outcome indicator The deviation is to change the denominator for the main outcome variable from number of consulting adult patients in a given time period (e.g., one month) to number of registered adult patients. In the pre-submission pre-protocol, the primary outcome was to be the proportion of consulting adult patients (aged 18+ years) intervened (alcohol consumption measured and advice given to heavy drinkers), calculated as the number of AUDIT-C positive patients that received oral advice or referral for advice to another provider in or outside the PHCU, divided by the total number of adult consultations of the participating providers per PHCU. In the final protocol, the primary outcome will be the cumulative proportion of the number of adults (aged 18+ years) registered with the PHCU that have their alcohol consumption measured with AUDIT-C. The rationale is that the revised primary outcome is a measure of coverage, which is considered more intuitive and relevant for health systems change (similar to blood pressure - the proportion of patients that have had their blood pressure measured).

Recalculation of statistical power The change in the main outcome measure required a re-calculation of the statistical power. The study remains adequately powered.

For peer review only - http://bmjopen.bmj.com/site/about/guidelines.xhtml

Supplement Table 1 Clinical Package and Training by Study Arm

	Standard package and training (Arm 4)	Shorter package and training (Arms 2 and 3)	Control (Arm 1)
**Instruments**	Short tally sheet: AUDIT-C [2] completed; if AUDIT-C ≥8, AUDIT-10 [3] and PHQ2 [4] completed; if PHQ2 ≥3, PHQ9 [5] completed.	Very short tally sheet: AUDIT-C completed; if AUDIT-C ≥8, PHQ2 completed.	Very short tally sheet: AUDIT-C completed; if AUDIT-C ≥8, PHQ2 completed.
**Provider material**	Provider booklet on alcohol and depression: 43 pages plus 12-page 'quick guide'.	Provider booklet on alcohol and depression: 16 pages.	Provider booklet on alcohol and depression: 11 pages.
**Patient advice and material for alcohol**	Alcohol advice: 5-minute 10-step plan plus 10-page patient brief advice booklet.	Alcohol advice: 1-minute simple advice that the patient needs to drink less, plus 1-page patient brief advice leaflet.	Alcohol advice: 1-minute simple advice that the patient needs to drink less and provide a brief advice leaflet (if available).
	Patient alcohol leaflet: 1 page folded in half to give 4 sides.	Patient alcohol leaflet: 1 page folded in half to give 4 sides.	SCALA patient leaflet on alcohol not given. Provider booklet advises "If available, provide a leaflet on self-management of heavy drinking."
**Patient advice and material for depression**	PHQ9 score 10-14, provide patient leaflet on depression; PHQ 9 ≥14, use clinical judgement to consider if referral is required - if not provide patient leaflet on depression.	PHQ2 ≥3, patient leaflet on depression given.	SCALA patient leaflet on depression not given. Provider booklet advises "If available, provide a leaflet on self-management of depression and action to take if symptoms persist or worsen."
	Patient depression advice leaflet: 1 page, 3 columns.	Patient depression advice leaflet: 1 page, 3 columns.	Present practice.
**Referral**	Referral for very heavy drinking, depression, suicide risk: existing clinical judgement and practice.	Referral for very heavy drinking, depression, suicide risk: existing clinical judgement and practice.	Referral for very heavy drinking, depression, suicide risk: existing clinical judgement and practice.
**Training**	Training: two times two-hours training plus two times one-hour booster sessions (six hours total).<br>Training will take place within	Training: one two-hours training in PHCU, plus one-hour booster session (three hours total).<br>Training will focus on	Present practice.

For peer review only

	the PHCU or clusters of PHCUs. Training will focus on practical skills in undertaking measurement and assessment, and in delivering brief advice, in using the questionnaires, and in knowing when and how to refer patients with more severe heavy drinking and moderately severe or severe depression to available services, such as community-based mental health and addiction centres. Training will, in addition, address attitudes, and perceived barriers and facilitators in implementing measurement and brief advice, contextualized to local circumstances.	practical skills in undertaking measurement and assessment, and in delivering brief advice for harmful alcohol use; instruction of 'care-as-usual' + leaflet for depression and severe cases requiring referral. Training will, in addition, address attitudes, and perceived barriers and facilitators in implementing measurement and brief advice, contextualized to local circumstances.	
	Training for both the standard and shorter packages will be undertaken by members of the research team, accredited teachers, or addiction consultants, who will receive a full two-day train-the-trainers session from a senior addiction specialist trainer. The training formats employed are didactic input, guided discussions, skills and practice modeled through videos and role plays. Training sessions are developed from [6-7].		

For peer review only - http://bmjopen.bmj.com/site/about/guidelines.xhtml

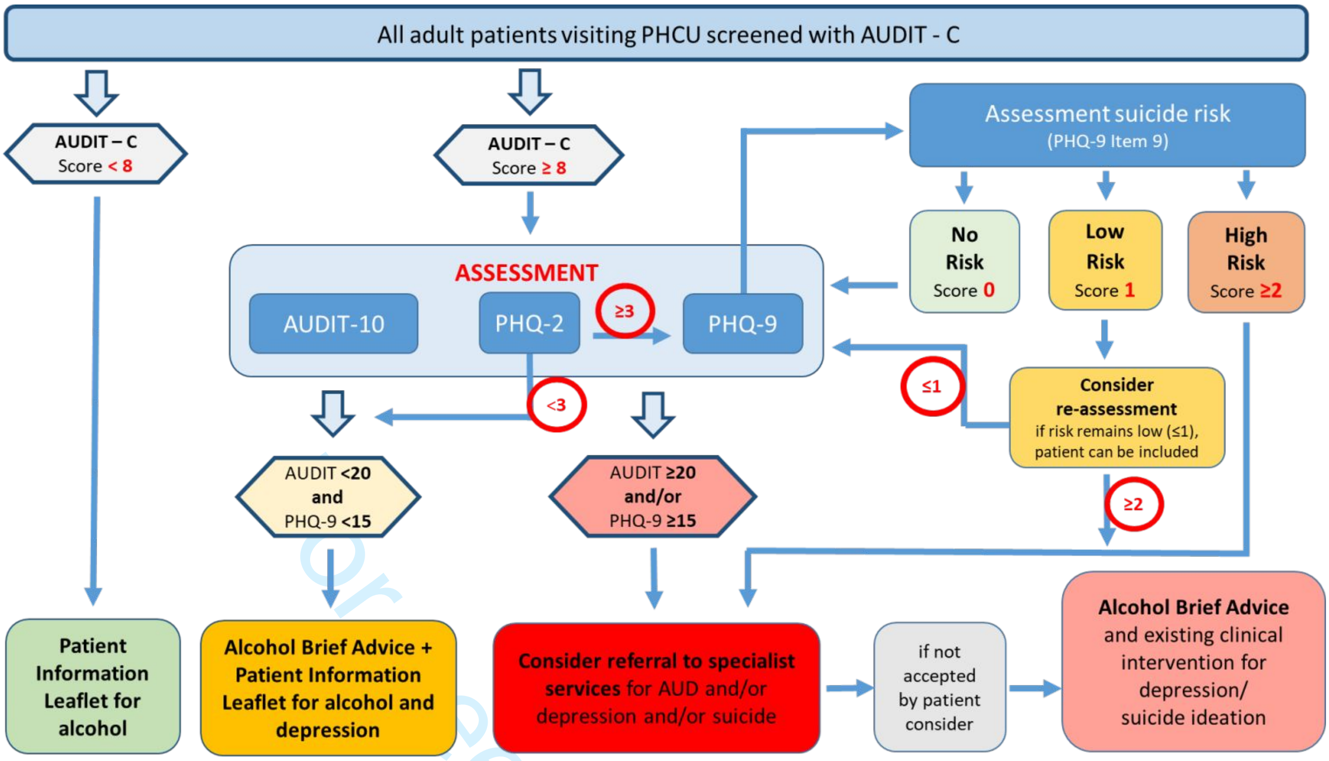

Supplement Figure 1. Standard Care Pathway for Arm 4

For peer review only - http://bmjopen.bmj.com/site/about/guidelines.xhtml

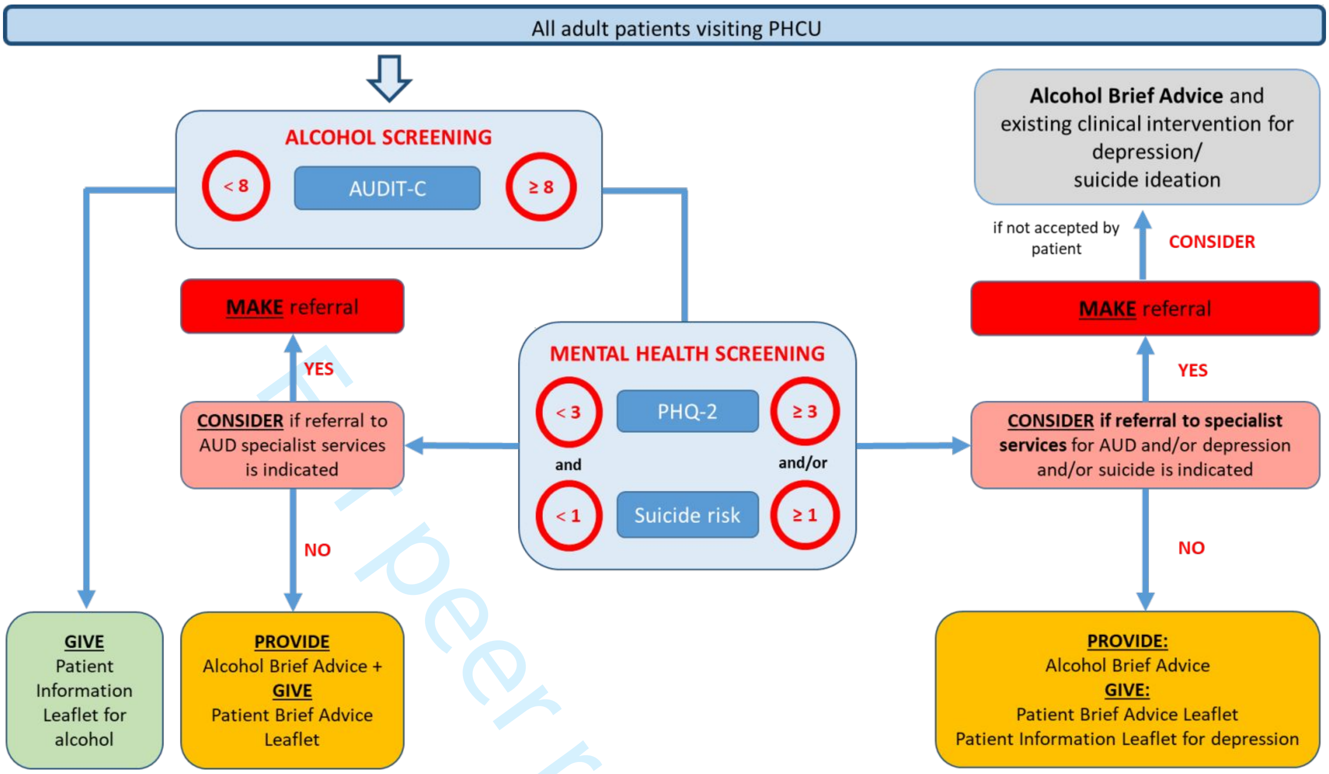

Supplement Figure 2. Short Care Pathway for Arms 1, 2, and 3

For peer review only - http://bmjopen.bmj.com/site/about/guidelines.xhtml

Supplement Table 2 Municipal Integration and Support by Study Arm

**Intervention Municipal Area (Arms 3 and 4)**	**Comparator Municipal Area (Arms 1 and 2)**
Community Advisory Board (CAB) of local stakeholders set up (including representatives of municipal area, PHCU, health services, non-governmental organizations, academia, media).	Present practice.
User Panel (UP) of local providers and patients set up.	Present practice.
CAB and UP review and tailor relevant materials of clinical package and training courses within the seven domains of: local and national guideline factors; individual health care provider factors; patient factors; interactions between different professional groups; incentives and resources; capacity for organizational change; and, social, political and legal factors [8-10].	Present practice.
CAB reviews barriers and facilitators and potential drivers of successful action [11-12].	Present practice.
CAB identifies potential adoption mechanisms and support systems [13], and reviews plans and components of community-based communication and media campaigns [14-16].	Present practice.
Integrator (champion and knowledge and practice broker) to serve as trusted and accountable leader [13]: facilitating agreement within the municipal area and health systems on shared goals and metrics; assessing and acting on relevant community resources; working at the systems level to make relevant practice changes for sustainability; gathering, analysing, monitoring, integrating, learning, and sharing data at the individual PHCU and city levels; identifying and connecting with system navigators who help PHCUs coordinate, access, and manage multiple services and supports; and developing a system of ongoing and intentional communication with PHCUs and cities.	Present practice.
Adoption mechanisms implemented [13], including: (i) demonstration of the superiority of the PHC package, its simplicity, and its alignment with the latest evidence of preventing and managing heavy drinking and of implementation science; (ii) engagement of identified leaders and building their capacity to lead and ensure broad adoption of the PHC package through guiding and supporting large-scale change; (iii) communicating the value of the PHC package to both municipal and PHC frontline staff; (iv) identifying and adjusting, as appropriate and possible, relevant policies at PHC and city levels to expedite the adoption of the PHC package, for example by adapting electronic health records; and, (v) identifying gaps in health system performance and the urgent need to prevent and manage heavy drinking to promote the needed will and energy to bring implementation of the PHC package to scale.	Present practice.
Support mechanisms implemented [13], including: (i) development of professional capacity for scale-up; (ii) development of infrastructure for scale-up, achieved through redesign rather than addition of new resources; (iii) linking to monitoring and evaluation, using reliable data collection and reporting systems that track and provide feedback on the performance of key processes and outcomes, for example monthly reporting on measurement and brief advice activity; (iv) setting up learning systems to capture change ideas that are shown to result in improved performance assembling ideas into a change package. Knowledge should be shared between municipal actors and PHCUs through regular electronic newsletters and communications; and, (v) creating design factors that enhance sustainability including high reliability of the new processes, inspection systems	Present practice.

For peer review only - http://bmjopen.bmj.com/site/about/guidelines.xhtml

to ensure desired results are being achieved, support for structural elements, and ongoing learning systems.	
Communication and media campaign implemented [14-16], including (i) posters, leaflets and/or brochures placed at visible spots in the intervention municipality, e.g., in waiting rooms of PHCUs, health departments, banks, markets; (ii) regular communications, including emails and WhatsApp messages) sent to the healthcare providers and other involved stakeholders in the intervention municipality, (iii) media presence through e.g. articles in local newspapers; interviews, reportages, promotion spots and/or media appearances on local radio, local TV and other local media, and (iv) workshops, forums and/or public local meetings for interested stakeholders such as healthcare providers, representatives of municipal health institutions and patients. All abovementioned activities will focus on reframing that it is heavy drinking that is the problem and that this can be helped to be reduced through primary health care-based measurement and advice programmes, addressing topics such as the harm of hazardous alcohol use in the general population, the (cost)effectiveness and importance of brief alcohol interventions and SCALA success stories.	Present practice.

For peer review only

Supplement Table 3 Data collected at municipal level (if not available, at city, regional or country level)

-	Geographical location in city;
-	Demographic size of municipal area;
-	Indicators of deprivation;
-	Information on prevalence of alcohol consumption and related harm;
-	Information on prevalence of depression;
-	Description of current action to reduce alcohol-related harm;
-	Jurisdictional responsibilities for health-related prevention and treatment;
-	Structural relationships with primary health care services;
-	Structural relationships with hospital-based services;
-	Available data mapped to OECD better life initiative [17], including material living conditions (housing, income and jobs) and quality of life (community, education, environment, governance, health, life satisfaction, safety and work-life balance);
-	Sustainable Governance Indicators [18], including the Status Index, which 'examines each state's reform needs in terms of the quality of democracy and performance in key policy fields', and the Management Index, focused on 'governance capacities in terms of steering capability and accountability'; and,
-	World Values Survey data [19] for cross-cultural variation (Traditional vs. Secular-rational; and, Survival vs. Self-expression).

For peer review only - http://bmjopen.bmj.com/site/about/guidelines.xhtml

Supplement Table 4 Overview of the measures used in the provider questionnaire

Measure used	Constructs measured
Shortened Alcohol and Alcohol Problems Perception questionnaire [20]	Role security, therapeutic commitment
Abbreviated Maslach Burnout Inventory [21]	Emotional exhaustion, depersonalization, personal accomplishment
Utrecht Work Engagement Scale [22]	Work engagement
Alcohol knowledge [23]	Awareness of drinking guidelines, social norms regarding drinking
Perceived barriers questionnaire [24]	Perceived barriers
Opinion on screening (based on [25])	Pros and cons of screening, social norms of screening, intention to screen
Self-efficacy in delivering the SCALA protocol (based on [26])	Self-efficacy
Context assessment for community health (COACH) tool [27]	Resources, Community engagement, Monitoring services for action, Work culture, Leadership
Evaluation of SCALA community action [15]	Exposure to campaign/adoption mechanisms/support systems, perceptions of campaign/adoption mechanisms/support systems
Attributes of innovation questionnaire [28]<br>- Only intervention group	Relative advantage, Compatibility, Complexity, Trialability and Observability
Experienced barriers (based on the driver diagram [12])<br>- Only intervention group	Experienced barriers

For peer review only - http://bmjopen.bmj.com/site/about/guidelines.xhtml

Supplement Table 5. Country-level collection of economic data for return-of-investment analyses

Costs of Investment		Gains of investment	
Cost unit	***Data source***	***Cost unit***	***Data Source***
Cost of providing training and booster sessions to PHCU staff	Time and materials required, documented by study team	Costs and utilization of *primary health care* (number of visits) by major disease/injury categories	National statistics, ministry of health, local researchers, or other publications
Setting up and maintaining Community Advisory Boards and User Panels	Time and materials required, documented by study team	Costs and utilization of *emergency* facilities (number of admissions) by major disease/injury categories	National statistics, ministry of health, local researchers, or other publications
Direct costs for implementing the clinical pathway (routine measurement, further assessment, brief interventions, referral)	Staff salary and time required, documented by PHCU administration and providers	Costs and utilization of *inpatient* facilities (number of admissions, length of stay) and of *outpatient* facilities (number of admissions) by major disease/injury categories	National statistics, ministry of health, local researchers, or other publications
Additional costs for implementing the clinical pathway	Documented by PHCU administration	Avoided mortality	National statistics, ministry of health, local researchers, or other publications

For peer review only - http://bmjopen.bmj.com/site/about/guidelines.xhtml

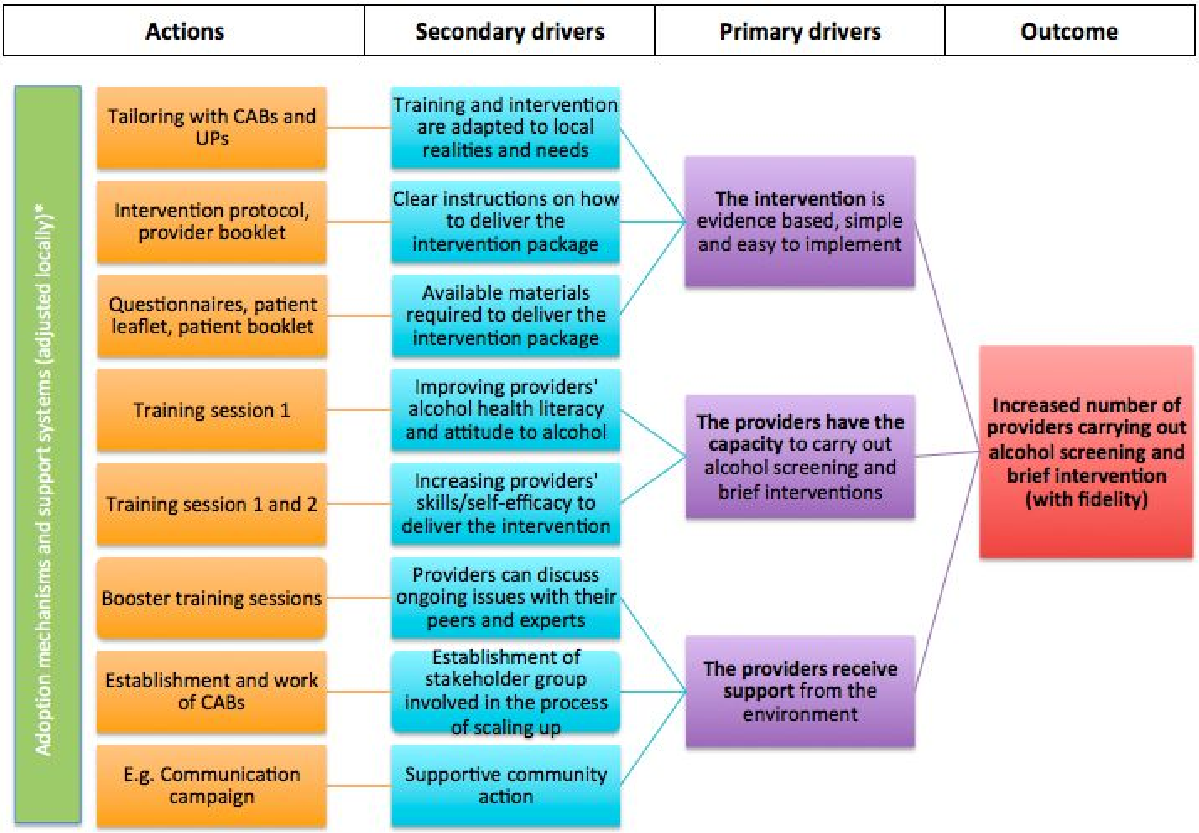

Supplement Figure 3. Driver diagram of the SCALA protocol

review only

For peer review only - http://bmjopen.bmj.com/site/about/guidelines.xhtml

Supplement Table 6 Process evaluation topics based on MRC framework [29]

Part of process evaluation		Topic of investigation	Method
**Description of the intervention**		The description of the intervention and its causal assumptions	Driver diagram
**Implementation**	*Adaptation*	Experience of intervention tailoring	Key informant interview
		Experience with training tailoring	Key informant interview
	Dose delivered (completeness of delivery)	Implementation of the protocol (number of measurements, brief advice given, referrals done)	Tally sheets
		Length of implemented training	Observation
		Implementation of adoption mechanisms and support systems on municipal and organisational level	Key informant interview, Document analysis
		Implementation of CAB meetings	Observation, document analysis
		Implementation of communication campaign	Key informant interview, document analysis
	Fidelity (quality of implementation)	Following the care pathway as intended	Tally sheets, patient questionnaire
		Training active ingredient delivery	Observation
	Reach	Number of patients and providers involved	Document analysis
		Number of providers attending the training	Document analysis
**Mechanisms of impact**	*Participant responses*	Patients' perception of acceptability of intervention	Patient questionnaire
		Providers' satisfaction with the training	Post-training questionnaire
		Providers' perceived utility of training sessions	Post-training questionnaire
		Perception of the intervention	Key informant interview
		Perception of the campaign	Provider questionnaire, patient questionnaire
		Perception of the municipal action	Key stakeholder interview
	Mediators	Influence of training on attitude and self-efficacy	Provider questionnaire
		Influence of communication campaign on beliefs and social norms	Provider questionnaire
		Perception of the attributes of the intervention	Provider questionnaire
	Unintended consequences	Possible unexpected side effects emerging	Key stakeholder interview
**Context**		Perceptions of organisational context	Provider questionnaire
		Individual moderating characteristics	Provider questionnaire
		Description of organisational context changes	Key informant interview, logbook
		Contextual factors influencing training	Observation, key informant interview
		Contextual factors influencing municipal action	Key informant interview, document analysis
**Outcomes**		Integration of process evaluation information with the results of the outcome evaluation	Integration of data collected through abovementioned methods with the tally sheet data

For peer review only

For peer review only - http://bmjopen.bmj.com/site/about/guidelines.xhtml

Supplement Table 7 Completed seven-point checklist for SCALA study design [30]

Quality Measure	SCALA
1.Was the intervention/(answer "yes" to more than 1 item, if applicable)	
Allocated to (provided for / administered to / chosen by) individuals?	No
Allocated to (provided for / administered to / chosen by) clusters of individuals?	No
Clustered in the way it was provided (by practitioner or organisational unit)?	YES
**2. Were outcome data available: *(answer "yes" to only 1 item)***	
After intervention / comparator only (same individuals)?	-
After intervention / comparator only (not all same individuals)?	-
Before (once) AND after intervention / comparator (same individuals)?	YES
Before (once) AND after intervention / comparator (not all same individuals)?	-
Multiple times before AND multiple times after intervention / comparator(same individuals)?	-
Multiple times before AND multiple times after intervention / comparator (not all same individuals)?	-
**3. Was the intervention effect estimated by: *(answer "yes" to only 1 item)***	
CHANGE OVER TIME (same individuals at different time points)?	-
CHANGE OVER TIME (not all same individuals at different time points)?	-
DIFFERENCE BETWEEN GROUPS (of individuals or clusters receiving either intervention or comparator)?	YES
**4. Did the researchers aim to control for confounding (design or analysis) *(answer "yes" to only 1 item)*:**	
Using methods that control in principle for any confounding?	-
Using methods that control in principle for time invariant unobserved confounding?	-
Using methods that control only for confounding by observed covariates?	YES
**5. Were groups of individuals or clusters formed by *(answer "yes" to more than 1 item, if applicable)*:**	
· Randomization?	No
· Quasi-randomization?<br>· Explicit rule for allocation based on a threshold for a variable measured on a continuous or ordinal scale or boundary (in conjunction with identifying the variable dimension, below)?	No
· Some other action of researchers?	YES
· Time differences?	No
· Location differences?	YES
· Healthcare decision makers / practitioners?	No
· Participants' preferences?	No
· Policy maker	No
· On the basis of outcome?	No
· Some other process? (specify)	No
**6. Were the following features of the study carried out after the study was designed *(answer "yes" item, if applicable): to more than 1***	
Characterization of individuals / clusters before intervention?	YES
Actions/choices leading to an individual/cluster becoming a member of a group?	YES
Assessment of outcomes?	YES

For peer review only - http://bmjopen.bmj.com/site/about/guidelines.xhtml

**7. Were the following variables measured before intervention:** ***(answer "yes" to more than 1 item,*** **If applicable)**	
Potential confounders?	YES
Outcome variable(s)?	YES

For peer review only

For peer review only - http://bmjopen.bmj.com/site/about/guidelines.xhtml

References

1. Shadish WR, Cook TD, Campbell DT: Experimental and quasi-experimental designs for generalized causal inference. 2nd edition. Houghton Mifflin, Michigan,2002. Reference Source

2. SAMHSA. AUDIT-C. https://www.integration.samhsa.gov/images/res/tool_auditc.pdf

3. Babor TF, Higgins-Biddle JC, Saunders JB, Monteiro MG AUDIT: The Alcohol Use Disorders Identification Test Guidelines for Use in Primary Care (second edition). https://www.who.int/substance_abuse/publications/audit/en/

4. US Preventive Service task Force. Patient Health Questionnaire PHQ-9. https://www.uspreventiveservicestaskforce.org/Home/GetFileByID/218

5. Center for Quality Assessment and Improvement in Mental Health Patient Health Questionnaire PHQ-2. http://www.cqaimh.org/pdf/tool_phq2.pdf

6. Gual A, Anderson P, Segura L, et al.: Alcohol and Primary health care: Training programme on identification and brief interventions. Barcelona: department of Health of the Government of Catalonia,2005. Reference Source

7. Anderson P, Bendsten P, Spak F, et al.: Improving the delivery of brief interventions for heavy drinking in primary health care: outcome results of the Optimizing Delivery of Health Care Intervention (ODHIN) five-country cluster randomized factorial trial. *Addiction.* 2016;111(11):1935–1945. 27237081 10.1111/add.13476

8. Wensing M, Oxman A, Baker R, et al.: Tailored Implementation For Chronic Diseases (TICD): a project protocol. *Implement Sci.* 2011;6:103. 21899753 10.1186/1748-5908-6-103 3179734

9. Wensing M, Huntink E, van Lieshout J, et al.: Tailored Implementation of Evidence-Based Practice for Patients with Chronic Diseases. *PLoS One.* 2014;9(7):e101981. 25003371 10.1371/journal.pone.0101981 4087017

10. Flottorp SA, Oxman AD, Krause J, et al.: A checklist for identifying determinants of practice: a systematic review and synthesis of frameworks and taxonomies of factors that prevent or enable improvements in healthcare professional practice. *Implement Sci.* 2013;8:35. 23522377 10.1186/1748-5908-8-35 3617095

11. Prasad A, Kano M, Dagg KA, et al.: Prioritizing action on health inequities in cities: An evaluation of Urban Health Equity Assessment and Response Tool (Urban HEART) in 15 cities from Asia and Africa. *Soc Sci Med.* 2015;145(2015):237–242. 26456133 10.1016/j.socscimed.2015.09.031

12. Svoronos T, Mate KS: Evaluating large-scale health programmes at a district level in resource-limited countries. *Bull World Health Organ.* 2011;89(11):831–837. 22084529 10.2471/BLT.11.088138 3209726

13. Barker PM, Reid A, Schall MW: A framework for scaling up health interventions: lessons from large-scale improvement initiatives in Africa. *Implement Sci.* 2016;11(1):12. 26821910 10.1186/s13012-016-0374-x 4731989

14. Heather N: WHO Collaborative Project on Identification and Management of Alcohol-related Problems in PHC – Report to the World Health Organisation on Phase IV: Development of Country-wide Strategies for Implementing Early Identification and Brief Intervention in PHC. Geneva: World Health Organisation, Department of Mental Health and Substance Abuse:2006. Reference Source

15. Keller, P. A., & Lehmann, D. R. (2008). Designing effective health communications: a meta-analysis. *Journal of Public Policy & Marketing*, *27*(2), 117-130.

16. Rice, R. E., & Atkin, C. K. (Eds.). (2012). *Public communication campaigns*. Sage.

For peer review only - http://bmjopen.bmj.com/site/about/guidelines.xhtml

17. OECD: Compendium of OECD well-being indicators. OECD Better Life Initiative.2011; (Accessed 18 December 2016). Reference Source

18. Bertelsmann Stiftung. [website].2016; (Accessed 18 December 2016). Reference Source

19. Inglehart R, Welzel C: Modernization, Cultural Change and Democracy. New York: Cambridge University Press,2005. Reference Source

20. Anderson P, Clement S: The AAPPQ revisited: the measurement of general practitioners' attitudes to alcohol problems. *Br J Addict.* 1987;82(7):753–9. 3478065 10.1111/j.1360-0443.1987.tb01542.x

21 Riley MR, Mohr DC, Waddimba AC. The reliability and validity of three-item screening measures for burnout: Evidence from group-employed health care practitioners in upstate New York. Stress and Health. 2018 Feb;34(1):187-93.

22 Schaufeli WB, Shimazu A, Hakanen J, Salanova M, De Witte H. An Ultra-Short Measure for Work Engagement: The UWES-3 Validation Across Five Countries. European Journal of Psychological Assessment 2017

23 Rosenberg G, Bauld L, Hooper L et al. New national alcohol guidelines in the UK: public awareness, understanding and behavioural intentions. *Journal of Public Health*, 2018 40, 549–556,

24 Anderson P, Wojnar M, Jakubczyk A, Gual A, Reynolds J, Segura L, Sovinova H, Csemy L, Kaner E, Newbury-Birch D, Fornasin A. Managing alcohol problems in general practice in Europe: results from the European ODHIN survey of general practitioners. Alcohol and Alcoholism. 2014 Jul 16;49(5):531-9.

25 Francis J, Eccles MP, Johnston M, Walker AE, Grimshaw JM, Foy R, Kaner EF, Smith L, Bonetti D. Constructing questionnaires based on the theory of planned behaviour: A manual for health services researchers.

26 Bandura A. Guide for constructing self-efficacy scales. Self-efficacy beliefs of adolescents. 2006 Feb 1;5(1):307-37.

27 Bergström A, Skeen S, Duc DM, Blandon EZ, Estabrooks C, Gustavsson P, Hoa DT, Källestål C, Målqvist M, Nga NT, Persson LÅ. Health system context and implementation of evidence-based practices—development and validation of the Context Assessment for Community Health (COACH) tool for low-and middle-income settings. Implementation Science. 2015 Dec;10(1):120.

28 Scott SD, Plotnikoff RC, Karunamuni N, Bize R, Rodgers W. Factors influencing the adoption of an innovation: An examination of the uptake of the Canadian Heart Health Kit (HHK). Implementation Science. 2008 Dec;3(1):41.

29. Moore GF, Audrey S, Barker M, et al.: Process evaluation of complex interventions: Medical Research Council guidance. *BMJ.* 2015;350:h1258. 25791983 10.1136/bmj.h1258 4366184

30. Reeves BC, Wells GA, Waddington H: Quasi-experimental study designs series-paper 5: classifying studies evaluating effects of health interventions-a taxonomy without labels. *J Clin Epidemiol.* 2017; pii: S0895-4356(17)30288-3. 28351692 10.1016/j.jclinepi.2017.02.016

For peer review only - http://bmjopen.bmj.com/site/about/guidelines.xhtml

SCALA - DATA MANAGEMENT PLAN

Draft version 1: 23 January, 2018
Draft version 2: 1 February, 2018
Draft version 3: 27 February, 2018
Draft version 4: 2 March, 2018
Draft version 5: 15 March, 2018
Draft version 6: 15 May, 2018
Draft version 7: 23 May, 2018
version 8: 24 May, 2018

Abbreviations and definitions:

DMP	= data management plan
IRB	= Institutional Research Board
PHCCs	= primary health care centres
SCALA	= Scale-up of Prevention and Management of Alcohol Use Disorder in Latin America
Data center	= Technische Universität Dresden, Germany (supervisor: Jürgen Rehm)

Contents

1. Data Summary 1
2. FAIR data 5
2.1. Making data findable, including provisions for metadata 5
2.2. Making data openly accessible 5
2.3. Making data interoperable 6
2.4. Increase data re-use (through clarifying licences) 7
3. Allocation of resources 8
4. Data security 9
5. Ethical aspects 10
6. Other issues 11
7. Data analysis plan 12
7.1. REACH 13
7.2. EFFECTIVENESS 14
7.3. ADOPTION 14
7.4. IMPLEMENTATION 15
7.5. MAINTENANCE 15
8. Appendix 18

For peer review only

1. Data Summary

Introduction

During the course of the SCALA study, quantitative, qualitative, as well as publicly available data will be collected in PHCCs in three American countries: Mexico, Peru, Colombia. All collected data are required for a thorough evaluation of the main study goal and it corollaries, ie. to improve alcohol management in PHCCs by increasing screening rates and delivery of adequate advice and treatment for screen positives. The following qualitative and quantitative data will be obtained from patients and providers in PHCCs. All data will be transferred first to the data center serving as SCALA data repository at the TU Dresden (for details on data transfer, see ***section 4***). After cleaning the data and bringing it into the standard format (for details, see ***section 2.2***), the data will be forwarded to partners based on the workplan or upon request. While all data will be kept with the data center, they are collectively owned by all partners.

Data origin

Q1) **PHCC structure data (quantitative):**
Collection of data from the participating PHCCs before start of data collection. The PHCC administration will be asked to fill out a form (see '***Q1_PHCC Description Form.pdf***'), including the number of registered patients, as well as number of health professionals working in the centre. The data will be entered into spreadsheets (see '***Q1_PHCC Description Form_spreadsheet template.xlsx***'), which will then be sent to the data center.

Q2) **Short tally sheet for routine care data (quantitative):**
Collection of routine care data on **all** adult patients consulting PHCCs. For this purpose, a tally sheet (see '***Q2_Short Patient Tally Sheet.pdf***') will be applied to collect all necessary information on sociodemographics (sex, age, socioeconomic status) and drinking patterns (AUDIT-C) for all patients. For screen positives, the tally sheet will also capture the results of in-depth assessment of alcohol problems (AUDIT) and depression (PHQ-2 and - if above threshold - PHQ-9) and the decisions made concerning brief advice and treatment and referral to specialist care. The tally sheets will be collected by local researchers on a weekly basis and entered into spreadsheet templates (see '***Q2_Short Patient Tally Sheet_spreadsheet template.xlsx***'). These spreadsheets will be submitted monthly to the data center.

Q3) **Long tally sheet for quality control data (quantitative):**
Collection by respective PHCC of a more extensive set of routine care data for quality control on **a subset** of adult patients consulting PHCCs. Quality control data will only be collected during predefined periods during the 18 months implementation period, resulting in about 1 in 10 patients being assessed. In order to allow for comparisons between long tally sheet and interview data, the periods for application of long tally sheets will be aligned with realisation of patient interviews. The long tally sheet will cover all variables from the short tally sheet (see Q2 and '***Q3_Long Patient Tally Sheet.pdf***'), in addition to assessment of educational level (1 question), attempts on cutting down drinking (2 questions), alcohol health literacy (4 questions), and injunctive social norms (2 questions). As with short tally sheets, long tally sheets will also be collected weekly by local researchers and entered into spreadsheet templates (see '***Q3_Long Patient Tally Sheet_spreadsheet template.xlsx***'). These spreadsheets will be submitted to the data center whenever data were collected.

For peer review only - http://bmjopen.bmj.com/site/about/guidelines.xhtml

Q4) Tally Sheets Cover Form (quantitative):
Short and long tally sheets will be distributed to the PHCCs by local researchers on a weekly basis and each set of tally sheets will have a cover form (see '***Q4_Tally Sheets Cover Form.pdf***'). On this cover form, the PHCC administration will be asked to fill in the number of adult consultations during the respective week for each participating provider. The cover forms will be collected together with the short/long tally sheets and will be entered in the same spreadsheets and then submitted to the data center.

Q5) Tally Sheet Appendix (consent taking for patient interview):
In predefined weeks during month 3 of the 18-month implementation period, PHCC providers will ask all patients to participate in researcher-conducted personal interviews. Patient consent and contact details will be collected on a form appended to either short or long tally sheets during these weeks (see '***Q5_Patient Tally Sheet Appendix.pdf***'). To allow for a stratified sampling of interviewees according to screening results (ratio of positively and negatively screened patients = 2:1) by local researchers, the providers will also note down the AUDIT-C screening result on the form. These forms will be collected alongside the short/long tally sheets and the data will only be used to sample and recruit interviewees.

Q6) Patient interview data:
Collection of individual data through patient interviews at month 3 and subsequent follow-ups at months 6 and 12. Random samples of positively and negatively screened patients (ratio 2:1) will be interviewed across all municipalities, resulting in a total number of N=1,080 patients. The interview will contain all questions from the long tally sheet (see '***Q3_Long Patient Tally Sheet.pdf***'), in addition to 2 questions for quality control assessing experience of screening/brief advice with PHCC providers, a six-item modified version of the HLS-EU-16 to assess alcohol health literacy, the World Health Organization Disability Assessment Schedule to assess the degree of disability, and questions on health resource utilization (see '***Q6_Patient Interview.pdf***'). The patient interview will be conducted as face-to-face or telephone interview and collected data will be entered into prepared spreadsheets (see '***Q6_Patient interview_spredsheet sample.xlsx***') and sent to the data center.

Q7) Provider questionnaire data (quantitative):
Collection of data from health care providers, which will be assessed prior to or during the 4-week baseline period and repeated at months 4.5 and 13.5. All providers will be asked to fill out questions on alcohol knowledge, alcohol health literacy, as well as on attitudes towards alcohol users and alcohol problems (SAAPP Questionnaire, see '***Q7_Provider questionnaire.pdf***'). The data will be entered into prepared spreadsheets (see '***Q7_Provider questionnaire_spredsheet sample.xlsx***') and sent to the data center.

Q8) Provider interview data (qualitative):
At the end of the 18-month implementation period, a random sample of 1 in 20 PHCC providers of both control and intervention groups will be invited to participate in a 15 minute semi-standardized interview (see '***Q8_Provider Interview from Annexe 25.pdf***'), which will be taped and conducted via telephone. The interviews aim to assess provider experiences on implementing the intervention package in their routines. Recordings of the provider interviews will be transcribed.

Q9) Process data interviews (qualitative):

For peer review only - http://bmjopen.bmj.com/site/about/guidelines.xhtml

As part of the process evaluation, semi-structured focus-group interviews will be conducted with the User Panels, Community Advisory Boards, and local research groups. The focus groups will cover the topics of tailoring of materials, and decision making processes for adoption mechanisms, support systems, and completing driver diagrams and barriers and facilitator tables.

Q10) Recruitment documentation (quantitative):

Local researchers will be given forms to document the entire PHCC recruitment process (see '***Q10_Recruitment documentation.pdf***'). For each municipality, they will document the total number of PHCCs and the number of contacted PHCCs for study participation. Among contacted PHCCs, the number of non-responding, refusing, and accepting PHCCs will be assessed. For each PHCC contacted for study participation, the following data will be assessed: number of registered patients and number of workers, type and number of contacts with PHCC, PHCC response (acceptance, refusal, non-response), and reasons for refusal or non-response if applicable. The data will be entered into prepared spreadsheets (see '***Q10_Recruitment documentation_spreadsheet template.xlsx***') and sent to the data center.

Q11) Follow-up documentation (quantitative):

Local researchers will monitor key activities of each PHCC provider during the course of the study using a standardized sheet (see '***Q11_Follow-up documentation.pdf***'). Key activities to be documented relate to participation in training sessions and potential reasons for non-participation. If providers drop out of the study prior to end of the 18 months implementation period, this will also be documented, in addition to any reasons for drop out. On the same follow-up documentation form, sex and age of the provider will be assessed as well. The data will be entered into prepared spreadsheets (see '***Q11_Follow-up documentation_spreadsheet template.xlsx***') and sent to the data center.

All quantitative data will be collected directly by PHC providers and the country research teams, through patient interviews or provider surveys.

Data types, format, and size

The total size of all quantitative data collected in the course of this study is unlikely to exceed 100MB and will be stored as easily accessible spreadsheets (.csv - format). Transcripts from qualitative interviews will be stored as Microsoft Word documents (.docx - format), not exceeding 100MB in total.

Purpose of data collection with regard to study objectives

The quantitative data will be required to evaluate if study objectives can be reached (for an overview of the study objectives, see '***Figure_RE-AIM.png***'). In particular, Q2 (short tally sheet), Q3 (long tally sheet) and Q4 (patient interview) data will provide outcome measures, which allows for evaluation of the ***REACH*** (maximising exposure to screening and brief advice/treatment in PHC) and ***EFFECTIVENESS*** (increasing adequate alcohol management in PHC) study objectives.

All qualitative data will be obtained through interviews with User Panels, Community Advisory Boards, local research groups, patients and providers, which will be used to evaluate the ***IMPLEMENTATION*** (factors affecting the implementation of intervention package) and ***ADOPTION*** (increase adoption of the intervention package in PHC) study objectives.

For peer review only - http://bmjopen.bmj.com/site/about/guidelines.xhtml

Furthermore, publicly available and process data will be obtained during the course of the study. In detail, this will comprise information necessary to characterize countries, cities and municipalities, contextual, political, socio-economic, and alcohol policy factors (e.g. legislation), and a thorough description of Community Advisory Boards. These data will contribute to the process evaluation (Work Package 5) and serve as base to evaluate the ***MAINTENANCE*** (long term effects of implementation) study objective.

A detailed description of the analytic steps planned to achieve study objectives can be found in ***section 7***.

Re-using data

Most of the data collected during the course of this study will be primary data collected through health care professionals and from patients directly. However, publicly available data form an important pillar in this study as it will be required for process evaluation and economic analyses.

Data utility

The collected data will not only be used to achieve the above listed study goals; they can be used by other researchers to plan similar studies, to examine other hypotheses, or for population modelling purposes.

For peer review only

2. FAIR data

2.1. Making data findable, including provisions for metadata

Making data discoverable, identifiable, and locatable

All quantitative data sets will be made publicly available through the UK Data Service after publication of the results, or, at the latest, 12 months after the finalization of the study.[1] Each data set published with the UK Data Service will be attached with a unique 'Digital Objective Identifier' (DOI).

Data derived from qualitative interviews will not be stored in the UK data archive as anonymity of qualitative interviews cannot be ensured.

Naming conventions and version numbers

For all data sets a predefined title standard ("SCALA_data_*NAME*_v1_*DATE.csv*") and the same author group ("SCALA study group") will always be used. Within titles, consecutive version numbers will be used to facilitate updates and corrections to uploaded data sets and to ensure unambiguous identification of data sets.

Key word conventions

All stored data will be labelled with the following keywords: SCALA, Americas, Mexico, Peru, Colombia, Primary Health Care, Alcohol, Heavy Drinking, Depression, Prevention, Screening, Brief Advice, Treatment. Additional keywords will be considered to characterize the respective data set. As data on resource use will be used for economic analyses, data sets containing relevant data will further be classified using 'JEL Classification Codes'.[2]

Meta data handling

There are no standards on handling metadata in this discipline and there is no intention to manage metadata of the publicly stored data sets apart from the measures listed above.

2.2. Making data openly accessible

Making data openly available

By default, all quantitative datasets generated in the course of the SCALA study will be made openly available through the UK Data Service upon publication of the results. Prior to publication, all data will be formatted to meet UK Data Service requirements.

Access conditions and required software

All quantitative data will be provided as 'comma separated values' (CSV) – an efficient and open source format to store larger data sets. This is a generic, widely used file format, which can be handled by all major software packages used for quantitative analyses (eg. Microsoft Excel, SAS, SPSS, Stata, R). In

[1] http://www.data-archive.ac.uk/
[2] https://en.wikipedia.org/wiki/JEL_classification_codes

For peer review only

order to maintain accessibility, large data sets will be split into smaller parts, which will not exceed 50 MB file size.

Depositing metadata, documentation, and code

Each dataset stored with the UK Data Service will be accompanied by a set of documenting files, which comprises relevant publications, consent forms, questionnaires/interview guidelines, and codebooks. The codebooks stored alongside the dataset will be Excel files (".xlsx") that contain extensive metadata for each variable in the associated data set, such as original questions, value labels, defined missing values, and possible coding rules applied.

Arrangements with the UK Data Service

The UK Data Service has been contacted and the study team received a positive response with regard to storing study data with the service. When preparing files to be published online, guidelines and checklists of the UK Data Service will be considered (see [3,4]). Licence agreements will be finalized after obtaining approval of all IRBs.

Data not being made available

All qualitative data will be generated from semi-standardized interviews. Excerpts of these interviews will be appended to respective publications if applicable. However, full interview transcripts will not be published for the following reasons: first, sharing full interview transcripts is uncommon in this field; and, second, sharing poses a potential risk for disclosing the identity of the interviewee.

Restrictions of use and data access committee

As all relevant data will be made publicly available, there will be no need for a data access committee. If other researchers wish to examine interview transcripts, fully anonymized excerpts can be made available through the responsible researchers.

Ascertainment of identity of person accessing the data

It is aimed that all relevant data are to be shared as 'Open Data'.[5] This will imply that all data will be fully anonymized and there will be no means necessary to ascertain the identity of persons accessing the data.

2.3. Making data interoperable

Interoperability of data

All gathered data will be completely interoperable as they will be stored in widely used data formats, which make them accessible by a broad spectrum of data processing software packages, including open source applications.

[3] https://www.ukdataservice.ac.uk/deposit-data/preparing-data
[4] https://www.ukdataservice.ac.uk/media/440320/depositsurvey.pdf
[5] https://www.ukdataservice.ac.uk/get-data/data-access-policy/open-data

For peer review only - http://bmjopen.bmj.com/site/about/guidelines.xhtml

Data and metadata vocabularies, standards, or methodologies

As there is no standard vocabulary set for variable names in our discipline, a simple and easy-to-comprehend nomenclature will be developed and applied to all quantitative data sets and summarized in accompanying codebooks. For prospective assessments on the same individuals, data sets will be structured in a 'long data format', i.e. one variable will indicate the time of assessment of the same variables (see [6] for a more comprehensive explanation).

2.4. Increase data re-use (through clarifying licences)

Data licence

All study data stored with the UK Data Service will be published as "open data" if possible. For this storage mode, the information in the data set will not allow disclosure of any respondents. "Open data" is published using the Open Government Licence[7] and users will have direct access of data without prior registration with UK data service, facilitating wide reach and potential re-use of data collected in this study.

Time of data availability

All quantitative data sets will be made publicly available after publication of the results, or, at the latest, 12 months after the finalization of the study.

Duration of data storage

All data stored with the UK Data Service are held in perpetuity (see [8]).

Re-use by third parties

Data re-use by third parties is explicitly encouraged and will be facilitated by publication of codebooks and documentation along the data sets.

Data quality assurance processes

Prior to sharing the data with the UK Data Service, the study team will clean the data to ensure internal consistency. Several checks of the study team will be conducted before the data will be shared publicly.

[6] http://www.theanalysisfactor.com/wide-and-long-data/
[7] http://www.nationalarchives.gov.uk/doc/open-government-licence/version/2/
[8] https://www.ukdataservice.ac.uk/media/173249/UKDS_Collections_Development_Policy_02_00.pdf

For peer review only - http://bmjopen.bmj.com/site/about/guidelines.xhtml

3. Allocation of resources

Costs for open access publications

In total, the study budget includes €36,000 to pay 'open access' publication licence fees.

Costs for sharing data through repository

Storage of study data with the UK Data Service does not require any fees.

Long term costs for preservation

No long term costs are anticipated.

Data protection, data transfer and data sharing

The Data Protection Officers of both Technical University Dresden and of Maastricht University are the focal points for reviewing data protection, data transfer and data sharing, and required ethics reporting.

For peer review only

4. Data security

Data security - transfer

All collected data will be transferred to the data center in encrypted packages created with the open access 7-zip software. The 'Advanced Encryption Standard' (AES) with 256 bits will be applied, which has been widely recognized as standard encryption technique [9]. The same data transfer methods will be used to transfer the data to the other partners who request or need the data.

Copies of transcribed data notes that are required for the process evaluation in Work Package 6 will be sent by registered courier to ESADE.

Data security - storage

All electronic data will be stored on encrypted hard drives by respective partners. This will include mail communication, study documentation and codes applied to manipulate data and to generate results. Backup hard drives will be used to facilitate recovery of lost data.

All analogue data sources (tally sheets, interview notes, etc.) will be kept by the local research teams, where the data will be kept and stored adhering to local regulations.

All data stored with the UK Data Service are securely kept for perpetuity.

For peer review only

[9] https://en.wikipedia.org/wiki/Advanced_Encryption_Standard

5. Ethical aspects

Ethical or legal issues regarding data sharing

After collection of the raw data, local researchers will assign predefined identification codes to each individual and remove all potentially identifying information from the data. The key to match individuals to the assigned identification code will remain with the local researchers. After the data has been securely transferred to the data center for cleaning and subsequent analyses, there will be no possibility no identify individuals from the data.

All data collection, processing, and sharing procedures will adhere to national and international laws including the General Data Protection Regulation (EU Regulation 2016/679).

The SCALA study team currently seeks approval for the study design, data collection and analysis from the research ethics board at the TU Dresden, Germany (registration number: 'EK 90032018'). In addition, ethical review is currently under way in Colombia, Mexico, and Peru.

Informed consent for data sharing and long term preservation

Informed consent will be obtained from providers and patients providing individual level data (through interviews or questionnaires) to allow data sharing through the UK Data Service.

For peer review only

6. Other issues

Use of other procedures for data management

Data management in the SCALA study will adhere to EU Regulation 2016/679. There are no further national or institutional requirements which would counteract or extend this regulation or any of the procedures specified in this document.

For peer review only

7. Data analysis plan

In Section 1, data sources are mapped to study goals. For each study goal, the required definition of variables and planned statistical analyses are described in the following.

General considerations

Given that SCALA is a quasi-experimental study design (technically, a non-randomized controlled trial (NRCT)), data for a range of potential confounders will be collected at baseline (with repeat measurements during the course of the 18-month implementation period) both to undertake propensity score matching between intervention and comparator municipalities, and include as confounders in the statistical analyses:

At the level of the PHCC, PHC-provider and patient:

- *Age, sex and profession (doctor, nurse, other health care worker) of provider:* Evidence suggests that the sex and age of the provider are unimportant in influencing screening and advice rates, whereas profession is. Nurses tend to screen more patients than doctors; doctors tend to advise more screen positive patients than nurses.
- *Number of monthly consultations:* Evidence suggests that the higher the number of consultations, the lower the proportion of patients screened.
- *Attitudes and knowledge of providers:* Evidence suggests that providers with more positive attitudes, in terms or role security and therapeutic commitment, and providers with high levels of alcohol-related knowledge, are more likely to screen and advise a greater proportion of patients.
- *AUDIT-C score:* The evidence suggests that the higher the AUDIT-C score, the greater the likelihood that screen positive patients will be given advice.

At the level of the municipality:

- A priori, comparator municipalities have been chosen to be similar to intervention municipalities in terms of socioeconomic and other characteristics which impact on drinking, health care and survival, comparable community mental health services. During the set-up phase, additional data will be collected form the municipalities on existing actions and training of PHC-based screening and brief advice for heavy drinking; availability and accessibility of specialist services for severe AUD and moderately severe or severe depression; and, existing municipal-based prevention and/or policy programmes to reduce heavy drinking

7.1. REACH

Primary outcome measures:

A1 Number of intervened patients per provider and per PHCC

Secondary outcome measures:

A2 Number of screened patients per provider and per PHCC
A3 Number of advised patients per provider and per PHCC
A4 Number of patients referred for severe AUD per provider and per PHCC

For peer review only - http://bmjopen.bmj.com/site/about/guidelines.xhtml

A5 Number of patients referred for moderately severe or severe depression per provider and per PHCC
A6 Provider attitudes
A7 Provider alcohol health literacy
A8 Representativeness of population intervened for AUD

Definition:

Measure A1 represents the *primary* outcome variables in this study and is assessed in three 4-week periods: in the first month 1 (t1), after 9 months (t2) and after 18 months (t3). It will be the proportion of consulting adult patients (aged 18+ years) intervened (screened and advice given to screen positives), calculated as the number of AUDIT-C positive patients that received oral advice or referral for advice to another provider in or outside the PHCC, divided by the total number of adult consultations of the participating providers per provider and per PHCC.

Measures A2 to A5 represent *secondary* outcome variables in this study and are assessed in the same three 4-week periods as measure A1: in the first month 1 (t1), after 9 months (t2) and after 18 months (t3). Measure A2 will be the proportion of patients screened, calculated as the number of completed screens divided by the total number of consultations of all adult patients per participating provider, and averaged per participating PHCC. Measure A3 will be the proportion of patients advised, calculated as the number of brief interventions delivered (received oral brief advice, and/or were referred to another provider in or outside the practice), divided by the total number of screen positives per participating provider and averaged per participating PHCC. Information will also be collected on the number of screen negatives who received brief advice. Measure A4 will be the proportion of patients with severe AUD referred to specialist treatment, calculated as the proportion of patients with an AUDIT-C score ≥8 and a full AUDIT score ≥20 documented as referred to treatment per participating provider, and per participating PHCC. Measure A5 will be the proportion of patients with moderately severe or severe depression referred to specialist treatment, calculated as the proportion of patients with an AUDIT-C score ≥8 and a PHQ-9 score ≥15 documented as referred to treatment per participating provider, and per participating PHCC.

Measures A6 and A7 are also *secondary* outcome variables in this study and will be assessed in three 4-week periods through provider questionnaires: at baseline (t1), after 4.5 months (t2) and after 13.5 months (t3). Measure A6 will be measured by the SAAPP questionnaire, with responses to be summed within the two scales of role security and therapeutic commitment. Individual missing values for any of the items in a domain will be assigned the mean value of the remaining items of the domain before summation. Measure A7 will be assessed through knowledge of risks due to drinking, and reported descriptive and injunctive social norms of drinking. Measure A8 will be determined through process evaluation activities conducted throughout the implementation period. Among other things, representativeness will be evaluated through comparing patients with people living in the catchment area of the respective PHC on a number of variables.

Analyses/Achievement:

For all measures, means and/or proportions (as applicable) will be presented descriptively by country, control and intervention municipality, and for the total sample. Given the relative rarity of some events (eg. measure A1 to A5) and the resulting distribution, we will use exact inference methods for comparison of intervention vs. comparator municipalities.

For peer review only - http://bmjopen.bmj.com/site/about/guidelines.xhtml

For further analyses, including covariates, regression models will be used, taking into consideration the hierarchical nature of the data, and characteristics at different hierarchy levels (i.e., characteristics of the PHCC, characteristics at the municipal level, such as patterns of drinking). Multilevel models are well suited for this purpose and will be built to evaluate the intervention effect for measures A1 to A7. For the primary outcome, the model will be built as follows:

- *Dependent variable:* proportion of patients intervened among all consultations per provider and per PHCC
- *Independent variable 1:* Time (t1-t3)
- *Independent variable 2*: Control vs. intervention municipality
- *Hierarchical cluster*: Provider nested within PHCC nested within country (to control for design effects)
- *Statistic*: Interaction effect between time and group allocation

After testing for the necessary assumptions, the above outlined generalized linear model will be applied to the actual distribution of the outcome measure. Thus, skewness of data resulting from rare events would be analysed using zero-inflated negative binomial regression. For all remaining outcome measures, similar models will be applied.

7.2. EFFECTIVENESS

Outcome measures:

B1 Increased health literacy in PHCC patients using a modified version of the UK-based Newest Vital Sign and a six-item adapted version of Health Literacy Survey-EU Questionnaire (HLS-EU-16)

B2 Reduction in alcohol consumption of AUD+ drinkers

Definition:

Data for measures B1 and B2 are collected through patient interviews (conducted in month 3, 6 and 12).

Analyses/Achievement:

Similar multilevel regression models as applied for primary and secondary outcomes mapped to study goal *REACH* will be applied to measures B1 and B2. The main difference will be that these measures will be analyzed on the individual level, which requires adding another level (patient nested with provider nested within PHCC nested within country) to the model.

7.3. ADOPTION

Outcome measures:

C1 Adoption rate and representativeness of PHCCs

C2 Adoption rate and representativeness of PHCC staff

Definition:

Adoption rate of PHCCs will be calculated as the number of PHCCs agreeing to be part of the study divided by the number of PHCCs contacted.

For peer review only - http://bmjopen.bmj.com/site/about/guidelines.xhtml

Adoption rate of PHCC providers within each PHCC that joins the study will be calculated as the number of PHCC providers agreeing to be part of the study divided by the total number of PHCC providers within each PHCC, stratified by profession (doctor, nurse, other).

Analyses/Achievement:

To determine the representativeness of PHCCs involved in the study, routine available data on the size, number of registered patients, and number and characteristics of staff will be used and compared between PHCCs who agreed to be part of the study and contacted PHCCs who declined to be part of the study.

To determine the representativeness of PHCC staff within the involved PHCC, routine available data on the number and characteristics of staff will be used to compare, within each PHCC, those staff who joined the study and those staff who declined to join the study.

7.4. IMPLEMENTATION

Outcome measures:

D1 Extent primary health care screening and advice package delivered as intended
D2 Multi-level evaluation of barriers/facilitators to scale-up using WHO's Urban Health Equity Assessment and Response Tool
D3 Extent implementation on city levels delivered as intended using Medical Research Council guidance
D4 Cost of package implementation

Definition:

All measures D1 to D3 will be assessed through process evaluation activities. The required data will be obtained through interviews with PHCC providers (D1) and with members from Community Advisory Boards (D2, D3). For D4, a comprehensive set of data will be required, comprising patient data on disability and health resource utilization obtained from patient interviews as well as data on unit costs obtained from public data sources.

Analyses/Achievement:

Measures D1 to D3 will be analyzed through qualitative evaluation. Measure D4 will be evaluated by a comprehensive economic evaluation, for which different sources of costs will be considered, such as costs attributable to implementation of the intervention routine as well as costs attributable to utilization of health care services. In a cost-effectiveness study, the hypothesized gain in quality of life among patients in intervention municipalities will be contrasted with recorded and calculated costs.

7.5. MAINTENANCE

Process measures:

E1 Assessment of outcomes 18 months post implementation
E2 Indicators of program-level maintenance

For peer review only

E3 Measures of cost of maintenance
E4 Dissemination / events

Definition:

For measure E1 data from PHC providers and patients up to 18 months after implementing the alcohol management routine need to be collected.

For measure E2, the required indicators will be collected through process evaluation activities, namely interviews with members of the Community Advisory Boards.

For measure E3, all costs will be collected throughout the implementation period within the economic evaluation framework (see measure D4), in order to estimate the costs of maintenance.

For measure E4, the study results will be disseminated through municipal, national, and international structures, following the 'Communication, Dissemination and Exploitation Plan'.

Analyses/Achievement:

Measure E1 will be achieved by continuous data collection across the entire implementation period of 18 months.

Measure E2 will be achieved by analysis of qualitative data. Measure E3 will be achieved through an economic evaluation of the implementation package considering the entire implementation period.

Measure E4 will be achieved by following the 'Communication, Dissemination and Exploitation Plan'.

For peer review only

8. Appendix

List of all documents referenced in the DMP:

Document	Page Number
1. Q1_PHCC Description Form template.pdf	18
2. Q1_PHCC Description Form_spreadsheet template.xlsx	Excel not attached
3. Q2_Short Patient Tally Sheet.pdf	19
4. Q2_Short Patient Tally Sheet_spreadsheet template.xlsx	Excel not attached
5. Q3_Long Patient Tally Sheet.pdf	22
6. Q3_Long Patient Tally Sheet_spreadsheet template.xlsx	Excel not attached
7. Q4_Tally Sheet Cover Form.pdf	26
8. Q5_Tally Sheet Appendix.pdf	27
9. Q6_Patient Interview.pdf	29
10. Q6_Patient interview_spreadsheet template.xlsx	Excel not attached
11. Q7_Provider questionnaire.pdf	34
12. Q7_Provider questionnaire_spreadsheet template.xlsx	Excel not attached
13. Q8_Provider Interview from Annexe 25.pdf	36
14. Q10_Recruitment documentation.pdf	53
15. Q10_Recruitment documentation_spreadsheet template.xlsx	Excel not attached
16. Q11_Follow-up documentation.pdf	55
17. Q11_Follow-up documentation_spreadsheet template.xlsx	Excel not attached
18. Figure_RE-AIM.png	58

For peer review only

PHCU Description Form

Country and municipality details
(to be filled in by local research team)

**Country**	☐ Colombia	☐ Mexico	☐ Peru
**Municipality**	________________	**Control or Experimental**	☐ Control<br>☐ Experimental
**ID of PHCU**	________________		

PHCU details
(to be filled in by PHC administration)

**Name/Address of PHCU**			________________
**Total number of registered patients**			________________
**Total number of registered *adult* (18+) patients**			________________
**Number of workers working in PHCU**	General Practitioners	**Part time**	________________
		**Full time**	________________
	Nurses	**Part time**	________________
		**Full time**	________________
	Assistants	**Part time**	________________
		**Full time**	________________
	Psychologists	**Part time**	________________
		**Full time**	________________
	Social workers	**Part time**	________________
		**Full time**	________________
	Others: __________	**Part time**	________________
		**Full time**	________________

For peer review only

Short Tally Sheet

Provider details and consultation

**Practice ID** (*pre-printed*)	________________	**Provider ID / Name** (*pre-printed*)	________________
**Date consultation**	____/ ____/ ____		

Patient details

**Sex**	☐ Male<br>☐ Female<br>☐ Other	**Age**	______ years
**Socioeconomic status**	☐ Below average	☐ Average	☐ Above average

AUDIT-C Alcohol Screening

Questions		0	1	2	3	4	Score
1	**How often do you have a drink containing alcohol?**	Never	Monthly or less	2-4 times per month	2-3 times per week	4+ times per week	
2	**How many units of alcohol do you drink on a typical day when you are drinking?**	1-2	3-4	5-6	7-9	10+	
3	**How often do you have 6 or more units on one occasion?**	Never	Less than monthly	Monthly	Weekly	Daily or almost daily	

Standard Drinks Placeholder

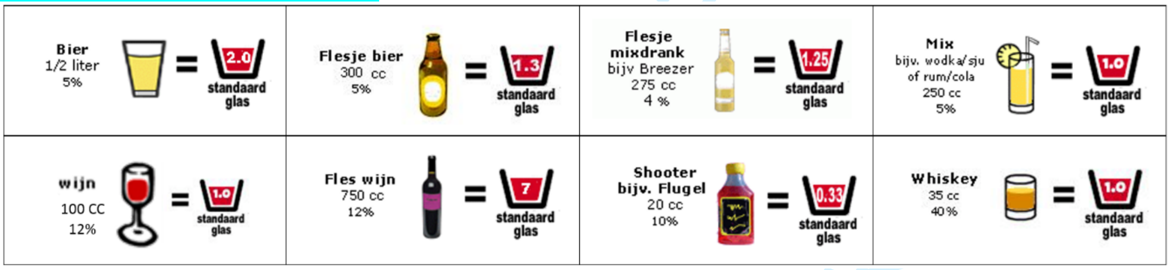

**Sum score AUDIT-C (possible range 0-12)**	__ __
**If AUDIT-C score ≥ 8 => Apply remaining AUDIT and PHQ-2 questionnaire**	

AUDIT (remaining scale)

Questions		0	1	2	3	4	Score
4	**How often during the last year have you found that you were not able to stop drinking once you had started?**	Never	Less than monthly	Monthly	Weekly	Daily or almost daily	
5	**How often during the last year have you failed to do what was normally**	Never	Less than monthly	Monthly	Weekly	Daily or almost daily	

Short Tally Sheet

	**expected from you because of drinking?**						
**6**	**How often during the last year have you needed a first drink in the morning to get yourself going after a heavy drinking session?**	Never	Less than monthly	Monthly	Weekly	Daily or almost daily	
**7**	**How often during the last year have you had a feeling of guilt or remorse after drinking?**	Never	Less than monthly	Monthly	Weekly	Daily or almost daily	
**8**	**How often during the last year have you been unable to remember what happened the night before because you had been drinking?**	Never	Less than monthly	Monthly	Weekly	Daily or almost daily	
**9**	**Have you or someone else been injured as a result of your drinking?**	No		Yes, but not in the last year		Yes, during the last year	
**10**	**Has a relative or friend or a doctor or another health worker been concerned about your drinking or suggested you cut down?**	No		Yes, but not in the last year		Yes, during the last year	
	**Sum score (possible range 0-28)**						__ __
	**Sum score full AUDIT (possible range 0-40)**						__ __
	**If full AUDIT score ≥ 8 => Apply remaining AUDIT and PHQ-2 questionnaire**						

PHQ-2 Depression Screening

**Over the last 2 weeks, how often have you been bothered by any of the following problems?**				
	**Not at all**	**Several days**	**More than half the days**	**Nearly every day**
**1 Little interest or pleasure in doing things**	0	1	2	3
**2 Feeling down, depressed, or hopeless**	0	1	2	3
**Sum score (possible range 0-6)**			__ __	
**If PHQ-2 score ≥ 3 => Apply remaining PHQ questionnaire**				

PHQ-9 (remaining scale)

**Over the last 2 weeks, how often have you been bothered by any of the following problems?**				
	**Not at all**	**Several days**	**More than half the days**	**Nearly every day**
**3 Trouble falling or staying asleep, or sleeping too much**	0	1	2	3
**4 Feeling tired or having little energy**	0	1	2	3
**5 Poor appetite or overeating**	0	1	2	3

For peer review only

Short Tally Sheet

6	Feeling bad about yourself or that you are a failure or have let yourself or your family down	0	1	2	3
7	Trouble concentrating on things, such as reading the newspaper or watching television	0	1	2	3
8	Moving or speaking so slowly that other people could have noticed. Or the opposite being so figety or restless that you have been moving around a lot more than usual	0	1	2	3
9	Thoughts that you would be better off dead, or of hurting yourself	0	1	2	3
	**Sum score (possible range 0-21)**	__ __			
	**Sum score full PHQ-9 (possible range 0-27)**	__ __			

Taking record of brief advice or referral

If full AUDIT < 26 and PHQ-9 < 15:

Brief advice *(more than one answer is possible)*

- ☐ Oral Brief Advice given
- ☐ Patient Leaflet given
- ☐ Continued Monitoring
- ☐ Patient referred to other provider in practice for brief advice
- ☐ Patient referred to other provider outside practice for brief advice
- ☐ Other ________________________
- ☐ Time did not allow, but
  - ☐ I made follow-up appointment
- ☐ Patient declined brief advice
- ☐ Patient not screen positive, but reinforced about keeping low risk drinking habits

If full AUDIT ≥ 26 and/or PHQ-9 ≥ 15:

Patient referred to special services: ☐ Yes ☐ No

For peer review only

Long Tally Sheet

Provider details and consultation

**Practice ID** (*pre-printed*)	________________	**Provider ID / Name** (*pre-printed*)	________________
**Date consultation**	____ / ____ / ____		

Patient details

**Sex**	☐ Male<br>☐ Female<br>☐ Other	**Age**	______ years
**Socioeconomic status**	☐ Below average	☐ Average	☐ Above average
**Highest level of education**	☐ No schooling completed<br>☐ Junior high school completed<br>☐ Business/Technical training<br>☐ Doctorate degree		☐ Primary school completed<br>☐ High school completed<br>☐ Bachelor's/Master's degree

For peer review only

Alcohol exposure, health literacy, and social norms

During the last 12 months have you tried to cut down on your drinking by:		
Choosing lower strength alcohol	☐ Yes	☐ No
Using smaller glasses	☐ Yes	☐ No
How easy is it to understand health information about drinking of alcohol?	☐ Always easy<br>☐ Usually easy	☐ Sometimes difficult<br>☐ Often difficult<br>☐ Always difficult
To the best of your knowledge, can drinking alcohol cause any of the following:		
High blood pressure	☐ Yes	☐ No
Liver problems	☐ Yes	☐ No
Cancer	☐ Yes	☐ No
Thinking about your friends, would you say that it is acceptable or unacceptable for them to drink:		
Regularly more than two drinks a day?	☐ Acceptable	☐ Unacceptable
More than six drinks on an occasion?	☐ Acceptable	☐ Unacceptable

AUDIT-C Alcohol Screening

	Questions	0	1	2	3	4	Score
1	**How often do you have a drink containing alcohol?**	Never	Monthly or less	2-4 times per month	2-3 times per week	4+ times per week	
2	**How many units of alcohol do you drink on a typical day when you are drinking?**	1-2	3-4	5-6	7-9	10+	

Long Tally Sheet

3	How often do you have 6 or more units on one occasion?	Never	Less than monthly	Monthly	Weekly	Daily or almost daily	

Standard Drinks Placeholder

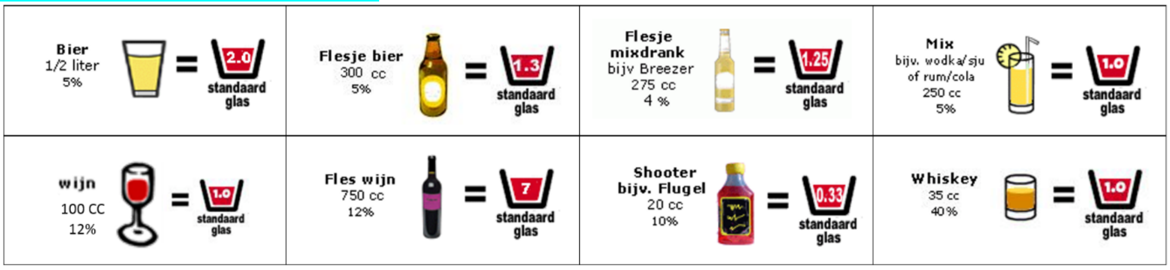

Sum score AUDIT-C (possible range 0-12)	__ __
If AUDIT-C score ≥ 8 => Apply remaining AUDIT and PHQ-2 questionnaire	

AUDIT (remaining scale)

Questions		0	1	2	3	4	Score
4	How often during the last year have you found that you were not able to stop drinking once you had started?	Never	Less than monthly	Monthly	Weekly	Daily or almost daily	
5	How often during the last year have you failed to do what was normally expected from you because of drinking?	Never	Less than monthly	Monthly	Weekly	Daily or almost daily	
6	How often during the last year have you needed a first drink in the morning to get yourself going after a heavy drinking session?	Never	Less than monthly	Monthly	Weekly	Daily or almost daily	
7	How often during the last year have you had a feeling of guilt or remorse after drinking?	Never	Less than monthly	Monthly	Weekly	Daily or almost daily	
8	How often during the last year have you been unable to remember what happened the night before because you had been drinking?	Never	Less than monthly	Monthly	Weekly	Daily or almost daily	
9	Have you or someone else been injured as a result of your drinking?	No		Yes, but not in the last year		Yes, during the last year	
10	Has a relative or friend or a doctor or another health worker been concerned about your drinking or suggested you cut down?	No		Yes, but not in the last year		Yes, during the last year	
	Sum score (possible range 0-28)						__ __
	Sum score full AUDIT (possible range 0-40)						

For peer review only

Long Tally Sheet

	__ __
**If full AUDIT score ≥ 8 => Apply remaining AUDIT and PHQ-2 questionnaire**	

PHQ-2 Depression screening

Over the last 2 weeks, how often have you been bothered by any of the following problems?	Not at all	Several days	More than half the days	Nearly every day
1 Little interest or pleasure in doing things	0	1	2	3
2 Feeling down, depressed, or hopeless	0	1	2	3
Sum score (possible range 0-6)			__ __	
**If PHQ-2 score ≥ 3 => Apply remaining PHQ questionnaire**				

PHQ-9 (remaining scale)

Over the last 2 weeks, how often have you been bothered by any of the following problems?	Not at all	Several days	More than half the days	Nearly every day
3 Trouble falling or staying asleep, or sleeping too much	0	1	2	3
4 Feeling tired or having little energy	0	1	2	3
5 Poor appetite or overeating	0	1	2	3
6 Feeling bad about yourself or that you are a failure or have let yourself or your family down	0	1	2	3
7 Trouble concentrating on things, such as reading the newspaper or watching television	0	1	2	3
8 Moving or speaking so slowly that other people could have noticed. Or the opposite being so figety or restless that you have been moving around a lot more than usual	0	1	2	3
9 Thoughts that you would be better off dead, or of hurting yourself	0	1	2	3
Sum score (possible range 0-21)			__ __	
Sum score full PHQ-9 (possible range 0-27)			__ __	

Taking record of brief advice or referral

**If full AUDIT < 26 and PHQ-9 < 15:**	
**Brief advice** *(more than one answer is possible)*	☐ Oral Brief Advice given<br>☐ Patient Leaflet given<br>☐ Continued Monitoring<br>☐ Patient referred to other provider in practice for brief advice<br>☐ Patient referred to other provider outside practice for brief advice<br>☐ Other<br>________________________<br>☐ Time did not allow, but<br>☐ I made follow-up appointment

For peer review only

☐ Patient declined brief advice
☐ Patient not screen positive, but reinforced about keeping low risk drinking habits

**If full AUDIT ≥ 26 and/or PHQ-9 ≥ 15:**	
**Patient referred to special services:**	☐ Yes<br>☐ No

For peer review only

Tally Sheets Cover Form

Provider details, consultation and type of tally sheets
(to be filled in by local research team)

**Practice ID**	_[pre-print]_	**Provider ID / Name**	_[pre-print]_
**Consultation period**	____ / ____ / ____ - ____ / ____ / ____ (DD / MM / YY)		
**Type of tally sheets**	☐ Short tally sheets	☐ Long tally sheets	

Adult consultations
(to be filled in by PHC provider or administrator)

**Number of adult consultations during consultation period for this provider**	_ _ _ _

For peer review only

Tally Sheet Appendix

PHC provider and consultation details

**Practice ID** (*pre-printed*)	________________	**Provider ID / Name** (*pre-printed*)	________________
**Date consultation**	____/____/____		

Patient interview

**Alcohol screening result**	☐ Positive (AUDIT-C >= 8)	☐ Negative (AUDIT-C < 8)
**Asked patient for interview participation**	☐ Yes	☐ No
**Patient interested in interview participation**	☐ Yes	☐ No

Patient contact details for interview
(only if patient expressed interest in interview participation)

**Name**	______________________________	
**Telephone number**	______________________________	
**Address**	______________________________	
**Preferred mode of interview**	☐ Face-to-face	☐ Telephone

Interview information

Introduction

The SCALA Study aims to find out the extent to which screening and brief advice implemented in primary health care can be increased to reduce the harmful use of alcohol. The study is taking place in cities from three countries from Latin America.
The harmful use of alcohol is prevalent in any countries, and alcohol, itself, is the seventh most important risk factor world-wide for ill-health and premature death (after high blood pressure, tobacco use, high fasting plasma glucose, high body mass index, poor diet, and low birthweight and short gestation).

Aim of the study

In this study, we aim to determine the extent of adequate prevention and management of harmful alcohol use in primary health care settings. Another major objective of this study is to improve the health of patients consulting primary health care centers.
The interview will take about 15 minutes and will cover questions on alcohol consumption, alcohol knowledge, wellbeing, and other health behavior. The same interview will be repeated twice, 3 and

For peer review only

9 months after the initial interview. Due to logistical reasons, not all patients agreed to be interviewed will eventually be asked for participation. If you have not been selected for interview participation, your contact details will be destroyed right away.

Data Handling and Sharing

Participation in this interview is entirely voluntary and you are free to skip any of the interview questions. During the interview, you will be asked questions on your personal wellbeing and health. The collected data will be entered into data bases and personal identifying information (such as name, address, and date of birth) will be replaced with an abstract personal identifier, the key to which remains with the local academic only. The data bases will be submitted to the data center at TU Dresden ('Technische Universität Dresden') in Germany using up-to-date encryption techniques. Here, all study data will be stored on encrypted hard drives and processed for further data analyses to be conducted by the study team. At all times, both analogue and digital data will be stored in secure environments. After publication of the study results, the relevant study data will be shared through the UK Data Service – a non-commercial data respository allowing other researchers to re-use the collected data for an indefinite period of time. All data shared through the UK Data Service will bear no risk of disclosure of the identity of the PHCC or of the participating providers.

Interview consent

Please check box

1. I confirm that I have read and understand the information for participating in the SCALA patient interview and have had the opportunity to ask questions. ☐

1. I consent that my contact details will be given to the SCALA study team and agree that the SCALA study team can use the contact details to ask me for interview participation and for repeating the interview. ☐

2. I understand that my participation is voluntary and that I am free to not participate, without giving any reason. ☐

3. I confirm that I have understand that study data collected through me will be processed at the TU Dresden (Germany) and shared through the UK Data Service. ☐

4.

__________________________ ________________ ____________________

Name of patient | Date | Signature

For peer review only

PATIENT INTERVIEW

Formalities

**Practice ID** *(pre-printed)*	____________	**Provider ID / Name** *(pre-printed)*	____________
**Patient ID** *(filled in by interviewer)*	____________	**Interview date**	____ / ____ / ____

Sociodemographics

For peer review only

**Sex**	☐ Male<br>☐ Female<br>☐ Other	**Age**	______ years
**Socioeconomic status**	☐ Below average	☐ Average	☐ Above average
**Highest level of education**	☐ No schooling completed<br>☐ Junior high school completed<br>☐ Business/Technical training<br>☐ Doctorate degree		☐ Primary school completed<br>☐ High school completed<br>☐ Bachelor's/Master's degree

Alcohol exposure, health literacy, and social norms

During the last 12 months have you tried to cut down on your drinking by:		
Choosing lower strength alcohol	☐ Yes	☐ No
Using smaller glasses	☐ Yes	☐ No
How easy is it to understand health information about drinking of alcohol?	☐ Always easy<br>☐ Usually easy	☐ Sometimes difficult<br>☐ Often difficult<br>☐ Always difficult
In the last 12 months, has any doctor or health worker asked you about how much alcohol you drink?	☐ Yes	☐ No
In the last 12 months, has any doctor or health worker advised you to reduce or stop drinking alcohol?	☐ Yes	☐ No
To the best of your knowledge, can drinking alcohol cause any of the following:		
High blood pressure	☐ Yes	☐ No
Liver problems	☐ Yes	☐ No
Cancer	☐ Yes	☐ No
Thinking about your friends, would you say that it is acceptable or unacceptable for them to drink:		
Regularly more than two drinks a day?	☐ Acceptable	☐ Unacceptable
More than six drinks on an occasion?	☐ Acceptable	☐ Unacceptable

AUDIT Alcohol Screening

Questions		0	1	2	3	4	Score
1	**How often do you have a drink containing alcohol?**	Never	Monthly or less	2-4 times per month	2-3 times per week	4+ times per week	
2	**How many units of alcohol do you drink on a typical day when you are drinking?**	1-2	3-4	5-6	7-9	10+	
3	**How often do you have 6 or more units on one occasion?**	Never	Less than monthly	Monthly	Weekly	Daily or almost daily	

Standard Drinks Placeholder

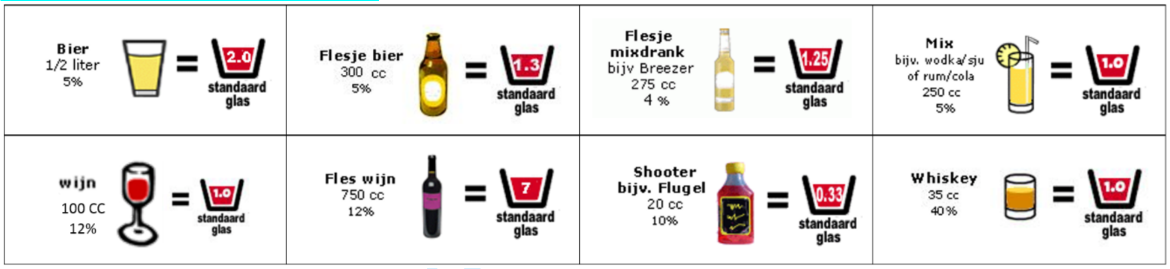

		0	1	2	3	4	Score
4	**How often during the last year have you found that you were not able to stop drinking once you had started?**	Never	Less than monthly	Monthly	Weekly	Daily or almost daily	
5	**How often during the last year have you failed to do what was normally expected from you because of drinking?**	Never	Less than monthly	Monthly	Weekly	Daily or almost daily	
6	**How often during the last year have you needed a first drink in the morning to get yourself going after a heavy drinking session?**	Never	Less than monthly	Monthly	Weekly	Daily or almost daily	
7	**How often during the last year have you had a feeling of guilt or remorse after drinking?**	Never	Less than monthly	Monthly	Weekly	Daily or almost daily	
8	**How often during the last year have you been unable to remember what happened the night before because you had been drinking?**	Never	Less than monthly	Monthly	Weekly	Daily or almost daily	
9	**Have you or someone else been injured as a result of your drinking?**	No		Yes, but not in the last year		Yes, during the last year	
10	**Has a relative or friend or a doctor or another health worker been concerned about your drinking or suggested you cut down?**	No		Yes, but not in the last year		Yes, during the last year	
	**Sum score AUDIT (possible range 0-40)**						__ __

For peer review only - http://bmjopen.bmj.com/site/about/guidelines.xhtml

PHQ-9 Depression Screening

Over the last 2 weeks, how often have you been bothered by any of the following problems?	Not at all	Several days	More than half the days	Nearly every day
1 Little interest or pleasure in doing things	0	1	2	3
2 Feeling down, depressed, or hopeless	0	1	2	3
3 Trouble falling or staying asleep, or sleeping too much	0	1	2	3
4 Feeling tired or having little energy	0	1	2	3
5 Poor appetite or overeating	0	1	2	3
6 Feeling bad about yourself or that you are a failure or have let yourself or your family down	0	1	2	3
7 Trouble concentrating on things, such as reading the newspaper or watching television	0	1	2	3
8 Moving or speaking so slowly that other people could have noticed. Or the opposite being so figety or restless that you have been moving around a lot more than usual	0	1	2	3
9 Thoughts that you would be better off dead, or of hurting yourself	0	1	2	3
Sum score PHQ-9 (possible range 0-27)	__ __			

Alcohol Literacy Assessment

On a scale from very difficult to very easy, how easy would you say it is to: …	Very difficult	Fairly difficult	Fairly easy	Very easy	Don't know
1 Question 1 Placeholder	0	1	2	3	5
2 Question 2 Placeholder	0	1	2	3	5
3 Question 3 Placeholder	0	1	2	3	5
4 Question 4 Placeholder	0	1	2	3	5
5 Question 5 Placeholder	0	1	2	3	5
6 Question 6 Placeholder	0	1	2	3	5
Sum score (possible range XX-XX)	__ __				

For peer review only

WHODAS 2.0 Disability Assessment

This questionnaire asks about difficulties due to health conditions. Health conditions include diseases or illnesses, other health problems that may be short or long lasting, injuries, mental or emotional problems, and problems with alcohol or drugs.

Think back over the past 30 days and answer these questions, thinking about how much difficulty you had doing the following activities. For each question, please circle only one response.

In the past 30 days, how much difficulty did you have in:					
**Questions**	None	Mild	Moderate	Severe	Extreme or cannot do
**1 Standing for long periods such as 30 minutes?**	1	2	3	4	5
**2 Taking care of your household responsibilities?**	1	2	3	4	5
**3 Learning a new task, for example, learning how to get to a new place?**	1	2	3	4	5
**4 Joining in community activities (for example, festivities, religious or other activities) in the same way as anyone else can?**	1	2	3	4	5
**5 How much have you been emotionally affected by your health problems?**	1	2	3	4	5
**6 Concentrating on doing something for ten minutes?**	1	2	3	4	5
**7 Walking a long distance such as a kilometre [*or equivalent*]?**	1	2	3	4	5
**8 Washing your whole body?**	1	2	3	4	5
**9 Getting dressed?**	1	2	3	4	5
**10 Dealing with people you do not know?**	1	2	3	4	5
**11 Maintaining a friendship?**	1	2	3	4	5
**12 Your day-to-day work?**	1	2	3	4	5
**Sum score (possible range 0-60)**	__ __				
**H1 Overall, in the past 30 days, how many days were these difficulties present?**	Record number of days: __ __ (0-30)				
**H2 In the past 30 days, for how many days were you totally unable to carry out your usual activities or work because of any health condition?**	Record number of days: __ __ (0-30)				
**H3 In the past 30 days, not counting the days that you were totally unable, for how many days did you cut back or reduce your usual activities or work because of any health condition?**	Record number of days: __ __ (0-30)				

For peer review only

Health resource utilization

Title Placeholder				
		Response 1	Response 2	Response 3
**1**	Question 1 Placeholder	0	1	2
**2**	Question 2 Placeholder	0	1	2
**3**	Question 3 Placeholder	0	1	2
**4**	Question 4 Placeholder	0	1	2
**5**	Question 5 Placeholder	0	1	2
**6**	Question 6 Placeholder	0	1	2

For peer review only

Primary Health Care Provider Questionnaire

Practice details and date

**Practice ID** (*pre-printed*)	________________	**Provider ID / Name** (*pre-printed*)	________________
**Date**	____ / ____ / ____	**Assessment**	☐ Baseline<br>☐ Follow-up 1<br>☐ Follow-up 2

Patient details

**Sex**	☐ Male<br>☐ Female<br>☐ Other	**Age**	______ years
**Profession**	☐ Doctor<br>☐ Nurse<br>☐ Psychologist		☐ Practice Assistant<br>☐ Social worker<br>☐ Other: ______

Alcohol Knowledge

	Questions	Per Day	Per Week	Per Occasion
1	Experts recommend that everyone should limit the amount of alcohol that they drink. What is this limit for men, in terms of drinks:	_ _ drinks	_ _ drinks	_ _ drinks
2	Experts recommend that everyone should limit the amount of alcohol that they drink. What is this limit for women, in terms of drinks:	_ _ drinks	_ _ drinks	_ _ drinks

		Acceptable	Unacceptable
3	Would you say that it is acceptable or unacceptable for you to drink regularly more than two drinks a day?		
4	Would you say that it is acceptable or unacceptable for you to drink more than six drinks on anyone occasion?		
5	Would you say that it is acceptable or unacceptable for your friends to drink regularly more than two drinks a day?		
6	Would you say that it is acceptable or unacceptable for your friends to drink more than six drinks on anyone occasion?		

Alcohol Health Literacy

On a scale from very difficult to very easy, how easy would you say it is to: …

		Very difficult	Fairly difficult	Fairly easy	Very easy	Don't know
1	Question 1 Placeholder	0	1	2	3	5
2	Question 2 Placeholder	0	1	2	3	5

For peer review only

Primary Health Care Provider Questionnaire

3	Question 3 Placeholder	0	1	2	3	5
4	Question 4 Placeholder	0	1	2	3	5
5	Question 5 Placeholder	0	1	2	3	5
6	Question 6 Placeholder	0	1	2	3	5
	Sum score (possible range XX-XX)	__ __				

The Short Alcohol and Alcohol Problems Perception Questionnaire

	There are no right or wrong answers. Please indicate the extent to which you agree or disagree with the following statements	Strongly disagree	Quite strongly disagree	Disagree	Neither agree or disagree	Agree	Quite strongly agree	Strongly agree
		1	2	3	4	5	6	7
1	I feel I know enough about causes of drinking problems to carry out my role when working with drinkers							
2	I feel I can appropriately advise my patients about drinking and its effects							
3	I feel I do not have much to be proud of when working with drinkers							
4	All in all, I am inclined to feel I am a failure with drinkers							
5	I want to work with drinkers							
6	Pessimism is the most realistic attitude to take towards drinkers							
7	I feel I have the right to ask patients questions about their drinking when necessary							
8	I feel that my patients believe I have the right to ask them questions about drinking when necessary							
9	In general, it is rewarding to work with drinkers							
10	In general, I like drinkers							

For peer review only

Annexe 25 Provider Interview

Telephone Interview of random sample of providers

Approximately 15-minute recorded telephone interview with open-ended questions

Country:

City:

PHCU ID Number:

PHC Provider ID Number:

Why?
Engagement: reasons for participating in the PHC action

How and for whom?
Description of the implementation process for screening and brief advice: description of proceedings and expectations of screening and brief advice

Under what circumstances?
What were the barriers and facilitators to following the guidelines on risky alcohol consumption?

What were the facilitators or barriers to implementing screening and brief advice?

Opinions and suggestions for organisational and political barriers and facilitators

Other thoughts and suggestions to speed up the implementation process

The responses will be analysed and coded according to Keurhorst et al. 2016:

Keurhorst M, Heinen M, Colom J et al. Strategies in primary healthcare to implement early identification of risky alcohol consumption: why do they work or not? A qualitative evaluation of the ODHIN study. Keurhorst et al. BMC Family Practice (2016) 17:70 DOI 10.1186/s12875-016-0461-8

For peer review only

SCALA – Documentation of PHCC Recruitment

1) Please specify the country as well as the name of the researcher responsible for PHCC recruitment:

**Country**	☐ Mexico<br>☐ Colombia<br>☐ Peru
**Responsible researcher**	______________________

2) During recruitment of the PHCCs, local researchers should document the following points *for each municipality*:

**Name of municipality**	______________________
**Control / Intervention**	☐ Control<br>☐ Intervention
**Total number of PHCCs in municipality**	______________________
**Number of PHCCs contacted for study participation**	______________________
**Number of non-responding PHCCs**	______________________
**Number of PHCCs refusing to participate**	______________________
**Number of PHCCs accepting to participate**	______________________

For peer review only

3) Further, the following points need to be documented *for each contacted PHCC*:

**Name/Address/Identifier of PHCC**	______________________
**Characteristics of PHCC (if known)**	☐ Number of registered patients: ______<br>☐ Number of GPs: ______<br>☐ Number of nurses: ______<br>☐ Number of all workers: ______<br>☐ other:<br>_________________________________
**Contact with PHCC**	☐ By mail<br>☐ By email<br>☐ By telephone<br>☐ Personal contact<br>☐ other:<br>_________________________________
**Number of contacts with PHCC before decision (acceptance/refusal/non-response)**	______________________
**Accepted / Refused / No response**	☐ Accepted<br>☐ Refused<br>☐ No response
**If refused, give reasons**	____<br>____<br>____
**If no response, any reasons suspected?**	____<br>____<br>____

For peer review only

SCALA – Provider follow-up documentation

Provider details

During the course of the study, each PHC provider should be followed up with regard to participation in training sessions. Further, potential drop outs should be documented here. Please fill in this sheet ***for each provider.***

**Country**	☐ Mexico<br>☐ Colombia<br>☐ Peru
**Responsible researcher**	____________________
**Name of municipality**	____________________
**Control / Intervention**	☐ Control<br>☐ Intervention
**Name/Address/Identifier of PHCC**	____________________
**Name/Identifier of provider**	____________________
**Gender of provider**	☐ Female<br>☐ Male<br>☐ Other
**Age of provider**	____________________ (in years of age)
**Baseline month**	from ____ / ____ / ____ until ____ / ____ / ____<br>(DD / MM / YY)

For peer review only

Participation in training sessions

**Training session**	☐ Pre-implementation Training 1<br>☐ Pre-implementation Training 2<br>☐ Booster 1<br>☐ Booster 2
**Date of training**	____ / ____ / ____ (DD / MM / YY)
**Training participation**	☐ Participated in training<br>☐ Absent in training
**Reason for training absence**	☐ with valid excuse, ie. __________________<br>☐ without valid excuse
**If absent at training, could training be repeated?**	☐ Yes, on ____ / ____ / ____ (DD / MM / YY)<br>☐ No

For peer review only

Drop out

If the provider dropped out before end of the study, the following section need to be filled in:

**Date of drop out**	____ / ____ / ____ (DD / MM / YY)
**Date of last tally sheet completed by provider**	____ / ____ / ____ (DD / MM / YY)
**Drop out in relation to data collection**	☐ Before baseline data collection<br>☐ During baseline data collection<br>☐ After baseline data collection, but before 18-month implementation period<br>☐ During specific month of 18-month implementation period (enter number of month from 1 to 18).
**Reasons for drop out**	____<br>____<br>____<br>____<br>____

For peer review only

RE-AIM Dimension, SCALA aims	SCALA activities	Main outcome/process measures
**REACH**<br>• In PHC, to maximise exposure to screening for AUD<br>• In PHC, to maximise exposure to advice and treatment for AUD and comorbid depression<br>• In PHC, to maximise exposure to alcohol health literacy information materials	• Recruitment of PHCCs in each city with large population coverage of about 160,000 registered patients per PHCC<br>• Recruitment of representative PHCC population within cities to maximise<br>– Take-up of alcohol health literacy information materials<br>– Numbers screened for AUD<br>– Numbers receiving appropriate advice/referral for AUD/depression	• Total number of PHCC patients screened for AUD<br>• Total number of screen positive patients receiving appropriate advice/referral for AUD/depression<br>• Representativeness of population screened and/or receiving appropriate advice/referral for AUD
**EFFECTIVENESS**<br>• To design and apply an evidence-based care pathway to address AUD and comorbid depression in primary health care	Design and delivery of an intervention package within a primary health care based care pathway that incorporates:<br>• State-of-the-art alcohol health literacy information materials<br>• AUDIT-C screening instrument<br>• Brief advice and treatment for case positives<br>• Referral of severe AUD and comorbid depression	• Increased health literacy in PHCC patients using UK-based Newest Vital Sign and an adapted version of Health Literacy Survey-EU Questionnaire (HLS-EU Q)<br>• Reduction in alcohol consumption of AUD+ drinkers
**ADOPTION**<br>• To increase the adoption of the intervention package in primary health care	• Design of a pragmatic, easy to use and replicate PHCC intervention package and associated care pathway<br>• Tailoring of the PHCC package according to local needs (PHC setting, PHCC) by using Community Advisory Boards (CABs) and User Panels (UPs)<br>• Provision of specific practice-based training and ongoing support to PHCC<br>• Development of city-based adoption mechanisms and support systems	• Adoption rate and representativeness of PHCCs<br>• Adoption rate and representativeness of PHCC staff
**IMPLEMENTATION**<br>• To assess the fidelity and costs of implementing the intervention package<br>• To evaluate which factors affect the implementation of the intervention package	• Continuous feedback on PHCC level drivers to package implementation gathered via qualitative and quantitative metrics<br>• Application of WHO Urban Health Equity Assessment and Response Tool<br>• Application of MRC framework to map and understand progress towards effective scale-up	• Extent primary health care screening and advice package delivered as intended<br>• Multi-level evaluation of barriers/facilitators to scale-up using WHO's Urban Health Equity Assessment and Response Tool<br>• Extent implementation on city levels delivered as intended using Medical Research Council guidance<br>• Cost of package implementation
**MAINTENANCE**<br>• To report on long-term effects of package at individual and organisational levels<br>• To understand how the programme can be maintained and achieve longevity within the test cities	• Support at the system level to make relevant practice changes for sustainability<br>• Monitoring system on long-term effectiveness<br>• Monitoring system on performance on PHCC level<br>• Production of Step-by-step SCALA Framework and Strategy	• Assessment of outcomes 18 months post implementation<br>• Indicators of program-level maintenance<br>• Measures of cost of maintenance<br>• Dissemination / events

BMJ Open

Implementing primary health care-based measurement, advice and treatment for heavy drinking and comorbid depression at the municipal level in three Latin American countries: final protocol for a quasi-experimental study (SCALA study)

Journal:	*BMJ Open*
Manuscript ID	bmjopen-2020-038226.R2
Article Type:	Protocol
Date Submitted by the Author:	06-May-2020
Complete List of Authors:	Jane LLopis, Eva; Ramon Llull University<br>Anderson, Peter; Maastricht Univ; Newcastle University, Institute Health and Society<br>Piazza, Marina; Universidad Peruana Cayetano Heredia<br>O'Donnell, Amy; Newcastle University, Institute of Health and Society<br>Gual, Antoni; Hospital Clínic de Barcelona, Addiction Unit<br>Schulte, Bernard; University Medical Center Hamburg-Eppendorf<br>Perez, Augusto; Corporación Nuevos Rumbos<br>de Vries, Hein; Maastricht University (CAPHRI), Health Promotion; Maastricht University<br>Natera Rey, Guillermina; Instituto Nacional de Psiquiatría Ramón de la Fuente Muñiz<br>Kokole, Dasa; Maastricht University Faculty of Health Medicine and Life Sciences<br>Bustamante, Ines; Universidad Peruana Cayetano Heredia<br>Braddick, Fleur; Hospital Clinic de Barcelona<br>Mejía Trujillo, Juliana; Corporación Nuevos Rumbos<br>Solovei, Adriana; Maastricht Universitair Medisch Centrum+<br>Pérez De León, Alexandra; Instituto Nacional de Psiquiatría Ramón de la Fuente Muñiz<br>Kaner, Eileen; Newcastle University, Institute<br>Matrai, Silvia; Hospital Clinic de Barcelona<br>Manthey, Jakob; Technische Universitat Dresden,<br>Mercken, Liesbeth; Maastricht University<br>López-Pelayo, Hugo; Hospital Clínic de Barcelona, Addiction Unit<br>Rowlands, Gillian; Newcastle University Institute for Health and Society,<br>Schmidt, CS; Universitatsklinikum Hamburg-Eppendorf,<br>Rehm, Jürgen; Centre for Addiction and Mental Health,
**Primary Subject Heading**:	General practice / Family practice
Secondary Subject Heading:	Public health
Keywords:	PRIMARY CARE, Substance misuse < PSYCHIATRY, Depression & mood disorders < PSYCHIATRY

SCHOLARONE™
Manuscripts

I, the Submitting Author has the right to grant and does grant on behalf of all authors of the Work (as defined in the below author licence), an exclusive licence and/or a non-exclusive licence for contributions from authors who are: i) UK Crown employees; ii) where BMJ has agreed a CC-BY licence shall apply, and/or iii) in accordance with the terms applicable for US Federal Government officers or employees acting as part of their official duties; on a worldwide, perpetual, irrevocable, royalty-free basis to BMJ Publishing Group Ltd ("BMJ") its licensees and where the relevant Journal is co-owned by BMJ to the co-owners of the Journal, to publish the Work in this journal and any other BMJ products and to exploit all rights, as set out in our licence.

The Submitting Author accepts and understands that any supply made under these terms is made by BMJ to the Submitting Author unless you are acting as an employee on behalf of your employer or a postgraduate student of an affiliated institution which is paying any applicable article publishing charge ("APC") for Open Access articles. Where the Submitting Author wishes to make the Work available on an Open Access basis (and intends to pay the relevant APC), the terms of reuse of such Open Access shall be governed by a Creative Commons licence – details of these licences and which Creative Commons licence will apply to this Work are set out in our licence referred to above.

Other than as permitted in any relevant BMJ Author's Self Archiving Policies, I confirm this Work has not been accepted for publication elsewhere, is not being considered for publication elsewhere and does not duplicate material already published. I confirm all authors consent to publication of this Work and authorise the granting of this licence.

For peer review only

Implementing primary health care-based measurement, advice and treatment for heavy drinking and comorbid depression at the municipal level in three Latin American countries: final protocol for a quasi-experimental study (SCALA study)

Eva Jané Llopis[1-3]
Peter Anderson[2,4]
Marina Piazza[5]
Amy O'Donnell[4]
Antoni Gual[6-8]
Bernd Schulte[9]
Augusto Pérez Gómez[10]
Hein de Vries[2]
Guillermina Natera Rey[11]
Dasa Kokole[2]
Ines V. Bustamante [5]
Fleur Braddick[7]
Juliana Mejía Trujillo[10]
Adriana Solovei[2]
Alexandra Pérez De León[11]
Eileen Kaner[4]
Silvia Matrai[7]
Jakob Manthey[9,12]
Liesbeth Mercken[2]
Hugo Lopez-Pelayo[6-8]
Gill Rowlands[4]
Christiane Schmidt[9]
Jürgen Rehm[3, 12-15]

[1] Univ. Ramon Llull, ESADE, Barcelona, Spain. Email: eva.jane@esade.edu

[2] Department of Health Promotion, CAPHRI Care and Public Health Research Institute, Maastricht University, POB 616, 6200 MD, Maastricht, The Netherlands. Email: e.llopis@maastrichtuniversity.nl; peter.anderson@maastrichtuniversity.nl; hein.devries@maastrichtuniversity.nl; d.kokole@maastrichtuniversity.nl; a.solovei@maastrichtuniversity.nl; Liesbeth.mercken@maastrichtuniversity.nl

[3] Institute for Mental Health Policy Research, CAMH, 33 Russell Street, Toronto, ON M5S 2S1, Canada. Email: evajanellopis@gmail.com; jtrehm@gmail.com

[4] Population Health Sciences Institute, Newcastle University, Baddiley-Clark Building, Richardson Road, Newcastle upon Tyne NE2 4AX, UK. Emails: Peter.Anderson@newcastle.ac.uk; amy.odonnell@newcastle.ac.uk; eileen.kaner@newcastle.ac.uk

[5]School of Public Health and Administration, Universidad Peruana Cayetano Heredia e-mail: marina.piazza@upch.pe; ines.bustamante@upch.pe

For peer review only - http://bmjopen.bmj.com/site/about/guidelines.xhtml

[6] Red de Trastornos Adictivos. Instituto Carlos III. Sinesio Delgado, 4; 28029 – Madrid. Email: TGUAL@clinic.cat; hlopez@clinic.cat

[7]Addictions Unit. Psychiatry Dept. Hospital Clínic. Villarroel 170; 08036 Barcelona, Spain. Email: TGUAL@clinic.cat, hlopez@clinic.cat; smatrai@clinic.cat; fmbooth@clinic.cat

[8] Institut d'Investigacions Biomèdiques August Pi Sunyer (IDIBAPS); Rosselló, 149-153; 08036 Barcelona, Spain. Email: TGUAL@clinic.cat; hlopez@clinic.cat

[9] Center for Interdisciplinary Addiction Research (ZIS), Department of Psychiatry and Psychotherapy, University Medical Center Hamburg-Eppendorf, Hamburg, Germany. Email: b.schulte@uke.uni-hamburg.de; christiane.schmidt@uke.de; jakobmanthey@snappyquest.org

[10] Corporación Nuevos Rumbos, Calle 108 A # 4-15, Bogotá, Colombia. Email: aperez@nuevosrumbos.org; jmejia@nuevosrumbos.org

[11] Instituto Nacional de Psiquiatría Ramón de la Fuente Muñiz, Calz México-Xochimilco 101, Huipulco, 14370 Ciudad de México, CDMX, Mexico. Email: naterar@imp.edu.mx; apdeleon@imp.edu.mx

[12] Institute for Clinical Psychology and Psychotherapy, TU Dresden, Chemnitzer Str. 46, 01187 Dresden, Germany. Email: jtrehm@gmail.com; jakob.manthey@tu-dresden.de

[13] Dalla Lana School of Public Health, University of Toronto, 155 College Street, 6th Floor, Toronto, ON M5T 3M7, Canada. Email: jtrehm@gmail.com

[14] Department of Psychiatry, University of Toronto, 250 College Street, 8th Floor, Toronto, ON M5T 1R8, Canada. Email: jtrehm@gmail.com

[15] Department of International Health Projects, Institute for Leadership and Health Management, I.M. Sechenov First Moscow State Medical University, Trubetskaya str., 8, b. 2, 119992, Moscow, Russian Federation jtrehm@gmail.com

Corresponding author: Eva Jané Llopis: eva.jane@esade.edu

For peer review only

For peer review only - http://bmjopen.bmj.com/site/about/guidelines.xhtml

Abstract

Introduction: While primary health care-based prevention and management of heavy drinking is clinically effective and cost-effective, it remains poorly implemented in routine practice. Systematic reviews and multi-country studies have demonstrated the ability of training and support programmes for healthcare professionals to increase primary health care-based measurement and brief advice activity to reduce heavy drinking. However, gains have been only modest and short term at best. WHO studies have concluded that a more effective uptake could be achieved by embedding primary health care activity within broader municipal-based support.

Methods and analysis: A quasi-experimental four-arm study will compare primary health care-based prevention and management of heavy drinking and co-morbid depression in three intervention municipal areas from Colombia, Mexico and Peru with three control municipal areas from the same countries. Fifty-four primary health care units will be enrolled. In the implementation municipal areas, 27 primary health care units will receive training on measuring alcohol consumption and managing heavy drinking and comorbid depression embedded within ongoing supportive municipal action over an 18-month implementation test period; 12 units will implement a standard alcohol measurement and advice package (Arm 4), and 15 units a short package (Arm 3). In the control municipal areas, 15 units will receive training (Arm 2), and 12 units will continue with practice as usual (Arm 1). All patients identified as heavy drinkers will be assessed and managed, as appropriate, for comorbid depression. The primary outcome is the proportion of the adult population (aged 18+ years) registered with the unit that has their alcohol consumption measured. Return-on-investment analyses and full process evaluation will be undertaken, coupled with an analysis of potential contextual, financial and political-economy influencing factors.

Ethics and dissemination: The Ethics Committee of the Technical University of Dresden gave final ethical approval for the SCALA project on 12 April 2019, EK90032018. A dissemination strategy is in place with Ministries of Health at municipal and country levels; and, with Pan American Health Organization at Latin American level to scale up the implementation strategy, once validated.

Trial Registration: Clinical Trials.gov ID: NCT03524599; Registered 15 May 2018; https://clinicaltrials.gov/ct2/show/NCT03524599

Protocol Version: Final version, 25 February 2020.

Key words: Primary health care; municipal action; heavy drinking; comorbid depression; Institute for Health Care Improvement; implementation; measurement of alcohol consumption; AUDIT-C.

For peer review only - http://bmjopen.bmj.com/site/about/guidelines.xhtml

Strengths and Limitations of Study

1. Uses a theory-based approach to tailor clinical materials and training programmes, creating city-based Community Advisory Boards, and user-based User Panels to ensure that tailoring matches user needs, municipal services, and co-production of health;
2. Tests the added value of embedding and implementing primary health care activity within municipal-based adoption mechanisms and support systems, and community-based communication campaigns;
3. Has a longer time frame (18 months) than is traditionally used in implementation studies, to assess longer term impacts;
4. Gives considerable emphasis to process evaluation, developing logic models to document the fidelity of all implementation strategies, and to identify, the drivers and barriers and facilitators to successful implementation and scale-up; and
5. Due to municipal-based political and technical considerations, we are unable to randomize the involved municipal areas. We adopt a quasi-experimental design, optimizing comparator municipal areas for confounding, and by using propensity score matching.

For peer review only

For peer review only - http://bmjopen.bmj.com/site/about/guidelines.xhtml

ABBREVIATIONS AND ACRONYMS

AIDS: Acquired Immune Deficiency Syndrome

AUDIT-10: Alcohol Use Disorders Identification Test, full 10-item version

AUDIT-C: Alcohol Use Disorders Identification Test, 3-item consumption version

CAB: Community Advisory Board

HIV: Human Immunodeficiency Virus Infection

IHI: Institute for Healthcare Improvement.

NCD: Non-Communicable Disease

ODHIN: Optimizing Delivery of Health Care Interventions

OECD: Organization for Economic Cooperation and Development

PHC: Primary Health Care

PHCU: Primary Health Care Unit

PHQ-2: Patient Health Questionnaire (mental disorders), 2-item version

PHQ-9: Patient Health Questionnaire (mental disorders), 9-item version

PSM: Propensity Score Matching

RE-AIM: Reach, Effectiveness, Adoption, Implementation, and Maintenance

ROI: Return on Investment

SAAPPQ: Short Alcohol and Alcohol Problems Perception Questionnaire

SAMHSA: Substance Abuse and Mental Health Services Administration

SBIRT: Screening, Brief Intervention and Referral to Treatment

SCALA: Scale-up of Prevention and Management of Alcohol Use Disorders and Comorbid Depression in Latin America

TB: Tuberculosis

UP: User Panel

WHO: World Health Organization

For peer review only - http://bmjopen.bmj.com/site/about/guidelines.xhtml

INTRODUCTION

This paper outlines the protocol for a quasi-experimental study[1] to test the implementation of primary health care-based measurement, advice and treatment for heavy drinking and comorbid depression at the municipal level in three Latin American countries, Colombia, Mexico and Peru (SCALA study).

Heavy drinking is a cause of considerable disability, morbidity, and mortality[2]. Heavy drinking is a causal factor for some communicable diseases (including TB and HIV/AIDS), for many non-communicable diseases (including cancers, cardiovascular diseases and gastrointestinal diseases) and for many mental and behavioural disorders, including depression, dementias and suicide[3,4].

In PHC settings, two-fifths of people with heavy drinking have depression, with risks of incident depression higher for heavier as opposed to lighter drinkers[5]. In addition to its role in the aetiology of depression, heavy drinking is associated with worsening the depression course, including suicide risk, impaired social functioning and impaired health care utilization[6].

Heavy drinking is also a major contributor to global health inequalities, with alcohol-related harm aggravated by lower socio-economic status[7] and extending beyond the individual drinker to families, communities, health systems, and the wider economy. Tackling the multiple individual and societal level harms caused by heavy drinking is essential for achieving global targets of reducing deaths from NCDs by 25% between 2010 and 2025[8], more so as risk of exposure to harmful use of alcohol increases with increasing socio-economic status[9]. In line with tackling harm due to lower socio-economic status, United Nations Sustainable Development Goals include Target 3.5, to strengthen the prevention and treatment of harmful use of alcohol, with two proposed indicators: coverage of treatment interventions (pharmacological, psychosocial and rehabilitation and aftercare services) for harmful use of alcohol; and per capita alcohol consumption[10,11].

Countries in Latin America have the highest alcohol-attributable disease burden after Eastern Europe and Sub-Saharan Africa, with particularly high risks in alcohol-attributable traffic injury including violence[12]. The burden of alcohol-attributable diseases in Latin America lead to marked economic costs, with numerous calls to implement effective and cost-effective policies (e.g.[13]).

A robust and extensive body of literature demonstrates the range of evidence-based strategies that can be implemented to reduce heavy drinking in health care settings[14]. Questionnaire-based measurement and brief advice programmes delivered in PHC are effective[15] and cost-effective[16,17] in reducing heavy drinking. In addition to brief advice, treatment for heavy drinking includes cognitive behavioural therapy and pharmacotherapy, both of which are found to be effective in reducing heavy drinking[18]. Were the proportion of eligible patients receiving advice and treatment for heavy drinking to increase to 30% of eligible patients, the prevalence of harmful use of alcohol could decrease by between 10% and 15% across OECD member countries[19]. However, to date, measurement and brief advice and treatment programmes have failed to achieve widespread take-up[19].

Two systematic reviews[20,21] and two multi-country studies[22-24] have demonstrated that the proportion of PHC patients whose alcohol consumption is measured, and of heavy drinking patients given advice

For peer review only - http://bmjopen.bmj.com/site/about/guidelines.xhtml

can be increased by providing training and support to PHC providers, albeit from very low baseline levels, and with effects not generally sustained over the longer term. Moreover, whilst there has been some previous research in countries of Latin America[25-30], most implementation work to date has been undertaken in high-income countries. The SCALA study will build on previous evidence[31] to fast-track scale-up research and practice in Latin American primary health care settings.

Out of a range of implementation frameworks that include a sequential approach for scale-up, and that provide practical guidance for how to work with organizations, health systems, and communities to implement and scale-up best practices[32-39], we adopt the Institute for Healthcare Improvement's (IHI) Framework for going to Full Scale, which identifies adoption mechanisms and support systems for use across sequential steps, and describes the implementation methods that can be used at each step[40].

SCALA seeks to address three specific barriers to sustained implementation of primary health care-based measurement, advice and treatment for heavy drinking. The first barrier recognizes that most PHC-based programmes focus on providers alone, whereas successful implementation of health interventions within complex health system demands addressing a range of underlying structural and support systems[40]. Phase IV of the WHO study on the identification and management of alcohol-related problems in primary care concluded that embedding PHC-based measurement and brief advice programmes within the frame of supportive community and municipal environments might lead to improved outcomes[41], although this has never been formally evaluated. Similar conclusions were reached by the European ODHIN study[42] and the US-based SAMHSA SBIRT initiative[43-45].

The second barrier is that standard cut-off points for the frequently used alcohol measurement instrument, AUDIT-C[46] (commonly a score of five for both men and women, or five for men and four for women) to trigger advice are too low[47], being equivalent to an average daily alcohol consumption of about 20 grams of alcohol (around 2 standard drinks) or less[48]. Practitioners may well find it problematic to give advice at such levels, which would also have huge time implications, with one in three or four patients being eligible for advice in many countries, under this criterion[24, 49]. We have argued to adopt similar models to blood pressure, where cut-off points for managing raised blood pressure are often determined by levels of blood pressure at which treatment has shown to be effective[50,51]. Similarly, cut-off points for brief advice could be the baseline levels of alcohol consumption found in the randomized controlled trials that have investigated the effectiveness of PHC-delivered brief advice. In the first Cochrane review of the topic that focused on primary health care, mean baseline levels were 313 grams of alcohol per week[52], equivalent to an AUDIT-C cut-off of 8[48].

The third and final barrier concerns the cost of implementing measurement and brief-advice for heavy drinking in primary health care setting. Although, alcohol advice and treatment programmes can lead to substantial reductions in health care costs[16], freeing considerable numbers of working age people from alcohol-related diseases[19], their initial implementation can require a significant time-commitment on the part of providers, in terms of both initial training requirements and the time taken to deliver advice in routine practice. The largest part of the costs of implementing measurement and

For peer review only - http://bmjopen.bmj.com/site/about/guidelines.xhtml

brief advice for heavy drinking in primary health care settings are directly caused by the time spent by the health care providers delivering this intervention[53]. Moreover, this large amount of time is experienced by health care providers as an important barrier to deliver routine measurement and brief advice to their patients[54]. As evidence suggests that shorter sessions of brief advice are not less effective compared to longer sessions[52, 55, 56], it seems that reducing the time spent by health care professionals in preparing for these sessions could be a viable strategy to increase the overall adoption and implementation of alcohol measurement and brief advice at primary health care level.

Given the strong comorbidity between heavy drinking and depression, our protocol includes screening for depression for those patients identified as heavy drinkers, with appropriate referral or PHC support for treatment[57, 58, 59].

In the SCALA study, we implement three interventions (independent variables) for the PHCU:

i. Intensity of clinical package and training (standard, versus short, versus none);
ii. Training of providers (present, versus absent); and,
iii. Community integration and support (municipal action present, versus absent).

The main outcome (dependent variable) is the cumulative proportion of the adult (aged 18+ years) population registered with the PHCU that has their alcohol consumption measured within the 18-month implementation test period (defined as coverage). Three hypotheses are to be tested:

Hypothesis 1: Municipal action leads to more sustainable coverage. After 18 months, the difference in coverage between municipal action present and municipal action absent for those PHCU that receive training is larger than after 12 months;

Hypothesis 2: In the absence of municipal action, PHCU that have received training obtain higher coverage than PHCU that do not receive training; and,

Hypotheses 3: In the presence of municipal action, the short clinical package and short training do not lead to less measurement coverage than the standard clinical package and standard training.

METHODS AND ANALYSIS

The study is a quasi-experimental design[1], comparing changes in measurement and assessment for alcohol consumption and comorbid depression, and, if needed, advice and/or referral for treatment between primary health care units (PHCUs) in intervention municipal areas and PHCUs in similar control municipal areas. In 2017, prior to a grant application, we published a pre-protocol for a three-country study to test the scale-up of primary health care-based programmes to identify and manage the harmful use of alcohol and comorbid depression[60]. Since the application, and during the grant negotiation and planning phase, the design of the study has changed considerably, essentially moving from a two-arm design to a four-arm design, and changing the primary outcome measure to the proportion of the adult population registered with a PHCU that has their alcohol consumption

For peer review only - http://bmjopen.bmj.com/site/about/guidelines.xhtml

measured, Supplement File 1, Box 1. With all changes approved by the concerned ethics committee, this paper outlines the final protocol for a quasi-experimental study to test the implementation of primary health care-based measurement, advice and treatment for heavy drinking and comorbid depression at the community level in three Latin American countries, Colombia, Mexico and Peru (SCALA study).

Intervention municipal areas are investigator-selected from Bogotá (Colombia), Mexico City (Mexico) and Callao – Lima (Peru). Control municipal areas are investigator-selected in the same cities, on the basis of comparability with the intervention municipal area in terms of socio-economic and other characteristics which impact on drinking, health care and survival, comparable community mental health services, and sufficient geographical separation to minimize spill over effects from the intervention municipal area. Randomized selection of the municipal areas was not feasible due to organizational limitations. Municipal areas are chosen as a scalable implementation unit at mesosystem level that can be replicated as the intervention is scaled-up[40], given their jurisdictional responsibilities for prevention and health care services.

Within each intervention municipal area, a local Community Advisory Board (CAB) is created of key stakeholders, including representatives of local and regional government, directors of primary health care services, non-governmental organizations active in providing counselling and treatment services for alcohol and mental health, academic experts, and local media. The CABs meet regularly during the course of the study, giving advice on tailoring materials for local use, giving advice on adoption mechanisms, support systems and communication campaigns to support the action, and preparing for sustainability and scale-up at the end of the action.

The units of allocation and analysis, i.e., study participants, are 54 primary health care units (PHCUs) and the providers working in them. Within each PHCU, eligible providers include any fully trained health care provider working in the PHCU and involved in medical and/or preventive care. Within each PHCU, individual providers decide themselves whether or not to participate in the study; those who do sign an informed consent for their participation. Based on the five-country ODHIN study, we estimate that approximately two-fifths of providers will consent to join the study.[61] The overall study design is summarized in Figure 1. Fifty-four PHCU are invited to join the study until 27 are achieved within each of the two municipal areas (intervention and control) across the three countries (nine per municipal area within each of the three countries).

Within each intervention municipal area, a User Panel is created of providers and patients drawn from the primary health care centres to advise on the tailoring of patient and provider materials and on provider training programmes.

Figure 1 here

Figure 1 Study flow diagram

For the first six months of the 18-month implementation and test period, a four-arm design is adopted,

For peer review only - http://bmjopen.bmj.com/site/about/guidelines.xhtml

Figure 2. Within the comparator municipal area, twelve PHCUs out of the 27 are randomly allocated to control (Arm 1), and 15 are allocated to receive short training to implement a short clinical package (Arm 2). Within the intervention municipal area, in which all 27 PHCU receive municipal action, 15 PHCUs are randomly allocated to receive short training to implement a short clinical package (Arm 3), and twelve PHCUs are allocated to receive standard training to implement a standard clinical package (Arm 4). Random allocation was undertaken using Excel random number generator.

Figure 2 here

Figure 2. Study design for the first six months of the 18-month implementation period

The clinical package comprises measurement instruments, patient information and advice material, and provider guidance material, with the differences between the standard and short clinical materials are described in Supplement File 1, Table 1, with references. Supplement File 1, Table 1 also lists the material used in control Arm 1. The standard material is essentially that used in common clinical practice[60] and the short version a simplified version deliverable in practice during a short period of time. The packages include measurement instruments and patient advice material for comorbid depression implemented with patients with an AUDIT-C score of 8+. Supplement File 1, Table 1 summarizes the differences between the standard and short versions of the training programme.

The standard and short care pathways that are implemented are summarized in Supplement File 1, Figures 1 and 2.

Essentially, in all arms, primary health care providers are asked to measure the alcohol consumption of all adult patients who consult for whatever reason using AUDIT-C. The three AUDIT-C questions are included in a paper tally sheet completed by the provider, in which the providers document the outcome of the consultation (advice given, patient referred etc.). The local researchers visit each PHCU on a two to four weekly basis to collect completed tally sheets and deliver new tally sheets as required. The local researchers collect information on the total number of adult patients (aged 18+ years) registered with each PHCU and the monthly number of total adult consultations with each provider. Patients who score <8 with AUDIT-C are given a patient information leaflet. Patients who score 8+ with AUDIT-C are assessed and manged as appropriate for depression, and are advised to reduce their alcohol consumption, unless there are clinical indications for referral. Arm 4 differs from Arm 3 in having a lengthier assessment, if indicated, and a longer session of advice giving.

By Month 6, Hypotheses 3, i.e., non-superiority of Arm 4 (standard package with municipal action and standard training) over Arm 3 (short package with municipal action and short training) will be tested.

For peer review only - http://bmjopen.bmj.com/site/about/guidelines.xhtml

In the presence of clinical equivalence of a relative difference of the primary outcome, i.e., the cumulative coverage of patients whose alcohol consumption is measured, of less than 10%, Arm 4 will be replaced by Arm 3 from month 8 onwards, Figure 3.

Figure 3 here

Figure 3. Study design from month 8 onwards, assuming no superiority of Arm 4 over Arm 3 during first six months of implementation.

For peer review only

The municipal integration and support inputs to Arms 3 and 4 within the intervention municipal area are summarized in Supplement File 1, Table 2, with references. Municipal integration and support comprises:

i. Creation of local Community Advisory Boards of local stakeholders to advise on tailoring of materials, support local implementation and review drivers of successful action;
ii. Appointment of local project champion to advocate for successful implementation of programmes;
iii. Implementation of five evidence-based adoption mechanisms;
iv. Implementation of five evidence-based support systems; and
v. Implementation of community-based communication campaigns.

Tailoring

The CABs and UPs review and tailor relevant materials of the clinical package and training courses and of the municipal integration and support inputs within the seven domains of: (i) local and national guideline factors; (ii) individual health care provider factors; (iii) patient factors; (iv) interactions between different professional groups; (v) incentives and resources; (vi) capacity for organizational change; and, (vii) social, political and legal factors[62-64].

The study timetable is summarized in Figure 4. The data management plan, as submitted to the European Commission, is available as Supplement File 2.

Figure 4 here

Figure 4. Study timetable.

For peer review only - http://bmjopen.bmj.com/site/about/guidelines.xhtml

Data collection and instruments

1. During set-up phase for Arms 1-4

Municipal level information

At the level of the municipal area (or, when not available, at whole city, regional or country level), the following information will be collected from routinely available data on socio-demographic factors, alcohol and mental health data, health system structures, quality of life, sustainable governance and values, Supplement File 1, Table 3.

PHCU and provider level information

All contacted PHCU, including those who did and did not agree to be part of the study, will provide information on:

- Numbers of registered patients, divided into age 0-17 years and 18+ years; and,
- Numbers and professions of provider staff (including physicians, nurses, nurse technicians, midwifes, psychologists, social workers, and others).

At recruitment, PHC providers will provide data on their:

- Age;
- Gender;
- Profession (doctor, nurse, practice assistant etc.);
- Time worked in the PHC;
- data on their attitudes and experiences to working with patients with heavy drinking and comorbid depression (Supplement File 1, Table 4).

Since we are unable to randomize the municipal areas involved, we will use propensity score matching (PSM) based on data collected at the level of the municipal area and the PHCU, to take into account potential confounding variables between control and intervention municipal areas, and minimise bias on account of these.

2. During one-month baseline measurement period for Arms 1-4

Provider-based measurement and assessment of alcohol consumption and comorbid depression and record of advice and treatment given (tally sheets)

Based on the validated methodology of the ODHIN project[22,24], PHC providers will be asked to document activity by completing anonymous paper tally sheets that record eligible patients' (aged 18+ years) AUDIT-C scores[65], and, if administered (as documented in Supplement File 1, Table 1), AUDIT-10[66], PHQ-2[67] and PHQ-9[68] scores, and the advice or treatment given to each patient. The tally sheets will record the age, sex, and educational level of the patient, the latter as a proxy measure of socio-economic status. PHCUs will return data on the number of adult (aged 18+ years) consultations per provider for the one-month baseline measurement period. Tally sheets will be delivered to the

For peer review only - http://bmjopen.bmj.com/site/about/guidelines.xhtml

PHCU to be distributed to the participating providers at the beginning of the one-month baseline measurement period and collected at the end of the period, with no other contact during the period.

3. During training prior to implementation for Arms 2-4

Providers will complete a short questionnaire after the initial training sessions. The questionnaires, which are adapted based on specific training contents (standard or short package), will assess the participants' experience of the training, measuring satisfaction with the components of the training aspects, as well as their perceived utility. Two measures included in the main provider questionnaires, SAAPPQ[69] and self-efficacy[70], will be included in order to assess the specific impact of the training, independent of the effect of the implementation of the intervention.

4. During 18-month implementation period for Arms 1-4

Provider-based measurement and assessment of alcohol consumption and comorbid depression and record of advice and treatment given (tally sheets)

The same mechanism, for tally sheets used during the baseline measurement period will continue for each calendar month of the 18-month implementation period. Tally sheets will be delivered monthly to each PHCU to distribute to participating providers. Completed tally sheets will be collected at the end of each month. Following training in Arms 2 to 4, and municipal support in Arms 3 to 4, each provider determines use and completion of the tally sheets, with no additional prompting. Monthly data will be collected and reported with accumulation of coverage over time. Formal reporting will be undertaken at baseline, and for coverage achieved by month 12 and by month 18 of the 18-month implementation and test period. Tally sheets will include an identifying code of the provider, PHCU, country and study arm, but no identifying code of the patient. Data will be extracted and sent to the project's data warehouse at Technical University Dresden on a monthly basis.

Extended Tally Sheets

As part of quality control, in all four Arms at two time points, during the 18-month implementation and test period (months 3 and 15), providers will complete extended tally sheets on two separate days in each month. The extended tally sheets will include an identifying code of the provider but no identifying code of the patient. The extended tally sheet will include: additional information from the patient on alcohol knowledge[71], social norms[72] and health literacy[73] applied to alcohol, as it informs the content of advice given; and, additional information from the provider on contextual characteristics that informed their advice giving. The extended tally sheets will include a consent form for the patient and self-completed additional questions for the patient to complete, once the consultation has ended.

Self-completed additional questions by patient

On two separate days, during months 3 and 13, coinciding with and following the consultation with the provider using the extended tally sheet, patients who are able to read and write will be invited to give consent to self-complete additional questions to the extended tally sheet in the waiting room before leaving the PHCU, handing the completed tally sheet and questions to a researcher in

For peer review only - http://bmjopen.bmj.com/site/about/guidelines.xhtml

attendance. No patient identifying information will be included in the questionnaires. Six domains, serving as quality control, will be included:

i. AUDIT-C[65];
ii. PHQ-2[67];
iii. Experiences of the consultation;
iv. Views on being asked about alcohol consumption;
v. Health Literacy[73] as it applies to alcohol; and,
vi. Exposure to communication and media campaigns on alcohol.

On each day, 270 patient questionnaires will be collected across all PHCUs, with up to 1080 (540 during each of months 3 and 13) questionnaires completed in total across the four days.

Provider-based attitudes and experiences.

At two time points during the 18-month implementation period (months 3 and 13), providers will provide data on their attitudes and experiences to working with patients with heavy drinking and comorbid depression, Supplement File 1, Table 4.

Providers will complete a short questionnaire after each of the booster training sessions that they attended (at months 4 and 8). The specific content, number and timing of the training-related questionnaires will depend on the study arm: Arm 2 and 3 participants will fill in one questionnaire after the booster session; while Arm 4 participants will fill in two after each of the two booster sessions.

Observations

The training sessions with the primary health care providers, and the meetings of the CABs will be observed by a neutral observer in order to take note of additional possible barriers in the implementation of the protocol that emerge through the training sessions and meetings. Participant responsiveness will also be observed.

Economic data for return-of-investment analyses

Within SCALA, we will conduct return-on-investment (RoI) analyses, by assessing for each EURO invested in scaling up delivery of screening and brief interventions in primary health care in Columbia, Mexico, and Peru, how many EUROs will be saved by reductions in future health care utilization. The return of investment will be defined as the [return on investment = (gain from investment – cost of investment) / cost of investment]. For details on the data required for RoI analyses, Supplement File 1, Table 5.

For the RoI analyses, the effects of increased coverage of alcohol brief advice among primary health care patients will be modelled using effect sizes from previous meta-analyses[52, 74]. To translate the reduced intake of alcohol into health gains, we will calculate alcohol-attributable fractions for major

For peer review only - http://bmjopen.bmj.com/site/about/guidelines.xhtml

disease and injury categories. These fractions will then be applied to the cost data outlined in Supplement File 1, Table 5 to estimate the alcohol-attributable costs per disease category.

Process evaluation

As the intervention is embedded in a complex system involving actions and actors at different levels (individual, organisational, municipal), a thorough process evaluation will be carried out to complement and better understand the outcomes. Through the process evaluation, the implementation with its fidelity and adaptation will be assessed, along with the drivers of scale-up and contextual factors influencing the implementation, the drivers, and the outcomes. This will be achieved in four blocks: driver diagram creation; barriers and facilitators analysis; assessment of implementation, mechanisms of impact and context; and, further contextual and policy analysis.

Key informant interviews

A number of individual or group interviews will be undertaken throughout the project with key stakeholders – providers, user panel members, CAB members, municipal and primary health care-based clinical leaders, project partners, and any other people involved in the implementation of the SCALA project. Depending on the stakeholder and their involvement in the project, the topics of the interviews will cover topics such as the necessary adaptation to the protocol; the experience of implementing the programme in primary health care practice; and the perception of the municipal support and the community campaigns.

Driver diagrams

Driver diagrams[75] will be used in order to describe the intervention and its causal assumptions, providing the theory of change through displaying what contributes to intervention aim and what are the relationships between primary drivers, secondary drivers and specific change ideas/activities. The initial general driver diagram, Supplement File 1, Figure 3, will be modified based on local contexts and adapted throughout the duration of the project in order to understand how scale up varies in the different cities.

Barriers and facilitators assessment

Factors influencing the implementation of the SCALA protocol will be assessed before the implementation, as well as monitored throughout. The anticipated barriers and facilitators to implementation will be assessed through development of evaluation tool based on literature review[76-78] and implementation framework[62], with subsequent refinement and adaptation to the local context through focus group discussions and workshops with the CABs. The aim of the tool is to identify the barriers that would have to be addressed and monitored throughout implementation and the facilitators that would incentivize and engage providers and the PHCU unit managers in uptake and scaling up of the SCALA protocol. The experienced barriers and facilitators will be further monitored through meeting observations, provider questionnaires and interviews, as well as interviews with

For peer review only - http://bmjopen.bmj.com/site/about/guidelines.xhtml

other involved stakeholders (e.g. CAB members, PHCU managers).

Implementation, mechanisms of impact and context

The factors influencing the progress from scale-up to outcomes will be identified and documented based on UK Medical Research Council guidance[79], analysing factors within five groups: (i) description of intervention and its causal assumptions; (ii) implementation; (iii)mechanisms of impact ; (iv)context ; and, (v) outcomes. All aspects of the intervention will be taken into consideration: the intervention, intervention tailoring, training, training tailoring, as well as the municipal action, consisting of the CABs and the communication campaign, combining both quantitative and qualitative methods in order to obtain a comprehensive picture of the integration and interaction of included variables. A detailed description of the topics of interest and accompanied methods is presented in Supplement File 1, Table 6.

The five groups will be assessed as follows:

i. *Description of the intervention.* The description of the intervention and its causal assumptions draws from the previously described driver diagram;

ii. *Implementation.* Delivery of the training will be assessed though document analysis (reports from training), observation and self-reports from the trainers. Delivery of the intervention will be assessed through document analysis, interviews with patients and providers. The areas of focus will be fidelity, adaptation, dose and reach. Implementation of the CAB meetings and community action will be assessed mainly through document analysis, as well as key informant interviews;

iii. *Mechanisms of impact*. The following three areas will be covered: participant responses to the intervention, mediators and unintended consequences. Mechanisms of impact of intervention delivery will be assessed through patient and providers' questionnaires. The patient interviews will focus on their responsiveness to the intervention, specifically looking at perceived acceptability. In order to evaluate participants' responses to the training, a post-training questionnaire examining satisfaction with the training and perceived utility of training sessions will be applied, triangulated with data from observation and trainers' self-report. Additionally, providers' self-efficacy will be tested as potential mechanism of impact that links the implementation to the outcomes. Mechanisms of impact of the CAB meetings and community action will be examined through key informant interviews and questionnaires. Specific focus will be placed on perceptions and mechanisms of actions of the communication campaign, examining its effect on attitudes and social norms of both providers and patients;

iv. *Context.* Contextual factors that should be considered in order to better understand the success of the intervention will be assessed through meeting observation, document analysis, and provider questionnaires, as well as stakeholder interviews, with the main focus primarily on individual and organisational level characteristics of the context. For the training evaluation, context will be assessed through observation and trainers' self-report. Context of municipal level actions will be assessed through key informant interviews. Additionally, contextual and policy factors on national and municipal levels will be assessed as described below.

For peer review only - http://bmjopen.bmj.com/site/about/guidelines.xhtml

v. *Outcomes.* The data collected through process evaluation will be combined with the outcomes and presented within the RE-AIM framework[80-82], evaluating SCALA's impact across the dimensions of reach, effectiveness, adoption, implementation and maintenance.

Contextual and policy factors

Based on methodology of Ysa et al[83], contextual and policy factors on national and municipal level will be identified through document analysis and key informant interviews. The main variables considered for contextual analysis will be: (1) available data similar to that of the OECD better life initiative[84]; (2) Sustainable Governance Indicators[85]; and, (3) World Values Survey data[86]]. For policy analysis, the information sought will be for a for alcohol policy-related strategies, action plans, legislation and evaluations, both on country and municipal level. The existing contextual and policy factors will be mapped onto the test of the scale-up of the SCALA package to describe and identify those factors on national and municipal level that might influence going to full-scale beyond the tested scalable units.

Outcomes

Primary outcome:

The primary outcome will be the cumulative proportion of the number of adults (aged 18+ years) registered with the PHCU that have their alcohol consumption measured with a completed AUDIT-C instrument during the study period (coverage). The number of adults registered is provided by the administrative office of the PHCU and includes all adult patients covered by the PHCU, whether or not they consult during the 18-month implementation test period.

Secondary outcomes:

- **Proportion of consulting patients who have their alcohol consumption measured by AUDIT-C:** Calculated as the number of adults who have their alcohol consumption measured by AUDIT-C divided by the total number of adults who consult the PHCU during the same time period per participating provider and per PHCU;
- **At risk population receiving advice and/or treatment for heavy drinking:** Calculated as the number of adults with an AUDIT-C score of 8+ who receive brief advice and/or referral for their heavy drinking divided by the total number of patients with an AUDIT-C score of 8+ per participating provider and per PHCU. Information will also be collected on the number of patients with an AUDIT-C score of <8 who receive brief advice and/or treatment for their heavy drinking;
- **Proportion of patients with AUDIT-C score of 8+ who receive assessment for depression:** Calculated as the number of consulting adults with an AUDIT-C score of 8+ who complete PHQ-2 divided by the total number of patients with an AUDIT-C score of 8+ per participating provider and per PHCU;
- **At risk population receiving advice and/or treatment for comorbid depression:** Calculated as the number of adults with a PHQ-2 score of 3+ who receive a patient leaflet and/or referral for their depression divided by the total number of patients with a PHQ-2 score of 3+ per

For peer review only - http://bmjopen.bmj.com/site/about/guidelines.xhtml

participating provider and per PHCU; and,

- **Provider attitudes:** Attitudes of the participating providers will be measured by the short version of the Alcohol and Alcohol Problems Perception questionnaire, SAAPPQ.[65] The responses will be summed within the two scales of role security and therapeutic commitment. Individual missing values for any of the items in a domain will be assigned the mean value of the remaining items of the domain before summation.

Statistical tests of key hypotheses

Primary study goal: Multilevel regression analyses will be undertaken at 12 months' time of the implementation test period, using cumulative results at months 1-12, and at 18 months' time using cumulative results months 1-18. Both analyses will include co-variates of country and results during baseline month, analysed at the levels of the PHCU by study arm, taking into consideration the hierarchical nature of the data. For any PHCU that drops out during the study, outcome values for subsequent measurement points will be set at the last value obtained.

Hypothesis 1

Municipal action leads to more sustainable coverage amongst PHCU that receive training. We will compare results on primary outcome after 18 months with results after 12 months between Arm 3 versus Arm 2 via regression.

<u>Dependent variables:</u>

- For each PHCU, cumulative results of months 1-18 of number of patients whose alcohol consumption is measured with AUDIT-C per 1,000 registered patients; and cumulative results of months 1-12 per 1,000 registered patients.

<u>Random effects:</u>

- Country as random intercept (test for inclusion)

<u>Independent variables:</u>

- Proportion of consulting patients who have their alcohol consumption measured with a completed AUDIT-C instrument during the baseline measurement month
- Condition:
  - Municipal action (yes vs. no)
- Covariate:
  - Proportion of consulting patients who have their alcohol consumption measured with a completed AUDIT-C instrument during the baseline measurement month

It is postulated that coverage for Arm 3 will be significantly higher than for Arm 2.

Hypothesis 2

For peer review only - http://bmjopen.bmj.com/site/about/guidelines.xhtml

Training leads to higher coverage than no training. For both months 1-12 and months 1-18, compare cumulative coverage as per primary outcome between Arms 1 and 2 via multilevel regression analyses.

Dependent variable

- Cumulative results months 1-12, and cumulative results months 1-18 of number of patients whose alcohol consumption is measured with AUDIT-C per 1,000 registered patients with
- PHCU

Random effects:

- Country as random intercept (test for inclusion)

Independent variables:

- Condition:
    - Training (Arm 2 vs. Arm 1)
- Covariate:
    - Proportion of consulting patients who have their alcohol consumption measured with a completed AUDIT-C instrument during the baseline measurement month

It is postulated that coverage for Arm 2 will be significantly higher than for Arm 1.

Hypotheses 3

In the presence of municipal action, the short clinical package and short training do not lead to less coverage than the standard clinical package and standard training. In the presence of clinical equivalence of a relative difference of cumulative coverage of patients screened by less than 10% by month 6, the difference between Arm 3 (all 15 PHCU across the three countries) and Arm 4 (all 12 PHCU across the three countries) will be assessed with regression analyses. If Arm 4 is not superior to Arm 3, both arms will be collapsed into Arm 3 (shorter package) from month 8 onwards.

Dependent variable

- Cumulative results months 1-6 per 1,000 patients

Random effects:

- Country as random intercept (test for inclusion)

Independent variables

- Condition:
    - Length of clinical package (longer = arm 4 vs. shorter = arm 3)
- Covariate:
    - Proportion of consulting patients who have their alcohol consumption measured with a completed AUDIT-C instrument during the baseline measurement month

It is postulated that Arm 4 is not significantly superior to Arm 3.

For peer review only - http://bmjopen.bmj.com/site/about/guidelines.xhtml

Sample size calculations for main hypothesis

As the outcome of the primary study goal is predicted to be Arm3 > Arm2 > Arm1, we compared both Arm 2 > Arm 1, and Arm 3 > Arm 2.

Our power calculations are based on the following assumptions: given an average size of a PHCU of approximately 15,000 adults, with an average of 1500 new consultations per month, we expect a cumulative coverage after 12 months of 0.0325 of the registered adult population to have had their alcohol consumption measured in the control condition (Arm 1) (data extrapolated from month 3 and month 9 assessments of control group from ODHIN study[22,24]; Anderson, personal communication). For the short clinical package and short training (Arm 2), we expect this to increase to 0.075 (data extrapolated from month 3 and month 9 assessments of training group from ODHIN study[22,24]; Anderson, personal communication). Although the WHO Phase IV study predicts an additional beneficial impact of municipal support[41], precise empirical data is not available – however, we consider an estimate for Arm 3, with municipal support, to be 0.15, a proportion that would need to be achieved to consider municipal support to be worthwhile. To detect the difference between Arm 2 and Arm 1, assuming a design effect of 15 PHCUs (clusters) across the three municipal areas in Arm 2, with 15,000 patients (items), and 12 PHCUs (clusters) in Arm 1, with 15,000 patients (items), with an ICC for PHCUs of 0.03 (data from ODHIN study[22,24]; Anderson, personal communication) we would have 82% power at a significance level of 5%[87]. For the difference between Arm 3 and Arm 2 (15 PHCUs/clusters in each arm), we would have 96.5% power.

Patient and public involvement

Patients were not involved in the design of the study but are involved in the tailoring processes. Existing literature suggests that most patients find it acceptable for primary health care providers to ask about their drinking using validated measurement instruments, and support the delivery of brief advice to those drinking above recommended levels[88-96]. However, the majority of the evidence to date draws on research conducted in Europe, and thus the findings are potentially less transferable to Latin American populations. In order to ensure the design and content of the intervention package, including related outcome measures, are appropriate for implementation in the target SCALA sites, we work closely with patients in each city to tailor patient materials. Within the intervention municipal areas in each of the three countries, User Panels are created with representatives of patients from the primary health care centres. As part of the tailoring process, people and patients within the User Panels have the opportunity to comment on the materials and information designed for use by patients. The results of the study will be disseminated directly to patients and the public through information made available via the primary health care units.

DISCUSSION

For peer review only - http://bmjopen.bmj.com/site/about/guidelines.xhtml

The study has several features worth mentioning. It:

1. uses a theory-based approach[62-64] to tailoring clinical materials and training programmes, creating city-based Community Advisory Boards, and user-based User Panels to ensure that tailoring matches user needs, municipal services[97] , and co-production of health[98];
2. sets a higher cut-off score for AUDIT-C (8+) than is commonly used to trigger advice-giving, matching definitions of heavy drinking[99,100], and similar to baseline levels of alcohol consumption in PHC-based trials to reduce heavy drinking[52] . We set the same cut-offs for men and women, based on epidemiological evidence[101], and to minimize unintended consequences of using different cut offs for men and women[102]. We recognize the importance of comorbid depression by building in identification, management, and referral mechanisms[57-59];
3. tests for non-superiority of implementing a standard measurement and 5-minute brief advice intervention with six hours of training, compared with implementing a shorter 1-minute brief advice intervention with three hours of training, taking into account that brief advice is as effective and cost-effective as more extended advice or treatment in reducing heavy drinking[55, 103, 104], and the need for very brief clinical and training programmes for time-constrained providers;
4. tests the added value of embedding and implementing PHC activity within municipal-based adoption mechanisms and support systems[40], and communication campaigns over and above training programmes solely directed to primary health care providers;
5. has a longer time frame (18 months) than is traditionally used in implementation studies[105, 106], to assess longer term impacts; and,
6. gives considerable emphasis to process evaluation[79], developing logic models to document the fidelity of all implementation strategies, and to identify, the drivers and barriers and facilitators to successful implementation and scale-up, and the political and economic contextual factors that might influence scale-up.

There are some limitations to the study design. A trial with random assignment of municipal areas is not feasible due municipal-based political and technical considerations. As we are unable to randomize the involved municipal areas, we adopt a quasi-experimental design[1], trying to optimize control municipal areas for confounding, and by using propensity score matching (PSM). While full comparisons via randomization, and thus establishment of causality, are not possible, together with the qualitative evaluation component of the study, we will be able to clearly identify the mechanisms which were crucial in leading to the outcomes. According to a recent 7-item checklist for classifying quasi-experimental studies for Cochrane reviews[107], our approach is, nevertheless, ranked as a strong design, Supplement Table 7.

Although our focus on embedding PHC activity within supportive municipal actions is hypothesized to increase measurement and brief activity over and above that previously demonstrated, such an approach also brings risks. Municipal and national governments change; and, thus health priorities may change. Although our approach minimizes the need for extra resources (and in some jurisdictions, could be resource saving[19], it is not resource free. Funding constraints could limit future scale-up and

For peer review only - http://bmjopen.bmj.com/site/about/guidelines.xhtml

sustainability.

We have based our protocol adopted on a model of transdisciplinary research to promote sustainability. Such a model identifies, structures, analyses, and deals with specific problems in a way that grasps the complexity of problems[108]; it takes into account the diversity of real-world and scientific perceptions of problems; and develops knowledge and practices that promote what is generally accepted to be the common good[109]. As such, we include municipalities and health systems as stakeholders to form explicitly orchestrated and managed ecosystems that cross organizational boundaries. Municipal areas and health systems create an engagement platform that provides the necessary environment, including people and resources, for sustainability.

ETHICS AND DISSEMINATION

This protocol outlines a quasi-experimental study[1] to test the extent to which embedding PHC-based measurement and brief advice activity within supportive municipal action leads to improved scale-up of an intervention package, with more patients having their alcohol consumption measured, and with heavy drinkers receiving subsequent appropriate advice and treatment. It is not envisaged that there will be any substantial protocol modifications during the course of the study. Any modification to the protocol will be described will be described in all scientific publications.

The Ethics Committee of the Technical University of Dresden gave final ethical approval for the SCALA project on 12 April 2019, EK90032018. All participating primary health care units and participating primary health care providers sign an informed consent form for participation with the country-based research team. Selected patients at two separate time points sign an informed consent form with the country-based research team to provide additional anonymized information following a consultation with a primary health care provider. The consent forms are included within Annexe Data Management Plan. All data collection, processing, and sharing procedures will adhere to national and international laws including the General Data Protection Regulation (EU Regulation 2016/679), as described within the Annexe Data Management Plan.

All materials are publicly available on the project website: https://www.scalaproject.eu/. According to the SCALA data management plan, by default, all quantitative datasets generated in the course of the SCALA study will be made openly available through the UK Data Service upon publication of the results (http://www.data-archive.ac.uk/). Prior to publication, all data will be formatted to meet UK Data Service requirements.

Ministries of Health at municipal and country levels are represented in the Community Advisory Boards created in each intervention municipality to facilitate scale-up at municipal and country levels, once the implementation strategy is validated. SCALA works closely with the Pan American Health Organization (PAHO), with the principal investigator form Mexico being a Collaborating Centre with PAHO, to facilitate scale-up at Latin American levels, once the implementation strategy is validated.

For peer review only - http://bmjopen.bmj.com/site/about/guidelines.xhtml

DECLARATIONS

Ethics approval and consent to participate

The Ethics Committee of the Technical University of Dresden gave final ethical approval for the SCALA project on 12 April 2019, EK90032018. All participating primary health care units and participating primary health care providers sign an informed consent form for participation. Selected patients at two separate time points sign an informed consent form to provide additional anonymized information following a consultation with a primary health care provider.

Consent for publication

No individual person's data will be published in any form.

Availability of data and materials

All materials are publicly available on the project website: https://www.scalaproject.eu/. According to the SCALA data management plan, by default, all quantitative datasets generated in the course of the SCALA study will be made openly available through the UK Data Service upon publication of the results (http://www.data-archive.ac.uk/). Prior to publication, all data will be formatted to meet UK Data Service requirements.

Competing interests

None declared

Funding

The research leading to these results or outcomes has received funding from the European Horizon 2020 Programme for research, technological development and demonstration under Grant Agreement no. 778048 – Scale-up of Prevention and Management of Alcohol Use Disorders and Comorbid Depression in Latin America (SCALA). Participant organisations in SCALA can be seen at: www.scalaproject.eu. The views expressed here reflect those of the authors only and the European Union is not liable for any use that may be made of the information contained therein. The Funder was not involved in the study design. The funder will not be involved in the collection, analysis, interpretation of data, and preparations of any publication.

Authors' contributions

EJL, PA, MP, AO'D, AG, BS, APG, HdV, GNR, DK, IVB, FB, JMT, AS, APdL, EK, SM, JM, LM, HLP, GR, CS, and JR contributed to the Grant Application, on which this protocol is based and adapted. EJL drafted the first version of the paper, and revised the paper based on author's feedback and comments. PA prepared the paper and material for submission and undertook the submission process. EJL, PA, MP, AO'D, AG, BS, APG, HdV, GNR, DK, IVB, FB, JMT, AS, APdL, EK, SM, JM, LM, HLP, GR, CS, and JR commented on drafts of the manuscript and read and approved the final version. PA undertook

For peer review only - http://bmjopen.bmj.com/site/about/guidelines.xhtml

random allocation generation. APG and JMT assigned PHCU to arms in Colombia; GNR and APdL assigned PHCU to arms in Mexico; MP and IVB assigned PHCU to arms in Peru.

Acknowledgements

Not applicable

References

01. Shadish WR, Cook TD, Campbell DT: Experimental and quasi-experimental designs for generalized causal inference. 2nd edition. Houghton Mifflin, Michigan,2002. Reference Source

02. World Health Organization. (2018). Global Status Report on Alcohol and Health 2018. Geneva, Switzerland: WHO Press.

03. Rehm J, Gmel GE, Gmel G et al. The relationship between different dimensions of alcohol use and the burden of disease - an update. Addiction 2017 doi:10.1111/add.13757

04. Odlaug BL, Gual A, DeCourcy J, Perry R, Pike J, Heron L, Rehm J. Alcohol dependence, co-occurring conditions and attributable burden. Alcohol and Alcoholism 2016 51 201-209.

05. Bellos S, Skapinakis P, Rai D et al. Longitudinal association between different levels of alcohol consumption and a new onset of depression and generalized anxiety disorder: Results from an international study in primary care. Psychiatry Research 2016 243 30-34.

06. Boden J. M., Fergusson D. M. Alcohol and depression. Addiction 2011; 106: 906–14.

07. Probst C, Roerecke M, Behrendt S, Rehm J. Socioeconomic differences in alcohol-attributable mortality compared with all-cause mortality: A systematic review and meta-analysis. *Int J Epidemiol* 2014; **43**(4): 1314-27

08. Kontis V, Mathers, CD, Rehm J, Stevens G A, Shield K D, Bonita R, et al. Contribution of six risk factors to achieving the "25×25" NCD mortality reduction target. Lancet 2014 http://dx.doi.org/10.1016/ S0140-6736(14)60616-4.

09. Kontis V, Mathers CD, Bonita R et al. Regional contributions of six preventable risk factors to achieving the 25 × 25 non-communicable disease mortality reduction target: a modelling study. *Lancet Glob Health* 2015; 3: e746–57

10. United Nations General Assembly. Political declaration of the High-level Meeting of the General Assembly on the Prevention and Control of Non-communicable Diseases. http://www.un.org/en/ga/ncdmeeting2011/ Acessed 01/02/2017

11. GBD 2015 SDG Collaborators. Measuring the health-related Sustainable Development Goals in 188 countries: a baseline analysis from the Global Burden of Disease Study 2015. Lancet 2016 388 1813-50.

For peer review only - http://bmjopen.bmj.com/site/about/guidelines.xhtml

12. Shield, K.D., Manthey, J., Rylett, M., Probst, C., Wettlaufer, A., Parry, C.D.H. & Rehm, J. (2020). National, regional, and global burdens of disease from 2000 to 2016 attributable to alcohol use. Lancet Public Health, 5(1), e51–61. doi: 10.1016/S2468-2667(19)30231-

13. Economic Dimensions of Noncommunicable Diseases in Latin America and the Caribbean: Policy Agenda Considerations 143 George Alleyne. In: http://iris.paho.org/xmlui/bitstream/handle/123456789/28501/9789275119051_eng.pdf?sequence=1&isAllowed=y

14. Levin C, Chisholm D. Cost-Effectiveness and Affordability of Interventions, Policies, and Platforms for the Prevention and Treatment of Mental, Neurological, and Substance Use Disorders. In Patel, V., D. Chisholm., T. Dua, R. Laxminarayan, and M. E. Medina-Mora, editors. 2015. *Mental, Neurological, and Substance Use Disorders*. Disease Control Priorities, third edition, volume 4. Washington, DC: World Bank. doi:10.1596/978-1-4648-0426-7.

15. O'Donnell, A., Anderson, P., Newbury-Birch, D., Schulte, B., Schmidt, C., Reimer, J. & Kaner, E. The Impact of Brief Alcohol Interventions in Primary Healthcare: A Systematic Review of Reviews. Alcohol and Alcoholism doi: 10.1093/alcalc/agt170.

16. Angus C, Thomas C, Anderson P, et al. Estimating the cost-effectiveness of brief interventions for heavy drinking in primary health care across Europe. Eur J Public Health. 2017;27:345–351.

17. Rehm J & Barbosa C. (2018) The cost-effectiveness of therapies to treat alcohol use disorders, Expert Review of Pharmacoeconomics & Outcomes Research, 18:1, 43-49, DOI: 10.1080/14737167.2018.1392241

18. Rehm, J., Shield, K. D., Gmel, G., Rehm, M. X., & Frick, U. (2013). Modeling the impact of alcohol dependence on mortality burden and the effect of available treatment interventions in the European Union. European Neuropsychopharmacology, 23(2), 89–97.

19. Organisation for Economic Co-operation and Development. *Tackling Harmful Alcohol Use: Economics and Public Health Policy*. Paris: Organisation for Economic Co-operation and Development, 2015.

20. Anderson, P., M. Laurant, E. Kaner, M. Wensing and R. Grol (2004). "Engaging general practitioners in the management of hazardous and harmful alcohol consumption: results of a meta-analysis." J Stud Alcohol 65(2): 191-199.

21. Keurhorst M, van de Glind I, Bitarello do Amaral-Sabadini M, Anderson P, Kaner E, Newbury-Birch D, Braspenning J, Wensing M, Heinen M, Laurant M (2015) Determinants of successful implementation of screening and brief interventions for hazardous and harmful alcohol consumption in primary healthcare. A systematic review and meta-regression analysis. Addiction Addiction 110:877–900.

22. Anderson, P., Gual, T., Coulton, S., Kaner, E., Bendsten, P., Kłoda, K., Reynolds, J., Keurhorst., M. N., Segura, Wojnar, M., Mierzecki, A., Deluca, P., Newbury-Birch, D., Parkinson, K., Okulicz- Kozaryn, K., Drummond, C., Laurant, M. (2017) Improving the delivery of brief interventions for heavy drinking in primary health care: nine month outcomes of the ODHIN five country cluster randomized factorial trial. Annals of Family Practice. In Press.

For peer review only - http://bmjopen.bmj.com/site/about/guidelines.xhtml

23. Anderson, P., E. Kaner, S. Wutzke, M. Funk, N. Heather, M. Wensing, R. Grol, A. Gual, L. Pas and W. H. O. B. I. S. Group (2004). "Attitudes and managing alcohol problems in general practice: an interaction analysis based on findings from a WHO collaborative study." Alcohol Alcohol 39(4): 351-356.

24. Anderson, P., Bendsten, P., Spak, F., Reynolds, J., Drummond, C., Segura, L., Keurhorst., M. N., Palacio-Vieira, J., Wojnar, M., Parkinson, K., Colom, J., Kłoda, K., Deluca, P., Baena, B., Newbury-Birch, D., Wallace, P., Heinen, M., Wolstenholme, A., van Steenkiste, B., Mierzecki, A., Okulicz-Kozaryn, K., Ronda, G., Kaner, E., Laurant, M., Coulton, S., Gual, T. (2016) 'Improving the delivery of brief interventions for heavy drinking in primary health care: outcome results of the ODHIN five country cluster randomized factorial trial' Addiction DOI: 10.1111/add.13476.

25. Carlos Campillo Serrano, Rosa Díaz Martínez, Martha P. Romero Mendoza, Juan Cerrud Sánchez, Jorge Villatoro (1992). La intervención eficaz del médico general en el tratamiento de bebedores cuyo hábito alcohólico representa un riesgo para su salud o ya les ha ocasionado algún daño. (Preliminary results of a prospective double blind clinical trial). *Salud mental*, 15 (2).

26. Hoffman KA, Beltrán J, Ponce J, García-Fernandez L, Calderón M, Muench J, et al. Barreras para implementar el despistaje, intervenciones breves y referencia al tratamiento por problemas de consumo de alcohol y otras drogas en hospitales que atienden personas que viven con el VIH/SIDA en el Perú. *Rev Peru Med Exp Salud Publica*. 2016;33(3):432-7.

27. Lillian Gelberg, Guillermina Natera Rey, Ronald M. Andersen, Miriam Arroyo, Ietza Bojorquez-Chapela, Melvin W. Rico, Mani Vahidi, Julia Yacenda-Murphy, Lisa Arangua & Martin Serota (2017): Prevalence of Substance Use Among Patients of Community Health Centers in East Los Angeles and Tijuana, *Substance Use & Misuse*, 52:3, 359-372

28. Soto-Brandt, G. et al, (2013). Evidencia de validez en Chile del Alcohol, Smoking and Substance Involvement Screening Test (ASSIST). *Adiccione*s vol.26 (4).

29. Costa PH, Mota DC, Cruvinel E, Paiva FS, Ronzani TM. (2013). A methodology to implement preventive actions against harmful drug use in the context of primary health care in Latin America *Rev Panam Salud Publica*. 33(5):325-31.

30. Natera R et al., 2014: Final Narrative Report of the bi-national assist screening and quit using drugs intervention trial (quit) Tijuana/Los Angeles. Mexico, National Institute on Psychiatry de la Fuente Muniz.

31. Kaner E. Brief alcohol interventions – time for translational research. Addiction 2010; 105: 960-961.

32. Eccles M, Grimshaw J, Walker A, et al.: Changing the behavior of healthcare professionals: the use of theory in promoting the uptake of research findings. *J Clin Epidemiol.* 2005;58(2):107–112. 15680740 10.1016/j.jclinepi.2004.09.002

33. Norton W, Mittman B: Scaling-up Health Promotion/Disease Prevention Programmes in Community Settings: Barriers, Facilitators, and Initial Recommendations. West Hartford, Connecticut, The Patrick and Catherine Weldon Donaghue Medical Research Foundation.2010. Reference Source

For peer review only - http://bmjopen.bmj.com/site/about/guidelines.xhtml

34. Woolf SH, Johnson RE: The break-even point: when medical advances are less important than improving the fidelity with which they are delivered. *Ann Fam Med.* 2005;3(6):545–552. 16338919 10.1370/afm.406 1466946

35. Massoud R: An approach to rapid scale-up using HIV/AIDS treatment and care as an example. Geneva: World Health Organization;2004. Reference Source

36. Cooley L, Kohl R: Scaling up—from vision to large-scale change: a management framework for practitioners. Washington D.C: Management Systems International;2006. Reference Source

37. Damschroder LJ, Aron DC, Keith RE, et al.: Fostering implementation of health services research findings into practice: a consolidated framework for advancing implementation science. *Implement Sci.* 2009;4:50. 19664226 10.1186/1748-5908-4-50 2736161

38. Yamey G: Scaling up global health interventions: a proposed framework for success. *PLoS Med.* 2011;8(6):e1001049. 21738450 10.1371/journal.pmed.1001049 3125181

39. Adamou B: Guide for monitoring scale-up of health practices and interventions: MEASURE evaluation.2013; Accessed 4 November 2016. Reference Source

40. Barker PM, Reid A, Schall MW: A framework for scaling up health interventions: lessons from large-scale improvement initiatives in Africa. *Implement Sci.* 2016;11(1):12. 26821910 10.1186/s13012-016-0374-x 4731989

41. Heather N. editor. WHO Collaborative Project on Identification and Management of Alcohol-related Problems in Primary Health Care – Report to the World Health Organisation on Phase IV: Development of Country-wide Strategies for Implementing Early Identification and Brief Intervention in Primary Health Care. Geneva: World Health Organisation, Department of Mental Health and Substance Abuse: 2006. http://www.who.int/substance_abuse/publications/identification_management_alcoholproblems_phaseiv.pdf

42. Keurhorst M, Heinen M, Colom J et al. Strategies in primary healthcare to implement early identification of risky alcohol consumption: why do they work or not? A qualitative evaluation of the ODHIN study. Keurhorst et al. BMC Family Practice (2016) 17:70 DOI 10.1186/s12875-016-0461-8.

43. Babor T, Del Boca F, Bray JW. Screening, brief intervention and referral to treatment: implications of SAMHSA's SBIRT initiative for substance abuse policy and practice. Addiction 2017 112 (Supple. 2) 110-117.

44. Vendetti J., Gmyrek A., Damon D., Singh M., McRee B., Del Boca F. Screening, Brief Intervention and Referral to Treatment (SBIRT): implementation barriers, facilitators and model migration. Addiction 2017; 112 (Suppl. 2), 23–33.

45. Singh M., Gmyrek A., Hernandez A., Damon D., Hayashi S. Sustaining Screening, Brief Intervention and Referral to Treatment (SBIRT) services in health-care settings. Addiction 2017; 112 (Suppl. 2), 92–100.

46. Jonas DE, Garbutt JC, Brown JM, Amick HR, Brownley KA, Council CL, et al. Screening, Behavioral Counseling, and Referral in Primary Care to Reduce Alcohol Misuse. Comparative Effectiveness

For peer review only

For peer review only - http://bmjopen.bmj.com/site/about/guidelines.xhtml

Review No. 64. Rockville, MD: Agency for Healthcare Research and Quality; July 2012. Accessed at www.ncbi .nlm.nih.gov/books/NBK99199/ on 16 April 2016.

47. Rehm J, Anderson P, Manthey J, Shield KD, Struzzo P, Wojnar M, Gual A. (2016). Alcohol use disorders in primary health are: what do we know and where do we go? Alcohol and Alcoholism 51 422-427.

48. Rubinsky, A.D., Dawson, D.A., Williams, E.C., Kivlahan, D.R. & Bradley, K.A. AUDIT-C Scores as a Scaled Marker of Mean Daily Drinking, Alcohol use disorders Severity, and Probability of Alcohol Dependence in a U.S. General Population Sample of Drinkers. Alcoholism: Clinical and Experimental Research 2013 37 1380-1390.

49. Rehm J., Shield K. D., Rehm M. X., Gmel G., Frick U. Alcohol Consumption, Alcohol Dependence, and Attributable Burden of Disease in Europe: Potential Gains From Effective Interventions for Alcohol Dependence. Toronto, Canada: Centre for Addiction and Mental Health; 2012.

50. National Clinical Guideline Centre (2011). The clinical management of primary hypertension in adults. 2011 https://www.nice.org.uk/guidance/cg127/evidence/cg127-hypertension-full-guideline3 (accessed 1 December 2016).

51. Rehm, J., Anderson, P., Arbesu Prieto, J.A., Armstrong, I., Aubin, H.-J., Bachmann, M., Bastida Bastús N, Brotons, C., Burton, R., Cardoso, M., Colom, J., Duprez, D., Gmel, G., Gual, A., Kraus, L., Kreutz, R., Liira, H., Manthey, J., Møller, L., Okruhlica, L., Roerecke, M., Scafato, E., Schulte, B., Segura-Garcia, L., Shield, K.D., Sierra, C., Vyshinskiy, K., Wojnar, M., & Zarco, J. (2017). Towards new recommendations to reduce the burden of alcohol-induced hypertension in the European Union. BMC Medicine, 15, 173. doi: 10.1186/s12916-017-0934-1.

52. Kaner EF, Beyer FR, Dickinson HO, *et al.*: Effectiveness of brief alcohol interventions in primary care populations. *Cochrane Database Syst Rev.* 2007; (2): CD004148.

53 Bray, J. W., Zarkin, G. A., Hinde, J. M., & Mills, M. J. (2012). Costs of alcohol screening and brief intervention in medical settings: a review of the literature. *Journal of studies on alcohol and drugs*, *73*(6), 911-919. doi: 10.15288/jsad.2012.73.911

54. Groves, P., Pick, S., Davis, P., Cloudesley, R., Cooke, R., Forsythe, M., & Pilling, S. (2010). Routine alcohol screening and brief interventions in general hospital in-patient wards: acceptability and barriers. *Drugs: education, prevention and policy*, *17*(1), 55-71.doi: 10.3109/09687630802088208

55. Platt L, Melendez-Torres GJ, O'Donnell A, et al.: How effective are brief interventions in reducing alcohol consumption: do the setting, practitioner group and content matter? Findings from a systematic review and metaregression analysis. *BMJ Open.* 2016;6(8):e011473. 27515753 10.1136/bmjopen-2016-011473 4985973

56. Kaner, E., Bland, M., Cassidy, P., Coulton, S., Dale, V., Deluca, P., ... & Newbury-Birch, D. (2013). Effectiveness of screening and brief alcohol intervention in primary care (SIPS trial): pragmatic cluster randomised controlled trial. *Bmj*, *346*, e8501. doi: 10.1136/bmj.e8501

57. Wilson GB, Wray C, McGovern R, et al.: Intervention to reduce excessive alcohol consumption and improve comorbidity outcomes in hypertensive or depressed primary care patients: two parallel

For peer review only - http://bmjopen.bmj.com/site/about/guidelines.xhtml

cluster randomized feasibility trials. *Trials.* 2014;15:235. 24947447 10.1186/1745-6215-15-235 4076249

58. Linde K, Sigterman K, Kriston L, et al.: Effectiveness of psychological treatments for depressive disorders in primary care: systematic review and meta-analysis. *Ann Fam Med.* 2015;13(1):56–68. 25583894 10.1370/afm.1719 4291267

59. Gilbody S, Whitty P, Grimshaw J, et al.: Educational and organizational interventions to improve the management of depression in primary care: a systematic review. *JAMA.* 2003;289(23):3145–3151. 12813120 10.1001/jama.289.23.3145

60. Anderson P, O'Donnell A, Kaner E *et al.* Scaling-up primary health care-based prevention and management of alcohol use disorder at the municipal level in middle-income countries in Latin America: Background and pre-protocol for a three-country quasi-experimental study. *F1000Research* 2017, 6:311 (doi: 10.12688/f1000research.11173.1).

61. Anderson P, Kłoda K, Kaner E, Reynolds J, Bendtsen P, Pelgrum-Keurhorst MN, Segura L, Wojnar M, Mierzecki A, Deluca P, Newbury-Birch D, Parkinson K, Okulicz-Kozaryn K, Drummond C, Laurant MGH, Gual A. Impact of practice, provider and patient characteristics on delivering screening and brief advice for heavy drinking in primary healthcare: Secondary analyses of data from the ODHIN five-country cluster randomized factorial trial. Eur J Gen Pract. 2017 23):241-245. doi: 10.1080/13814788.2017.1374365.

62. Flottorp SA, Oxman AD, Krause J, et al.: A checklist for identifying determinants of practice: a systematic review and synthesis of frameworks and taxonomies of factors that prevent or enable improvements in healthcare professional practice. *Implement Sci.* 2013;8:35. 23522377 10.1186/1748-5908-8-35 3617095

63. Wensing M, Oxman A, Baker R, et al.: Tailored Implementation For Chronic Diseases (TICD): a project protocol. *Implement Sci.* 2011;6:103. 21899753 10.1186/1748-5908-6-103 3179734

64. Wensing M, Huntink E, van Lieshout J, et al.: Tailored Implementation of Evidence-Based Practice for Patients with Chronic Diseases. *PLoS One.* 2014;9(7):e101981. 25003371 10.1371/journal.pone.0101981 4087017

65. SAMHSA. AUDIT-C. https://www.integration.samhsa.gov/images/res/tool_auditc.pdf

66. Babor TF, Higgins-Biddle JC, Saunders JB, Monteiro MG AUDIT: The Alcohol Use Disorders Identification Test Guidelines for Use in Primary Care (second edition). https://www.who.int/substance_abuse/publications/audit/en/

67. Center for Quality Assessment and Improvement in Mental Health Patient Health Questionnaire PHQ-2. http://www.cqaimh.org/pdf/tool_phq2.pdf

68. US Preventive Service task Force. Patient Health Questionnaire PHQ-9. https://www.uspreventiveservicestaskforce.org/Home/GetFileByID/218

69. Anderson P, Clement S: The AAPPQ revisited: the measurement of general practitioners' attitudes to alcohol problems. *Br J Addict.* 1987;82(7):753–9. 3478065 10.1111/j.1360-0443.1987.tb01542.x

For peer review only - http://bmjopen.bmj.com/site/about/guidelines.xhtml

70. Schaufeli WB, Shimazu A, Hakanen J, Salanova M, De Witte H. An Ultra-Short Measure for Work Engagement: The UWES-3 Validation Across Five Countries. European Journal of Psychological Assessment 2017

71. Rosenberg G, Bauld L, Hooper L et al. New national alcohol guidelines in the UK: public awareness, understanding and behavioural intentions. *Journal of Public Health*, 2018 40, 549–556,

72. Chung A, Rimal RN: Social norms: A review. *Review of Communication Research.* 2016;4:1–29. 10.12840/issn.2255-4165.2016.04.01.008

73. Sørensen K, Van den Broucke S, Pelikan JM, et al.: Measuring health literacy in populations: illuminating the design and development process of the European Health Literacy Survey Questionnaire (HLS-EU-Q). *BMC Public Health.* 2013;13:948. 24112855 10.1186/1471-2458-13-948 4016258

74. Kaner EF, Dickinson HO, Beyer FR, Campbell F, Schlesinger C, Heather N, Saunders JB, Burnand B, Pienaar ED. Effectiveness of brief alcohol interventions in primary care populations. *Cochrane Database of Systematic Reviews* 2007, Issue 2. Art. No.: CD004148. DOI: 10.1002/14651858.CD004148.pub3.

75. Svoronos T, Mate KS: Evaluating large-scale health programmes at a district level in resource-limited countries. *Bull World Health Organ.* 2011;89(11):831–837. 22084529 10.2471/BLT.11.088138 3209726

76. Derges, J., Kidger, J., Fox, F., Campbell, R., Kaner, E., & Hickman, M. (2017). Alcohol screening and brief interventions for adults and young people in health and community-based settings: A qualitative systematic literature review. *BMC Public Health*, *17*(1), 1–12. https://doi.org/10.1186/s12889-017-4476-4

77. Johnson, M., Jackson, R., Guillaume, L., Meier, P., & Goyder, E. (2011). Barriers and facilitators to implementing screening and brief intervention for alcohol misuse: A systematic review of qualitative evidence. *Journal of Public Health*, *33*(3), 412–421. https://doi.org/10.1093/pubmed/fdq095

78. O'Donnell, A., Wallace, P., & Kaner, E. (2014). From efficacy to effectiveness and beyond: What next for brief interventions in primary care? *Frontiers in Psychiatry*, *5*(AUG), 1–8. https://doi.org/10.3389/fpsyt.2014.00113

79. Moore GF, Audrey S, Barker M, et al.: Process evaluation of complex interventions: Medical Research Council guidance. *BMJ.* 2015;350:h1258. 25791983 10.1136/bmj.h1258 4366184

80. Glasgow RE, Vogt TM, Boles SM: Evaluating the public health impact of health promotion interventions: the RE-AIM framework. *Am J Public Health.* 1999;89(9):1322–7. 10474547 10.2105/AJPH.89.9.1322 1508772

81. Glasgow RE, Klesges LM, Dzewaltowski DA, et al.: Evaluating the impact of health promotion programs: using the RE-AIM framework to form summary measures for decision making involving complex issues. *Health Educ Res.* 2006;21(5):688–694. 16945984 10.1093/her/cyl081

82. Harden SM, Gaglio B, Shoup JA, et al.: Fidelity to and comparative results across behavioral interventions evaluated through the RE-AIM framework: a systematic review. *Syst Rev.* 2015;4:155. 26547687 10.1186/s13643-015-0141-0 4637141

For peer review only - http://bmjopen.bmj.com/site/about/guidelines.xhtml

83. Ysa T, Colom J, Albareda A, et al.: Governance of Addictions. European Public Policies. Oxford University Press,2014. Reference Source

84. OECD: Compendium of OECD well-being indicators. OECD Better Life Initiative.2011; (Accessed 18 December 2016). Reference Source

85. Bertelsmann Stiftung. [website].2016; (Accessed 18 December 2016). Reference Source

86. Inglehart R, Welzel C: Modernization, Cultural Change and Democracy. New York: Cambridge University Press,2005. Reference Source

87. PASS[16] sample size software. https://www.ncss.com/software/pass/: Donner, A. and Klar, N. 2000. Design and Analysis of Cluster Randomization Trials in Health Research. Arnold. London.

88. Hutchings, D., et al., *Implementing screening and brief alcohol interventions in primary care: views from both sides of the consultation.* Primary Health Care Research and Development, 2006. **7**: p. 221-229.

89. Tam, C.W.M., et al., *Alcohol enquiry by GPs - Understanding patients' perspectives: A qualitative study.* Australian family physician, 2015. **44**(11): p. 833-838.

90. Lock, C.A., *Alcohol and brief intervention in primary health care: what do patients think?* Primary Health Care Research and Development, 2006. **5**(2): p. 162-178.

91. Khadjesari, Z., et al., *'I'm not a real boozer': a qualitative study of primary care patients' views on drinking and its consequences.* Journal of public health (Oxford, England), 2018: p. fdy067-fdy067.

92. O'Donnell, A., et al., *Beliefs and attitudes about addressing alcohol consumption in health care: a population survey in England.* BMC Public Health, 2018. **18**(1): p. 391.

93. Miller, P.M., S.E. Thomas, and R. Mallin, *PATIENT ATTITUDES TOWARDS SELF-REPORT AND BIOMARKER ALCOHOL SCREENING BY PRIMARY CARE PHYSICIANS.* Alcohol and Alcoholism, 2006. **41**(3): p. 306-310.

94. Nilsen, P., et al., *Brief interventions in routine health care: a population-based study of conversations about alcohol in Sweden.* Addiction, 2011. **106**: p. 1748 - 1756.

95. Aalto, M., P. Pekuri, and K. Seppä, *Primary health care professionals' activity in intervening in patients' alcohol drinking: a patient perspective.* Drug and Alcohol Dependence, 2002. **66**(1): p. 39-43.

96. O'Donnell, A., et al., *Patients' experiences of alcohol screening and advice in primary care: A qualitative study.* BMC Family Practice, in press. **DOI: 10.1186/s12875-020-01142-9**.

97. Dietz WH, Solomon LS, Pronk N, et al.: An Integrated Framework For The Prevention And Treatment Of Obesity And Its Related Chronic Diseases. *Health Aff (Millwood).* 2015;34(9):1456–1463. 26355046 10.1377/hlthaff.2015.0371

98. Palumbo R: Contextualizing co-production of health care: a systematic literature review. *Int J Public Se Manage.* 2016;29(1):72–90. 10.1108/IJPSM-07-2015-0125

99. Rehm J, Room R, Monteiro M, et al.: Alcohol Use. In: *Comparative quantification of health risks: global and regional burden of disease attributable to selected major risk factors*. Ezzati M *et al.* (eds),

For peer review only - http://bmjopen.bmj.com/site/about/guidelines.xhtml

Geneva, Switzerland: World Health Organization.2004;959–1109. Reference Source

100. European Medicines Agency: Guideline on the development of medicinal products for the treatment of alcohol dependence.2010. Reference Source

101. Rehm J, Lachenmeier DW, Room R: Why does society accept a higher risk for alcohol than for other voluntary or involuntary risks? *BMC Med.* 2014;12:189. 25424648 10.1186/s12916-014-0189-z 4203927

102. Cunningham JA: Unintended impact of using different inclusion cut-offs for males and females in intervention trials for hazardous drinking. *Addiction.* 2017. 28168847 10.1111/add.13760

103. Aldridge A, Dowd W, Bray J: The relative impact of brief treatment versus brief intervention in primary health-care screening programs for substance use disorders. *Addiction.* 2017;112(Suppl 2):54–64. 28074568 10.1111/add.13653

104. Barbosa C, Cowell A, Dowd W, et al.: The cost-effectiveness of brief intervention versus brief treatment of screening, brief intervention, and referral to treatment (SBIRT) in the United States. *Addiction.* 2017;112(Suppl 2):73–81. 28074567 10.1111/add.13658

105. Anderson P, Kaner E, Keurhorst M, et al.: Attitudes and Learning through Practice Are Key to Delivering Brief Interventions for Heavy Drinking in Primary Health Care: Analyses from the ODHIN Five Country Cluster Randomized Factorial Trial. *Int J Environ Res Public Health.* 2017;14(2): pii: E121. 28134783 10.3390/ijerph14020121 5334675

106. Funk M, Wutzke S, Kaner E, et al.: A multicountry comparator led trial of strategies to promote dissemination and implementation of brief alcohol intervention in primary health care: findings of a World Health Organization collaborative study. *J Stud Alcohol.* 2005;66(3):379–388. 16047527 10.15288/jsa.2005.66.379

107. Reeves BC, Wells GA, Waddington H: Quasi-experimental study designs series-paper 5: classifying studies evaluating effects of health interventions-a taxonomy without labels. *J Clin Epidemiol.* 2017; pii: S0895-4356(17)30288-3. 28351692 10.1016/j.jclinepi.2017.02.016

108. Jantsch E: Inter- and Transdisciplinary University: A systems approach to education and innovation. *Policy Sciences.* 1970;1(1):403–428. 10.1007/BF00145222

109. Pohl C, Hirsch Hadorn G: Principles for Designing Transdisciplinary Research. proposed by the Swiss Academies of Arts and Sciences. München: oekom Verlag.2007. Reference Source

For peer review only

For peer review only - http://bmjopen.bmj.com/site/about/guidelines.xhtml

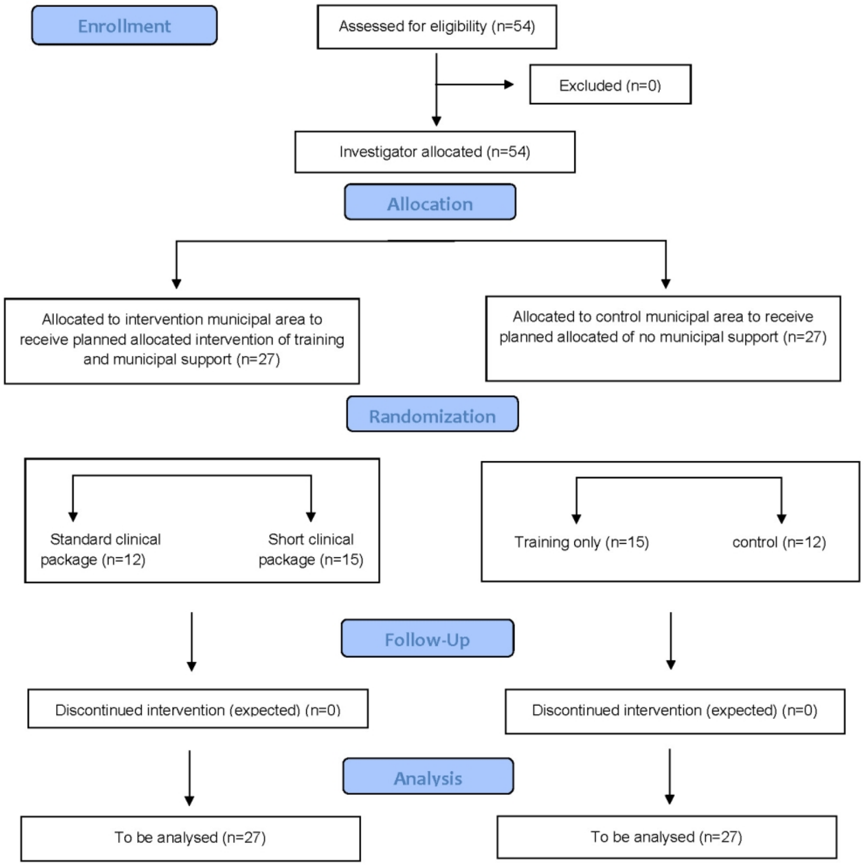

Figure 1 Study flow diagram

215x279mm (200 x 200 DPI)

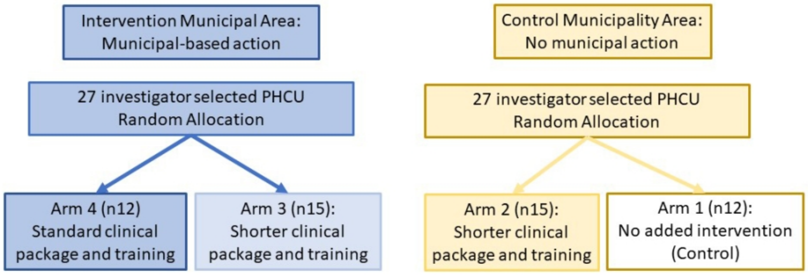

Figure 2. Study design for the first six months of the 18-month implementation period

338x190mm (96 x 96 DPI)

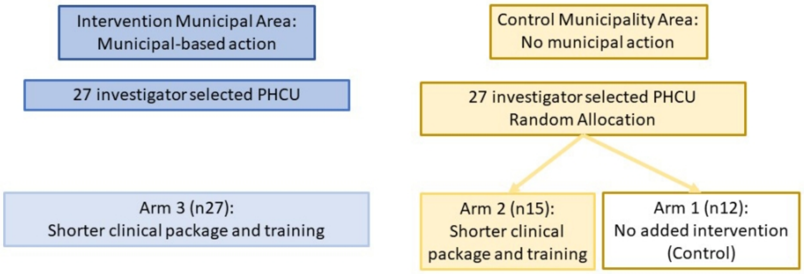

Figure 3. Study design from month 8 onwards, assuming no superiority of Arm 4 over Arm 3 during first six months of implementation.

338x190mm (96 x 96 DPI)

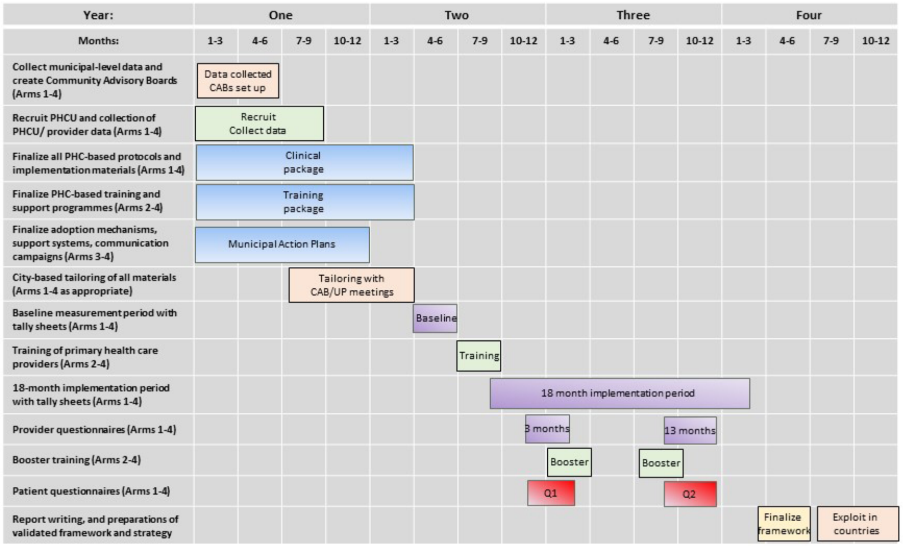

Figure 4. Study timetable.

254x190mm (96 x 96 DPI)

Supplement Box 1 Deviations from pre-grant submission pre-protocol

Moving from two-arm to four-arm design In the pre-submission pre-protocol for the quasi-experimental study [1], within each country, two municipal jurisdictions were to be investigator-selected, each with nine primary health care units (PHCU) as part of the study. In one municipal jurisdiction, the intervention municipality, the PHCU would receive both training and municipal support; in the other municipal jurisdiction, the comparator municipality, PHCU would continue practice as usual, with no training or municipal support. The hypothesis was that PHCU in the intervention municipality would measure the alcohol consumption of more patients and give advice to more heavy drinking patients than the PHCU in the comparator municipality.

In the final protocol, within each country, the nine PHCU in the comparator municipality are randomly allocated to five PHCU receiving training (new Arm 2) and four PHCU continuing practice as usual (new Arm 1). The rationale for this approach is that it will enable us to test the independent impact of municipal support over and above just training. The hypothesis to be tested is that PHCU that receive both training and municipal support in the intervention municipality will measure the alcohol consumption of more patients and give advice to more heavy drinking patients than the PHCU who just receive training (Arm 2).

In addition, in the final protocol, within each country, the nine PHCU in the intervention municipality are randomly allocated to four PHCU receiving a standard and longer clinical package and training (new Arm 4) and five PHCU receiving a shorter clinical package and training (new Arm 3), both new Arms 3 and 4 receiving municipal support. The hypothesis to be tested is that the PHCU that receive the standard and longer clinical package and training that is commonly implemented (new Arm 4) will not measure the alcohol consumption of more patients and not give advice to more heavy drinking patients than the PHCU that receive a shorter clinical package and training (new Arm 3). This will be tested over the first six months of the 18-month implementation period, and, if there is non-superiority of Arm 4 over Arm 3, Arm 4 will be collapsed into Arm 3 from month 8 onwards.

Cross-sectional patient self-complete questionnaire instead of prospective interview The deviation is to move from patient follow-up interviews to cross-sectional patient self-completed questionnaires. In the pre-submission pre-protocol, during month 3 of the 18-month implementation period, the first six consecutive screen-negative patients and the first six consecutive screen-positive patients identified by each PHCU were to be invited by the health care provider to give their written consent to complete two follow-up questionnaires, at six months and twelve months after the initial screening. In the final protocol, at two time points, during the 18-month implementation period (months 3 and 15), on two separate days in each of month 3 and 15, providers will seek consent from the patient to self-complete additional questions in the waiting room before leaving the PHCU, handing the completed questions to a researcher in attendance. The rationale for the change is that, primarily due to the nature of the catchments area of patients, it became apparent that it would be impossible to achieve sufficient follow-up rates required for valid analysis of data, with much too high a proportion of country-based resources used in order to try to achieve adequate follow-up rates.

Adjustment in primary outcome indicator The deviation is to change the denominator for the main outcome variable from number of consulting adult patients in a given time period (e.g., one month) to number of registered adult patients. In the pre-submission pre-protocol, the primary outcome was to be the proportion of consulting adult patients (aged 18+ years) intervened (alcohol consumption measured and advice given to heavy drinkers), calculated as the number of AUDIT-C positive patients that received oral advice or referral for advice to another provider in or outside the PHCU, divided by the total number of adult consultations of the participating providers per PHCU. In the final protocol, the primary outcome will be the cumulative proportion of the number of adults (aged 18+ years) registered with the PHCU that have their alcohol consumption measured with AUDIT-C. The rationale is that the revised primary outcome is a measure of coverage, which is considered more intuitive and relevant for health systems change (similar to blood pressure - the proportion of patients that have had their blood pressure measured).

Recalculation of statistical power The change in the main outcome measure required a re-calculation of the statistical power. The study remains adequately powered.

For peer review only - http://bmjopen.bmj.com/site/about/guidelines.xhtml

Supplement Table 1 Clinical Package and Training by Study Arm

	Standard package and training (Arm 4)	Shorter package and training (Arms 2 and 3)	Control (Arm 1)
**Instruments**	Short tally sheet: AUDIT-C [2] completed; if AUDIT-C ≥8, AUDIT-10 [3] and PHQ2 [4] completed; if PHQ2 ≥3, PHQ9 [5] completed.	Very short tally sheet: AUDIT-C completed; if AUDIT-C ≥8, PHQ2 completed.	Very short tally sheet: AUDIT-C completed; if AUDIT-C ≥8, PHQ2 completed.
**Provider material**	Provider booklet on alcohol and depression: 43 pages plus 12-page 'quick guide'.	Provider booklet on alcohol and depression: 16 pages.	Provider booklet on alcohol and depression: 11 pages.
**Patient advice and material for alcohol**	Alcohol advice: 5-minute 10-step plan plus 10-page patient brief advice booklet.	Alcohol advice: 1-minute simple advice that the patient needs to drink less, plus 1-page patient brief advice leaflet.	Alcohol advice: 1-minute simple advice that the patient needs to drink less and provide a brief advice leaflet (if available).
	Patient alcohol leaflet: 1 page folded in half to give 4 sides.	Patient alcohol leaflet: 1 page folded in half to give 4 sides.	SCALA patient leaflet on alcohol not given. Provider booklet advises "If available, provide a leaflet on self-management of heavy drinking."
**Patient advice and material for depression**	PHQ9 score 10-14, provide patient leaflet on depression; PHQ 9 ≥14, use clinical judgement to consider if referral is required - if not provide patient leaflet on depression.	PHQ2 ≥3, patient leaflet on depression given.	SCALA patient leaflet on depression not given. Provider booklet advises "If available, provide a leaflet on self-management of depression and action to take if symptoms persist or worsen."
	Patient depression advice leaflet: 1 page, 3 columns.	Patient depression advice leaflet: 1 page, 3 columns.	Present practice.
**Referral**	Referral for very heavy drinking, depression, suicide risk: existing clinical judgement and practice.	Referral for very heavy drinking, depression, suicide risk: existing clinical judgement and practice.	Referral for very heavy drinking, depression, suicide risk: existing clinical judgement and practice.
**Training**	Training: two times two-hours training plus two times one-hour booster sessions (six hours total).<br>Training will take place within	Training: one two-hours training in PHCU, plus one-hour booster session (three hours total).<br>Training will focus on	Present practice.

For peer review only - http://bmjopen.bmj.com/site/about/guidelines.xhtml

	the PHCU or clusters of PHCUs. Training will focus on practical skills in undertaking measurement and assessment, and in delivering brief advice, in using the questionnaires, and in knowing when and how to refer patients with more severe heavy drinking and moderately severe or severe depression to available services, such as community-based mental health and addiction centres. Training will, in addition, address attitudes, and perceived barriers and facilitators in implementing measurement and brief advice, contextualized to local circumstances.	practical skills in undertaking measurement and assessment, and in delivering brief advice for harmful alcohol use; instruction of 'care-as-usual' + leaflet for depression and severe cases requiring referral. Training will, in addition, address attitudes, and perceived barriers and facilitators in implementing measurement and brief advice, contextualized to local circumstances.	
	Training for both the standard and shorter packages will be undertaken by members of the research team, accredited teachers, or addiction consultants, who will receive a full two-day train-the-trainers session from a senior addiction specialist trainer. The training formats employed are didactic input, guided discussions, skills and practice modeled through videos and role plays. Training sessions are developed from [6-7].		

For peer review only - http://bmjopen.bmj.com/site/about/guidelines.xhtml

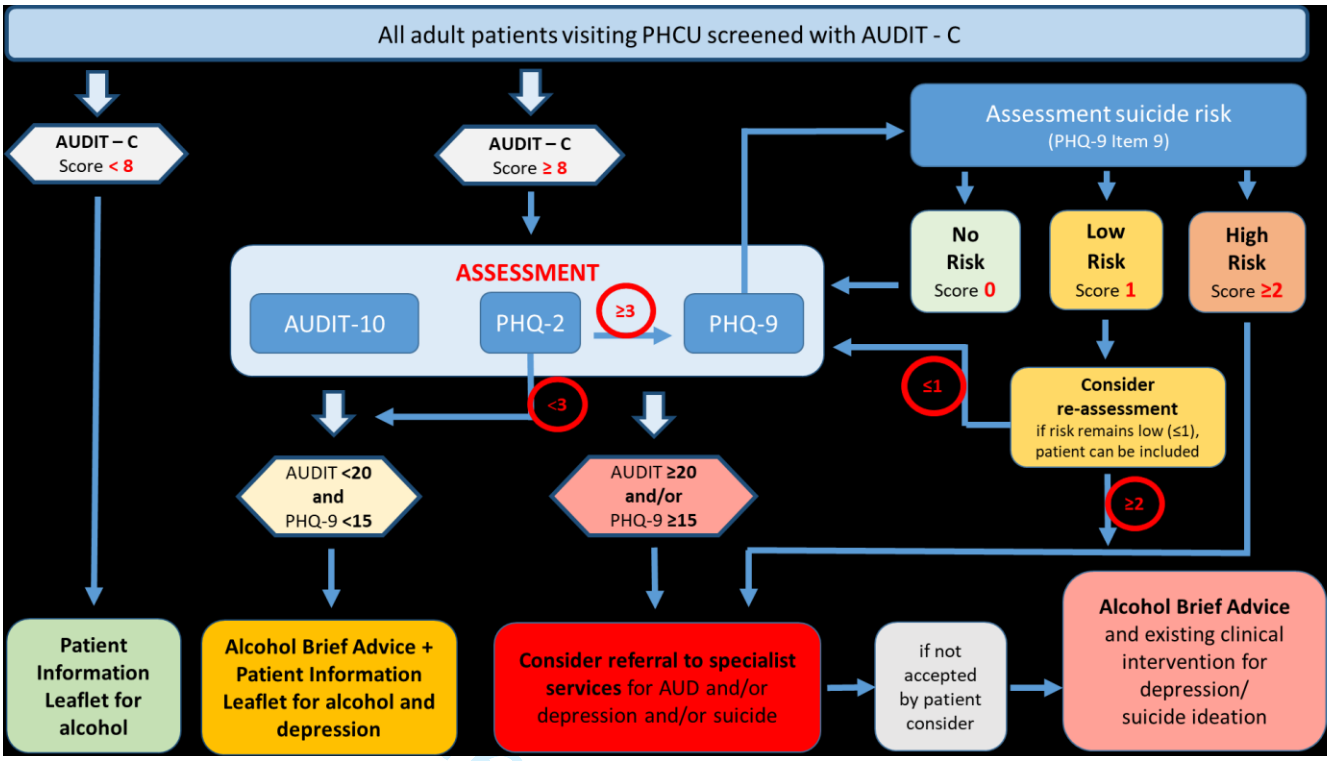

Supplement Figure 1. Standard Care Pathway for Arm 4

For peer review only

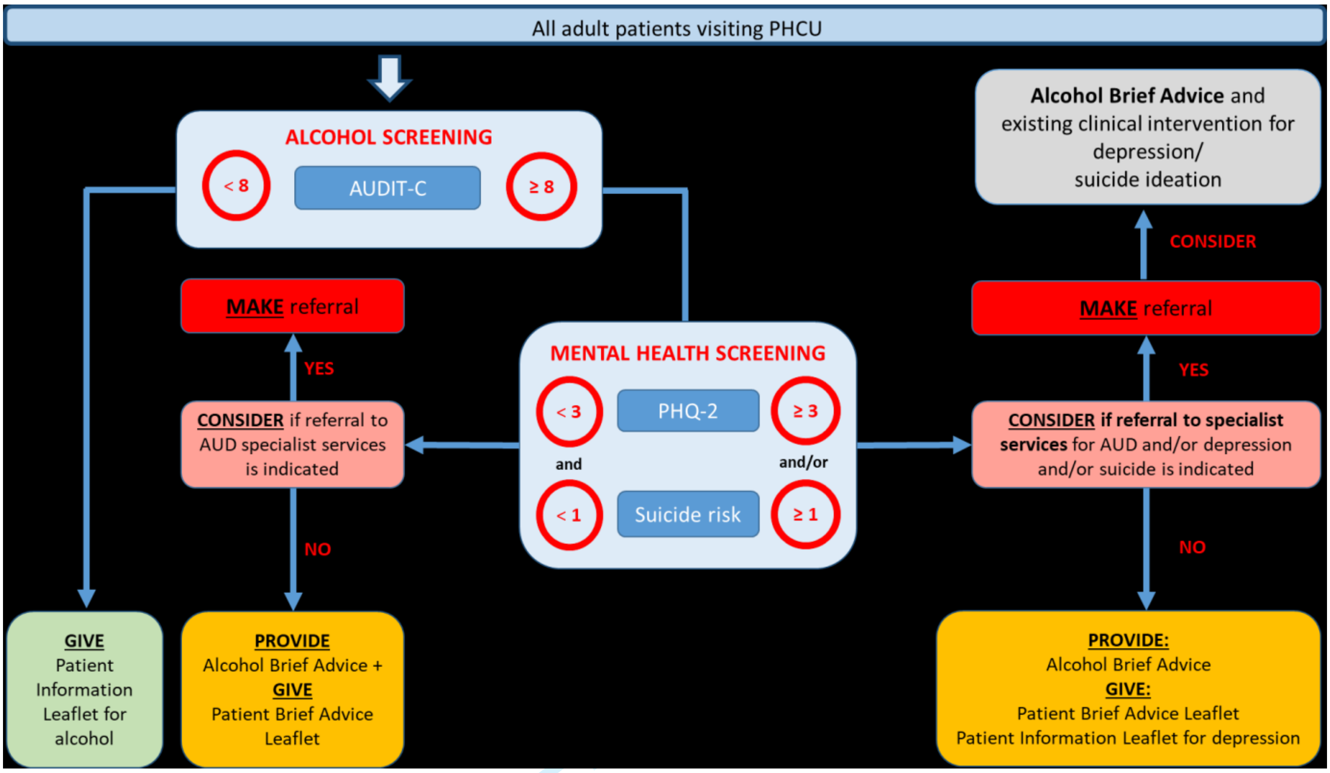

Supplement Figure 2. Short Care Pathway for Arms 1, 2, and 3

For peer review only - http://bmjopen.bmj.com/site/about/guidelines.xhtml

Supplement Table 2 Municipal Integration and Support by Study Arm

Intervention Municipal Area (Arms 3 and 4)	Comparator Municipal Area (Arms 1 and 2)
Community Advisory Board (CAB) of local stakeholders set up (including representatives of municipal area, PHCU, health services, non-governmental organizations, academia, media).	Present practice.
User Panel (UP) of local providers and patients set up.	Present practice.
CAB and UP review and tailor relevant materials of clinical package and training courses within the seven domains of: local and national guideline factors; individual health care provider factors; patient factors; interactions between different professional groups; incentives and resources; capacity for organizational change; and, social, political and legal factors [8-10].	Present practice.
CAB reviews barriers and facilitators and potential drivers of successful action [11-12].	Present practice.
CAB identifies potential adoption mechanisms and support systems [13], and reviews plans and components of community-based communication and media campaigns [14-16].	Present practice.
Integrator (champion and knowledge and practice broker) to serve as trusted and accountable leader [13]: facilitating agreement within the municipal area and health systems on shared goals and metrics; assessing and acting on relevant community resources; working at the systems level to make relevant practice changes for sustainability; gathering, analysing, monitoring, integrating, learning, and sharing data at the individual PHCU and city levels; identifying and connecting with system navigators who help PHCUs coordinate, access, and manage multiple services and supports; and developing a system of ongoing and intentional communication with PHCUs and cities.	Present practice.
Adoption mechanisms implemented [13], including: (i) demonstration of the superiority of the PHC package, its simplicity, and its alignment with the latest evidence of preventing and managing heavy drinking and of implementation science; (ii) engagement of identified leaders and building their capacity to lead and ensure broad adoption of the PHC package through guiding and supporting large-scale change; (iii) communicating the value of the PHC package to both municipal and PHC frontline staff; (iv) identifying and adjusting, as appropriate and possible, relevant policies at PHC and city levels to expedite the adoption of the PHC package, for example by adapting electronic health records; and, (v) identifying gaps in health system performance and the urgent need to prevent and manage heavy drinking to promote the needed will and energy to bring implementation of the PHC package to scale.	Present practice.
Support mechanisms implemented [13], including: (i) development of professional capacity for scale-up; (ii) development of infrastructure for scale-up, achieved through redesign rather than addition of new resources; (iii) linking to monitoring and evaluation, using reliable data collection and reporting systems that track and provide feedback on the performance of key processes and outcomes, for example monthly reporting on measurement and brief advice activity; (iv) setting up learning systems to capture change ideas that are shown to result in improved performance assembling ideas into a change package. Knowledge should be shared between municipal actors and PHCUs through regular electronic newsletters and communications; and, (v) creating design factors that enhance sustainability including high reliability of the new processes, inspection systems	Present practice.

For peer review only - http://bmjopen.bmj.com/site/about/guidelines.xhtml

to ensure desired results are being achieved, support for structural elements, and ongoing learning systems.	
Communication and media campaign implemented [14-16], including (i) posters, leaflets and/or brochures placed at visible spots in the intervention municipality, e.g., in waiting rooms of PHCUs, health departments, banks, markets; (ii) regular communications, including emails and WhatsApp messages) sent to the healthcare providers and other involved stakeholders in the intervention municipality, (iii) media presence through e.g. articles in local newspapers; interviews, reportages, promotion spots and/or media appearances on local radio, local TV and other local media, and (iv) workshops, forums and/or public local meetings for interested stakeholders such as healthcare providers, representatives of municipal health institutions and patients. All abovementioned activities will focus on reframing that it is heavy drinking that is the problem and that this can be helped to be reduced through primary health care-based measurement and advice programmes, addressing topics such as the harm of hazardous alcohol use in the general population, the (cost)effectiveness and importance of brief alcohol interventions and SCALA success stories.	Present practice.

For peer review only - http://bmjopen.bmj.com/site/about/guidelines.xhtml

Supplement Table 3 Data collected at municipal level (if not available, at city, regional or country level)

-	Geographical location in city;
-	Demographic size of municipal area;
-	Indicators of deprivation;
-	Information on prevalence of alcohol consumption and related harm;
-	Information on prevalence of depression;
-	Description of current action to reduce alcohol-related harm;
-	Jurisdictional responsibilities for health-related prevention and treatment;
-	Structural relationships with primary health care services;
-	Structural relationships with hospital-based services;
-	Available data mapped to OECD better life initiative [17], including material living conditions (housing, income and jobs) and quality of life (community, education, environment, governance, health, life satisfaction, safety and work-life balance);
-	Sustainable Governance Indicators [18], including the Status Index, which 'examines each state's reform needs in terms of the quality of democracy and performance in key policy fields', and the Management Index, focused on 'governance capacities in terms of steering capability and accountability'; and,
-	World Values Survey data [19] for cross-cultural variation (Traditional vs. Secular-rational; and, Survival vs. Self-expression).

For peer review only - http://bmjopen.bmj.com/site/about/guidelines.xhtml

Supplement Table 4 Overview of the measures used in the provider questionnaire

Measure used	Constructs measured
Shortened Alcohol and Alcohol Problems Perception questionnaire [20]	Role security, therapeutic commitment
Abbreviated Maslach Burnout Inventory [21]	Emotional exhaustion, depersonalization, personal accomplishment
Utrecht Work Engagement Scale [22]	Work engagement
Alcohol knowledge [23]	Awareness of drinking guidelines, social norms regarding drinking
Perceived barriers questionnaire [24]	Perceived barriers
Opinion on screening (based on [25])	Pros and cons of screening, social norms of screening, intention to screen
Self-efficacy in delivering the SCALA protocol (based on [26])	Self-efficacy
Context assessment for community health (COACH) tool [27]	Resources, Community engagement, Monitoring services for action, Work culture, Leadership
Evaluation of SCALA community action [15]	Exposure to campaign/adoption mechanisms/support systems, perceptions of campaign/adoption mechanisms/support systems
Attributes of innovation questionnaire [28]<br>- Only intervention group	Relative advantage, Compatibility, Complexity, Trialability and Observability
Experienced barriers (based on the driver diagram [12])<br>- Only intervention group	Experienced barriers

For peer review only - http://bmjopen.bmj.com/site/about/guidelines.xhtml

Supplement Table 5. Country-level collection of economic data for return-of-investment analyses

Costs of Investment		Gains of investment	
Cost unit	***Data source***	***Cost unit***	***Data Source***
Cost of providing training and booster sessions to PHCU staff	Time and materials required, documented by study team	Costs and utilization of *primary health care* (number of visits) by major disease/injury categories	National statistics, ministry of health, local researchers, or other publications
Setting up and maintaining Community Advisory Boards and User Panels	Time and materials required, documented by study team	Costs and utilization of *emergency* facilities (number of admissions) by major disease/injury categories	National statistics, ministry of health, local researchers, or other publications
Direct costs for implementing the clinical pathway (routine measurement, further assessment, brief interventions, referral)	Staff salary and time required, documented by PHCU administration and providers	Costs and utilization of *inpatient* facilities (number of admissions, length of stay) and of *outpatient* facilities (number of admissions) by major disease/injury categories	National statistics, ministry of health, local researchers, or other publications
Additional costs for implementing the clinical pathway	Documented by PHCU administration	Avoided mortality	National statistics, ministry of health, local researchers, or other publications

For peer review only

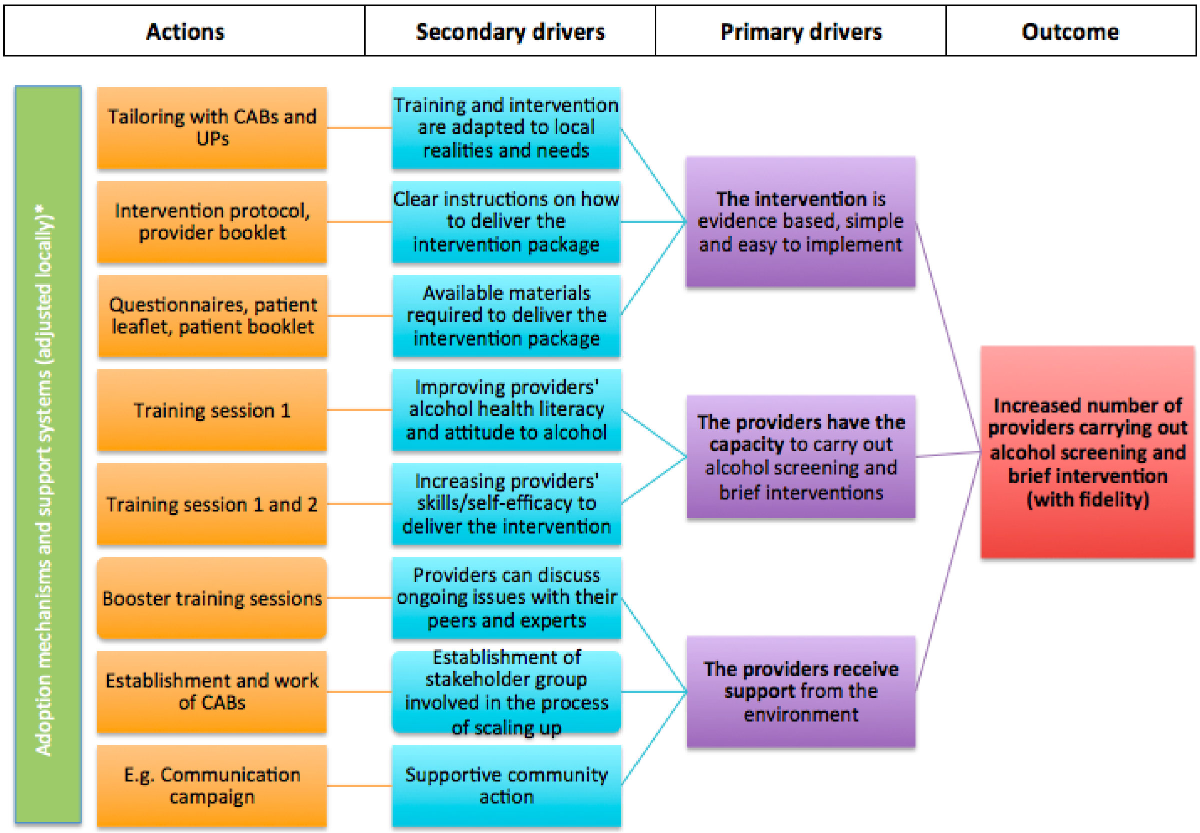

Supplement Figure 3. Driver diagram of the SCALA protocol

review only

Supplement Table 6 Process evaluation topics based on MRC framework [29]

Part of process evaluation		Topic of investigation	Method
**Description of the intervention**		The description of the intervention and its causal assumptions	Driver diagram
**Implementation**	*Adaptation*	Experience of intervention tailoring	Key informant interview
		Experience with training tailoring	Key informant interview
	Dose delivered (completeness of delivery)	Implementation of the protocol (number of measurements, brief advice given, referrals done)	Tally sheets
		Length of implemented training	Observation
		Implementation of adoption mechanisms and support systems on municipal and organisational level	Key informant interview, Document analysis
		Implementation of CAB meetings	Observation, document analysis
		Implementation of communication campaign	Key informant interview, document analysis
	Fidelity (quality of implementation)	Following the care pathway as intended	Tally sheets, patient questionnaire
		Training active ingredient delivery	Observation
	Reach	Number of patients and providers involved	Document analysis
		Number of providers attending the training	Document analysis
**Mechanisms of impact**	*Participant responses*	Patients' perception of acceptability of intervention	Patient questionnaire
		Providers' satisfaction with the training	Post-training questionnaire
		Providers' perceived utility of training sessions	Post-training questionnaire
		Perception of the intervention	Key informant interview
		Perception of the campaign	Provider questionnaire, patient questionnaire
		Perception of the municipal action	Key stakeholder interview
	Mediators	Influence of training on attitude and self-efficacy	Provider questionnaire
		Influence of communication campaign on beliefs and social norms	Provider questionnaire
		Perception of the attributes of the intervention	Provider questionnaire
	Unintended consequences	Possible unexpected side effects emerging	Key stakeholder interview
**Context**		Perceptions of organisational context	Provider questionnaire
		Individual moderating characteristics	Provider questionnaire
		Description of organisational context changes	Key informant interview, logbook
		Contextual factors influencing training	Observation, key informant interview
		Contextual factors influencing municipal action	Key informant interview, document analysis
**Outcomes**		Integration of process evaluation information with the results of the outcome evaluation	Integration of data collected through abovementioned methods with the tally sheet data

For peer review only

For peer review only - http://bmjopen.bmj.com/site/about/guidelines.xhtml

Supplement Table 7 Completed seven-point checklist for SCALA study design [30]

Quality Measure	SCALA
1.Was the intervention/(answer "yes" to more than 1 item, if applicable)	
Allocated to (provided for / administered to / chosen by) individuals?	No
Allocated to (provided for / administered to / chosen by) clusters of individuals?	No
Clustered in the way it was provided (by practitioner or organisational unit)?	YES
**2. Were outcome data available: *(answer "yes" to only 1 item)***	
After intervention / comparator only (same individuals)?	-
After intervention / comparator only (not all same individuals)?	-
Before (once) AND after intervention / comparator (same individuals)?	YES
Before (once) AND after intervention / comparator (not all same individuals)?	-
Multiple times before AND multiple times after intervention / comparator(same individuals)?	-
Multiple times before AND multiple times after intervention / comparator (not all same individuals)?	-
**3. Was the intervention effect estimated by: *(answer "yes" to only 1 item)***	
CHANGE OVER TIME (same individuals at different time points)?	-
CHANGE OVER TIME (not all same individuals at different time points)?	-
DIFFERENCE BETWEEN GROUPS (of individuals or clusters receiving either intervention or comparator)?	YES
**4. Did the researchers aim to control for confounding (design or analysis) *(answer "yes" to only 1 item)*:**	
Using methods that control in principle for any confounding?	-
Using methods that control in principle for time invariant unobserved confounding?	-
Using methods that control only for confounding by observed covariates?	YES
**5. Were groups of individuals or clusters formed by *(answer "yes" to more than 1 item, if applicable)*:**	
· Randomization?	No
· Quasi-randomization?<br>· Explicit rule for allocation based on a threshold for a variable measured on a continuous or ordinal scale or boundary (in conjunction with identifying the variable dimension, below)?	No
· Some other action of researchers?	YES
· Time differences?	No
· Location differences?	YES
· Healthcare decision makers / practitioners?	No
· Participants' preferences?	No
· Policy maker	No
· On the basis of outcome?	No
· Some other process? (specify)	No
**6. Were the following features of the study carried out after the study was designed *(answer "yes" item, if applicable): to more than 1***	
Characterization of individuals / clusters before intervention?	YES
Actions/choices leading to an individual/cluster becoming a member of a group?	YES
Assessment of outcomes?	YES

For peer review only - http://bmjopen.bmj.com/site/about/guidelines.xhtml

**7. Were the following variables measured before intervention:** ***(answer "yes" to more than 1 item,*** **If applicable)**	
Potential confounders?	YES
Outcome variable(s)?	YES

For peer review only

References

1. Shadish WR, Cook TD, Campbell DT: Experimental and quasi-experimental designs for generalized causal inference. 2nd edition. Houghton Mifflin, Michigan,2002. Reference Source

2. SAMHSA. AUDIT-C. https://www.integration.samhsa.gov/images/res/tool_auditc.pdf

3. Babor TF, Higgins-Biddle JC, Saunders JB, Monteiro MG AUDIT: The Alcohol Use Disorders Identification Test Guidelines for Use in Primary Care (second edition). https://www.who.int/substance_abuse/publications/audit/en/

4. US Preventive Service task Force. Patient Health Questionnaire PHQ-9. https://www.uspreventiveservicestaskforce.org/Home/GetFileByID/218

5. Center for Quality Assessment and Improvement in Mental Health Patient Health Questionnaire PHQ-2. http://www.cqaimh.org/pdf/tool_phq2.pdf

6. Gual A, Anderson P, Segura L, et al.: Alcohol and Primary health care: Training programme on identification and brief interventions. Barcelona: department of Health of the Government of Catalonia,2005. Reference Source

7. Anderson P, Bendsten P, Spak F, et al.: Improving the delivery of brief interventions for heavy drinking in primary health care: outcome results of the Optimizing Delivery of Health Care Intervention (ODHIN) five-country cluster randomized factorial trial. *Addiction.* 2016;111(11):1935–1945. 27237081 10.1111/add.13476

8. Wensing M, Oxman A, Baker R, et al.: Tailored Implementation For Chronic Diseases (TICD): a project protocol. *Implement Sci.* 2011;6:103. 21899753 10.1186/1748-5908-6-103 3179734

9. Wensing M, Huntink E, van Lieshout J, et al.: Tailored Implementation of Evidence-Based Practice for Patients with Chronic Diseases. *PLoS One.* 2014;9(7):e101981. 25003371 10.1371/journal.pone.0101981 4087017

10. Flottorp SA, Oxman AD, Krause J, et al.: A checklist for identifying determinants of practice: a systematic review and synthesis of frameworks and taxonomies of factors that prevent or enable improvements in healthcare professional practice. *Implement Sci.* 2013;8:35. 23522377 10.1186/1748-5908-8-35 3617095

11. Prasad A, Kano M, Dagg KA, et al.: Prioritizing action on health inequities in cities: An evaluation of Urban Health Equity Assessment and Response Tool (Urban HEART) in 15 cities from Asia and Africa. *Soc Sci Med.* 2015;145(2015):237–242. 26456133 10.1016/j.socscimed.2015.09.031

12. Svoronos T, Mate KS: Evaluating large-scale health programmes at a district level in resource-limited countries. *Bull World Health Organ.* 2011;89(11):831–837. 22084529 10.2471/BLT.11.088138 3209726

13. Barker PM, Reid A, Schall MW: A framework for scaling up health interventions: lessons from large-scale improvement initiatives in Africa. *Implement Sci.* 2016;11(1):12. 26821910 10.1186/s13012-016-0374-x 4731989

14. Heather N: WHO Collaborative Project on Identification and Management of Alcohol-related Problems in PHC – Report to the World Health Organisation on Phase IV: Development of Country-wide Strategies for Implementing Early Identification and Brief Intervention in PHC. Geneva: World Health Organisation, Department of Mental Health and Substance Abuse:2006. Reference Source

15. Keller, P. A., & Lehmann, D. R. (2008). Designing effective health communications: a meta-analysis. *Journal of Public Policy & Marketing*, *27*(2), 117-130.

16. Rice, R. E., & Atkin, C. K. (Eds.). (2012). *Public communication campaigns*. Sage.

For peer review only - http://bmjopen.bmj.com/site/about/guidelines.xhtml

17. OECD: Compendium of OECD well-being indicators. OECD Better Life Initiative.2011; (Accessed 18 December 2016). Reference Source

18. Bertelsmann Stiftung. [website].2016; (Accessed 18 December 2016). Reference Source

19. Inglehart R, Welzel C: Modernization, Cultural Change and Democracy. New York: Cambridge University Press,2005. Reference Source

20. Anderson P, Clement S: The AAPPQ revisited: the measurement of general practitioners' attitudes to alcohol problems. *Br J Addict.* 1987;82(7):753–9. 3478065 10.1111/j.1360-0443.1987.tb01542.x

21 Riley MR, Mohr DC, Waddimba AC. The reliability and validity of three-item screening measures for burnout: Evidence from group-employed health care practitioners in upstate New York. Stress and Health. 2018 Feb;34(1):187-93.

22 Schaufeli WB, Shimazu A, Hakanen J, Salanova M, De Witte H. An Ultra-Short Measure for Work Engagement: The UWES-3 Validation Across Five Countries. European Journal of Psychological Assessment 2017

23 Rosenberg G, Bauld L, Hooper L et al. New national alcohol guidelines in the UK: public awareness, understanding and behavioural intentions. *Journal of Public Health*, 2018 40, 549–556,

24 Anderson P, Wojnar M, Jakubczyk A, Gual A, Reynolds J, Segura L, Sovinova H, Csemy L, Kaner E, Newbury-Birch D, Fornasin A. Managing alcohol problems in general practice in Europe: results from the European ODHIN survey of general practitioners. Alcohol and Alcoholism. 2014 Jul 16;49(5):531-9.

25 Francis J, Eccles MP, Johnston M, Walker AE, Grimshaw JM, Foy R, Kaner EF, Smith L, Bonetti D. Constructing questionnaires based on the theory of planned behaviour: A manual for health services researchers.

26 Bandura A. Guide for constructing self-efficacy scales. Self-efficacy beliefs of adolescents. 2006 Feb 1;5(1):307-37.

27 Bergström A, Skeen S, Duc DM, Blandon EZ, Estabrooks C, Gustavsson P, Hoa DT, Källestål C, Målqvist M, Nga NT, Persson LÅ. Health system context and implementation of evidence-based practices—development and validation of the Context Assessment for Community Health (COACH) tool for low-and middle-income settings. Implementation Science. 2015 Dec;10(1):120.

28 Scott SD, Plotnikoff RC, Karunamuni N, Bize R, Rodgers W. Factors influencing the adoption of an innovation: An examination of the uptake of the Canadian Heart Health Kit (HHK). Implementation Science. 2008 Dec;3(1):41.

29. Moore GF, Audrey S, Barker M, et al.: Process evaluation of complex interventions: Medical Research Council guidance. *BMJ.* 2015;350:h1258. 25791983 10.1136/bmj.h1258 4366184

30. Reeves BC, Wells GA, Waddington H: Quasi-experimental study designs series-paper 5: classifying studies evaluating effects of health interventions-a taxonomy without labels. *J Clin Epidemiol.* 2017; pii: S0895-4356(17)30288-3. 28351692 10.1016/j.jclinepi.2017.02.016

For peer review only - http://bmjopen.bmj.com/site/about/guidelines.xhtml

SCALA - DATA MANAGEMENT PLAN

Draft version 1: 23 January, 2018
Draft version 2: 1 February, 2018
Draft version 3: 27 February, 2018
Draft version 4: 2 March, 2018
Draft version 5: 15 March, 2018
Draft version 6: 15 May, 2018
Draft version 7: 23 May, 2018
version 8: 24 May, 2018

Abbreviations and definitions:

DMP	= data management plan
IRB	= Institutional Research Board
PHCCs	= primary health care centres
SCALA	= Scale-up of Prevention and Management of Alcohol Use Disorder in Latin America
Data center	= Technische Universität Dresden, Germany (supervisor: Jürgen Rehm)

Contents

1. Data Summary ... 1
2. FAIR data ... 5
2.1. Making data findable, including provisions for metadata ... 5
2.2. Making data openly accessible ... 5
2.3. Making data interoperable ... 6
2.4. Increase data re-use (through clarifying licences) ... 7
3. Allocation of resources ... 8
4. Data security ... 9
5. Ethical aspects ... 10
6. Other issues ... 11
7. Data analysis plan ... 12
7.1. REACH ... 13
7.2. EFFECTIVENESS ... 14
7.3. ADOPTION ... 14
7.4. IMPLEMENTATION ... 15
7.5. MAINTENANCE ... 15
8. Appendix ... 18

For peer review only

1. Data Summary

Introduction

During the course of the SCALA study, quantitative, qualitative, as well as publicly available data will be collected in PHCCs in three American countries: Mexico, Peru, Colombia. All collected data are required for a thorough evaluation of the main study goal and it corollaries, ie. to improve alcohol management in PHCCs by increasing screening rates and delivery of adequate advice and treatment for screen positives. The following qualitative and quantitative data will be obtained from patients and providers in PHCCs. All data will be transferred first to the data center serving as SCALA data repository at the TU Dresden (for details on data transfer, see ***section 4***). After cleaning the data and bringing it into the standard format (for details, see ***section 2.2***), the data will be forwarded to partners based on the workplan or upon request. While all data will be kept with the data center, they are collectively owned by all partners.

Data origin

Q1) **PHCC structure data (quantitative):**
Collection of data from the participating PHCCs before start of data collection. The PHCC administration will be asked to fill out a form (see '***Q1_PHCC Description Form.pdf***'), including the number of registered patients, as well as number of health professionals working in the centre. The data will be entered into spreadsheets (see '***Q1_PHCC Description Form_spreadsheet template.xlsx***'), which will then be sent to the data center.

Q2) **Short tally sheet for routine care data (quantitative):**
Collection of routine care data on **all** adult patients consulting PHCCs. For this purpose, a tally sheet (see '***Q2_Short Patient Tally Sheet.pdf***') will be applied to collect all necessary information on sociodemographics (sex, age, socioeconomic status) and drinking patterns (AUDIT-C) for all patients. For screen positives, the tally sheet will also capture the results of in-depth assessment of alcohol problems (AUDIT) and depression (PHQ-2 and - if above threshold - PHQ-9) and the decisions made concerning brief advice and treatment and referral to specialist care. The tally sheets will be collected by local researchers on a weekly basis and entered into spreadsheet templates (see '***Q2_Short Patient Tally Sheet_spreadsheet template.xlsx***'). These spreadsheets will be submitted monthly to the data center.

Q3) **Long tally sheet for quality control data (quantitative):**
Collection by respective PHCC of a more extensive set of routine care data for quality control on **a subset** of adult patients consulting PHCCs. Quality control data will only be collected during predefined periods during the 18 months implementation period, resulting in about 1 in 10 patients being assessed. In order to allow for comparisons between long tally sheet and interview data, the periods for application of long tally sheets will be aligned with realisation of patient interviews. The long tally sheet will cover all variables from the short tally sheet (see Q2 and '***Q3_Long Patient Tally Sheet.pdf***'), in addition to assessment of educational level (1 question), attempts on cutting down drinking (2 questions), alcohol health literacy (4 questions), and injunctive social norms (2 questions). As with short tally sheets, long tally sheets will also be collected weekly by local researchers and entered into spreadsheet templates (see '***Q3_Long Patient Tally Sheet_spreadsheet template.xlsx***'). These spreadsheets will be submitted to the data center whenever data were collected.

For peer review only

Q4) Tally Sheets Cover Form (quantitative):
Short and long tally sheets will be distributed to the PHCCs by local researchers on a weekly basis and each set of tally sheets will have a cover form (see '***Q4_Tally Sheets Cover Form.pdf***'). On this cover form, the PHCC administration will be asked to fill in the number of adult consultations during the respective week for each participating provider. The cover forms will be collected together with the short/long tally sheets and will be entered in the same spreadsheets and then submitted to the data center.

Q5) Tally Sheet Appendix (consent taking for patient interview):
In predefined weeks during month 3 of the 18-month implementation period, PHCC providers will ask all patients to participate in researcher-conducted personal interviews. Patient consent and contact details will be collected on a form appended to either short or long tally sheets during these weeks (see '***Q5_Patient Tally Sheet Appendix.pdf***'). To allow for a stratified sampling of interviewees according to screening results (ratio of positively and negatively screened patients = 2:1) by local researchers, the providers will also note down the AUDIT-C screening result on the form. These forms will be collected alongside the short/long tally sheets and the data will only be used to sample and recruit interviewees.

Q6) Patient interview data:
Collection of individual data through patient interviews at month 3 and subsequent follow-ups at months 6 and 12. Random samples of positively and negatively screened patients (ratio 2:1) will be interviewed across all municipalities, resulting in a total number of N=1,080 patients. The interview will contain all questions from the long tally sheet (see '***Q3_Long Patient Tally Sheet.pdf***'), in addition to 2 questions for quality control assessing experience of screening/brief advice with PHCC providers, a six-item modified version of the HLS-EU-16 to assess alcohol health literacy, the World Health Organization Disability Assessment Schedule to assess the degree of disability, and questions on health resource utilization (see '***Q6_Patient Interview.pdf***'). The patient interview will be conducted as face-to-face or telephone interview and collected data will be entered into prepared spreadsheets (see '***Q6_Patient interview_spredsheet sample.xlsx***') and sent to the data center.

Q7) Provider questionnaire data (quantitative):
Collection of data from health care providers, which will be assessed prior to or during the 4-week baseline period and repeated at months 4.5 and 13.5. All providers will be asked to fill out questions on alcohol knowledge, alcohol health literacy, as well as on attitudes towards alcohol users and alcohol problems (SAAPP Questionnaire, see '***Q7_Provider questionnaire.pdf***'). The data will be entered into prepared spreadsheets (see '***Q7_Provider questionnaire_spredsheet sample.xlsx***') and sent to the data center.

Q8) Provider interview data (qualitative):
At the end of the 18-month implementation period, a random sample of 1 in 20 PHCC providers of both control and intervention groups will be invited to participate in a 15 minute semi-standardized interview (see '***Q8_Provider Interview from Annexe 25.pdf***'), which will be taped and conducted via telephone. The interviews aim to assess provider experiences on implementing the intervention package in their routines. Recordings of the provider interviews will be transcribed.

Q9) Process data interviews (qualitative):

For peer review only

As part of the process evaluation, semi-structured focus-group interviews will be conducted with the User Panels, Community Advisory Boards, and local research groups. The focus groups will cover the topics of tailoring of materials, and decision making processes for adoption mechanisms, support systems, and completing driver diagrams and barriers and facilitator tables.

Q10) Recruitment documentation (quantitative):

Local researchers will be given forms to document the entire PHCC recruitment process (see '***Q10_Recruitment documentation.pdf***'). For each municipality, they will document the total number of PHCCs and the number of contacted PHCCs for study participation. Among contacted PHCCs, the number of non-responding, refusing, and accepting PHCCs will be assessed. For each PHCC contacted for study participation, the following data will be assessed: number of registered patients and number of workers, type and number of contacts with PHCC, PHCC response (acceptance, refusal, non-response), and reasons for refusal or non-response if applicable. The data will be entered into prepared spreadsheets (see '***Q10_Recruitment documentation_spreadsheet template.xlsx***') and sent to the data center.

Q11) Follow-up documentation (quantitative):

Local researchers will monitor key activities of each PHCC provider during the course of the study using a standardized sheet (see '***Q11_Follow-up documentation.pdf***'). Key activities to be documented relate to participation in training sessions and potential reasons for non-participation. If providers drop out of the study prior to end of the 18 months implementation period, this will also be documented, in addition to any reasons for drop out. On the same follow-up documentation form, sex and age of the provider will be assessed as well. The data will be entered into prepared spreadsheets (see '***Q11_Follow-up documentation_spreadsheet template.xlsx***') and sent to the data center.

All quantitative data will be collected directly by PHC providers and the country research teams, through patient interviews or provider surveys.

Data types, format, and size

The total size of all quantitative data collected in the course of this study is unlikely to exceed 100MB and will be stored as easily accessible spreadsheets (.csv - format). Transcripts from qualitative interviews will be stored as Microsoft Word documents (.docx - format), not exceeding 100MB in total.

Purpose of data collection with regard to study objectives

The quantitative data will be required to evaluate if study objectives can be reached (for an overview of the study objectives, see '***Figure_RE-AIM.png***'). In particular, Q2 (short tally sheet), Q3 (long tally sheet) and Q4 (patient interview) data will provide outcome measures, which allows for evaluation of the ***REACH*** (maximising exposure to screening and brief advice/treatment in PHC) and ***EFFECTIVENESS*** (increasing adequate alcohol management in PHC) study objectives.

All qualitative data will be obtained through interviews with User Panels, Community Advisory Boards, local research groups, patients and providers, which will be used to evaluate the ***IMPLEMENTATION*** (factors affecting the implementation of intervention package) and ***ADOPTION*** (increase adoption of the intervention package in PHC) study objectives.

For peer review only

Furthermore, publicly available and process data will be obtained during the course of the study. In detail, this will comprise information necessary to characterize countries, cities and municipalities, contextual, political, socio-economic, and alcohol policy factors (e.g. legislation), and a thorough description of Community Advisory Boards. These data will contribute to the process evaluation (Work Package 5) and serve as base to evaluate the ***MAINTENANCE*** (long term effects of implementation) study objective.

A detailed description of the analytic steps planned to achieve study objectives can be found in ***section 7***.

Re-using data

Most of the data collected during the course of this study will be primary data collected through health care professionals and from patients directly. However, publicly available data form an important pillar in this study as it will be required for process evaluation and economic analyses.

Data utility

The collected data will not only be used to achieve the above listed study goals; they can be used by other researchers to plan similar studies, to examine other hypotheses, or for population modelling purposes.

For peer review only

2. FAIR data

2.1. Making data findable, including provisions for metadata

Making data discoverable, identifiable, and locatable

All quantitative data sets will be made publicly available through the UK Data Service after publication of the results, or, at the latest, 12 months after the finalization of the study.[1] Each data set published with the UK Data Service will be attached with a unique 'Digital Objective Identifier' (DOI).

Data derived from qualitative interviews will not be stored in the UK data archive as anonymity of qualitative interviews cannot be ensured.

Naming conventions and version numbers

For all data sets a predefined title standard ("SCALA_data_*NAME*_v1_*DATE.csv*") and the same author group ("SCALA study group") will always be used. Within titles, consecutive version numbers will be used to facilitate updates and corrections to uploaded data sets and to ensure unambiguous identification of data sets.

Key word conventions

All stored data will be labelled with the following keywords: SCALA, Americas, Mexico, Peru, Colombia, Primary Health Care, Alcohol, Heavy Drinking, Depression, Prevention, Screening, Brief Advice, Treatment. Additional keywords will be considered to characterize the respective data set. As data on resource use will be used for economic analyses, data sets containing relevant data will further be classified using 'JEL Classification Codes'.[2]

Meta data handling

There are no standards on handling metadata in this discipline and there is no intention to manage metadata of the publicly stored data sets apart from the measures listed above.

2.2. Making data openly accessible

Making data openly available

By default, all quantitative datasets generated in the course of the SCALA study will be made openly available through the UK Data Service upon publication of the results. Prior to publication, all data will be formatted to meet UK Data Service requirements.

Access conditions and required software

All quantitative data will be provided as 'comma separated values' (CSV) – an efficient and open source format to store larger data sets. This is a generic, widely used file format, which can be handled by all major software packages used for quantitative analyses (eg. Microsoft Excel, SAS, SPSS, Stata, R). In

[1] http://www.data-archive.ac.uk/
[2] https://en.wikipedia.org/wiki/JEL_classification_codes

For peer review only

order to maintain accessibility, large data sets will be split into smaller parts, which will not exceed 50 MB file size.

Depositing metadata, documentation, and code

Each dataset stored with the UK Data Service will be accompanied by a set of documenting files, which comprises relevant publications, consent forms, questionnaires/interview guidelines, and codebooks. The codebooks stored alongside the dataset will be Excel files (".xlsx") that contain extensive metadata for each variable in the associated data set, such as original questions, value labels, defined missing values, and possible coding rules applied.

Arrangements with the UK Data Service

The UK Data Service has been contacted and the study team received a positive response with regard to storing study data with the service. When preparing files to be published online, guidelines and checklists of the UK Data Service will be considered (see [3,4]). Licence agreements will be finalized after obtaining approval of all IRBs.

Data not being made available

All qualitative data will be generated from semi-standardized interviews. Excerpts of these interviews will be appended to respective publications if applicable. However, full interview transcripts will not be published for the following reasons: first, sharing full interview transcripts is uncommon in this field; and, second, sharing poses a potential risk for disclosing the identity of the interviewee.

Restrictions of use and data access committee

As all relevant data will be made publicly available, there will be no need for a data access committee. If other researchers wish to examine interview transcripts, fully anonymized excerpts can be made available through the responsible researchers.

Ascertainment of identity of person accessing the data

It is aimed that all relevant data are to be shared as 'Open Data'.[5] This will imply that all data will be fully anonymized and there will be no means necessary to ascertain the identity of persons accessing the data.

2.3. Making data interoperable

Interoperability of data

All gathered data will be completely interoperable as they will be stored in widely used data formats, which make them accessible by a broad spectrum of data processing software packages, including open source applications.

[3] https://www.ukdataservice.ac.uk/deposit-data/preparing-data
[4] https://www.ukdataservice.ac.uk/media/440320/depositsurvey.pdf
[5] https://www.ukdataservice.ac.uk/get-data/data-access-policy/open-data

For peer review only - http://bmjopen.bmj.com/site/about/guidelines.xhtml

Data and metadata vocabularies, standards, or methodologies

As there is no standard vocabulary set for variable names in our discipline, a simple and easy-to-comprehend nomenclature will be developed and applied to all quantitative data sets and summarized in accompanying codebooks. For prospective assessments on the same individuals, data sets will be structured in a 'long data format', i.e. one variable will indicate the time of assessment of the same variables (see [6] for a more comprehensive explanation).

2.4. Increase data re-use (through clarifying licences)

Data licence

All study data stored with the UK Data Service will be published as "open data" if possible. For this storage mode, the information in the data set will not allow disclosure of any respondents. "Open data" is published using the Open Government Licence[7] and users will have direct access of data without prior registration with UK data service, facilitating wide reach and potential re-use of data collected in this study.

Time of data availability

All quantitative data sets will be made publicly available after publication of the results, or, at the latest, 12 months after the finalization of the study.

Duration of data storage

All data stored with the UK Data Service are held in perpetuity (see [8]).

Re-use by third parties

Data re-use by third parties is explicitly encouraged and will be facilitated by publication of codebooks and documentation along the data sets.

Data quality assurance processes

Prior to sharing the data with the UK Data Service, the study team will clean the data to ensure internal consistency. Several checks of the study team will be conducted before the data will be shared publicly.

[6] http://www.theanalysisfactor.com/wide-and-long-data/
[7] http://www.nationalarchives.gov.uk/doc/open-government-licence/version/2/
[8] https://www.ukdataservice.ac.uk/media/173249/UKDS_Collections_Development_Policy_02_00.pdf

For peer review only

3. Allocation of resources

Costs for open access publications

In total, the study budget includes €36,000 to pay 'open access' publication licence fees.

Costs for sharing data through repository

Storage of study data with the UK Data Service does not require any fees.

Long term costs for preservation

No long term costs are anticipated.

Data protection, data transfer and data sharing

The Data Protection Officers of both Technical University Dresden and of Maastricht University are the focal points for reviewing data protection, data transfer and data sharing, and required ethics reporting.

For peer review only

4. Data security

Data security - transfer

All collected data will be transferred to the data center in encrypted packages created with the open access 7-zip software. The 'Advanced Encryption Standard' (AES) with 256 bits will be applied, which has been widely recognized as standard encryption technique [9]. The same data transfer methods will be used to transfer the data to the other partners who request or need the data.

Copies of transcribed data notes that are required for the process evaluation in Work Package 6 will be sent by registered courier to ESADE.

Data security - storage

All electronic data will be stored on encrypted hard drives by respective partners. This will include mail communication, study documentation and codes applied to manipulate data and to generate results. Backup hard drives will be used to facilitate recovery of lost data.

All analogue data sources (tally sheets, interview notes, etc.) will be kept by the local research teams, where the data will be kept and stored adhering to local regulations.

All data stored with the UK Data Service are securely kept for perpetuity.

For peer review only

[9] https://en.wikipedia.org/wiki/Advanced_Encryption_Standard

5. Ethical aspects

Ethical or legal issues regarding data sharing

After collection of the raw data, local researchers will assign predefined identification codes to each individual and remove all potentially identifying information from the data. The key to match individuals to the assigned identification code will remain with the local researchers. After the data has been securely transferred to the data center for cleaning and subsequent analyses, there will be no possibility no identify individuals from the data.

All data collection, processing, and sharing procedures will adhere to national and international laws including the General Data Protection Regulation (EU Regulation 2016/679).

The SCALA study team currently seeks approval for the study design, data collection and analysis from the research ethics board at the TU Dresden, Germany (registration number: 'EK 90032018'). In addition, ethical review is currently under way in Colombia, Mexico, and Peru.

Informed consent for data sharing and long term preservation

Informed consent will be obtained from providers and patients providing individual level data (through interviews or questionnaires) to allow data sharing through the UK Data Service.

For peer review only

6. Other issues

Use of other procedures for data management

Data management in the SCALA study will adhere to EU Regulation 2016/679. There are no further national or institutional requirements which would counteract or extend this regulation or any of the procedures specified in this document.

For peer review only

7. Data analysis plan

In Section 1, data sources are mapped to study goals. For each study goal, the required definition of variables and planned statistical analyses are described in the following.

General considerations

Given that SCALA is a quasi-experimental study design (technically, a non-randomized controlled trial (NRCT)), data for a range of potential confounders will be collected at baseline (with repeat measurements during the course of the 18-month implementation period) both to undertake propensity score matching between intervention and comparator municipalities, and include as confounders in the statistical analyses:

<u>At the level of the PHCC, PHC-provider and patient:</u>

- *Age, sex and profession (doctor, nurse, other health care worker) of provider:* Evidence suggests that the sex and age of the provider are unimportant in influencing screening and advice rates, whereas profession is. Nurses tend to screen more patients than doctors; doctors tend to advise more screen positive patients than nurses.
- *Number of monthly consultations:* Evidence suggests that the higher the number of consultations, the lower the proportion of patients screened.
- *Attitudes and knowledge of providers:* Evidence suggests that providers with more positive attitudes, in terms or role security and therapeutic commitment, and providers with high levels of alcohol-related knowledge, are more likely to screen and advise a greater proportion of patients.
- *AUDIT-C score:* The evidence suggests that the higher the AUDIT-C score, the greater the likelihood that screen positive patients will be given advice.

<u>At the level of the municipality:</u>

- A priori, comparator municipalities have been chosen to be similar to intervention municipalities in terms of socioeconomic and other characteristics which impact on drinking, health care and survival, comparable community mental health services. During the set-up phase, additional data will be collected form the municipalities on existing actions and training of PHC-based screening and brief advice for heavy drinking; availability and accessibility of specialist services for severe AUD and moderately severe or severe depression; and, existing municipal-based prevention and/or policy programmes to reduce heavy drinking

7.1. REACH

Primary outcome measures:

A1 Number of intervened patients per provider and per PHCC

Secondary outcome measures:

A2 Number of screened patients per provider and per PHCC
A3 Number of advised patients per provider and per PHCC
A4 Number of patients referred for severe AUD per provider and per PHCC

For peer review only - http://bmjopen.bmj.com/site/about/guidelines.xhtml

A5 Number of patients referred for moderately severe or severe depression per provider and per PHCC
A6 Provider attitudes
A7 Provider alcohol health literacy
A8 Representativeness of population intervened for AUD

Definition:

Measure A1 represents the *primary* outcome variables in this study and is assessed in three 4-week periods: in the first month 1 (t1), after 9 months (t2) and after 18 months (t3). It will be the proportion of consulting adult patients (aged 18+ years) intervened (screened and advice given to screen positives), calculated as the number of AUDIT-C positive patients that received oral advice or referral for advice to another provider in or outside the PHCC, divided by the total number of adult consultations of the participating providers per provider and per PHCC.

Measures A2 to A5 represent *secondary* outcome variables in this study and are assessed in the same three 4-week periods as measure A1: in the first month 1 (t1), after 9 months (t2) and after 18 months (t3). Measure A2 will be the proportion of patients screened, calculated as the number of completed screens divided by the total number of consultations of all adult patients per participating provider, and averaged per participating PHCC. Measure A3 will be the proportion of patients advised, calculated as the number of brief interventions delivered (received oral brief advice, and/or were referred to another provider in or outside the practice), divided by the total number of screen positives per participating provider and averaged per participating PHCC. Information will also be collected on the number of screen negatives who received brief advice. Measure A4 will be the proportion of patients with severe AUD referred to specialist treatment, calculated as the proportion of patients with an AUDIT-C score ≥8 and a full AUDIT score ≥20 documented as referred to treatment per participating provider, and per participating PHCC. Measure A5 will be the proportion of patients with moderately severe or severe depression referred to specialist treatment, calculated as the proportion of patients with an AUDIT-C score ≥8 and a PHQ-9 score ≥15 documented as referred to treatment per participating provider, and per participating PHCC.

Measures A6 and A7 are also *secondary* outcome variables in this study and will be assessed in three 4-week periods through provider questionnaires: at baseline (t1), after 4.5 months (t2) and after 13.5 months (t3). Measure A6 will be measured by the SAAPP questionnaire, with responses to be summed within the two scales of role security and therapeutic commitment. Individual missing values for any of the items in a domain will be assigned the mean value of the remaining items of the domain before summation. Measure A7 will be assessed through knowledge of risks due to drinking, and reported descriptive and injunctive social norms of drinking. Measure A8 will be determined through process evaluation activities conducted throughout the implementation period. Among other things, representativeness will be evaluated through comparing patients with people living in the catchment area of the respective PHC on a number of variables.

Analyses/Achievement:

For all measures, means and/or proportions (as applicable) will be presented descriptively by country, control and intervention municipality, and for the total sample. Given the relative rarity of some events (eg. measure A1 to A5) and the resulting distribution, we will use exact inference methods for comparison of intervention vs. comparator municipalities.

For peer review only - http://bmjopen.bmj.com/site/about/guidelines.xhtml

For further analyses, including covariates, regression models will be used, taking into consideration the hierarchical nature of the data, and characteristics at different hierarchy levels (i.e., characteristics of the PHCC, characteristics at the municipal level, such as patterns of drinking). Multilevel models are well suited for this purpose and will be built to evaluate the intervention effect for measures A1 to A7. For the primary outcome, the model will be built as follows:

- *Dependent variable:* proportion of patients intervened among all consultations per provider and per PHCC
- *Independent variable 1:* Time (t1-t3)
- *Independent variable 2*: Control vs. intervention municipality
- *Hierarchical cluster*: Provider nested within PHCC nested within country (to control for design effects)
- *Statistic*: Interaction effect between time and group allocation

After testing for the necessary assumptions, the above outlined generalized linear model will be applied to the actual distribution of the outcome measure. Thus, skewness of data resulting from rare events would be analysed using zero-inflated negative binomial regression. For all remaining outcome measures, similar models will be applied.

7.2. EFFECTIVENESS

Outcome measures:

B1 Increased health literacy in PHCC patients using a modified version of the UK-based Newest Vital Sign and a six-item adapted version of Health Literacy Survey-EU Questionnaire (HLS-EU-16)

B2 Reduction in alcohol consumption of AUD+ drinkers

Definition:

Data for measures B1 and B2 are collected through patient interviews (conducted in month 3, 6 and 12).

Analyses/Achievement:

Similar multilevel regression models as applied for primary and secondary outcomes mapped to study goal *REACH* will be applied to measures B1 and B2. The main difference will be that these measures will be analyzed on the individual level, which requires adding another level (patient nested with provider nested within PHCC nested within country) to the model.

7.3. ADOPTION

Outcome measures:

C1 Adoption rate and representativeness of PHCCs

C2 Adoption rate and representativeness of PHCC staff

Definition:

Adoption rate of PHCCs will be calculated as the number of PHCCs agreeing to be part of the study divided by the number of PHCCs contacted.

For peer review only - http://bmjopen.bmj.com/site/about/guidelines.xhtml

Adoption rate of PHCC providers within each PHCC that joins the study will be calculated as the number of PHCC providers agreeing to be part of the study divided by the total number of PHCC providers within each PHCC, stratified by profession (doctor, nurse, other).

Analyses/Achievement:

To determine the representativeness of PHCCs involved in the study, routine available data on the size, number of registered patients, and number and characteristics of staff will be used and compared between PHCCs who agreed to be part of the study and contacted PHCCs who declined to be part of the study.

To determine the representativeness of PHCC staff within the involved PHCC, routine available data on the number and characteristics of staff will be used to compare, within each PHCC, those staff who joined the study and those staff who declined to join the study.

7.4. IMPLEMENTATION

Outcome measures:

D1 Extent primary health care screening and advice package delivered as intended

D2 Multi-level evaluation of barriers/facilitators to scale-up using WHO's Urban Health Equity Assessment and Response Tool

D3 Extent implementation on city levels delivered as intended using Medical Research Council guidance

D4 Cost of package implementation

Definition:

All measures D1 to D3 will be assessed through process evaluation activities. The required data will be obtained through interviews with PHCC providers (D1) and with members from Community Advisory Boards (D2, D3). For D4, a comprehensive set of data will be required, comprising patient data on disability and health resource utilization obtained from patient interviews as well as data on unit costs obtained from public data sources.

Analyses/Achievement:

Measures D1 to D3 will be analyzed through qualitative evaluation. Measure D4 will be evaluated by a comprehensive economic evaluation, for which different sources of costs will be considered, such as costs attributable to implementation of the intervention routine as well as costs attributable to utilization of health care services. In a cost-effectiveness study, the hypothesized gain in quality of life among patients in intervention municipalities will be contrasted with recorded and calculated costs.

7.5. MAINTENANCE

Process measures:

E1 Assessment of outcomes 18 months post implementation

E2 Indicators of program-level maintenance

For peer review only - http://bmjopen.bmj.com/site/about/guidelines.xhtml

E3 Measures of cost of maintenance
E4 Dissemination / events

Definition:

For measure E1 data from PHC providers and patients up to 18 months after implementing the alcohol management routine need to be collected.

For measure E2, the required indicators will be collected through process evaluation activities, namely interviews with members of the Community Advisory Boards.

For measure E3, all costs will be collected throughout the implementation period within the economic evaluation framework (see measure D4), in order to estimate the costs of maintenance.

For measure E4, the study results will be disseminated through municipal, national, and international structures, following the 'Communication, Dissemination and Exploitation Plan'.

Analyses/Achievement:

Measure E1 will be achieved by continuous data collection across the entire implementation period of 18 months.

Measure E2 will be achieved by analysis of qualitative data. Measure E3 will be achieved through an economic evaluation of the implementation package considering the entire implementation period.

Measure E4 will be achieved by following the 'Communication, Dissemination and Exploitation Plan'.

For peer review only

8. Appendix

List of all documents referenced in the DMP:

Document	Page Number
1. Q1_PHCC Description Form template.pdf	18
2. Q1_PHCC Description Form_spreadsheet template.xlsx	Excel not attached
3. Q2_Short Patient Tally Sheet.pdf	19
4. Q2_Short Patient Tally Sheet_spreadsheet template.xlsx	Excel not attached
5. Q3_Long Patient Tally Sheet.pdf	22
6. Q3_Long Patient Tally Sheet_spreadsheet template.xlsx	Excel not attached
7. Q4_Tally Sheet Cover Form.pdf	26
8. Q5_Tally Sheet Appendix.pdf	27
9. Q6_Patient Interview.pdf	29
10. Q6_Patient interview_spreadsheet template.xlsx	Excel not attached
11. Q7_Provider questionnaire.pdf	34
12. Q7_Provider questionnaire_spreadsheet template.xlsx	Excel not attached
13. Q8_Provider Interview from Annexe 25.pdf	36
14. Q10_Recruitment documentation.pdf	53
15. Q10_Recruitment documentation_spreadsheet template.xlsx	Excel not attached
16. Q11_Follow-up documentation.pdf	55
17. Q11_Follow-up documentation_spreadsheet template.xlsx	Excel not attached
18. Figure_RE-AIM.png	58

For peer review only

PHCU Description Form

Country and municipality details
(to be filled in by local research team)

**Country**	☐ Colombia	☐ Mexico	☐ Peru
**Municipality**	________________	**Control or Experimental**	☐ Control<br>☐ Experimental
**ID of PHCU**	________________		

PHCU details
(to be filled in by PHC administration)

**Name/Address of PHCU**		________________	
**Total number of registered patients**		________________	
**Total number of registered *adult* (18+) patients**		________________	
**Number of workers working in PHCU**	General Practitioners	**Part time**	________________
		**Full time**	________________
	Nurses	**Part time**	________________
		**Full time**	________________
	Assistants	**Part time**	________________
		**Full time**	________________
	Psychologists	**Part time**	________________
		**Full time**	________________
	Social workers	**Part time**	________________
		**Full time**	________________
	Others: __________	**Part time**	________________
		**Full time**	________________

For peer review only

Short Tally Sheet

Provider details and consultation

**Practice ID** (*pre-printed*)	__________________	**Provider ID / Name** (*pre-printed*)	__________________
**Date consultation**	____ / ____ / ____		

Patient details

**Sex**	☐ Male<br>☐ Female<br>☐ Other	**Age**	______ years
**Socioeconomic status**	☐ Below average	☐ Average	☐ Above average

AUDIT-C Alcohol Screening

Questions	0	1	2	3	4	Score
1 How often do you have a drink containing alcohol?	Never	Monthly or less	2-4 times per month	2-3 times per week	4+ times per week	
2 How many units of alcohol do you drink on a typical day when you are drinking?	1-2	3-4	5-6	7-9	10+	
3 How often do you have 6 or more units on one occasion?	Never	Less than monthly	Monthly	Weekly	Daily or almost daily	

Standard Drinks Placeholder

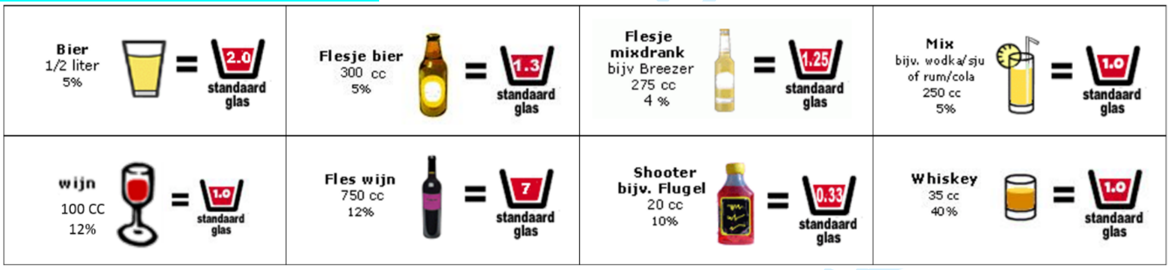

**Sum score AUDIT-C (possible range 0-12)**	__ __
**If AUDIT-C score ≥ 8 => Apply remaining AUDIT and PHQ-2 questionnaire**	

AUDIT (remaining scale)

Questions	0	1	2	3	4	Score
4 How often during the last year have you found that you were not able to stop drinking once you had started?	Never	Less than monthly	Monthly	Weekly	Daily or almost daily	
5 How often during the last year have you failed to do what was normally	Never	Less than monthly	Monthly	Weekly	Daily or almost daily	

For peer review only

Short Tally Sheet

	expected from you because of drinking?						
6	How often during the last year have you needed a first drink in the morning to get yourself going after a heavy drinking session?	Never	Less than monthly	Monthly	Weekly	Daily or almost daily	
7	How often during the last year have you had a feeling of guilt or remorse after drinking?	Never	Less than monthly	Monthly	Weekly	Daily or almost daily	
8	How often during the last year have you been unable to remember what happened the night before because you had been drinking?	Never	Less than monthly	Monthly	Weekly	Daily or almost daily	
9	Have you or someone else been injured as a result of your drinking?	No		Yes, but not in the last year		Yes, during the last year	
10	Has a relative or friend or a doctor or another health worker been concerned about your drinking or suggested you cut down?	No		Yes, but not in the last year		Yes, during the last year	
	**Sum score (possible range 0-28)**						__ __
	**Sum score full AUDIT (possible range 0-40)**						__ __
	**If full AUDIT score ≥ 8 => Apply remaining AUDIT and PHQ-2 questionnaire**						

PHQ-2 Depression Screening

**Over the last 2 weeks, how often have you been bothered by any of the following problems?**					
		**Not at all**	**Several days**	**More than half the days**	**Nearly every day**
1	Little interest or pleasure in doing things	0	1	2	3
2	Feeling down, depressed, or hopeless	0	1	2	3
	**Sum score (possible range 0-6)**			__ __	
	**If PHQ-2 score ≥ 3 => Apply remaining PHQ questionnaire**				

PHQ-9 (remaining scale)

**Over the last 2 weeks, how often have you been bothered by any of the following problems?**					
		**Not at all**	**Several days**	**More than half the days**	**Nearly every day**
3	Trouble falling or staying asleep, or sleeping too much	0	1	2	3
4	Feeling tired or having little energy	0	1	2	3
5	Poor appetite or overeating	0	1	2	3

For peer review only - http://bmjopen.bmj.com/site/about/guidelines.xhtml

Short Tally Sheet

6	**Feeling bad about yourself or that you are a failure or have let yourself or your family down**	0	1	2	3
7	**Trouble concentrating on things, such as reading the newspaper or watching television**	0	1	2	3
8	**Moving or speaking so slowly that other people could have noticed. Or the opposite being so figety or restless that you have been moving around a lot more than usual**	0	1	2	3
9	**Thoughts that you would be better off dead, or of hurting yourself**	0	1	2	3
	**Sum score (possible range 0-21)**	__ __			
	**Sum score full PHQ-9 (possible range 0-27)**	__ __			

Taking record of brief advice or referral

**If full AUDIT < 26 and PHQ-9 < 15:**	
**Brief advice** *(more than one answer is possible)*	☐ Oral Brief Advice given<br>☐ Patient Leaflet given<br>☐ Continued Monitoring<br>☐ Patient referred to other provider in practice for brief advice<br>☐ Patient referred to other provider outside practice for brief advice<br>☐ Other ________________________<br>☐ Time did not allow, but<br>☐ I made follow-up appointment<br>☐ Patient declined brief advice<br>☐ Patient not screen positive, but reinforced about keeping low risk drinking habits
**If full AUDIT ≥ 26 and/or PHQ-9 ≥ 15:**	
**Patient referred to special services:**	☐ Yes<br>☐ No

For peer review only

Long Tally Sheet

Provider details and consultation

**Practice ID** (*pre-printed*)	________________	**Provider ID / Name** (*pre-printed*)	________________
**Date consultation**	____ / ____ / ____		

Patient details

**Sex**	☐ Male<br>☐ Female<br>☐ Other	**Age**	______ years
**Socioeconomic status**	☐ Below average	☐ Average	☐ Above average
**Highest level of education**	☐ No schooling completed<br>☐ Junior high school completed<br>☐ Business/Technical training<br>☐ Doctorate degree		☐ Primary school completed<br>☐ High school completed<br>☐ Bachelor's/Master's degree

Alcohol exposure, health literacy, and social norms

During the last 12 months have you tried to cut down on your drinking by:		
Choosing lower strength alcohol	☐ Yes	☐ No
Using smaller glasses	☐ Yes	☐ No
How easy is it to understand health information about drinking of alcohol?	☐ Always easy<br>☐ Usually easy	☐ Sometimes difficult<br>☐ Often difficult<br>☐ Always difficult
To the best of your knowledge, can drinking alcohol cause any of the following:		
High blood pressure	☐ Yes	☐ No
Liver problems	☐ Yes	☐ No
Cancer	☐ Yes	☐ No
Thinking about your friends, would you say that it is acceptable or unacceptable for them to drink:		
Regularly more than two drinks a day?	☐ Acceptable	☐ Unacceptable
More than six drinks on an occasion?	☐ Acceptable	☐ Unacceptable

AUDIT-C Alcohol Screening

Questions		0	1	2	3	4	Score
1	**How often do you have a drink containing alcohol?**	Never	Monthly or less	2-4 times per month	2-3 times per week	4+ times per week	
2	**How many units of alcohol do you drink on a typical day when you are drinking?**	1-2	3-4	5-6	7-9	10+	

For peer review only

Long Tally Sheet

3	**How often do you have 6 or more units on one occasion?**	Never	Less than monthly	Monthly	Weekly	Daily or almost daily	

Standard Drinks Placeholder

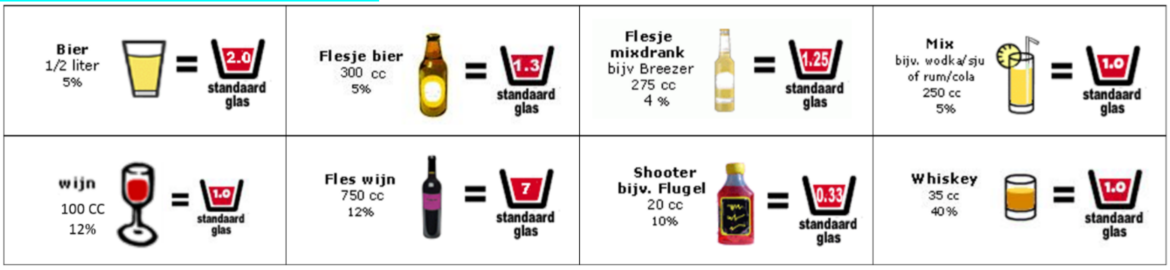

**Sum score AUDIT-C (possible range 0-12)**	__ __
**If AUDIT-C score ≥ 8 => Apply remaining AUDIT and PHQ-2 questionnaire**	

AUDIT (remaining scale)

Questions		0	1	2	3	4	Score
4	**How often during the last year have you found that you were not able to stop drinking once you had started?**	Never	Less than monthly	Monthly	Weekly	Daily or almost daily	
5	**How often during the last year have you failed to do what was normally expected from you because of drinking?**	Never	Less than monthly	Monthly	Weekly	Daily or almost daily	
6	**How often during the last year have you needed a first drink in the morning to get yourself going after a heavy drinking session?**	Never	Less than monthly	Monthly	Weekly	Daily or almost daily	
7	**How often during the last year have you had a feeling of guilt or remorse after drinking?**	Never	Less than monthly	Monthly	Weekly	Daily or almost daily	
8	**How often during the last year have you been unable to remember what happened the night before because you had been drinking?**	Never	Less than monthly	Monthly	Weekly	Daily or almost daily	
9	**Have you or someone else been injured as a result of your drinking?**	No		Yes, but not in the last year		Yes, during the last year	
10	**Has a relative or friend or a doctor or another health worker been concerned about your drinking or suggested you cut down?**	No		Yes, but not in the last year		Yes, during the last year	
	**Sum score (possible range 0-28)**						__ __
	**Sum score full AUDIT (possible range 0-40)**						

For peer review only

Long Tally Sheet

	__ __
**If full AUDIT score ≥ 8 => Apply remaining AUDIT and PHQ-2 questionnaire**	

PHQ-2 Depression screening

Over the last 2 weeks, how often have you been bothered by any of the following problems?	Not at all	Several days	More than half the days	Nearly every day
1 Little interest or pleasure in doing things	0	1	2	3
2 Feeling down, depressed, or hopeless	0	1	2	3
Sum score (possible range 0-6)			__ __	
**If PHQ-2 score ≥ 3 => Apply remaining PHQ questionnaire**				

PHQ-9 (remaining scale)

Over the last 2 weeks, how often have you been bothered by any of the following problems?	Not at all	Several days	More than half the days	Nearly every day
3 Trouble falling or staying asleep, or sleeping too much	0	1	2	3
4 Feeling tired or having little energy	0	1	2	3
5 Poor appetite or overeating	0	1	2	3
6 Feeling bad about yourself or that you are a failure or have let yourself or your family down	0	1	2	3
7 Trouble concentrating on things, such as reading the newspaper or watching television	0	1	2	3
8 Moving or speaking so slowly that other people could have noticed. Or the opposite being so figety or restless that you have been moving around a lot more than usual	0	1	2	3
9 Thoughts that you would be better off dead, or of hurting yourself	0	1	2	3
Sum score (possible range 0-21)	__ __			
Sum score full PHQ-9 (possible range 0-27)	__ __			

Taking record of brief advice or referral

**If full AUDIT < 26 and PHQ-9 < 15:**	
**Brief advice** *(more than one answer is possible)*	☐ Oral Brief Advice given<br>☐ Patient Leaflet given<br>☐ Continued Monitoring<br>☐ Patient referred to other provider in practice for brief advice<br>☐ Patient referred to other provider outside practice for brief advice<br>☐ Other<br>________________________<br>☐ Time did not allow, but<br>☐ I made follow-up appointment

For peer review only

Long Tally Sheet

	☐ Patient declined brief advice<br>☐ Patient not screen positive, but reinforced about keeping low risk drinking habits
**If full AUDIT ≥ 26 and/or PHQ-9 ≥ 15:**	
**Patient referred to special services:**	☐ Yes<br>☐ No

For peer review only

Tally Sheets Cover Form

Provider details, consultation and type of tally sheets
(to be filled in by local research team)

**Practice ID**	[pre-print]	**Provider ID / Name**	[pre-print]
**Consultation period**	____ / ____ / ____ - ____ / ____ / ____ (DD / MM / YY)		
**Type of tally sheets**	☐ Short tally sheets	☐ Long tally sheets	

Adult consultations
(to be filled in by PHC provider or administrator)

**Number of adult consultations during consultation period for this provider**	_ _ _ _

For peer review only

Tally Sheet Appendix

PHC provider and consultation details

**Practice ID** (*pre-printed*)	________________	**Provider ID / Name** (*pre-printed*)	________________
**Date consultation**	____/____/____		

Patient interview

**Alcohol screening result**	☐ Positive (AUDIT-C >= 8)	☐ Negative (AUDIT-C < 8)
**Asked patient for interview participation**	☐ Yes	☐ No
**Patient interested in interview participation**	☐ Yes	☐ No

Patient contact details for interview
(only if patient expressed interest in interview participation)

**Name**	____	
**Telephone number**	____	
**Address**	____	
**Preferred mode of interview**	☐ Face-to-face	☐ Telephone

Interview information

Introduction

The SCALA Study aims to find out the extent to which screening and brief advice implemented in primary health care can be increased to reduce the harmful use of alcohol. The study is taking place in cities from three countries from Latin America.
The harmful use of alcohol is prevalent in any countries, and alcohol, itself, is the seventh most important risk factor world-wide for ill-health and premature death (after high blood pressure, tobacco use, high fasting plasma glucose, high body mass index, poor diet, and low birthweight and short gestation).

Aim of the study

In this study, we aim to determine the extent of adequate prevention and management of harmful alcohol use in primary health care settings. Another major objective of this study is to improve the health of patients consulting primary health care centers.
The interview will take about 15 minutes and will cover questions on alcohol consumption, alcohol knowledge, wellbeing, and other health behavior. The same interview will be repeated twice, 3 and

For peer review only

9 months after the initial interview. Due to logistical reasons, not all patients agreed to be interviewed will eventually be asked for participation. If you have not been selected for interview participation, your contact details will be destroyed right away.

Data Handling and Sharing

Participation in this interview is entirely voluntary and you are free to skip any of the interview questions. During the interview, you will be asked questions on your personal wellbeing and health. The collected data will be entered into data bases and personal identifying information (such as name, address, and date of birth) will be replaced with an abstract personal identifier, the key to which remains with the local academic only. The data bases will be submitted to the data center at TU Dresden ('Technische Universität Dresden') in Germany using up-to-date encryption techniques. Here, all study data will be stored on encrypted hard drives and processed for further data analyses to be conducted by the study team. At all times, both analogue and digital data will be stored in secure environments. After publication of the study results, the relevant study data will be shared through the UK Data Service – a non-commercial data respository allowing other researchers to re-use the collected data for an indefinite period of time. All data shared through the UK Data Service will bear no risk of disclosure of the identity of the PHCC or of the participating providers.

Interview consent

Please check box

1. I confirm that I have read and understand the information for participating in the SCALA patient interview and have had the opportunity to ask questions. ☐

1. I consent that my contact details will be given to the SCALA study team and agree that the SCALA study team can use the contact details to ask me for interview participation and for repeating the interview. ☐

2. I understand that my participation is voluntary and that I am free to not participate, without giving any reason. ☐

3. I confirm that I have understand that study data collected through me will be processed at the TU Dresden (Germany) and shared through the UK Data Service. ☐

4.

___________________________	_________________	_____________________
Name of patient	Date	Signature

For peer review only

PATIENT INTERVIEW

Formalities

**Practice ID** *(pre-printed)*	____________	**Provider ID / Name** *(pre-printed)*	____________
**Patient ID** *(filled in by interviewer)*	____________	**Interview date**	____ / ____ / ____

Sociodemographics

**Sex**	☐ Male<br>☐ Female<br>☐ Other	**Age**	______ years
**Socioeconomic status**	☐ Below average	☐ Average	☐ Above average
**Highest level of education**	☐ No schooling completed<br>☐ Junior high school completed<br>☐ Business/Technical training<br>☐ Doctorate degree		☐ Primary school completed<br>☐ High school completed<br>☐ Bachelor's/Master's degree

Alcohol exposure, health literacy, and social norms

During the last 12 months have you tried to cut down on your drinking by:		
Choosing lower strength alcohol	☐ Yes	☐ No
Using smaller glasses	☐ Yes	☐ No
How easy is it to understand health information about drinking of alcohol?	☐ Always easy<br>☐ Usually easy	☐ Sometimes difficult<br>☐ Often difficult<br>☐ Always difficult
In the last 12 months, has any doctor or health worker asked you about how much alcohol you drink?	☐ Yes	☐ No
In the last 12 months, has any doctor or health worker advised you to reduce or stop drinking alcohol?	☐ Yes	☐ No
To the best of your knowledge, can drinking alcohol cause any of the following:		
High blood pressure	☐ Yes	☐ No
Liver problems	☐ Yes	☐ No
Cancer	☐ Yes	☐ No
Thinking about your friends, would you say that it is acceptable or unacceptable for them to drink:		
Regularly more than two drinks a day?	☐ Acceptable	☐ Unacceptable
More than six drinks on an occasion?	☐ Acceptable	☐ Unacceptable

For peer review only

AUDIT Alcohol Screening

Questions		0	1	2	3	4	Score
1	How often do you have a drink containing alcohol?	Never	Monthly or less	2-4 times per month	2-3 times per week	4+ times per week	
2	How many units of alcohol do you drink on a typical day when you are drinking?	1-2	3-4	5-6	7-9	10+	
3	How often do you have 6 or more units on one occasion?	Never	Less than monthly	Monthly	Weekly	Daily or almost daily	

Standard Drinks Placeholder

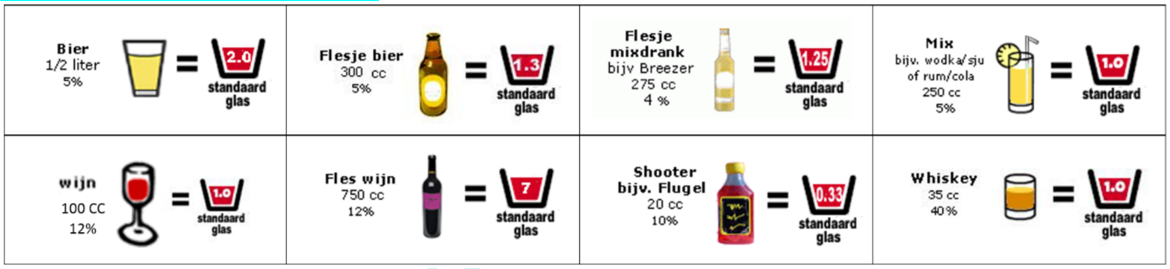

		0	1	2	3	4	Score
4	How often during the last year have you found that you were not able to stop drinking once you had started?	Never	Less than monthly	Monthly	Weekly	Daily or almost daily	
5	How often during the last year have you failed to do what was normally expected from you because of drinking?	Never	Less than monthly	Monthly	Weekly	Daily or almost daily	
6	How often during the last year have you needed a first drink in the morning to get yourself going after a heavy drinking session?	Never	Less than monthly	Monthly	Weekly	Daily or almost daily	
7	How often during the last year have you had a feeling of guilt or remorse after drinking?	Never	Less than monthly	Monthly	Weekly	Daily or almost daily	
8	How often during the last year have you been unable to remember what happened the night before because you had been drinking?	Never	Less than monthly	Monthly	Weekly	Daily or almost daily	
9	Have you or someone else been injured as a result of your drinking?	No		Yes, but not in the last year		Yes, during the last year	
10	Has a relative or friend or a doctor or another health worker been concerned about your drinking or suggested you cut down?	No		Yes, but not in the last year		Yes, during the last year	
	Sum score AUDIT (possible range 0-40)						__ __

PHQ-9 Depression Screening

Over the last 2 weeks, how often have you been bothered by any of the following problems?				
	Not at all	Several days	More than half the days	Nearly every day
1 Little interest or pleasure in doing things	0	1	2	3
2 Feeling down, depressed, or hopeless	0	1	2	3
3 Trouble falling or staying asleep, or sleeping too much	0	1	2	3
4 Feeling tired or having little energy	0	1	2	3
5 Poor appetite or overeating	0	1	2	3
6 Feeling bad about yourself or that you are a failure or have let yourself or your family down	0	1	2	3
7 Trouble concentrating on things, such as reading the newspaper or watching television	0	1	2	3
8 Moving or speaking so slowly that other people could have noticed. Or the opposite being so figety or restless that you have been moving around a lot more than usual	0	1	2	3
9 Thoughts that you would be better off dead, or of hurting yourself	0	1	2	3
Sum score PHQ-9 (possible range 0-27)	__ __			

Alcohol Literacy Assessment

On a scale from very difficult to very easy, how easy would you say it is to: …					
	Very difficult	Fairly difficult	Fairly easy	Very easy	Don't know
1 Question 1 Placeholder	0	1	2	3	5
2 Question 2 Placeholder	0	1	2	3	5
3 Question 3 Placeholder	0	1	2	3	5
4 Question 4 Placeholder	0	1	2	3	5
5 Question 5 Placeholder	0	1	2	3	5
6 Question 6 Placeholder	0	1	2	3	5
Sum score (possible range XX-XX)	__ __				

For peer review only

WHODAS 2.0 Disability Assessment

This questionnaire asks about difficulties due to health conditions. Health conditions include diseases or illnesses, other health problems that may be short or long lasting, injuries, mental or emotional problems, and problems with alcohol or drugs.

Think back over the past 30 days and answer these questions, thinking about how much difficulty you had doing the following activities. For each question, please circle only one response.

In the past 30 days, how much difficulty did you have in:						
	Questions	None	Mild	Moderate	Severe	Extreme or cannot do
1	Standing for long periods such as 30 minutes?	1	2	3	4	5
2	Taking care of your household responsibilities?	1	2	3	4	5
3	Learning a new task, for example, learning how to get to a new place?	1	2	3	4	5
4	Joining in community activities (for example, festivities, religious or other activities) in the same way as anyone else can?	1	2	3	4	5
5	How much have you been emotionally affected by your health problems?	1	2	3	4	5
6	Concentrating on doing something for ten minutes?	1	2	3	4	5
7	Walking a long distance such as a kilometre [*or equivalent*]?	1	2	3	4	5
8	Washing your whole body?	1	2	3	4	5
9	Getting dressed?	1	2	3	4	5
10	Dealing with people you do not know?	1	2	3	4	5
11	Maintaining a friendship?	1	2	3	4	5
12	Your day-to-day work?	1	2	3	4	5
	Sum score (possible range 0-60)	__ __				
H1	Overall, in the past 30 days, how many days were these difficulties present?	Record number of days: __ __ (0-30)				
H2	In the past 30 days, for how many days were you totally unable to carry out your usual activities or work because of any health condition?	Record number of days: __ __ (0-30)				
H3	In the past 30 days, not counting the days that you were totally unable, for how many days did you cut back or reduce your usual activities or work because of any health condition?	Record number of days: __ __ (0-30)				

For peer review only

Health resource utilization

Title Placeholder				
		Response 1	Response 2	Response 3
**1**	Question 1 Placeholder	0	1	2
**2**	Question 2 Placeholder	0	1	2
**3**	Question 3 Placeholder	0	1	2
**4**	Question 4 Placeholder	0	1	2
**5**	Question 5 Placeholder	0	1	2
**6**	Question 6 Placeholder	0	1	2

For peer review only

Primary Health Care Provider Questionnaire

Practice details and date

**Practice ID** (*pre-printed*)	__________________	**Provider ID / Name** (*pre-printed*)	__________________
**Date**	____ / ____ / ____	**Assessment**	☐ Baseline<br>☐ Follow-up 1<br>☐ Follow-up 2

Patient details

**Sex**	☐ Male<br>☐ Female<br>☐ Other	**Age**	_______ years
**Profession**	☐ Doctor<br>☐ Nurse<br>☐ Psychologist		☐ Practice Assistant<br>☐ Social worker<br>☐ Other: _______

Alcohol Knowledge

	Questions	Per Day	Per Week	Per Occasion
1	Experts recommend that everyone should limit the amount of alcohol that they drink. What is this limit for men, in terms of drinks:	_ _ drinks	_ _ drinks	_ _ drinks
2	Experts recommend that everyone should limit the amount of alcohol that they drink. What is this limit for women, in terms of drinks:	_ _ drinks	_ _ drinks	_ _ drinks

		Acceptable	Unacceptable
3	Would you say that it is acceptable or unacceptable for you to drink regularly more than two drinks a day?		
4	Would you say that it is acceptable or unacceptable for you to drink more than six drinks on anyone occasion?		
5	Would you say that it is acceptable or unacceptable for your friends to drink regularly more than two drinks a day?		
6	Would you say that it is acceptable or unacceptable for your friends to drink more than six drinks on anyone occasion?		

Alcohol Health Literacy

On a scale from very difficult to very easy, how easy would you say it is to: …

		Very difficult	Fairly difficult	Fairly easy	Very easy	Don't know
1	Question 1 Placeholder	0	1	2	3	5
2	Question 2 Placeholder	0	1	2	3	5

Primary Health Care Provider Questionnaire

**3**	**Question 3 Placeholder**	0	1	2	3	5
**4**	**Question 4 Placeholder**	0	1	2	3	5
**5**	**Question 5 Placeholder**	0	1	2	3	5
**6**	**Question 6 Placeholder**	0	1	2	3	5
	**Sum score (possible range XX-XX)**	__ __				

The Short Alcohol and Alcohol Problems Perception Questionnaire

	**There are no right or wrong answers. Please indicate the extent to which you agree or disagree with the following statements**	**Strongly disagree** 1	**Quite strongly disagree** 2	**Disagree** 3	**Neither agree or disagree** 4	**Agree** 5	**Quite strongly agree** 6	**Strongly agree** 7
1	I feel I know enough about causes of drinking problems to carry out my role when working with drinkers							
2	I feel I can appropriately advise my patients about drinking and its effects							
3	I feel I do not have much to be proud of when working with drinkers							
4	All in all, I am inclined to feel I am a failure with drinkers							
5	I want to work with drinkers							
6	Pessimism is the most realistic attitude to take towards drinkers							
7	I feel I have the right to ask patients questions about their drinking when necessary							
8	I feel that my patients believe I have the right to ask them questions about drinking when necessary							
9	In general, it is rewarding to work with drinkers							
10	In general, I like drinkers							

For peer review only

Annexe 25 Provider Interview

Telephone Interview of random sample of providers

Approximately 15-minute recorded telephone interview with open-ended questions

Country:

City:

PHCU ID Number:

PHC Provider ID Number:

Why?
Engagement: reasons for participating in the PHC action

How and for whom?
Description of the implementation process for screening and brief advice: description of proceedings and expectations of screening and brief advice

Under what circumstances?
What were the barriers and facilitators to following the guidelines on risky alcohol consumption?

What were the facilitators or barriers to implementing screening and brief advice?

Opinions and suggestions for organisational and political barriers and facilitators

Other thoughts and suggestions to speed up the implementation process

The responses will be analysed and coded according to Keurhorst et al. 2016:

Keurhorst M, Heinen M, Colom J et al. Strategies in primary healthcare to implement early identification of risky alcohol consumption: why do they work or not? A qualitative evaluation of the ODHIN study. Keurhorst et al. BMC Family Practice (2016) 17:70 DOI 10.1186/s12875-016-0461-8

For peer review only

SCALA – Documentation of PHCC Recruitment

1) Please specify the country as well as the name of the researcher responsible for PHCC recruitment:

**Country**	☐ Mexico<br>☐ Colombia<br>☐ Peru
**Responsible researcher**	______________________

2) During recruitment of the PHCCs, local researchers should document the following points *for each municipality*:

**Name of municipality**	______________________
**Control / Intervention**	☐ Control<br>☐ Intervention
**Total number of PHCCs in municipality**	______________________
**Number of PHCCs contacted for study participation**	______________________
**Number of non-responding PHCCs**	______________________
**Number of PHCCs refusing to participate**	______________________
**Number of PHCCs accepting to participate**	______________________

For peer review only

3) Further, the following points need to be documented *for each contacted PHCC*:

**Name/Address/Identifier of PHCC**	______________________
**Characteristics of PHCC (if known)**	☐ Number of registered patients: ______<br>☐ Number of GPs: ______<br>☐ Number of nurses: ______<br>☐ Number of all workers: ______<br>☐ other:<br>_________________________________
**Contact with PHCC**	☐ By mail<br>☐ By email<br>☐ By telephone<br>☐ Personal contact<br>☐ other:<br>_________________________________
**Number of contacts with PHCC before decision (acceptance/refusal/non-response)**	______________________
**Accepted / Refused / No response**	☐ Accepted<br>☐ Refused<br>☐ No response
**If refused, give reasons**	____<br>____<br>____
**If no response, any reasons suspected?**	____<br>____<br>____

For peer review only

SCALA – Provider follow-up documentation

Provider details

During the course of the study, each PHC provider should be followed up with regard to participation in training sessions. Further, potential drop outs should be documented here. Please fill in this sheet ***for each provider.***

**Country**	☐ Mexico<br>☐ Colombia<br>☐ Peru
**Responsible researcher**	____________________
**Name of municipality**	____________________
**Control / Intervention**	☐ Control<br>☐ Intervention
**Name/Address/Identifier of PHCC**	____________________
**Name/Identifier of provider**	____________________
**Gender of provider**	☐ Female<br>☐ Male<br>☐ Other
**Age of provider**	____________________ (in years of age)
**Baseline month**	from ____ / ____ / ____ until ____ / ____ / ____<br>(DD / MM / YY)

For peer review only

Participation in training sessions

**Training session**	☐ Pre-implementation Training 1<br>☐ Pre-implementation Training 2<br>☐ Booster 1<br>☐ Booster 2
**Date of training**	____ / ____ / ____ (DD / MM / YY)
**Training participation**	☐ Participated in training<br>☐ Absent in training
**Reason for training absence**	☐ with valid excuse, ie. ________________<br>☐ without valid excuse
**If absent at training, could training be repeated?**	☐ Yes, on ____ / ____ / ____ (DD / MM / YY)<br>☐ No

For peer review only

Drop out

If the provider dropped out before end of the study, the following section need to be filled in:

**Date of drop out**	____ / ____ / ____ (DD / MM / YY)
**Date of last tally sheet completed by provider**	____ / ____ / ____ (DD / MM / YY)
**Drop out in relation to data collection**	☐ Before baseline data collection<br>☐ During baseline data collection<br>☐ After baseline data collection, but before 18-month implementation period<br>☐ During specific month of 18-month implementation period (enter number of month from 1 to 18).
**Reasons for drop out**	____________________<br>____________________<br>____________________<br>____________________<br>____________________

For peer review only

RE-AIM Dimension, SCALA aims	SCALA activities	Main outcome/process measures
**REACH**<br>• In PHC, to maximise exposure to screening for AUD<br>• In PHC, to maximise exposure to advice and treatment for AUD and comorbid depression<br>• In PHC, to maximise exposure to alcohol health literacy information materials	• Recruitment of PHCCs in each city with large population coverage of about 160,000 registered patients per PHCC<br>• Recruitment of representative PHCC population within cities to maximise<br>– Take-up of alcohol health literacy information materials<br>– Numbers screened for AUD<br>– Numbers receiving appropriate advice/referral for AUD/depression	• Total number of PHCC patients screened for AUD<br>• Total number of screen positive patients receiving appropriate advice/referral for AUD/depression<br>• Representativeness of population screened and/or receiving appropriate advice/referral for AUD
**EFFECTIVENESS**<br>• To design and apply an evidence-based care pathway to address AUD and comorbid depression in primary health care	Design and delivery of an intervention package within a primary health care based care pathway that incorporates:<br>• State-of-the-art alcohol health literacy information materials<br>• AUDIT-C screening instrument<br>• Brief advice and treatment for case positives<br>• Referral of severe AUD and comorbid depression	• Increased health literacy in PHCC patients using UK-based Newest Vital Sign and an adapted version of Health Literacy Survey-EU Questionnaire (HLS-EU Q)<br>• Reduction in alcohol consumption of AUD+ drinkers
**ADOPTION**<br>• To increase the adoption of the intervention package in primary health care	• Design of a pragmatic, easy to use and replicate PHCC intervention package and associated care pathway<br>• Tailoring of the PHCC package according to local needs (PHC setting, PHCC) by using Community Advisory Boards (CABs) and User Panels (UPs)<br>• Provision of specific practice-based training and ongoing support to PHCC<br>• Development of city-based adoption mechanisms and support systems	• Adoption rate and representativeness of PHCCs<br>• Adoption rate and representativeness of PHCC staff
**IMPLEMENTATION**<br>• To assess the fidelity and costs of implementing the intervention package<br>• To evaluate which factors affect the implementation of the intervention package	• Continuous feedback on PHCC level drivers to package implementation gathered via qualitative and quantitative metrics<br>• Application of WHO Urban Health Equity Assessment and Response Tool<br>• Application of MRC framework to map and understand progress towards effective scale-up	• Extent primary health care screening and advice package delivered as intended<br>• Multi-level evaluation of barriers/facilitators to scale-up using WHO's Urban Health Equity Assessment and Response Tool<br>• Extent implementation on city levels delivered as intended using Medical Research Council guidance<br>• Cost of package implementation
**MAINTENANCE**<br>• To report on long-term effects of package at individual and organisational levels<br>• To understand how the programme can be maintained and achieve longevity within the test cities	• Support at the system level to make relevant practice changes for sustainability<br>• Monitoring system on long-term effectiveness<br>• Monitoring system on performance on PHCC level<br>• Production of Step-by-step SCALA Framework and Strategy	• Assessment of outcomes 18 months post implementation<br>• Indicators of program-level maintenance<br>• Measures of cost of maintenance<br>• Dissemination / events
